# Radon exposure and potential health effects other than lung cancer: a systematic review and meta-analysis

**DOI:** 10.3389/fpubh.2024.1439355

**Published:** 2024-09-25

**Authors:** Afi Mawulawoe Sylvie Henyoh, Olivier Laurent, Corinne Mandin, Enora Clero

**Affiliations:** Institut de Radioprotection et de Sûreté Nucléaire (IRSN), PSE-SANTE/SESANE/LEPID, Fontenay-aux-Roses, France

**Keywords:** radon, occupational exposure, residential exposure, cancer, non-malignant disease, cardiovascular disease, neurodegenerative disease, meta-analysis

## Abstract

**Context and objective:**

To date, lung cancer is the only well-established health effect associated with radon exposure in humans. To summarize available evidence on other potential health effects of radon exposure, we performed a comprehensive qualitative and quantitative synthesis of the available literature on radon exposure and health effects other than lung cancer, in both occupational and general populations.

**Method:**

Eligible studies published from January 1990 to March 2023, in English and French languages, were identified in PubMed, ScienceDirect, Scopus, ScieLo and HAL. In the meta-analysis, we estimated average weighted standardized incidence ratios (metaSIR), standardized mortality ratios (metaSMR), and risk ratio (metaRR) per 100 unit (Bq/m^3^ or Working level Month) increase in radon exposure concentration by combining estimates from the eligible studies using the random-effect inverse variance method. DerSimonian & Laird estimator was used to estimate the between-study variance. For each health outcome, analyses were performed separately for mine workers, children, and adults in the general population.

**Results:**

A total of 129 studies were included in the systematic review and 40 distinct studies in the meta-analysis. For most of these health outcomes, the results of the meta-analyses showed no statistically significant association, and heterogeneity was only present among occupational studies, especially between those included in the metaSIR or metaSMR analyses. However, the estimated exposure-risk associations were positive and close to the statistical significance threshold for: lymphohematological cancer incidence in children (metaRR = 1.01; 95%CI: 1.00–1.03; *p* = 0.08); malignant melanoma mortality among adults in the general population (metaRR = 1.10; 95%CI: 0.99–1.21; *p* = 0.07); liver cancer mortality among mine workers (metaRR = 1.04; 95%CI: 1.00–1.10; *p* = 0.06); intestine and rectal cancer mortality combined among mine workers (metaRR = 1.02; 95%CI: 1.00–1.04; *p* = 0.06).

**Conclusion:**

Although none of the exposure-risk associations estimated in the meta-analyses reached statistical significance, the hypothesis that radon may have other health effects apart from lung cancer could not be ruled-out and call for additional research. Larger and well-designed studies are needed to further investigate this question.

**Systematic review registration:**

https://www.crd.york.ac.uk/prospero/display_record.php?ID=CRD42023474542, ID: CRD42023474542.

## 1 Introduction

Radon is a natural radioactive noble gas originating from the decay series of uranium-238 present in rocks and soils. It is the most important source of natural background radiation (1). Epidemiological studies conducted in miners and in the general population have provided consistent evidence of the carcinogenic effect of radon on the lung (2–4). A recent systematic review and meta-analysis that included 24 single studies estimated a statistically significant 11% increase in the risk of lung cancer per 100 Becquerel/cubic meter [Bq/m^3^] increase in residential radon concentration, overall, and a 15% increased risk among lifelong never-smokers (5). A recent study conducted in the frame of the pooled uranium miners analysis (PUMA) consortium, composed of seven underground uranium miners cohorts from North America and Europe, estimated a 22% increase in lung cancer mortality risk per 100 working level months (WLM) (6). Since a few decades, a growing number of studies have investigated other potential health effects associated with radon exposure, but, individually, they did not allow for straightforward interpretations (7–9). Previously, several systematic reviews and meta-analyses were conducted on one or few diseases, and results were inconclusive (10–14). Most of these reviews and meta-analyses focused on only one type of radon exposure, occupational or residential, and were restricted to the child or adult population. To overcome these limitations, we carried out a comprehensive and up-to date systematic review and meta-analysis, covering both occupational and residential radon exposure, populations of children and adults, and incidence and mortality data for a wide range of malignant and non-malignant diseases, except lung cancer. This work was performed in the frame of the European project RadoNorm,^1^ which aims to manage risks from radon and Naturally-Occurring Radioactive Materials exposure situations to ensure effective radiation protection based on improved scientific evidence and social considerations.

## 2 Materials and methods

This work was carried out and reported in accordance with the Preferred Reporting Items for Systematic reviews and Meta-Analyses (PRISMA) (15, 16), and has been registered in the PROSPERO databases^2^ under the identification number CRD42023474542.

### 2.1 Information sources and search strategy

A comprehensive literature search was performed in March 2023 in five databases: PubMed, ScienceDirect, Scopus, ScieLo and HAL. The following bibliographic query, developed in collaboration with a professional librarian, was used: *(“Radon exposure”) OR (“Exposure to radon”) OR (“Exposure of radon”) OR (“Exposure to Rn”) OR (“Exposure of Rn”) OR (“Exposed to radon”) OR (“Residential radon”) OR (“Radon concentration”) OR (“Working level month”) OR (“WLM”)*. We did not specify disease names on purpose, to enable identification of published articles on all possible malignant and non-malignant diseases in association with radon exposure. We applied restrictions to the language (English and French) and the period of publication (from 1990 to the time of the search in March 2023). Finally, we uploaded the identified references into a platform called RAYYAN,^3^ which is a cloud-based software application designed for researchers conducting systematic literature reviews and meta-analyses.

### 2.2 Eligibility criteria

Studies were included if all of the following criteria were fulfilled: (1) they focused on occupational exposure to radon such as in miners or on residential (air or water) exposure to radon in the general population, in children and/or adults; (2) the control group, except in case–control studies, was composed of persons with lower (ideally, minimal) levels of exposure to radon (internal control group) or representing a given reference population (for instance, miners inside a country were often compared with the national population from the same country); (3) the outcome of interest was morbidity (incidence/prevalence) or mortality due to any malignant and non-malignant disease excluding lung cancer; (4) the study design was a single or a pooled original cohort, case–control, case-cohort, cross-sectional, or ecological study.

Studies were excluded when: (1) there was lack of data specific to radon exposure history; (2) there was no ability to disentangle radon exposure from exposure to other sources of ionizing radiations; (3) the outcome of interest was overall cancer, i.e., including lung cancer; (4) the design was case-report, systematic review, and meta-analysis of original studies; (5) only simulated data were analyzed.

If several studies focused on the same population (with total or partial overlap), we only retained the study with the longest follow-up period or the largest sample size. Also, pooled studies were preferred to single studies.

### 2.3 Studies’ selection and data extraction process

**Figure 1:**
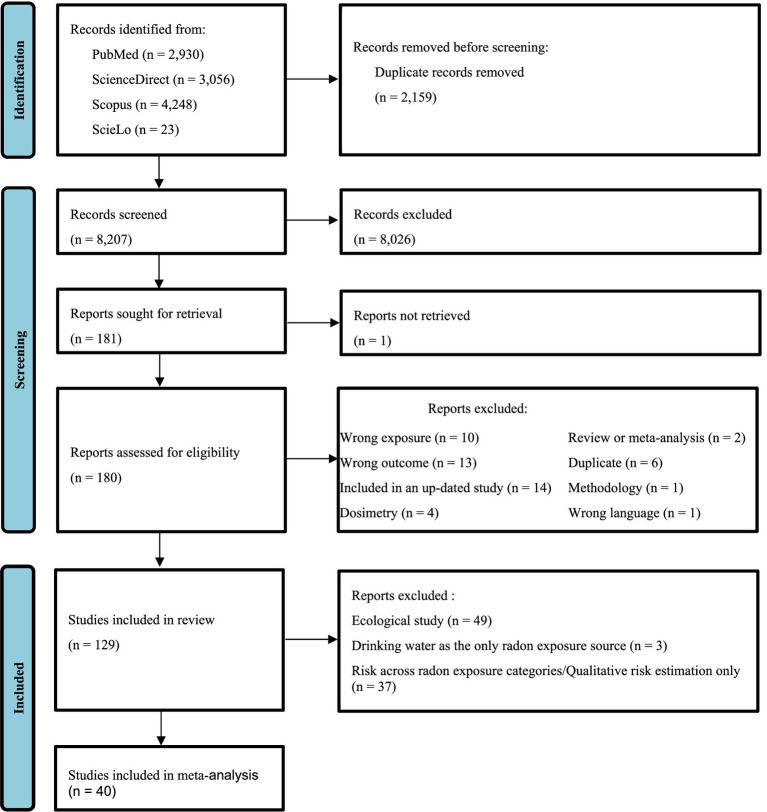
PRISMA flow diagram for the systematic review and meta-analysis.

After removal of duplicates, two authors (A.H. and E.C.) independently screened titles and if needed abstracts with regards to the eligibility criteria. Obviously irrelevant records were excluded. Disagreements between the two authors were resolved throughout discussions, and if necessary, the opinion of the third author (O.L.) was obtained. Full texts of the remaining potentially eligible studies were retrieved and carefully examined by one author (A.H.) for final inclusion or exclusion, and the other authors (E.C. and O.L.) were consulted in case of uncertainty. The reasons of exclusion at this stage were reported in Figure 1 and in Supplementary Table S1.

One author (A.H.) extracted relevant information from the included articles onto a spread sheet, including publication data (first author, year of publication, and location of investigation), follow-up period, study design, sample size, number of cases, and if relevant, number of controls, any other study population characteristics, health outcome(s) studied, characteristics of radon exposure assessment, variables included in statistical analyses to control for potential confounding, and main results.

If a study reported both incidence and mortality data, both were extracted. Results from multivariate/fully adjusted models were preferred for extraction to results from crude or more sparsely adjusted models. The overall study population results were preferred for extraction to the stratified ones (e.g., results per population subgroups). When several outcomes were studied and/or several effect measures were used within a same study, we extracted each of the results with a careful attention to avoid duplication or overlap with any other study.

### 2.4 Quality assessment of the included studies

One author (A.H.) assessed the quality of the included studies by using on the one hand, the New-castle Ottawa Scale (NOS) for cohort, case–control, and cross-sectional studies.^4^ On the other hand, an evaluation tool proposed by the United Nations Scientific Committee on the Effects of Atomic Radiation (UNSCEAR) in its 2017 report was also used (17). This UNSCEAR tool addresses methodological issues specific to radiation epidemiology studies, and applies to all the included studies, including the ecological ones which are not considered by the NOS scale. To control for potential subjectivity biases, a training and validation session was set during which two authors (A.H. and E.C.) independently assessed the quality of four randomly selected studies with different designs, using the two quality assessment tools. The overall and subdimension quality scores/tiers were compared, and any discordance was discussed to derive consolidated decision rules. The overall NOS score ranges from 0 to 9 for cohort and case–control studies, and from 0 to 10 for cross-sectional studies. Studies that obtained an overall NOS score of at least 6 (or at least 7 for cross-sectional studies) were considered to be of “high” quality. Those with an overall NOS score of 4–5 (or 5–6 for cross-sectional studies) were considered to be of “moderate” quality, otherwise, they were considered to be of “low” quality. The UNSCEAR quality assessment tool is composed of eight domains for which a study is appraised and judged to be of “very low, low, moderate or high” quality. To determine the overall quality tier of a study, we assigned a sub-score from 1 to 4 to each domain according to its quality tier. The sub-scores were then averaged, and the overall quality tier of the study was judged to be “very low, low, moderate, high” when the average score was “≤1.5, >1.5–2.5, >2.5–3.5, ≥3.5,” respectively.

### 2.5 Meta-analysis

#### 2.5.1 Additional eligibility criteria specific to the meta-analysis step

Further criteria were defined for including studies in the meta-analysis. Studies that did not provide quantitative estimate of the effect were excluded. We also excluded ecological studies given their limitation to transpose their results at individual level. Studies that considered only drinking water as radon exposure source were excluded. The meta-analyses were limited to studies in which radon exposure estimates were treated in the regression model as a continuous variable since we did not plan to perform so-called “dose–response meta-analysis” combining results from categorical analyses to derive estimates for continuous exposure variables (18, 19) given the related uncertainties and the very large number of analyses to be done. No exclusion was made based on studies’ quality, since all the eligible studies showed at least moderate quality based on the NOS and UNSCEAR quality assessment tools (see Supplementary Tables S2, S3).

The measures of effect of interest were Standardized Incidence Ratio (SIR), and Standardized Mortality Ratio (SMR), Incidence Rate Ratio or Relative Risk (IRR), Excess Relative Risk (ERR), Odds Ratio (OR), and Hazard Ratio (HR). Whenever needed, exposure-risk relationships estimates were converted to Risk Ratio (RR) (10) and pooled together in this way, assuming they yield similar risk estimate under appropriate conditions (for instance, for ORs, rare health outcomes and true RR less than 2) (20, 21).

An additional exclusion criterion was applied to studies that reported SIRs and SMRs estimates: we excluded results/studies for which the value of SIR or SMR was null because the logarithm reached infinity, and therefore could not be properly handled in the analyses.

#### 2.5.2 Data extraction and management for the meta-analysis

Estimates and their 95% confidence interval (CI) and/or *p*-value were retrieved from the eligible articles.

Regarding SIRs and SMRs, the 95%CIs were not provided in some cases. We then estimated them using Vanderbroucke method (22) with $95\%\mathrm{CI}=\frac{{\left(\sqrt{a}\pm Z_{1-\alpha /2}\times 0.5\right)}^{2}}{\lambda }$ where a is the number of observed cases, *λ* the number of expected cases, *Z* the value of a unit-normal test statistic corresponding to *α*, the probability of a type 1 error (here, *α* = 0.05, meaning *Z* = 1.96 for a two-tailed test). For mine workers studies, risk estimates were all expressed for 100 WLM increase in radon concentration. For residential exposure studies, risk estimates for 100 Bq/m^3^ increase in radon concentration were preferred to those for 10 or 1,000 Bq/m^3^, and where necessary we computed the corresponding risk estimates for 100 Bq/m^3^ increase.

In three studies (23–25), either IRR or OR per 1,000 Bq/m^3^-years were reported. We then computed the corresponding RR per 100 Bq/m^3^ using the following equation $RR_{100}=e^{\left(\log\left(RR_{1000}\right)/10\right)}$ . In one study (26) a HR per 10 Bq/m^3^ was reported, we then computed the corresponding RR per 100 Bq/m^3^ using the following equation $RR_{100}=e^{\left(\log\left(RR_{10}\right)\times 10\right)}$ .

As much as possible, risk estimates from non-linear risk models were preferred to linear risk models because risk estimates that have been derived from linear risk models are more challenging to combined in a meta-analytic way due to difficulty of existing statistical methods to reasonably quantify study-specific variances. Richardson et al. (27) recently proposed an alternative approach to address this challenge, but it requires to know the maximum concentration recorded in each included study, which is not systematically reported. When only ERR was provided, the RR was computed based on the equation RR=1+ERR (28). This was also applied to 95%CI bounds. In some cases, the lower bound (*l*) of the 95%CI was not available, but the upper bound (*u*) was provided (29–32). We then estimated the lower bound using the equation l=2×ERR−u , assuming the 95%CI was symmetric. When only the point estimate of the ERR and its associated *p*-value were given (29–35), the bounds of the 95%CI were computed based on the Wald statistic $95\%\mathrm{CI}=ERR\pm Z_{\alpha /2}\times \left(\frac{ERR}{Z_{p}}\right)$ where *α* = 0.05, $Z_{\alpha /2}=1.96$ , and $Z_{p}$ , the value of Z that corresponds to the associated *p*-value of the point estimate.

#### 2.5.3 Health outcomes definition

In most studies, diseases or group of diseases were defined using the international classification of diseases (ICD). A careful attention was given to the ICD codes reported in the articles to ensure that only studies using a similar definition for a given disease were pooled together. When a study did not focus on a given disease, but rather on one of its subtypes, we included it as such. In addition, where appropriate, we aggregated different diseases to form relatively broad and homogeneous disease groups.

#### 2.5.4 Statistical analysis

We investigated heterogeneity across studies using Cochrane’s *Q* test and the I-square index. A *Q* test with *p*-value of less than 0.1 was considered as “detecting heterogeneity,” and an I-square value about 25, 50% or 75% represented low, moderate or high heterogeneity, respectively (36).

We estimated average weighted SIR (metaSIR), average weighted SMR (metaSMR), and average weighted RR (metaRR) by combining at least two estimates from studies using the random-effect inverse variance method regardless of the heterogeneity tests results. DerSimonian & Laird estimator was used to estimate the between-study variance *τ*^2^ (37). For each health outcome, analyses were performed separately for mine workers exposed to radon, expressed in WLM, for children and for adults in the general population exposed to radon, expressed in annual average concentration in Bq/m^3^. Further, incidence and mortality data were analyzed separately.

We examined small-study effects and publication bias using Egger’s regression test (38) and Begg’s funnel plot (39). The existence of a publication bias was suspected if the *p*-value for Egger’s regression test was less than 0.05 and/or if Begg’s funnel plot showed an asymmetric shape.

In sensitivity analyses, for all metaRR close to the statistical significance threshold and whenever possible (i.e., with at least three studies), we investigated whether the result was driven by specific studies or estimates using the leave-one-out method (40). We also repeated all the average weighted effects estimation (metaSIR, metaSMR, and metaRR) using the fixed-effects inverse variance method.

All the statistical models were fitted using the meta and metafor packages in R, version 4.2.2.

## 3 Results

### 3.1 Systematic review

#### 3.1.1 Literature search and selection results

In total 10,366 bibliographic references were identified from the electronic databases, and 129 were included in the review. Details about the selection process are shown in Figure 1.

#### 3.1.2 Characteristics of the included studies

Regarding the exposure type, 43 studies focused on occupational radon exposure (9, 26, 29–35, 41–74) and 86 on residential radon exposure. Of these last ones, 24 were restricted to children (7, 23–25, 75–94) and 55 to adults in the general population (8, 95–148). The remaining seven studies included both children and adults from the general population (149–155), with results presented separately for children and adults in six of them. Regarding the design, studies on occupational radon exposure were predominantly cohorts (86%, *n* = 37), while those on residential radon exposure in adults were predominantly ecological studies (60%, *n* = 37); and finally, in children, most of the studies were ecological (50%, *n* = 15), followed by case–controls studies (30%, *n* = 9). In terms of geographical repartition, the studies were carried out worldwide, mainly in North America, Europe, and Asia. In terms of health outcomes, except for studies in children among which incidence data were mostly used, mortality data outweighed incidence data across studies including mine workers and adults in the general population.

**Figure 2:**
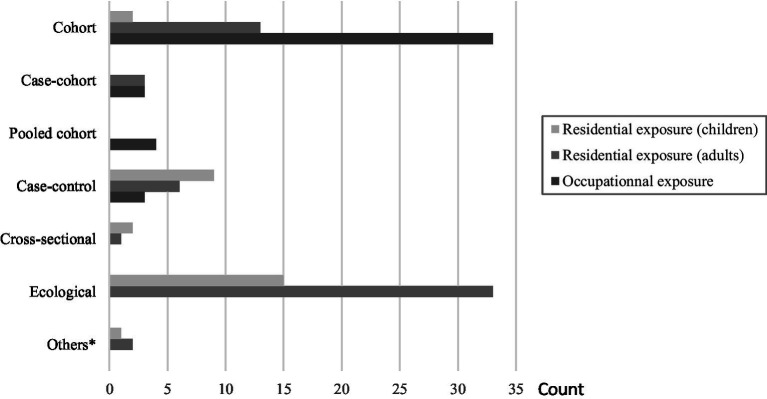
Repartition of studies included in the systematic review and meta-analysis by exposure type and design. *“Two-design in one” studies: ecological study & case-control study; Ecological study & case-only study; Ecological study & cohort study.

Detailed information about the included studies, their repartition by design, exposure and population types, and their main findings are reported in Figure 2 and Supplementary Tables S2, S3. A qualitative summary of the studies’ results is provided in Supplementary Tables S4–S7, showing for each health outcome, the number of studies reporting no, negative, or positive statistically significant association. Overall, there was an apparently good agreement between findings for occupational and residential radon exposure, and, subsequently, between children and adults in the general population, with respect to the health outcomes that were studied in common in these different settings, especially regarding lymphohematological cancers which were the most common health outcomes. In most cases, results of individual studies pointed toward a lack of statistically significant association.

### 3.2 Meta-analyses

In total, 40 distinct studies were included in the meta-analysis, and the number of single estimates of SIR/SMR and RR included in the analyses for each health outcome studied was up to 34 and 7, respectively (Tables 1, 2). The quality score of studies ranged from moderate to high for both NOS and UNSCEAR quality assessment tools. Results of the meta-analysis are reported in Table 1 for SIRs and SMRs, and Table 2 for exposure-risk relationships.

**Table 1:** Results of the meta-analyses for SIRs and SMRs of malignant and non-malignant health outcomes, except lung cancer, among mine workers using the random effect of DerSimonian & Laird (DL).

Health outcome		Number of estimates included in the meta-analysis (reference)^α^	Total cases/total sample size	Country	Meta SIR/SMR (95%CI)	*p* value for the meta SIR/SMR	Cochran’s *Q*-test *p* for residual heterogeneity	I-square value (%) for residual heterogeneity (95%CI)	Egger’s test p for publication bias*
Lymphohematological cancer									
	Incidence	9 (30, 46, 49)	599/193,734	Canada, Czech Republic	0.860 (0.748–0.988)	**0.033**	**0.009**	60.717 (18.492–81.067)	0.403
	Mortality	22 (26, 61, 63, 64, 66, 69, 72)	1,141/514,368	USA & Canada & France & Germany & Czech Republic, Brazil, USA, Italy, Sweden, UK	1.067 (0.956–1.192)	0.248	**0.019**	42.552 (4.72–65.362)	**0.001**
Leukemia									
	Incidence	3 (30, 46, 49)	206/60,759	Czech Republic, Canada	0.985 (0.693–1.399)	0.932	**0.003**	82.877 (47.764–94.387)	0.513
	Mortality	8 (26, 61, 63, 64, 69, 72)	468/134,223	USA & Canada & France & Germany & Czech Republic, Brazil, USA, Sweden, UK	1.120 (0.901–1.392)	0.308	0.130	37.505 (0.000–72.397)	0.170
Chronic lymphocytic leukemia		
	Mortality	3 (49, 62, 69)	27/33,805	Canada, USA	1.252 (0.704–2.227)	0.443	0.150	47.370 (0.000–84.580)	0.429
Leukemia excluding chronic lymphoblastic leukemia		
	Mortality	5 (29, 49, 53, 62, 69)	178/74,409	Canada, France, USA, Germany	1.025 (0.801–1.312)	0.843	0.059	55.891 (0.000–83.685)	0.135
Myeloid leukemia	
	Mortality	2 (48, 53)	69/39,524	Germany, Czech Republic	0.963 (0.762–1.217)	0.754	0.900	0.000 (NA–NA)	NA
Lymphoma	
	Incidence	7 (30, 46, 49)	308/137,952	Czech Republic, Canada	0.891 (0.748–1.063)	0.200	0.092	44.953 (0.000–76.827)	0.753
	Mortality	12 (26, 61, 64, 66, 69)	464/260,099	USA & Canada & France & Germany & Czech Republic, USA, Italy, Sweden	1.097 (0.912–1.32)	0.325	0.092	37.453 (0.000–68.382)	**0.039**
Hodgkin lymphoma	
	Incidence	3 (30, 46, 49)	48/60,759	Czech Republic, Canada	0.980 (0.563–1.706)	0.943	**0.013**	76.991 (25.008–92.940)	0.667
	Mortality	5 (26, 61, 64, 69)	77/127,588	USA & Canada & France & Germany & Czech Republic, USA, Sweden	1.223 (0.745–2.01)	0.426	0.130	43.800 (0.000–79.367)	0.383
Non-Hodgkin lymphoma	
	Incidence	4 (30, 46, 49)	260/77,193	Czech Republic, Canada	0.850 (0.754–0.958)	0.008	0.838	0.000 (0.000–84.688)	0.861
	Mortality	7 (26, 61, 64, 66, 69)	387/132,511	USA & Canada & France & Germany & Czech Republic, Italy, Sweden, USA	1.109 (0.873–1.407)	0.397	0.108	42.410 (0.000–75.789)	0.089
Multiple myeloma	
	Incidence	3 (30, 46, 49)	85/60,759	Czech Republic, Canada	0.955 (0.544–1.676)	0.872	**0.001**	85.576 (57.786–95.071)	0.809
	Mortality	5 (26, 61, 64, 69)	193/127,588	USA & Canada & France & Germany & Czech Republic, USA, Sweden	1.194 (0.815–1.751)	0.363	**0.036**	61.168 (0.000–85.416)	0.165
Brain and central nervous system cancer		
	Incidence	3 (32, 46, 49)	92/48,393	Czech Republic, Canada	0.821 (0.677–0.997)	0.046	0.450	0.000 (0.000–89.598)	0.298
	Mortality	4 (61, 64, 66, 69)	335/126,877	USA & Canada & France & Germany & Czech Republic, Italy, Sweden, USA	1.367 (0.815–2.292)	0.236	**<0.001**	83.333 (57.629–93.444)	0.133
Brain cancer	
	Incidence	2 (32, 46)	22/19,434	Czech Republic, Canada	0.975 (0.64–1.487)	0.907	0.259	21.587 (NA–NA)	NA
	Mortality	2 (32, 46)	18/19,079	Czech Republic, Canada	0.756 (0.511–1.117)	0.160	0.956	0.000 (NA–NA)	NA
Malignant melanoma	
	Incidence	3 (32, 46, 49)	70/48,393	Czech Republic, Canada	0.682 (0.314–1.48)	0.333	**<0.001**	91.194 (77.208–96.598)	0.202
	Mortality	7 (41, 46, 48, 49, 53, 62, 69)	108/93,018	USA, Czech Republic, Canada, Germany	1.075 (0.826–1.399)	0.591	0.097	44.021 (0.000–76.45)	0.181
Non-melanoma skin cancer		
	Mortality	6 (41, 46, 48, 53, 64)	19/61,274	USA, Czech Republic, Germany, Sweden	1.609 (0.683–3.788)	0.276	**0.002**	73.830 (40.234–88.541)	0.310
Extra-thoracic airways cancer		
	Incidence	5 (32, 46, 49)	320/80,352	Canada, Czech Republic	0.904 (0.696–1.173)	0.447	**0.001**	77.732 (46.352–90.757)	0.756
	Mortality	11 (29, 48, 53, 61, 63, 66, 69)	621/417,275	Germany, France, USA & Canada & France & Germany & Czech Republic, Brazil, Italy, USA, Czech Republic	0.899 (0.771–1.049)	0.175	**0.039**	47.638 (0.000–73.899)	0.861
Nose cancer	
	Mortality	3 (29, 48, 53)	23/44,924	Germany, France, Czech Republic	1.274 (0.855–1.899)	0.233	0.903	0.000 (0.000–89.598)	0.332
Laryngeal cancer									
	Incidence	3 (32, 46, 49)	139/48,393	Czech Republic, Canada	1.074 (0.757–1.524)	0.690	**0.025**	73.022 (9.342–91.972)	0.810
	Mortality	4 (61, 63, 66, 69)	246/128,439	USA & Canada & France & Germany & Czech Republic, Brazil, Italy, USA	1.071 (0.94–1.221)	0.300	0.454	0.000 (0.000–84.688)	**0.042**
Buccal and pharyngeal cancer		
	Incidence	3 (32, 46, 49)	187/48,393	Czech Republic, Canada	0.803 (0.62–1.041)	0.098	0.134	50.182 (0.000–85.581)	0.635
	Mortality	4 (61, 66, 69)	352/243,912	USA & Canada & France & Germany & Czech Republic, Italy, USA	0.795 (0.717–0.883)	**<0.001**	0.780	0.000 (0.000–84.688)	0.350
Tongue and mouth cancer	
	Mortality	2 (46, 48)	12/20,754	Czech Republic	1.17 (0.638–2.146)	0.611	0.244	26.429 (NA–NA)	NA
*Lip cancer*									
	Incidence	2 (32, 46)	11/19,434	Czech Republic, Canada	0.737 (0.455–1.194)	0.215	0.763	0.000 (NA–NA)	NA
Thyroid and other endocrine gland cancer		
	Mortality	5 (46, 49, 53, 62, 69)	21/85,443	Czech Republic, Canada, Germany, USA	1.017 (0.688–1.503)	0.933	0.913	0.000 (0.000–79.204)	0.756
Thyroid cancer	
	Incidence	2 (46, 49)	22/45,393	Czech Republic, Canada	0.803 (0.412–1.565)	0.519	0.087	65.808 (0.000–92.238)	NA
	Mortality	3 (46, 49, 53)	19/80,184	Czech Republic, Canada, Germany	1.008 (0.667–1.524)	0.968	0.802	0.000 (0.000–89.598)	0.797
Digestive cancer	
	Incidence	9 (32, 46, 49)	1,228/144,270	Canada, Czech Republic	0.838 (0.655–1.073)	0.161	**<0.001**	91.928 (86.911–95.022)	0.529
	Mortality	34 (26, 61, 63, 64, 66, 68, 69, 72)	4,546/782,389	USA & Canada & France & Germany & Czech Republic, Brazil, USA, China, Italy, Sweden, UK	1.009 (0.938–1.085)	0.811	**<0.001**	60.212 (42.110–72.653)	0.976
Esophagus cancer	
	Incidence	3 (32, 46, 49)	77/48,393	Czech Republic, Canada	0.964 (0.626–1.486)	0.869	0.049	66.788 (0.000–90.416)	0.880
	Mortality	6 (61, 63, 64, 66, 68, 69)	391/136,177	USA & Canada & France & Germany & Czech Republic, Brazil, Italy, USA, China, Sweden	0.914 (0.822–1.016)	0.095	0.543	0.000 (0.000–74.625)	0.676
Stomach cancer	
	Incidence	3 (32, 46, 49)	248/48,393	Czech Republic, Canada	0.969 (0.634–1.481)	0.884	**<0.001**	89.684 (72.225–96.169)	0.841
	Mortality	10 (26, 61, 63, 64, 66, 68, 69, 72)	1,222/145,407	USA & Canada & France & Germany & Czech Republic, USA, Brazil, Italy, China, Sweden, UK	1.119 (0.949–1.32)	0.182	0.052	46.347 (0–74.174)	0.825
Liver and gallbladder cancer		
	Mortality	6 (61, 64, 66, 68, 69)	599/134,615	Italy, China, USA & Canada & France & Germany & Czech Republic, Sweden, USA	0.969 (0.701–1.339)	0.850	**0.028**	60.139 (2.207–83.752)	0.399
Liver cancer	
	Incidence	2 (46, 49)	75/45,393	Czech Republic, Canada	1.095 (0.459–2.613)	0.837	**<0.001**	93.884 (80.475–98.084)	NA
	Mortality	8 (29, 46, 48, 49, 53, 64, 66, 68)	356/102,382	Czech Republic, Canada, France, Italy, China, Germany, Sweden	1.200 (0.969–1.485)	0.094	**0.003**	68.123 (33.081–84.815)	0.430
Gallbladder cancer	
	Mortality	5 (46, 48, 53, 54, 64)	96/61,306	Czech Republic, Germany, Sweden	1.118 (0.796–1.569)	0.520	0.058	56.237 (0.000–83.8)	0.512
Pancreatic cancer	
	Incidence	3 (32, 46, 49)	169/48,393	Canada, Czech Republic	0.998 (0.708–1.405)	0.989	0.017	75.618 (19.601–92.606)	0.990
	Mortality	4 (61, 64, 66, 69)	687/126,877	USA & Canada & France & Germany & Czech Republic, Italy, USA, Sweden	0.940 (0.795–1.112)	0.471	0.287	20.539 (0.000–87.833)	0.485
Intestine and rectal cancer		
	Mortality	12 (26, 61, 63, 64, 66, 69)	1,632/258,273	USA & Canada & France & Germany & Czech Republic, USA, Brazil, Italy, Sweden	0.963 (0.863–1.075)	0.505	0.066	41.297 (0.000–70.209)	0.655
Intestine cancer	
	Mortality	7 (26, 61, 64, 69)	1,044/128,540	USA & Canada & France & Germany & Czech Republic, USA, Sweden	0.936 (0.843–1.040)	0.217	0.345	11.047 (0.000–74.033)	0.386
Colorectal cancer	
	Incidence	5 (32, 46, 49)	739/67,827	Canada, Czech Republic	0.959 (0.690–1.333)	0.805	**<0.001**	93.059 (86.752–96.363)	0.456
Colon cancer	
	Incidence	2 (32, 46)	113/19,434	Canada, Czech Republic	0.975 (0.777–1.224)	0.830	0.217	34.464 (NA–NA)	NA
	Mortality	5 (32, 46, 54, 62, 69)	154/28,392	Czech Republic, Canada, USA, Germany	0.855 (0.711–1.028)	0.096	0.224	29.638 (0.000–72.755)	0.281
Rectal cancer	
	Incidence	2 (32, 54)	141/19,434	Czech Republic, Canada	1.190 (0.790–1.792)	0.405	0.059	71.877 (0.000–93.673)	NA
	Mortality	3 (61, 64, 69)	575/122,137	USA & Canada & France & Germany & Czech Republic, USA, Sweden	1.157 (0.737–1.816)	0.527	**0.038**	69.474 (0.000–91.095)	0.576
Kidney, ureter, other urinary organs cancer	
	Incidence	3 (32, 46, 49)	154/48,393	Canada, Czech Republic	0.684 (0.492–0.949)	0.023	**0.036**	69.922 (0.000–91.206)	0.772
	Mortality	7 (26, 61, 64, 66, 69)	436/133,097	USA & Canada & France & Germany & Czech Republic, Italy, Sweden, USA	0.983 (0.840–1.150)	0.830	0.364	8.404 (0.000–73.262)	0.596
Kidney cancer	
	Incidence	2 (32, 49)	105/31,959	Canada	0.584 (0.411–0.832)	0.003	0.256	22.644 (NA–NA)	NA
	Mortality	6 (26, 61, 64, 69)	429/128,357	USA & Canada & France & Germany & Czech Republic, Sweden, USA	0.976 (0.800–1.190)	0.809	0.309	16.259 (0.000–78.75)	0.781
Bladder and other urinary organ cancer		
	Incidence	3 (32, 46, 49)	280/48,393	Czech Republic, Canada	0.882 (0.632–1.230)	0.458	**0.003**	83.309 (49.393–94.495)	0.321
	Mortality	7 (26, 61, 64, 66, 69)	479/133,097	Italy, USA, USA & Canada & France & Germany & Czech Republic, Sweden	1.051 (0.789–1.399)	0.734	**0.034**	56.098 (0.000–81.124)	0.115
Bladder cancer	
	Incidence	2 (32, 46)	80/19,434	Czech Republic, Canada	1.014 (0.827–1.244)	0.891	0.506	0.000 (NA–NA)	NA
	Mortality	7 (29, 32, 46, 48, 53, 54, 66)	288/72,797	Czech Republic, France, Canada, Italy, Germany	1.063 (0.953–1.186)	0.273	0.982	0.000 (0.000–70.809)	**0.022**
Testis and other male genital organ cancer excluding prostate cancer			
	Mortality	5 (46, 48, 49, 53, 69)	NA/87,018	Czech Republic, Canada, Germany, USA	0.831 (0.593–1.164)	0.281	0.848	0.000 (0.000–79.204)	0.875
Testis cancer	
	Incidence	2 (46, 49)	28/45,393	Czech Republic, Canada	0.660 (0.408–1.068)	0.091	0.148	52.32 (0–88.065)	NA
	Mortality	4 (46, 48, 49, 53)	NA/84,504	Czech Republic, Canada, Germany	0.834 (0.591–1.176)	0.300	0.713	0.000 (0.000–84.688)	0.942
Prostate cancer	
	Incidence	3 (32, 46, 49)	761/48,393	Czech Republic, Canada	0.759 (0.520–1.109)	0.154	**<0.001**	93.366 (83.984–97.252)	0.124
	Mortality	5 (61, 63, 64, 66, 69)	952/129,733	USA & Canada & France & Germany & Czech Republic, Brazil, Italy, USA, Sweden	0.958 (0.787–1.166)	0.670	0.103	48.042 (0.000–80.962)	0.387
Bone cancer	
	Mortality	5 (41, 48, 49, 53, 69)	NA/73,839	USA, Canada, Germany, Czech Republic	1.086 (0.542–2.176)	0.817	**0.040**	60.216 (0.000–85.107)	0.300
Connective and other soft tissue cancer		
	Incidence	2 (46, 49)	22/45,393	Czech Republic, Canada	0.626 (0.429–0.915)	0.016	0.593	0.000 (NA–NA)	NA
	Mortality	5 (46, 48, 49, 53, 69)	30/87,018	Czech Republic, Canada, USA, Germany	0.855 (0.616–1.187)	0.350	0.422	0.000 (0.000–79.204)	0.073
Breast cancer	
	Mortality	3 (41, 49, 62)	NA/34,546	USA, Canada	0.967 (0.434–2.157)	0.935	0.403	0.000 (0.000–89.598)	0.206
Chronic obstructive pulmonary disease & asthma	
	Mortality	6 (26, 40, 61, 69)	1,912/131,084	USA & Canada & France & Germany & Czech Republic, USA	0.950 (0.769–1.174)	0.635	**0.002**	74.003 (40.703–88.603)	0.879
Chronic obstructive pulmonary disease		
	Mortality	4 (26, 61)	1,844/124,549	USA & Canada & France & Germany & Czech Republic, USA	0.892 (0.689–1.154)	0.384	**0.006**	76.113 (34.349–91.309)	0.628
Bronchitis, emphysema, and asthma		
	Mortality	4 (40, 42, 62, 69)	159/12,518	USA	1.638 (0.936–2.866)	0.084	**<0.001**	91.787 (82.417–94.565)	0.878
All circulatory system disease		
	Mortality	6 (61, 63, 66, 69, 71, 73)	18,643/133,600	USA & Canada & France & Germany & Czech Republic, Brazil, Italy, Finland, USA, Canada	0.921 (0.823–1.031)	0.154	**<0.001**	90.225 (84.509–95.458)	0.719
Ischemic heart disease	
	Mortality	5 (61, 63, 69, 71, 72)	10,289/127,306	USA & Canada & France & Germany & Czech Republic, Brazil, Finland, UK, USA	1.062 (0.923–1.222)	0.399	**<0.001**	88.711 (76.322–94.617)	0.171
Cerebrovascular disease/stroke		
	Mortality	11 (29, 32, 41, 46, 49, 53, 54, 62, 63, 69)	2,383/104,420	USA, Czech Republic, Canada, France, Brazil, Germany	0.853 (0.657–1.108)	0.235	**<0.001**	96.657 (95.344–97.600)	0.166
Hypertension	
	Mortality	4 (32, 41, 63)	35/9,523	USA, Brazil, Canada	0.891 (0.375–2.112)	0.793	**<0.001**	84.346 (60.757–93.755)	0.223
Diabetes mellitus	
	Mortality	8 (32, 41, 46, 62, 63, 66, 69)	175/35,956	USA, Czech Republic, Canada, Brazil, Italy	0.859 (0.681–1.084)	0.201	**0.015**	59.571 (11.908–81.445)	0.687
Digestive disorders	
	Mortality	4 (61, 63, 66, 69)	2,613/128,439	USA, USA & Canada & France & Germany & Czech Republic, Brazil, Italy	0.804 (0.653–0.990)	**0.040**	**0.016**	70.904 (16.875–89.816)	0.173
Cirrhosis and other liver disease		
	Mortality	4 (61, 63, 66, 69)	1,502/128,439	USA & Canada & France & Germany & Czech Republic, Brazil, USA, Italy	0.768 (0.554–1.063)	0.112	**0.001**	81.839 (52.943–92.991)	0.196
Mental and behavioral disorders		
	Mortality	6 (41, 46, 53, 62, 69, 70)	284/64,472	Czech Republic, USA, Germany	1.535 (0.902–2.613)	0.114	**<0.001**	92.735 (86.913–95.967)	0.102
Nervous system and sense organ disorders		
	Mortality	7 (32, 41, 46, 53, 62, 69)	330/63,564	Czech Republic, Canada, USA, Germany	0.962 (0.752–1.231)	0.758	**<0.001**	75.163 (47.359–88.281)	0.353

^α^One reference can contribute to the meta-analysis with more than one estimate depending on if estimates were available for different subtypes of the health outcome of interest, or for subgroups of the study population by sex, race, pay-roll status, etc. …. *NA was reported when the number of studies included in the meta-analysis is 2; NA, Not available; SIR, Standardize incidence ratio; SMR, Standardize mortality ratio; CI, Confidence interval. Bold values mean statistically significant values.

**Table 2:** Results of meta-analyses on exposure-risk relationships between radon exposure and malignant and non-malignant health outcomes, except lung cancer, among children, adults in the general population and mine workers, using random effect of DL.

Health outcome		Number of estimates included in the meta-analysis (reference)^α^	Total cases/total sample size	Region	MetaRR per 100 Bq/m^3β^ or 100 WLM^λ^ (95%CI)	*p* value for the metaRR	Cochran’s *Q*-test *p* for residual heterogeneity	I-square value (%) for residual heterogeneity (95%CI)	Egger’s test *p* for publication bias*
Lymphohematological cancer		
	Incidence among children	7 (23–25, 79, 85, 88)	17,106/2,069,256	Switzerland, Finland, Norway, France, Denmark, UK	1.014 (0.998–1.031)	0.083	0.890	0.000 (0.000–70.809)	0.106
	Incidence among mine workers	3 (30, 49, 55)	653/45,469	Canada, Germany	0.998 (0.951–1.047)	0.923	0.339	7.616 (0.000–90.390)	0.492
	Mortality among mine workers	6 (30, 47, 49, 52)	545/23,8,177	Czech Republic, Germany, Canada	1.011 (0.982–1.04)	0.473	0.944	0.000 (0.000–74.625)	0.862
Leukemia
	Incidence in children	6 (23–25, 79, 85, 88)	14,787/2,063,663	Switzerland, Finland, Norway, France, Denmark, UK	1.014 (0.996–1.033)	0.116	0.806	0.000 (0.000–74.625)	0.152
	Incidence among mine workers	4 (30, 49, 55)	545/60,835	Canada, Germany	0.993 (0.972–1.014)	0.502	0.430	0.000 (0.000–84.688)	0.781
	Leukemia mortality among mine workers	5 (30, 47, 49, 52)	301/136,637	Germany, Canada, Czech Republic	1.006 (0.968–1.046)	0.753	0.892	0.000 (0.000–79.204)	0.752
Chronic lymphocytic leukemia		
	Incidence among mine workers	3 (30, 49, 55)	227/44,477	Canada, Germany	0.991 (0.960–1.022)	0.563	0.731	0.000 (0.000–89.598)	0.910
	Mortality among mine workers	2 (47, 49)	29/44,980	Czech Republic, Canada	0.378 (0.032–4.494)	0.441	0.008	85.751 (42.766–96.453)	NA
Leukemia excluding Chronic lymphocytic leukemia		
	Incidence among mine workers	3 (30, 49, 55)	245/44,477	Canada, Germany	1.007 (0.898–1.13)	0.906	0.140	49.057 (0.000–85.189)	0.551
	Mortality among mine workers	2 (47, 49)	59/44,980	Czech Republic, Canada	1.223 (0.522–2.867)	0.643	0.076	68.198 (0.000–92.827)	NA
Lymphoma	
	Mortality among mine workers	5 (30, 47, 52)	161/124,327	Czech Republic, Germany, Canada	1.024 (0.963–1.088)	0.449	0.867	0.000 (0.000–79.204)	0.270
Hodgkin lymphoma	
	Mortality among mine workers	2 (30, 47)	15/32,670	Canada, Czech Republic	0.733 (0.241–2.226)	0.584	0.468	0.000 (NA–NA)	NA
Non-Hodgkin lymphoma	
	Mortality among mine workers	3 (30, 47, 52)	146/91,657	Czech Republic, Germany, Canada	1.025 (0.964–1.089)	0.431	0.822	0.000 (0.000–89.598)	0.475
Multiple myeloma	
	Mortality among mine workers	3 (30, 47, 52)	81/91,657	Germany, Canada, Czech Republic	1.007 (0.947–1.070)	0.823	0.992	0.000 (0.000–89.598)	0.966
Brain and central nervous system tumors		
	Incidence among children	4 (23, 25, 79, 85)	8,262/2,024,707	Switzerland, Norway, Denmark, UK	1.015 (0.979–1.052)	0.427	0.108	50.626 (0.000–83.674)	0.164
Brain and central nervous system cancer		
	Mortality among mine workers	2 (32, 52)	120/61,632	Germany, Canada	0.982 (0.947–1.018)	0.319	0.809	0.000 (NA–NA)	NA
Malignant melanoma	
	Mortality among adults in the general population	2 (8, 109)	5,226/5,716,404	Switzerland, USA	1.095 (0.993–1.209)	0.069	0.879	0.000 (NA–NA)	NA
Non-melanoma skin cancer		
	Mortality among adults in the general population	2 (8, 109)	1,431/5,716,404	Switzerland, USA	0.907 (0.612–1.345)	0.628	0.197	39.900 (NA–NA)	NA
Extra-thoracic airways cancer		
	Incidence among mine workers	3 (35, 44, 49)	401/45,738	Czech Republic, Canada	0.902 (0.737–1.106)	0.322	0.252	27.397 (0.000–92.448)	0.830
	Mortality among mine workers	3 (9, 47, 49)	1,747/103,670	Canada, Germany, Czech Republic	1.035 (0.993–1.079)	0.106	0.552	0.000 (0.000–89.598)	0.773
Digestive cancer	
	Incidence among mine workers	5 (35, 49)	468/90,010	Canada	0.977 (0.934–1.022)	0.312	0.740	0.000 (0.000–79.204)	0.737
	Mortality among mine workers	11 (32, 35, 47, 49, 52)	1,933/450,453	Czech Republic, Germany, Canada	1.010 (0.993–1.028)	0.231	0.144	31.889 (0.000–66.518)	0.968
Stomach cancer	
	Incidence among mine workers	2 (35, 49)	196/43,912	Canada	0.958 (0.895–1.025)	0.213	0.694	0.000 (NA–NA)	NA
	Mortality among mine workers	4 (35, 47, 49, 52)	880/120,203	Czech Republic, Germany, Canada	1.000 (0.960–1.043)	0.982	0.219	32.242 (0.000–75.843)	0.395
Liver cancer	
	Mortality among mine workers	2 (47, 52)	207/75,421	Germany, Czech Republic	1.045 (0.998–1.095)	0.063	0.784	0.000 (NA–NA)	NA
Pancreatic cancer	
	Mortality among mine workers	2 (35, 52)	296/75,223	Germany, Canada	1.000 (0.977–1.024)	0.982	0.823	0.000 (NA–NA)	NA
Intestine and rectal cancer		
	Mortality among mine workers	4 (32, 35, 52)	639/136,855	Germany, Canada	1.021 (0.999–1.043)	0.063	0.828	0.000 (0.000–89.598)	0.546
Rectal cancer	
	Mortality among mine workers	2 (32, 52)	256/61,632	Germany, Canada	1.028 (0.993–1.064)	0.113	0.751	0.000 (NA–NA)	NA
Kidney, ureter, other urinary organs cancer	
	Mortality among mine workers	5 (32, 47, 49, 59)	285/109,988	Canada, France, Germany, Czech Republic	1.022 (0.993–1.052)	0.137	0.509	0.000 (0.000–79.204)	0.283
Kidney cancer	
	Mortality among mine workers	3 (32, 49, 52)	230/90,178	Canada, Germany	0.794 (0.413–1.526)	0.488	0.200	37.781 (0.000–80.446)	0.477
Bladder and other urinary organ cancer		
	Mortality among mine workers	2 (32, 52)	187/61,632	Germany, Canada	1.020 (0.985–1.056)	0.264	0.923	0.000 (NA–NA)	NA
Prostate cancer	
	Mortality among mine workers	2 (35, 52)	362/75,223	Germany, Canada	0.998 (0.975–1.021)	0.866	0.521	0.000 (NA–NA)	NA
Chronic Obstructive Pulmonary Disease		
	Mortality among mine workers	3 (26, 34, 40)	1,073/69,120	USA, Germany	1.004 (0.991–1.016)	0.563	0.514	0.000 (0.000–89.598)	0.213
All circulatory system disease		
	Mortality among mine workers	6 (29, 33, 35, 73)	10,117/115,145	Germany, France, Canada	0.994 (0.982–1.006)	0.297	**0.065**	51.861 (0.000–80.813)	0.386
Ischemic heart disease	
	Mortality among mine workers	3 (29, 33, 35, 73)	6,830/82,673	Germany, France, Canada	0.997 (0.985–1.009)	0.627	0.321	11.997 (0.000–90.846)	0.617
Cerebrovascular disease/Stroke		
	Mortality among mine workers	4 (29, 33, 35, 73)	2,151/82,673	Germany, France, Canada	0.984 (0.932–1.038)	0.547	**0.003**	78.495 (42.187–92.000)	0.618

^α^One reference can contribute to the meta-analysis with more than one estimate depending on if estimates were available for different subtypes of the health outcome of interest, or for subgroups of the study population by sex, race, pay-roll status etc.; RR: risk ratio; ^β^100 Bq/m^3^ is the unite of exposure increment for residential exposure (among children and adults in the general population); ^λ^100 WLM is the unite of exposure increment for occupational exposure (among mine workers); CI, Confidence interval; *NA was reported when the number of studies included in the meta-analysis is 2; NA, Not available. Bold values mean statistically significant values.

#### 3.2.1 Estimates for incidence and mortality rates compared with an external group (reference population)

Only studies on occupational exposure to radon among mine workers were considered.

##### 3.2.1.1 Malignant health outcomes

Analyses were performed on incidence and/or mortality data for several cancer locations, including thyroid, other endocrine gland, brain, and central nervous system (CNS), breast, bone and connective tissue, lip, and different types of extra-thoracic airways, skin, digestive, genitourinary organs, and lymphohematological cancers. The metaSIRs indicate a statistically significant lower incidence rate than expected for lymphohematological cancer (especially for non-Hodgkin lymphoma), brain and CNS, kidney (only or combined with ureter and other urinary organs), and connective and soft tissue cancers. Analyses based on mortality data indicate a statistically significant lower mortality rate than expected only for buccal cavity and pharyngeal cancer [metaSMR = 0.80 (95%CI: 0.72–0.88); *p* < 0.001] (Table 1). In contrast to publication bias which was rare, substantial interstudy heterogeneity was detected for most health outcomes (Table 1 and Supplementary Figure S1). Sensitivity analyses using fixed-effect models suggest more statistically significant results than when a random-effect models was used, especially a statistically significant higher rate than expected for rectal cancer incidence [metaSIR = 1.33 (95%CI: 1.14–1.55); *p* < 0.001], stomach cancer mortality [metaSMR = 1.09 (95%CI: 1.03–1.16); *p* = 0.003], liver cancer mortality [metaSMR = 1.27 (95%CI: 1.15–1.41); *p* < 0.001], and liver and gallbladder cancer mortality combined [metaSMR = 1.12 (95%CI: 1.04–1.22); *p* = 0.004] (Supplementary Table S8).

##### 3.2.1.2 Non-malignant health outcomes

Analyses included mortality data for diabetes mellitus, mental and behavioral disorders, nervous and sense organ disorders, and different type of non-malignant obstructive respiratory diseases, circulatory system diseases, and non-malignant digestive disorders (Table 1). Results suggest a statistically significant lower mortality rate than expected for digestive disorders [metaSMR = 0.80 (95%CI: 0.65–0.99), *p* = 0.04]. We found non-significantly increased mortality rates for bronchitis, emphysema, and asthma combined [metaSMR = 1.64 (95%CI: 0.94–2.87); *p* = 0.08], and for mental and behavioral disorders group [metaSMR = 1.54 (95%CI: 0.90–2.61); *p* = 0.11]. Heterogeneity between studies was high across the health outcomes studied. Both funnel plot and the Egger’s test indicate the presence of a publication bias for mortality risk from mental and behavioral disorders. Sensitivity analysis using fixed-effect models suggest substantial changes, including a statistically significant high mortality rate for bronchitis, emphysema, and asthma combined [metaSMR = 1.58 (95%CI: 1.36–1.84), *p* < 0.001], and a statistically significant mortality deficit for all circulatory system disease [metaSMR = 0.88 (95%CI: 0.87–0.89), *p* < 0.001], ischemic heart disease [metaSMR = 0.93 (95%CI: 0.91–0.95), *p* < 0.001], and nervous system and sense organ disorders [metaSMR = 0.88 (95%CI: 0.80–0.98), *p* = 0.02] (Supplementary Table S8 and Supplementary Figure S1).

#### 3.2.2 Estimates for exposure-risk relationships

##### 3.2.2.1 Malignant health outcomes risks

###### 3.2.2.1.1 Among mine workers (occupational exposure)

Analyses were performed on incidence and/or mortality data for several cancer locations, including brain and CNS, extra-thoracic airways, and different types of lymphohematological, digestive, and male genito-urinary cancers. No statistically significant association was found. However, the metaRR per 100 WLM increase in radon exposure pointed toward an increased risk for several cancers: liver cancer mortality [metaRR = 1.05 (95% CI: 1.00–1.10); *p* = 0.06]; overall intestine and rectal cancer mortality [metaRR = 1.02 (95% CI: 1.00–1.04); *p* = 0.06]; leukemia excluding chronic lymphoblastic leukemia (non-CLL) mortality [metaRR = 1.22 (95%CI: 0.52–2.87); *p* = 0.64], etc. The lower bounds of the 95% CIs were close to 1 for mortality from several cancers (liver, intestine and rectal, and extra-thoracic airways, see Table 2). We found no influence of a specific study/estimate on the metaRR per 100 WLM for overall intestine and rectal cancer mortality, except an increase in the *p*-value when omitting one after the other the RRs of rectal cancer and intestine cancer retrieved from the study by Walsh et al. (53) (Supplementary Figure S3). The results remained unchanged when fixed-effect models were used (Supplementary Table S9). There was no evidence for residual heterogeneity among included studies for all health outcomes, except for chronic lymphoblastic leukemia (CLL) and non-CLL mortalities, Cochran’s *Q*-tests *p* = 0.008 and *p* = 0.076, respectively, and I-square value = 85.75% (95%CI: 42.77–96.45%), and 68.20% (95%CI: 0.00–92.83%), respectively. Overall, funnel plots and Egger’s tests suggest no evident publication bias (Table 2 and Supplementary Figure S2).

###### 3.2.2.1.2 Among children (residential exposure)

Analyses among children in the general population included incidence data on leukemia, all lymphohematological cancer, and central nervous system tumors. The metaRRs per 100 Bq/m^3^ increase in residential radon concentration and their 95%CI suggest a marginally increased risk, but not statistically significant [1.01 (95%CI: 1.00–1.03), *p* = 0.126; 1.01 (95%CI: 1.00–1.03), *p* = 0.08; 1.02 (95%CI: 0.98–1.05), *p* = 0.43 for the three types of cancer, respectively]. Lower bounds of the 95%CI were close to, but remained inferior to 1 (which was not visible for some of them after 2-digit rounding). We found no influence of a specific study/estimate on the metaRR per Bq/m^3^ for all lymphohematological cancer incidence, except an increase in the *p*-value when omitting one after the other the study by Raaschou-Nielson et al. (25), and the RRs of leukemia and lymphoma retrieved from the study by Kendall et al. (23) (Supplementary Figure S4). Results did not change when fixed-effect models were used (Supplementary Table S9). There was no indication of inter-study heterogeneity, except for CNS tumors for which a moderate but not statistically significant residual heterogeneity was found among the included studies [Cochran’s *Q*-test *p* = 0.108; I-square value = 50.6% (0.00–83.67%)]. Neither funnel plots nor Egger’s test suggest evidence of publication bias (Table 2 and Supplementary Figure S2).

###### 3.2.2.1.3 Among adults in the general population (residential exposure)

Only mortality from malignant melanoma and non-melanoma skin cancer were covered by a sufficient number of studies to be included in a meta-analysis. No statistically significant association was found. However, the metaRR per 100 Bq/m^3^ increase in residential radon concentration suggest a small decreased risk for non-melanoma skin cancer [0.907 (95%CI: 0.612–1.345); *p* = 0.069], whereas the metaRR for malignant melanoma was positive but not statistically significant [1.095 (95%CI: 0.993–1.209); *p* = 0.628]. The results did not change when fixed-effect model was used (Supplementary Table S9). There was no indication for residual heterogeneity among studies for both malignant melanoma and non-melanoma skin cancer. It was not possible to investigate for publication bias since the number of studies included in the analyses was less than three.

##### 3.2.2.2 Non-malignant health outcomes risks

Analyses focused on mortality from chronic obstructive pulmonary disease, all circulatory system disease, ischemic heart disease, and cerebrovascular disease among mine workers. We found no statistically significant association, even with fixed effect models (Table 2 and Supplementary Table S9). Substantial heterogeneity was found among studies for all circulatory system disease and for cerebrovascular disease [Cochran’s *Q*-test *p* = 0.065 and 0.003, respectively, and I-square value = 51.86% (95%CI: 0.00–80.81%), and 78.50% (95%CI: 42.19–92.00%), respectively]. Both funnel plots and Egger’s tests do not support the existence of publication bias (see Table 2 and Supplementary Figure S2 for more details).

## 4 Discussion

We conducted a comprehensive systematic review and meta-analysis of the potential health effects other than lung cancer associated with radon exposure, covering occupational and residential radon exposure in children and adult populations, for both morbidity and mortality outcomes. This review covered a wide range of malignant and non-malignant diseases. Overall, regardless of the study design, there was an apparently good agreement between findings in children, adults in the general population with residential radon exposure, and mine workers with occupational radon exposure, across health outcomes that were studied in common in these populations, particularly lymphohematological cancers which were the most frequently studied. In most cases, individual study results pointed toward a lack of statistically significant association with radon exposure. Nevertheless, for some cancers, the average weighted estimates of exposure-risk associations were close to the statistical significance threshold, clearly justifying further research on their potential association with radon exposure. This was the case among mine workers for mortality from liver cancer, and also from “intestine and rectal” cancers combined. A positive estimate of exposure-risk association close to the statistical significance threshold was also observed among children for lymphohematological cancer incidence. Finally, this was also observed for malignant melanoma mortality among adults in the general population. It is worth noting that only two studies were included in the analyses for liver cancer mortality among mine workers and malignant melanoma mortality among adults in the general population, meaning these results should be interpreted with caution and their robustness would be improved by pooling with results from further studies in the future. Inter-study heterogeneity was present only in the occupational studies, especially among those included in the metaSIR or metaSMR estimation analyses.

While there is clear evidence that radon can cause lung cancer, even at low exposure levels (156), evidence from our study regarding other potential carcinogenic and non-carcinogenic effects of radon in humans is still inconsistent, whether among mine workers or children or adults in the general population, as it was found in previous reviews on health effects of radon exposure (157, 158). Yet, the hypothesis of radon exposure inducing cancer other than lung cancer is biological plausible. Inhalation of radon predominantly results in the exposure of cells in the lungs to alpha-particles, and only a very low proportion of inhaled radionuclides may enter the blood stream, and deliver dose to other organs like the red bone marrow, brain, heart, digestive system organs, etc. (159). Although direct DNA damage can only occur in cells traversed by alpha-particles after exposure to radon, damages may indirectly extend to the surrounding non-target cells trough molecular signals (160). In addition, radon exposure has been showed to induce systemic inflammation in uranium miners, which is known to increase risks of various cancers and non-cancerous diseases throughout the human body (161, 162).

Several factors may hinder the detection of associations by epidemiological studies. First, retrospective assessment of individual exposure is challenging in epidemiological studies, and many of them suffered from uncertainties in exposure assessment that can influence the exposure-risk relationships estimates toward the null if not correctly addressed (163). In the one hand, studies in the general population often used ecological approaches to assess radon exposure, and it is well known that radon concentrations may vary greatly within small geographical areas and across dwellings. While some case–control studies undertook direct short or long-term radon measurements, ranging from 3 days to 6 months or 1 year, no adjustment was made for seasonal variation in most cases. In addition, case–control studies involving contacts with participants could introduce selection bias due to the risk of low participation rates in controls. On the other hand, in most mine workplaces, the assessment of individual cumulative radon exposure for the earliest time periods was based on retrospective exposure reconstruction by experts and ambient dosimetry measurements. Individual dosimeters were generally introduced later. As a result, in both general population and occupational settings, there is a risk of measurement error, that may affect the health risk estimates toward the null if not correctly managed. However, such limitations did not greatly impair in the past the ability to detect strong associations between radon exposure and lung cancer risk (164). If weaker associations exist with other health effects, the uncertainties in exposure measurement might dilute such associations strongly enough that they cannot be detected anymore. Second, most of studies used mortality data. The use of mortality as a surrogate for incidence is likely to underestimate the true risk for diseases, especially for chronic diseases with relatively good survival rate (165). Finally, some studies did not adjust for important potential confounders, leading to difficulty to effectively disentangle radon health effects from those of exposure to other sources of ionizing radiation, including medical and gamma radiations, and other factors such as tobacco smoking. The statistically significant lower rates than expected (metaSIR or metaSMR) found for incidence of non-Hodgkin lymphoma, brain and CNS cancer, connective and other soft tissue cancer, kidney cancer alone or combined with “ureter and other urinary organs,” and for mortality from buccal and pharyngeal cancer, non-malignant digestive system disorders, are likely to reflect underestimations of the true risk due to the healthy worker effect, which is an issue inherent to occupational cohort studies (166), rather than a protective effect of radon exposure. Thus, these results should be interpreted with caution.

This is the first systematic review and meta-analysis to investigate many diseases at once as a potential health effect of radon exposure, without restriction on study population and radon exposure type. We pooled occupational and residential exposure data separately, since differences in exposure pattern (high exposure over a short duration versus low exposure over a continuous time scale) may result in differences in biological response and risk estimate. We also pooled data for children and adults separately due to the differences in response that may result from differences in baseline risks, latency periods, potential confounding factors or effect modifiers. Furthermore, we pooled incidence and mortality data separately, given the potential for risk underestimation that may be inherent to the use of mortality data. These approaches enable comparisons by exposure and population types, and we recommend this approach as part of future reviews and meta-analyses to better understand the underlying mechanisms that may explain any future epidemiological finding. Additionally, a careful attention was given to ICD codes reported in the included studies for homogeneity purposes, to make sure that studies using the same definition of a disease are pooled together, and that the right disease name is used. Another strength of this work was the use of a double quality assessment tool, the generic and validated NOS (see text footnote 4) and the UNSCEAR tool (17) which is specific to radiation epidemiology. None of the studies included in the meta-analysis was of low quality, moreover, we excluded ecological studies from the meta-analysis so that it may reflect a similar level of quality as the included studies.

Our systematic review and meta-analysis present some limits that need to be highlighted. First, few studies could be included in the meta-analysis for most health outcomes, especially regarding the exposure-risk relationships analyses based on incidence data. This may lead to a lack of power or consistency in results. Second, significant heterogeneity was estimated in studies on occupational exposure to radon, especially among those included in the metaSIR or metaSMR estimation analyses, which limits the interpretation of the average weighted estimates for the health outcomes that were concerned. We did not perform meta-regressions or more in-depth sensitivity analyses to investigate the sources of heterogeneity given the relatively limited number of studies/estimates involved in the analyses. However, for all metaRR close to the statistical significance threshold and whenever possible, we investigated the influence of each single study/estimate on the average weighted estimates, and no noticeable influence was found. Moreover, thanks to pooled studies on residential and occupational exposure to radon separately, and subsequently, studies involving children and adults, incidence data and mortality data may allow us to reduce heterogeneity that may arise from age, exposure level, and data validity differences across studies settings. Several potential reasons were thought to have contributed to the observed residual heterogeneity, including differences in the ways diseases are coded in practice in clinical sittings of each country; but this reason seems unlikely to introduce heterogeneity since this was rarely present among studies included in exposure-risk analyses in this work. One explanation may be differences in the cumulative radon dose received by mine workers in each cohort, depending on the type of mine (uranium, fluor, ore, zinc, etc.), the specific activity (underground or open pit mining, milling), and the environmental conditions. Other plausible explanations would be the possible variability in the baseline risks from one reference population to another (e.g., because cohorts of mine workers were set up in countries with contrasted socioeconomic levels), in mean age at first employment and follow-up duration from one cohort to another. Third, the health risks related to radon exposure that were considered in the meta-analyses were in the great majority based on studies on occupational exposure, and less on residential exposure given the predominance of ecological studies in the general population setting, which were not eligible for the meta-analysis. However, the qualitative summary tables (Supplementary Tables S4–S7) partly offset for this issue, and enables comparison of risks tendencies between children, adults in the general population, and mine workers, for a wide range of health outcomes, especially lymphohematological cancers which were the most studied health outcome in the three population groups.

## 5 Conclusion

While carcinogenic (other than lung cancer) and non-carcinogenic effects associated with radon exposure are biological plausible, the results of this systematic review and meta-analysis did not allow us to confirm radon-related risks other than lung cancer based on currently available epidemiological studies. Existing epidemiological studies are subject to several methodological limitations regarding radon exposure assessment, outcomes and confounding/modifying factors which may dilute the risk estimation, most likely toward the null. However, for several cancers, estimates of exposure-risk associations were close to statistical significance, clearly justifying further research on their potential association with radon exposure. As recommended by the UNSCEAR in the 2019 Report (167), larger and well-designed studies are needed to further investigate whether radon can cause diseases other than lung cancer in humans, and if so, to what extent, as well as potential modifiers such as gender, age or smoking.

## Data Availability

The original contributions presented in the study are included in the article/Supplementary material, further inquiries can be directed to the corresponding author.

## Footnotes

^1^ https://www.radonorm.eu

^2^ https://www.crd.york.ac.uk/prospero/

^3^ https://www.rayyan.ai/

^4^ https://www.ohri.ca/programs/clinical_epidemiology/oxford.asp
