# Supplementary material for: Radon exposure and potential health effects other than lung cancer: a systematic review and meta-analysis
Source: Front Public Health. 2024 Sep 25;12:1439355. doi: 10.3389/fpubh.2024.1439355 (PMC11461271; doi:10.3389/fpubh.2024.1439355)
Supplement: Supplementary file 2 [file Presentation_1.pptx]

## Slide 1
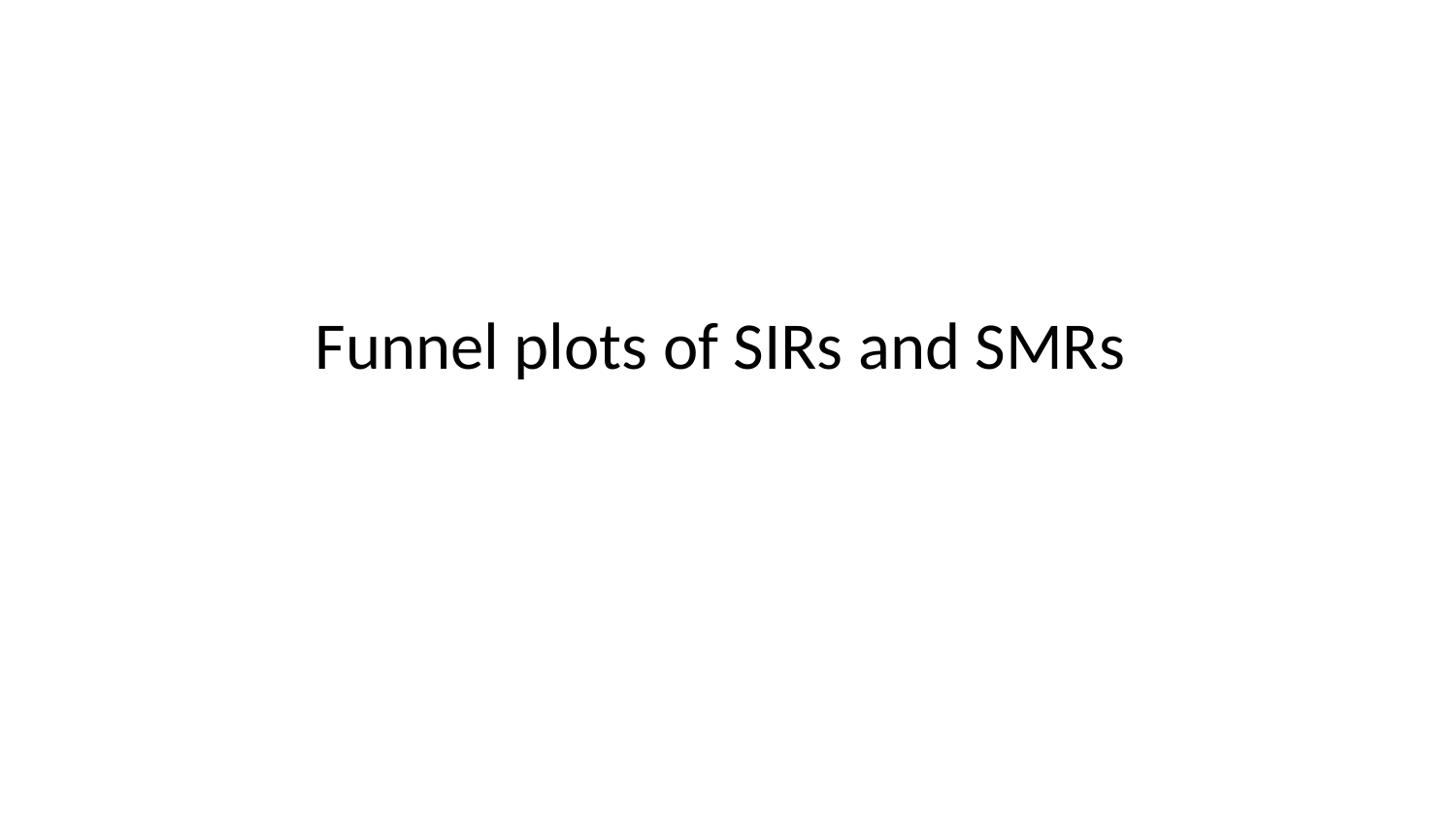

# Funnel plots of SIRs and SMRs

## Slide 2
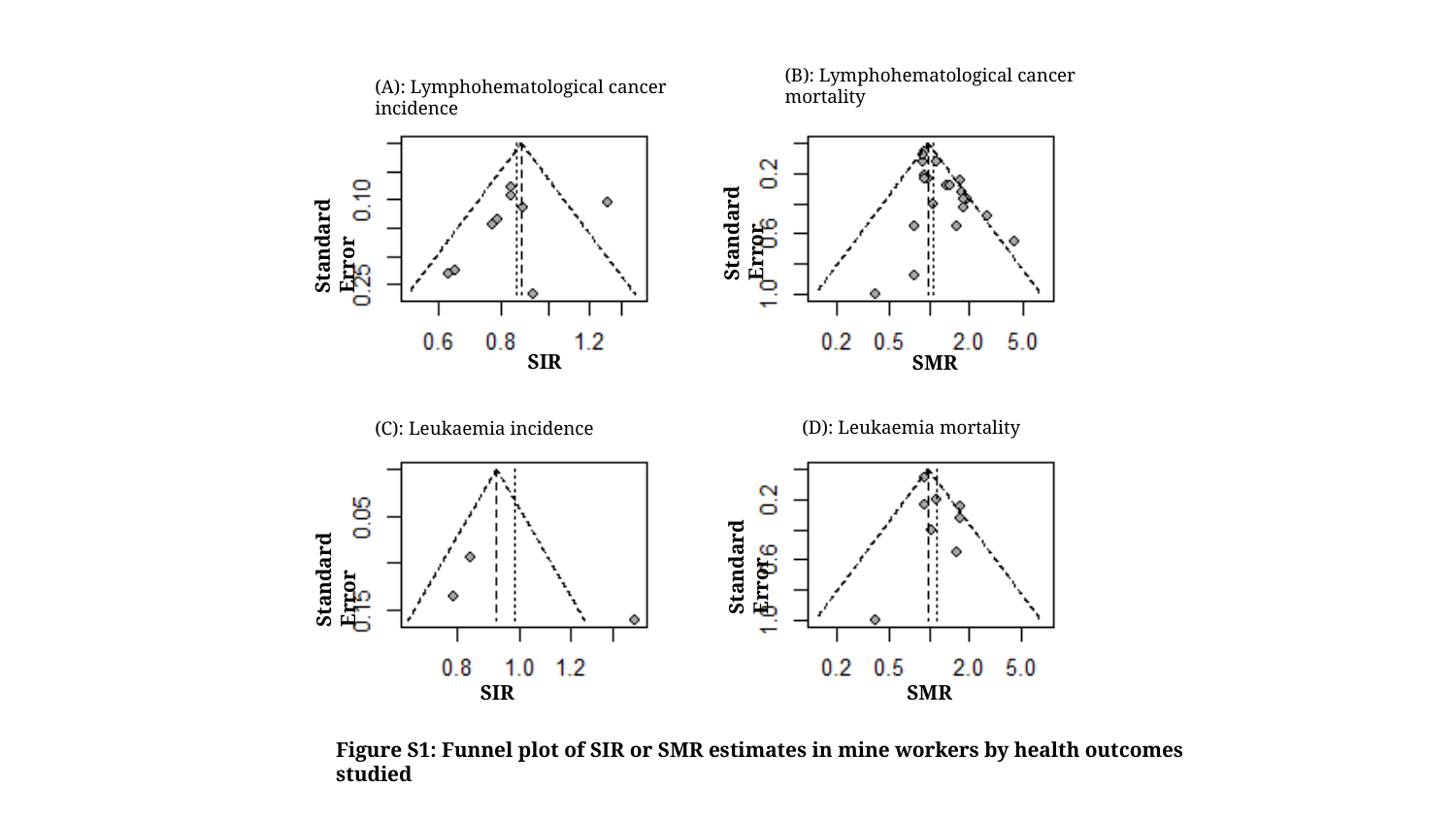

(B): Lymphohematological cancer mortality
(A): Lymphohematological cancer incidence
Standard Error
Standard Error
SIR
SMR
(D): Leukaemia mortality
(C): Leukaemia incidence
Standard Error
Standard Error
SMR
SIR
Figure S1: Funnel plot of SIR or SMR estimates in mine workers by health outcomes studied

## Slide 3
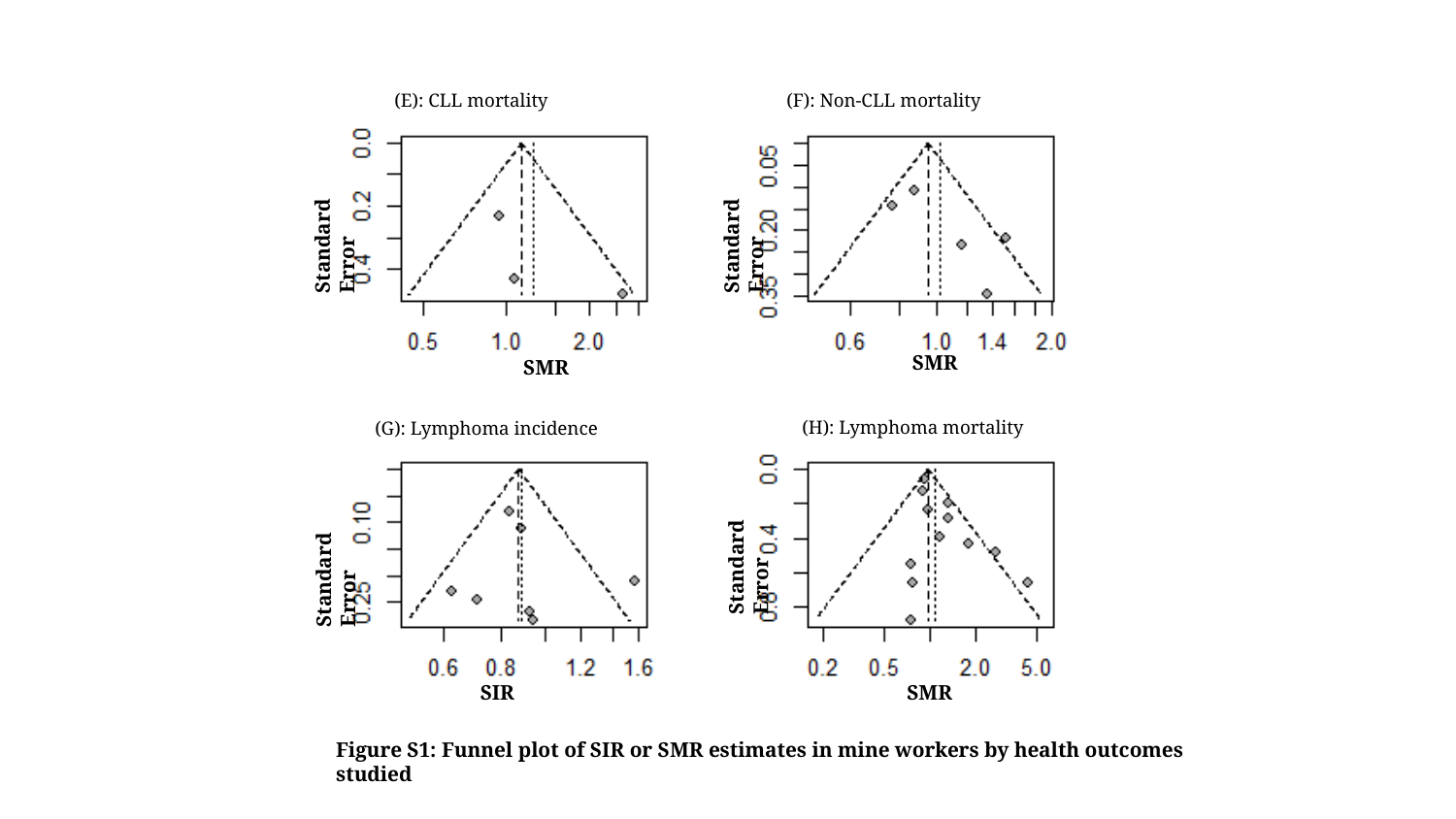

(E): CLL mortality
(F): Non-CLL mortality
Standard Error
Standard Error
SMR
SMR
(H): Lymphoma mortality
(G): Lymphoma incidence
Standard Error
Standard Error
SMR
SIR
Figure S1: Funnel plot of SIR or SMR estimates in mine workers by health outcomes studied

## Slide 4
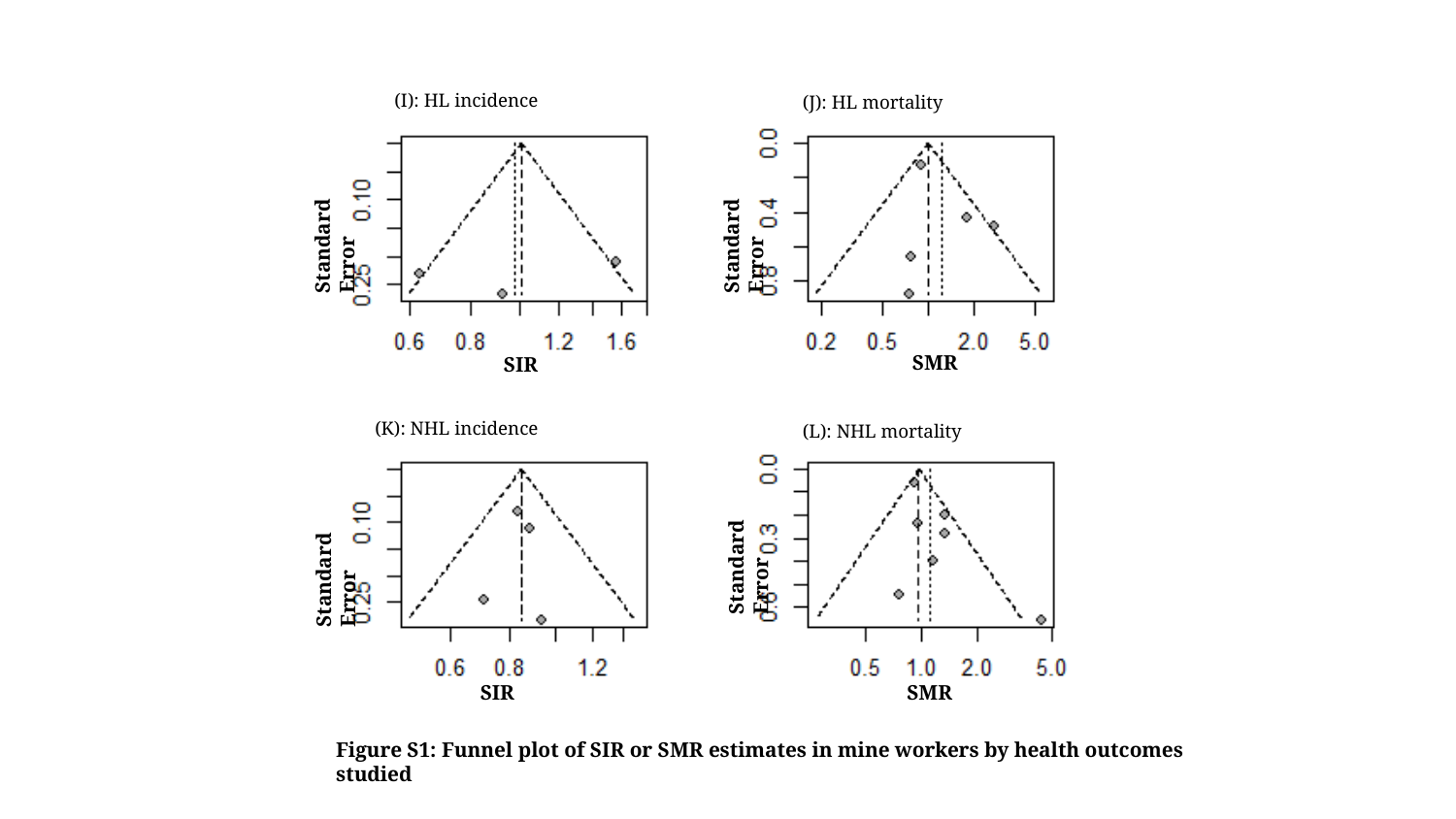

(I): HL incidence
(J): HL mortality
Standard Error
Standard Error
SMR
SIR
(K): NHL incidence
(L): NHL mortality
Standard Error
Standard Error
SMR
SIR
Figure S1: Funnel plot of SIR or SMR estimates in mine workers by health outcomes studied

## Slide 5
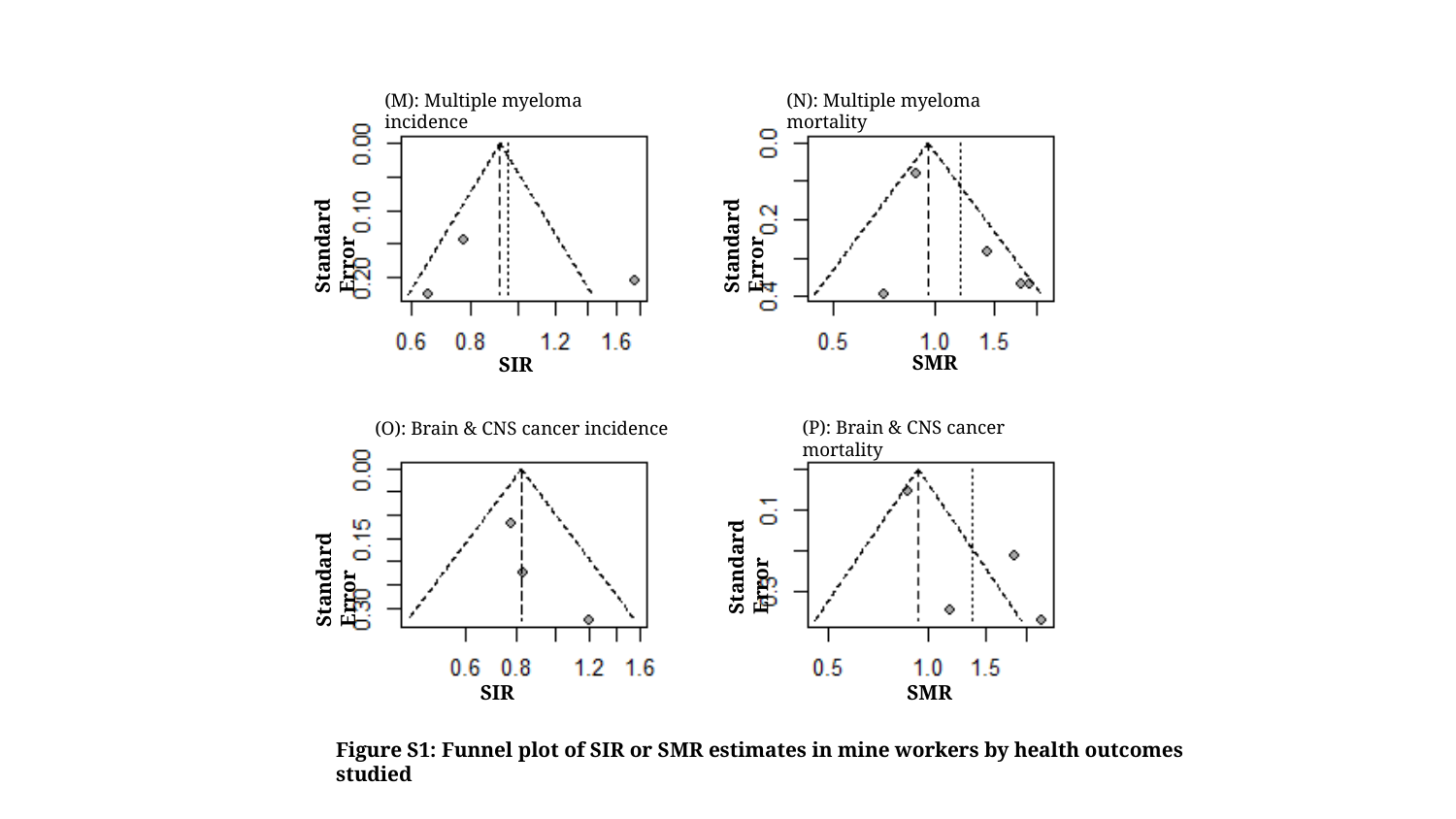

(M): Multiple myeloma incidence
(N): Multiple myeloma mortality
Standard Error
Standard Error
SMR
SIR
(P): Brain & CNS cancer mortality
(O): Brain & CNS cancer incidence
Standard Error
Standard Error
SMR
SIR
Figure S1: Funnel plot of SIR or SMR estimates in mine workers by health outcomes studied

## Slide 6
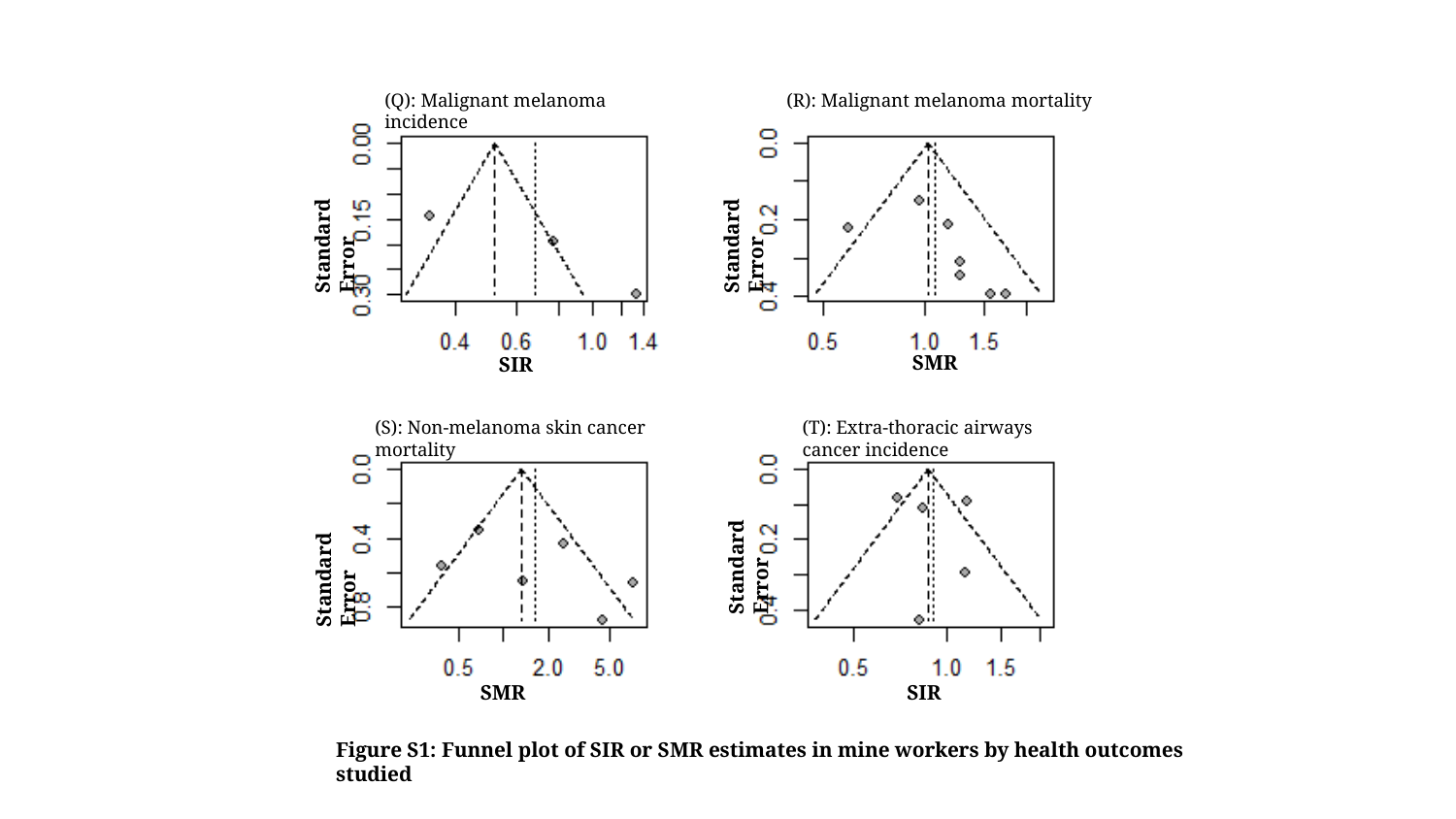

(Q): Malignant melanoma incidence
(R): Malignant melanoma mortality
Standard Error
Standard Error
SMR
SIR
(T): Extra-thoracic airways cancer incidence
(S): Non-melanoma skin cancer mortality
Standard Error
Standard Error
SIR
SMR
Figure S1: Funnel plot of SIR or SMR estimates in mine workers by health outcomes studied

## Slide 7
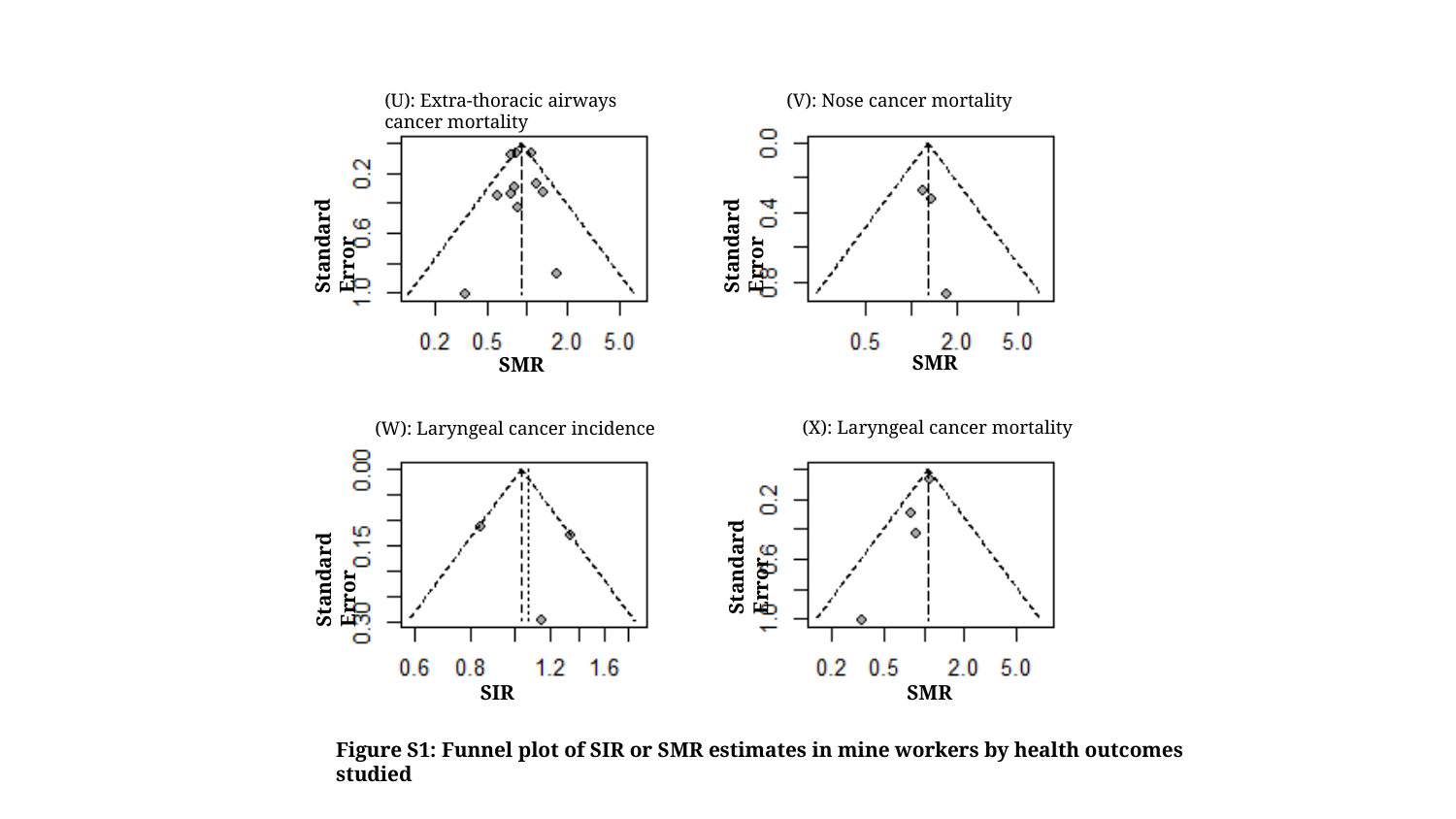

(U): Extra-thoracic airways cancer mortality
(V): Nose cancer mortality
Standard Error
Standard Error
SMR
SMR
(X): Laryngeal cancer mortality
(W): Laryngeal cancer incidence
Standard Error
Standard Error
SMR
SIR
Figure S1: Funnel plot of SIR or SMR estimates in mine workers by health outcomes studied

## Slide 8
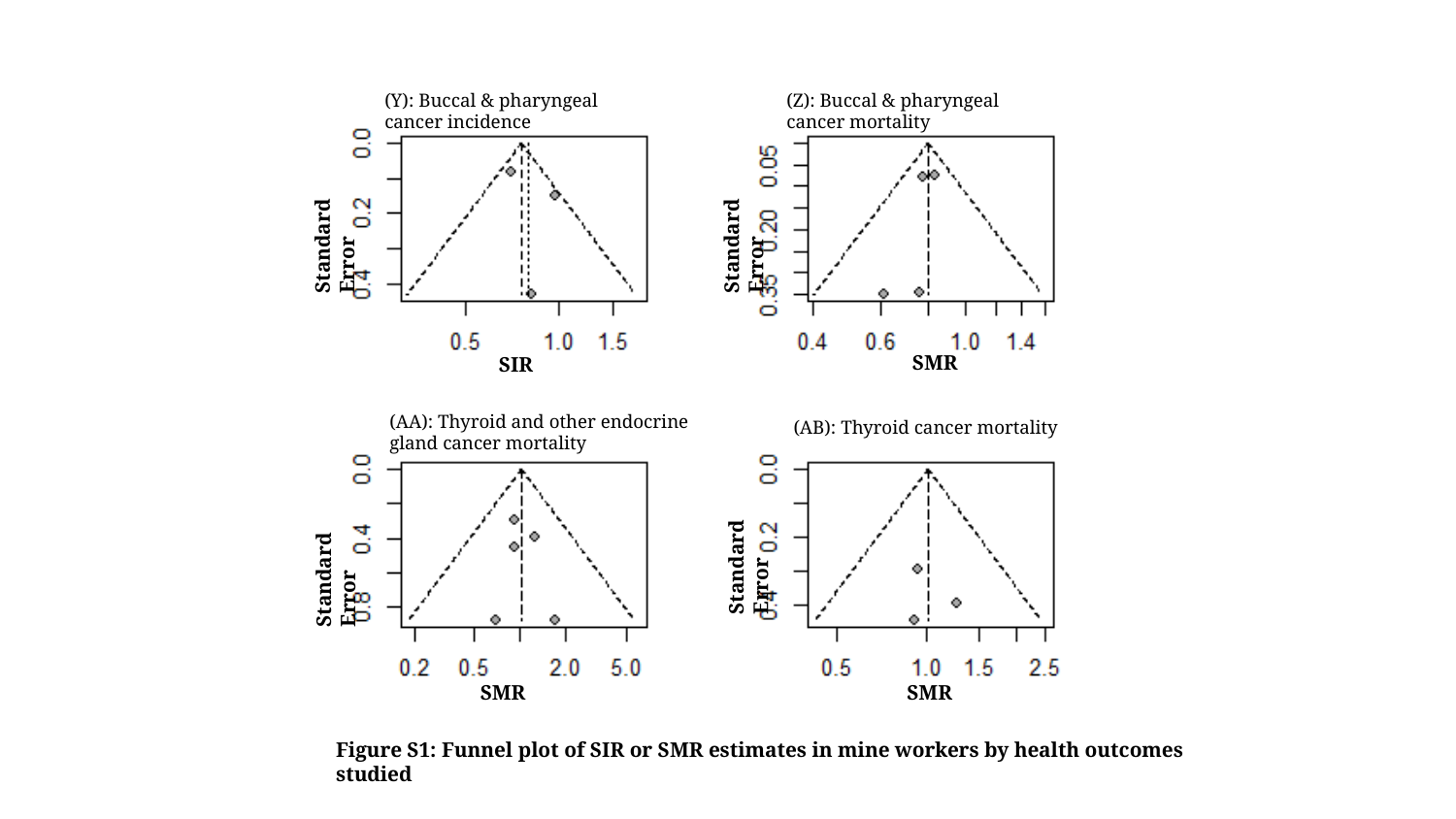

(Y): Buccal & pharyngeal cancer incidence
(Z): Buccal & pharyngeal cancer mortality
Standard Error
Standard Error
SMR
SIR
(AA): Thyroid and other endocrine gland cancer mortality
(AB): Thyroid cancer mortality
Standard Error
Standard Error
SMR
SMR
Figure S1: Funnel plot of SIR or SMR estimates in mine workers by health outcomes studied

## Slide 9
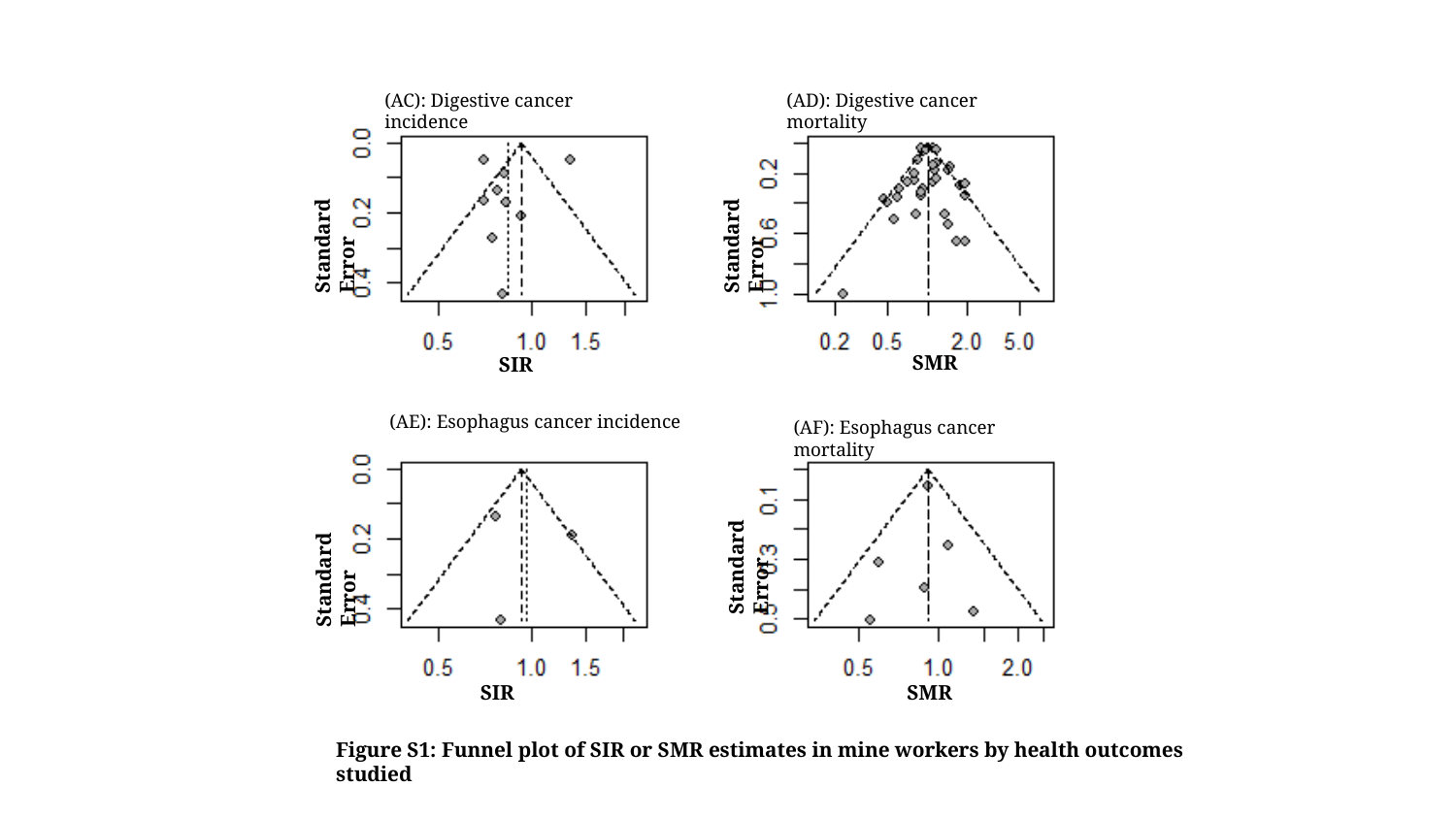

(AC): Digestive cancer incidence
(AD): Digestive cancer mortality
Standard Error
Standard Error
SMR
SIR
(AE): Esophagus cancer incidence
(AF): Esophagus cancer mortality
Standard Error
Standard Error
SMR
SIR
Figure S1: Funnel plot of SIR or SMR estimates in mine workers by health outcomes studied

## Slide 10
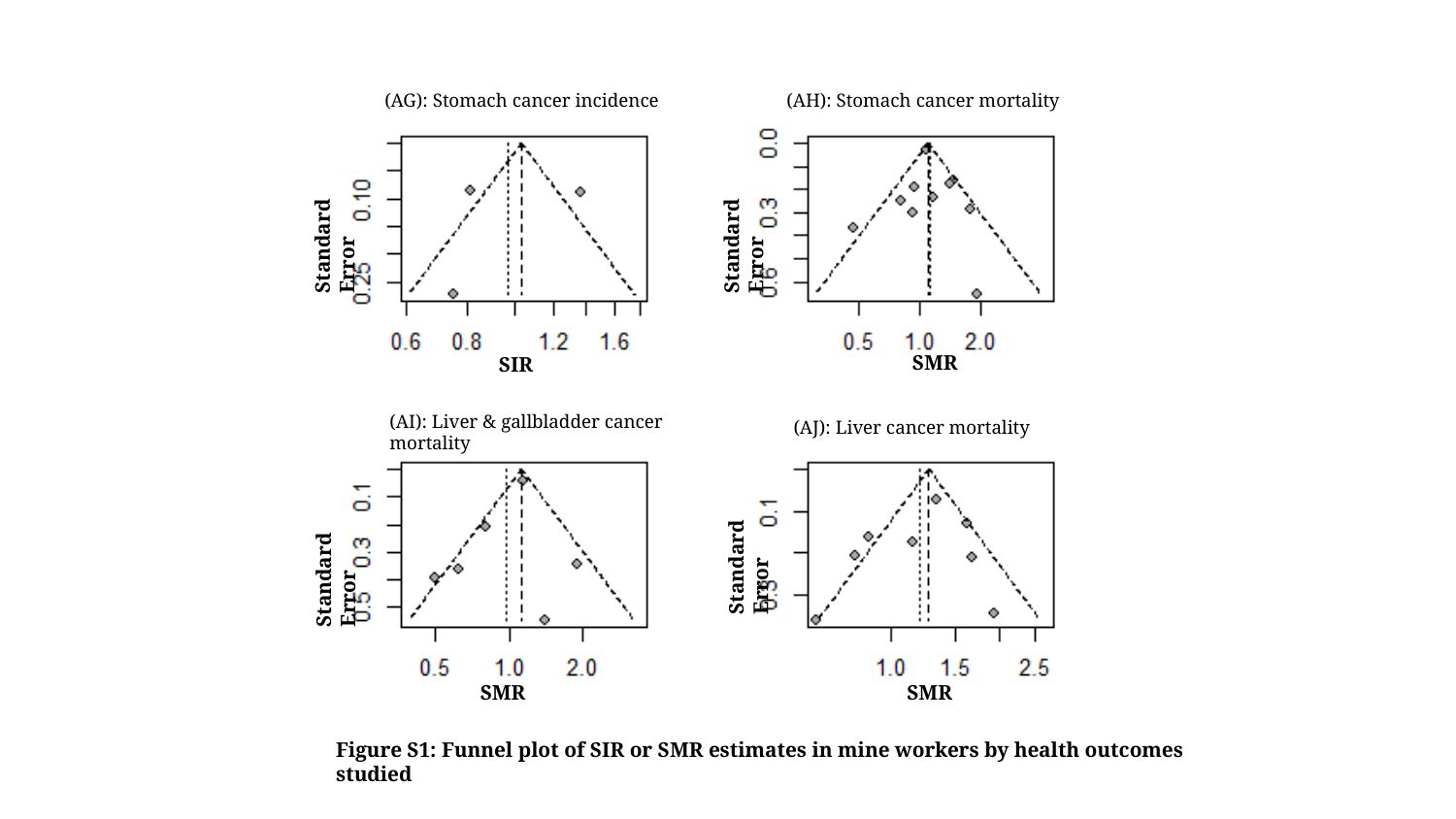

(AG): Stomach cancer incidence
(AH): Stomach cancer mortality
Standard Error
Standard Error
SMR
SIR
(AI): Liver & gallbladder cancer mortality
(AJ): Liver cancer mortality
Standard Error
Standard Error
SMR
SMR
Figure S1: Funnel plot of SIR or SMR estimates in mine workers by health outcomes studied

## Slide 11
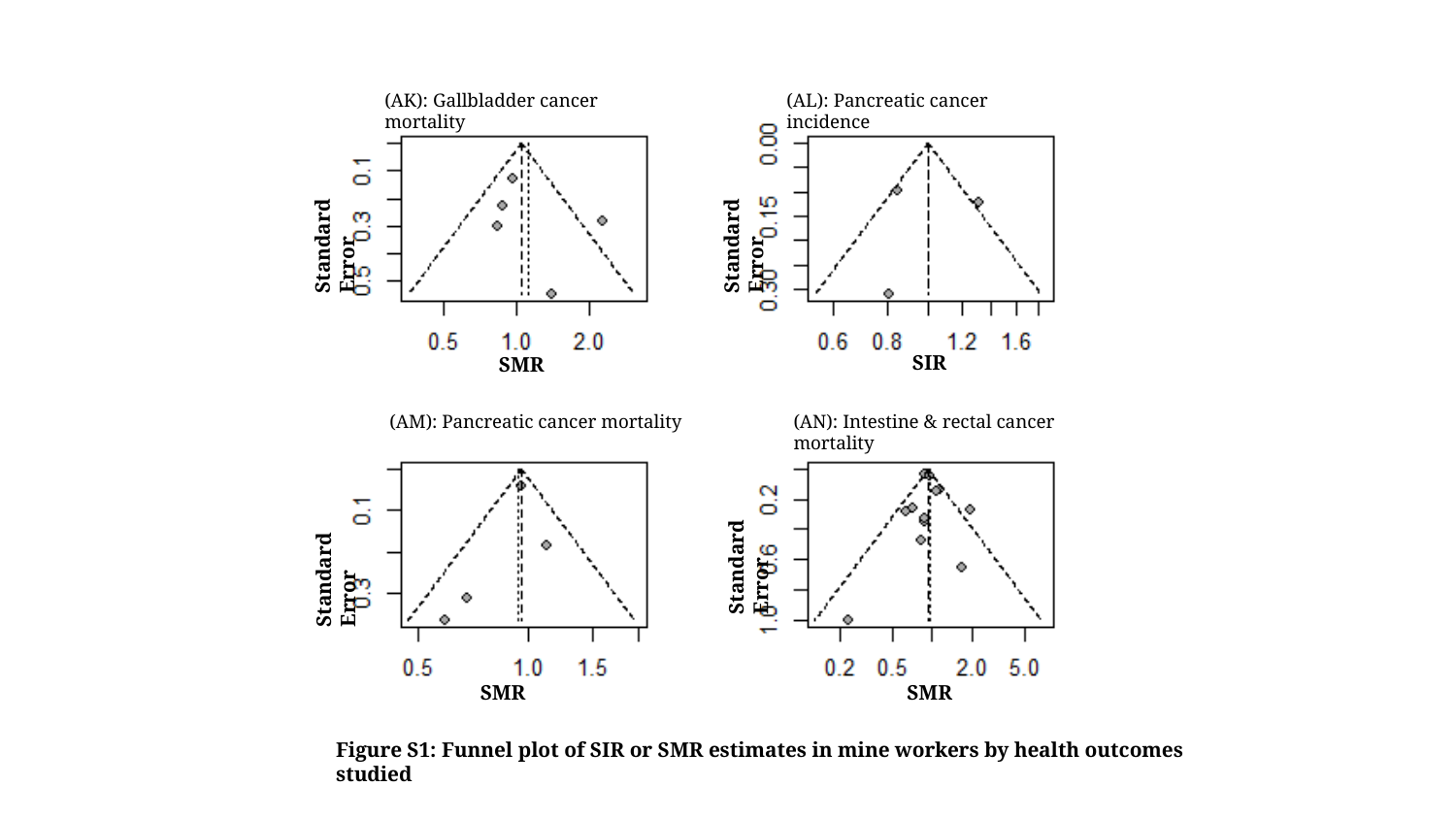

(AK): Gallbladder cancer mortality
(AL): Pancreatic cancer incidence
Standard Error
Standard Error
SIR
SMR
(AM): Pancreatic cancer mortality
(AN): Intestine & rectal cancer mortality
Standard Error
Standard Error
SMR
SMR
Figure S1: Funnel plot of SIR or SMR estimates in mine workers by health outcomes studied

## Slide 12
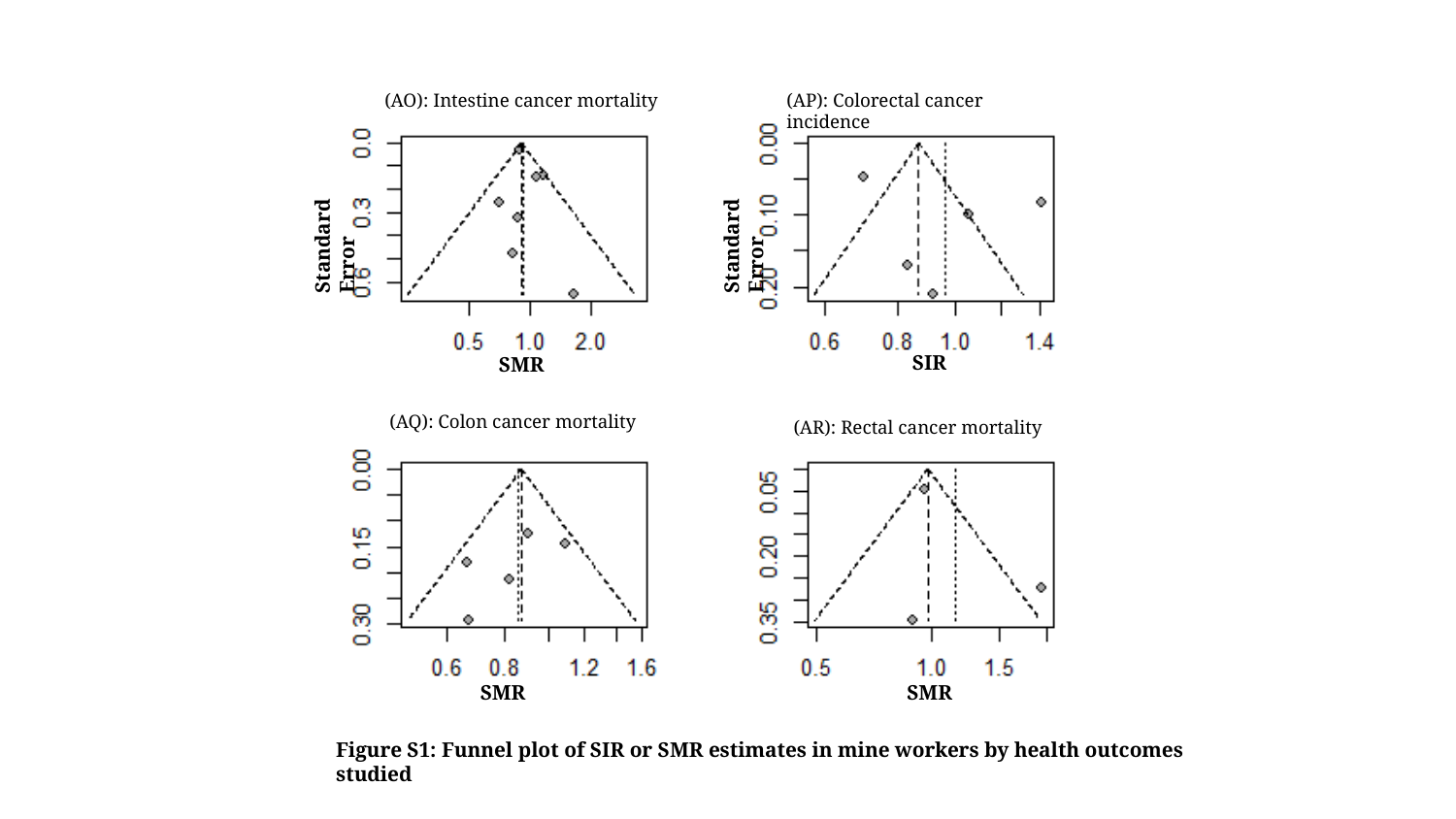

(AO): Intestine cancer mortality
(AP): Colorectal cancer incidence
Standard Error
Standard Error
SIR
SMR
(AQ): Colon cancer mortality
(AR): Rectal cancer mortality
SMR
SMR
Figure S1: Funnel plot of SIR or SMR estimates in mine workers by health outcomes studied

## Slide 13
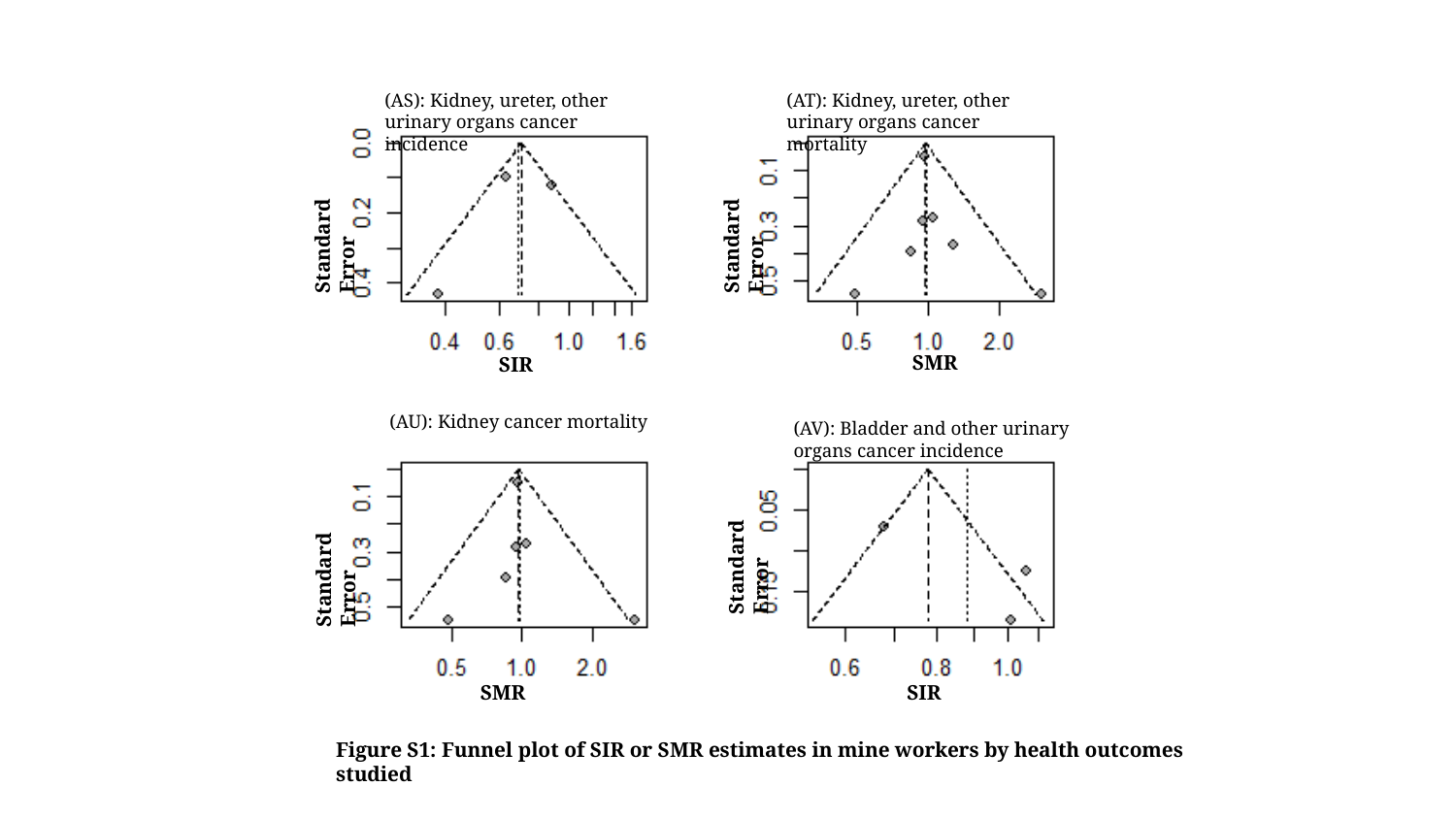

(AS): Kidney, ureter, other urinary organs cancer incidence
(AT): Kidney, ureter, other urinary organs cancer mortality
Standard Error
Standard Error
SMR
SIR
(AU): Kidney cancer mortality
(AV): Bladder and other urinary organs cancer incidence
Standard Error
Standard Error
SIR
SMR
Figure S1: Funnel plot of SIR or SMR estimates in mine workers by health outcomes studied

## Slide 14
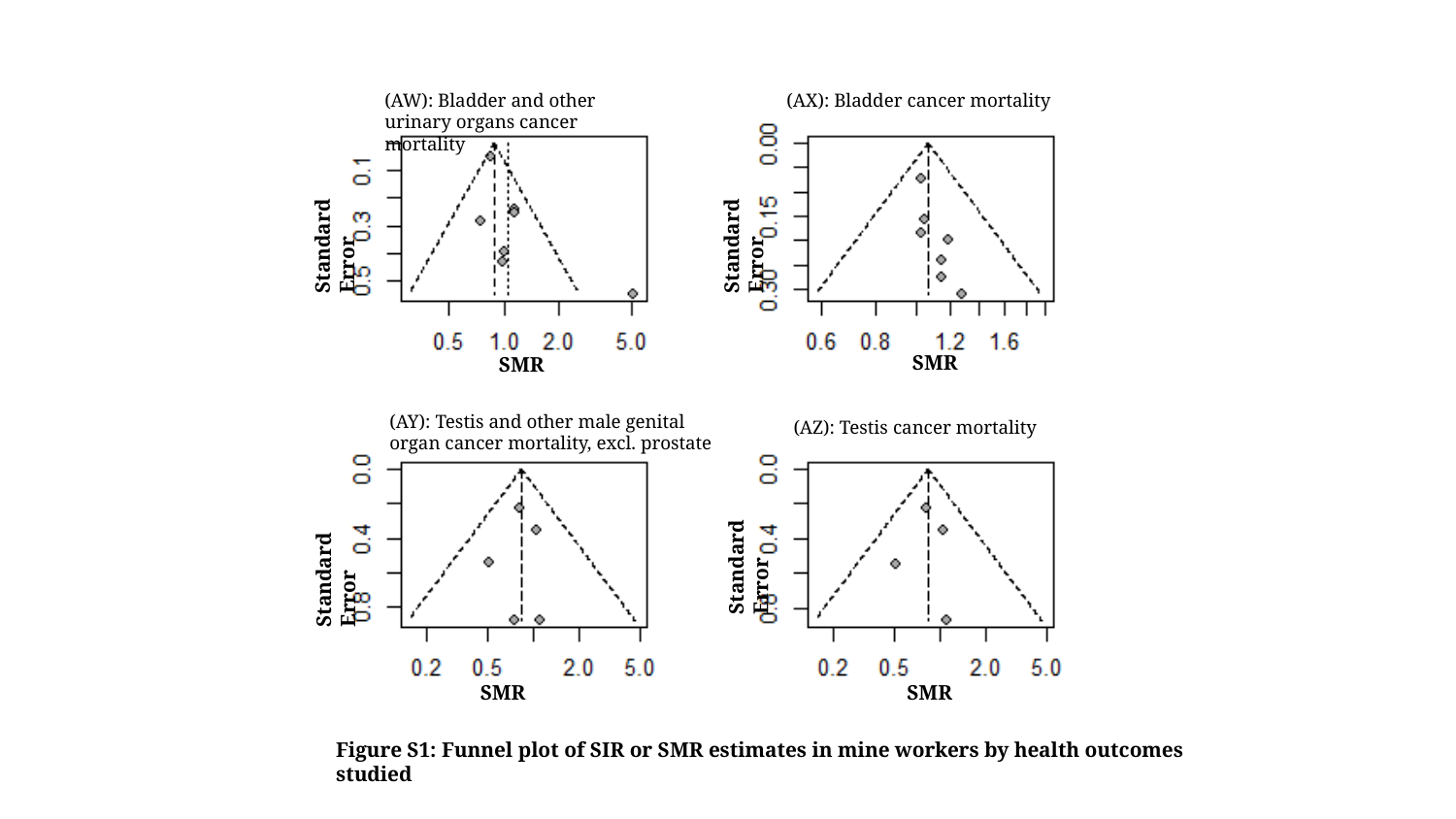

(AW): Bladder and other urinary organs cancer mortality
(AX): Bladder cancer mortality
Standard Error
Standard Error
SMR
SMR
(AY): Testis and other male genital organ cancer mortality, excl. prostate
(AZ): Testis cancer mortality
Standard Error
Standard Error
SMR
SMR
Figure S1: Funnel plot of SIR or SMR estimates in mine workers by health outcomes studied

## Slide 15
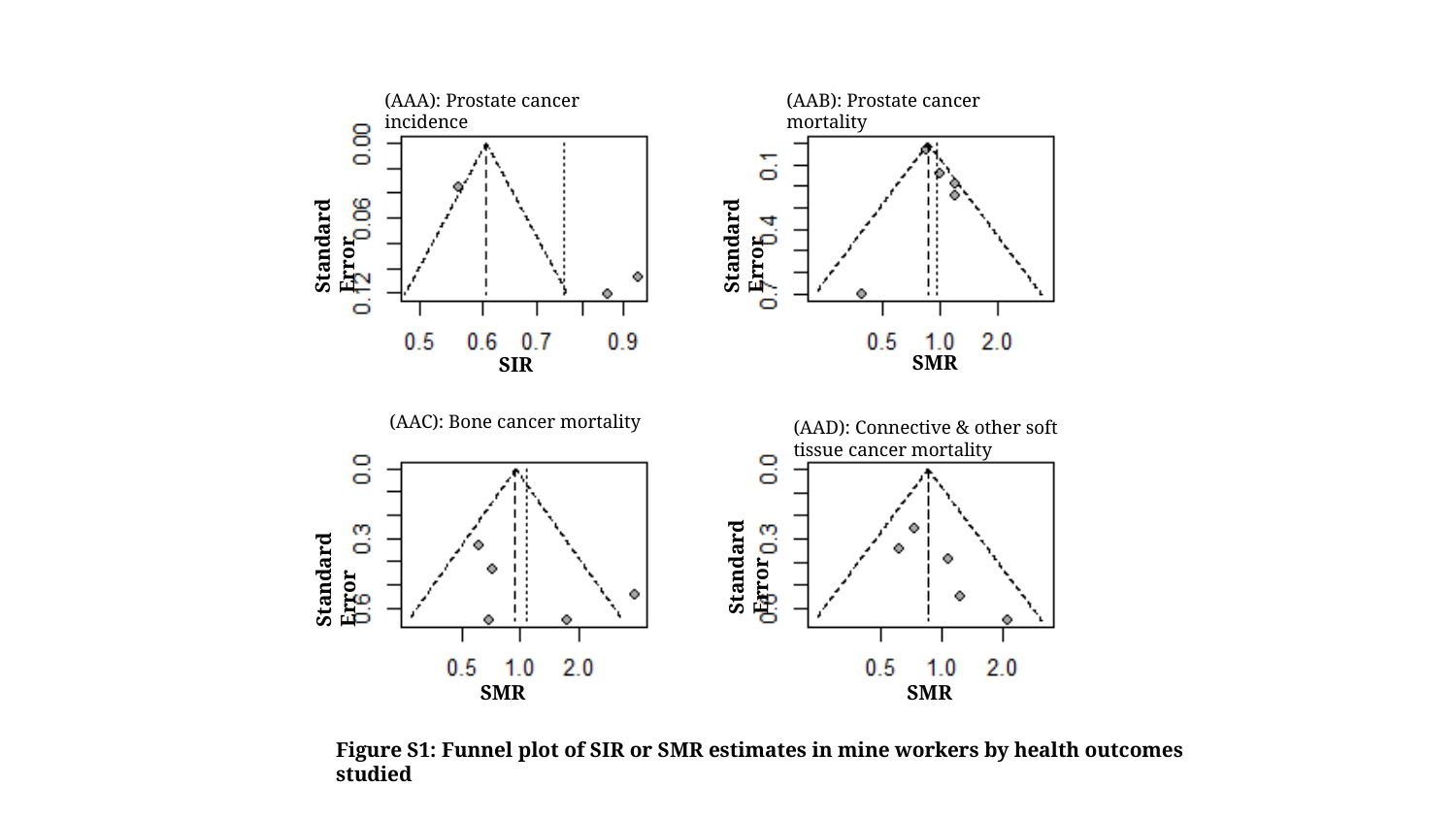

(AAA): Prostate cancer incidence
(AAB): Prostate cancer mortality
Standard Error
Standard Error
SMR
SIR
(AAC): Bone cancer mortality
(AAD): Connective & other soft tissue cancer mortality
Standard Error
Standard Error
SMR
SMR
Figure S1: Funnel plot of SIR or SMR estimates in mine workers by health outcomes studied

## Slide 16
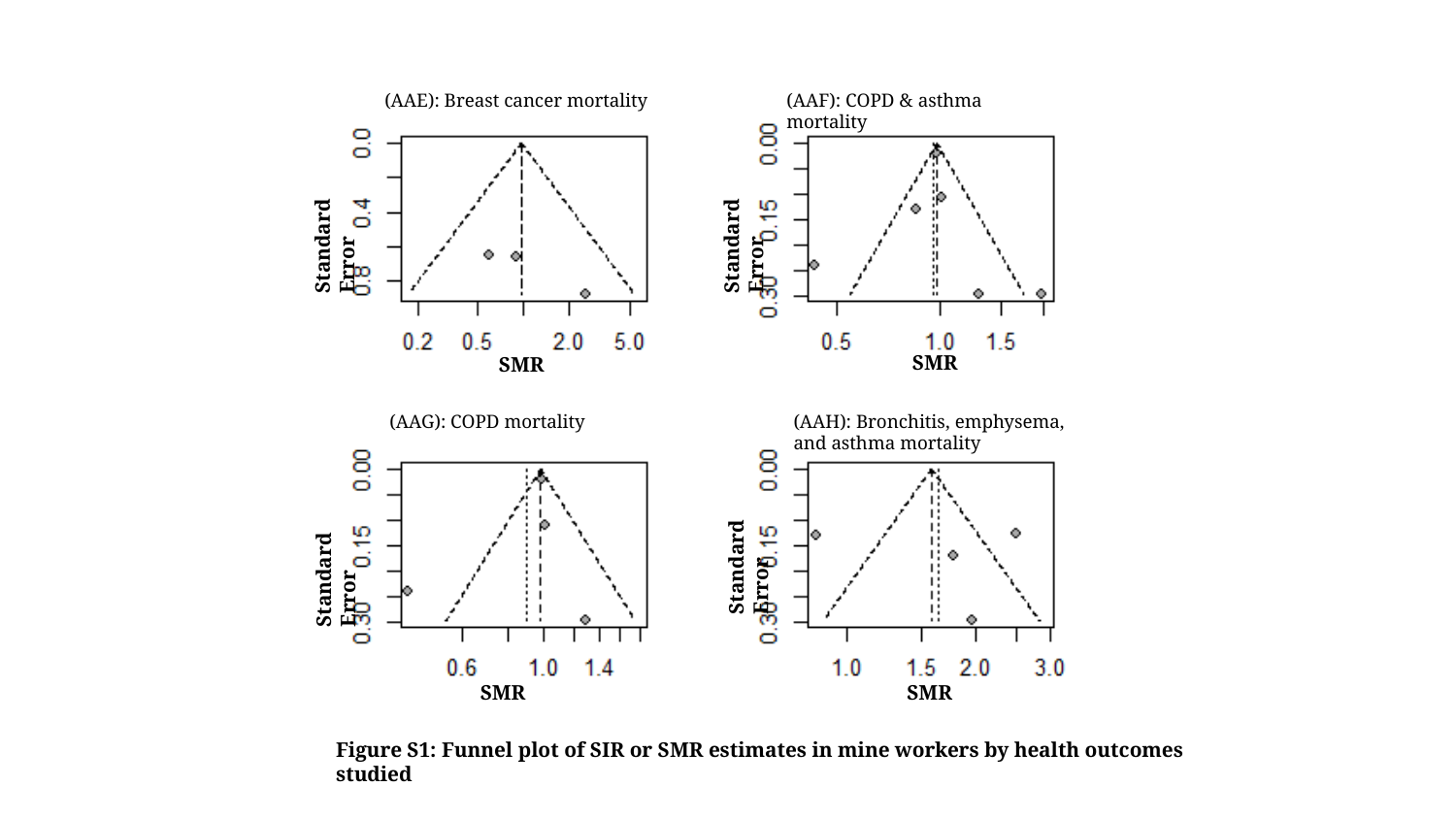

(AAE): Breast cancer mortality
(AAF): COPD & asthma mortality
Standard Error
Standard Error
SMR
SMR
(AAG): COPD mortality
(AAH): Bronchitis, emphysema, and asthma mortality
Standard Error
Standard Error
SMR
SMR
Figure S1: Funnel plot of SIR or SMR estimates in mine workers by health outcomes studied

## Slide 17
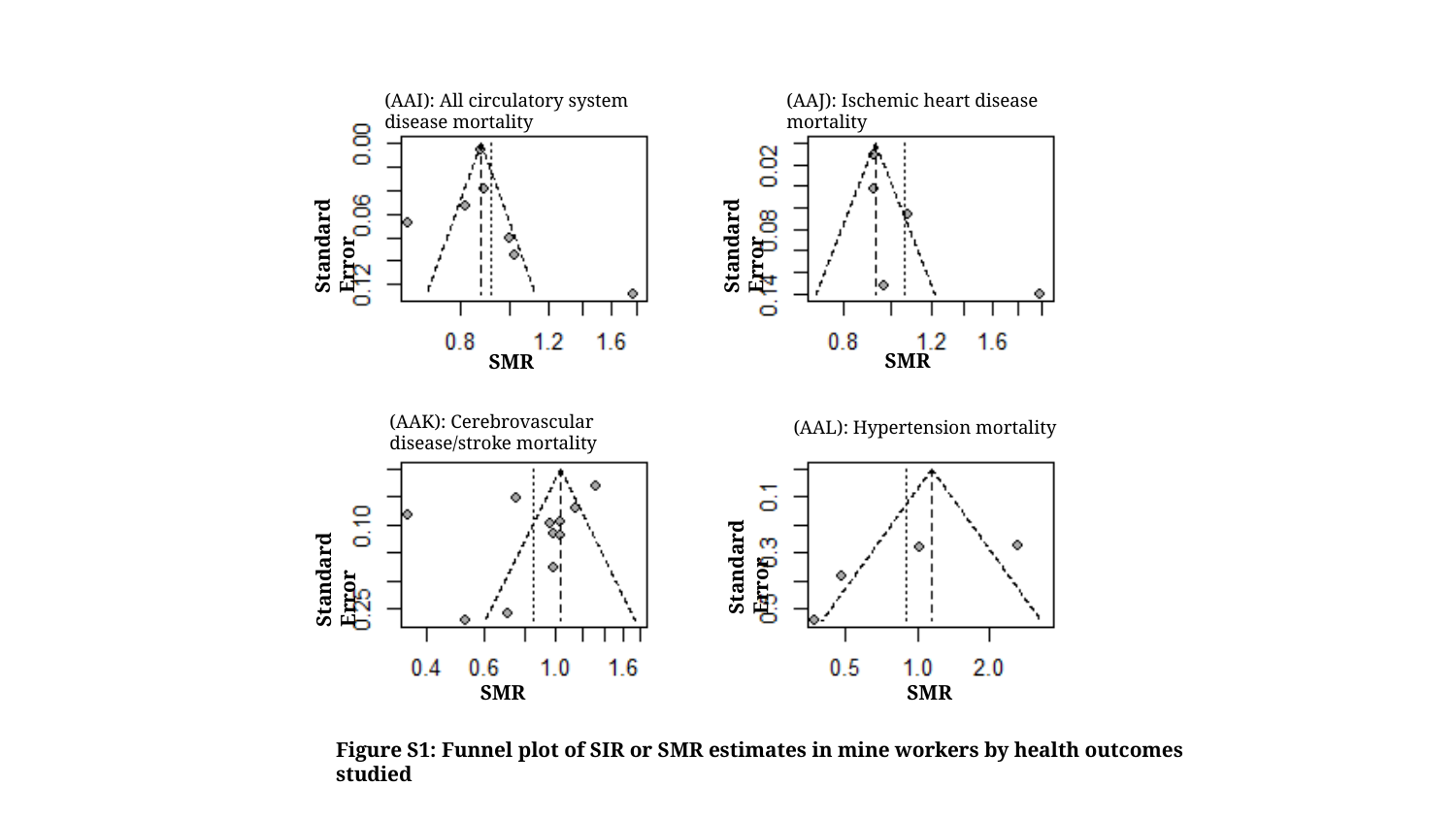

(AAI): All circulatory system disease mortality
(AAJ): Ischemic heart disease mortality
Standard Error
Standard Error
SMR
SMR
(AAK): Cerebrovascular disease/stroke mortality
(AAL): Hypertension mortality
Standard Error
Standard Error
SMR
SMR
Figure S1: Funnel plot of SIR or SMR estimates in mine workers by health outcomes studied

## Slide 18
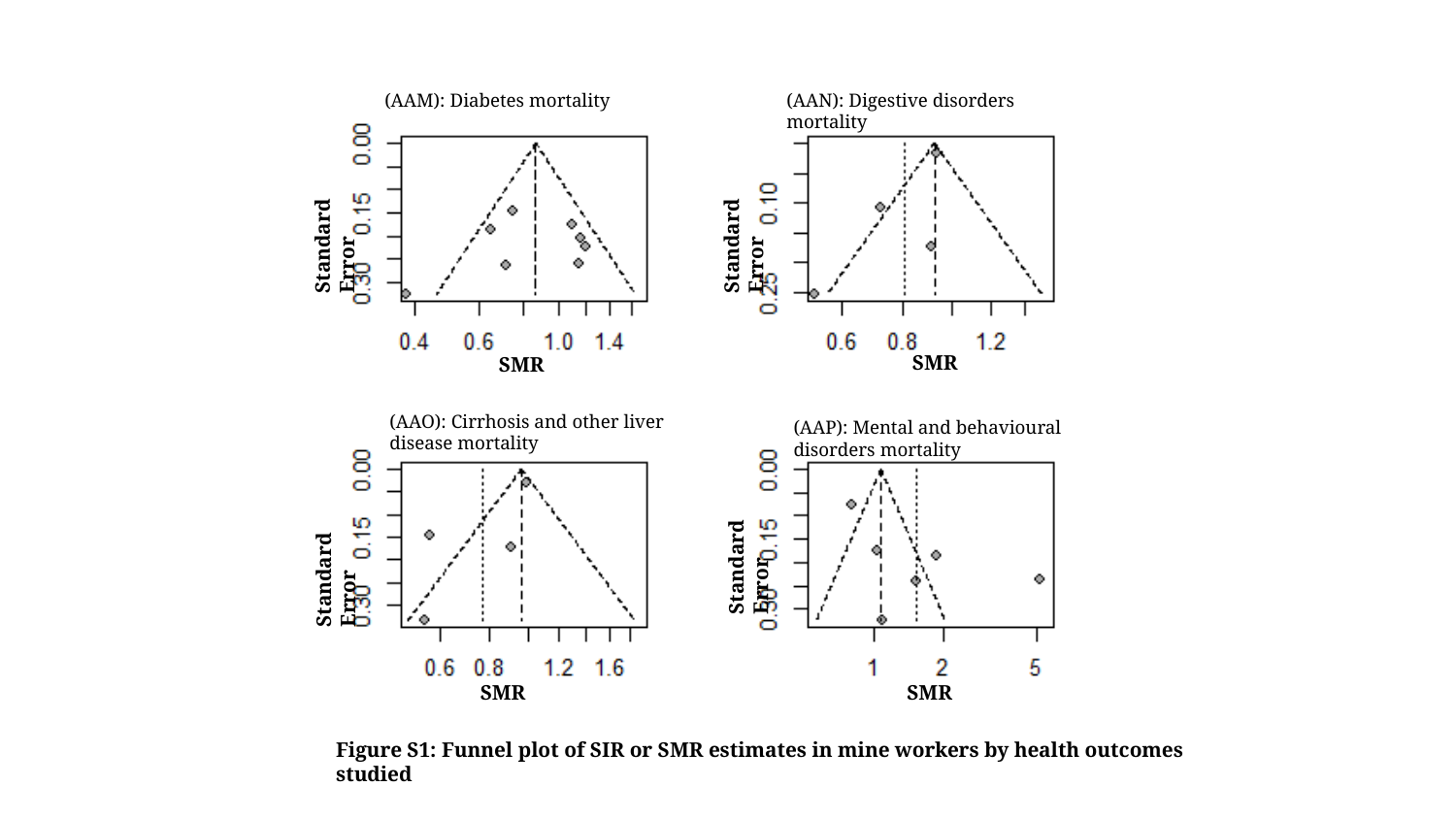

(AAM): Diabetes mortality
(AAN): Digestive disorders mortality
Standard Error
Standard Error
SMR
SMR
(AAO): Cirrhosis and other liver disease mortality
(AAP): Mental and behavioural disorders mortality
Standard Error
Standard Error
SMR
SMR
Figure S1: Funnel plot of SIR or SMR estimates in mine workers by health outcomes studied

## Slide 19
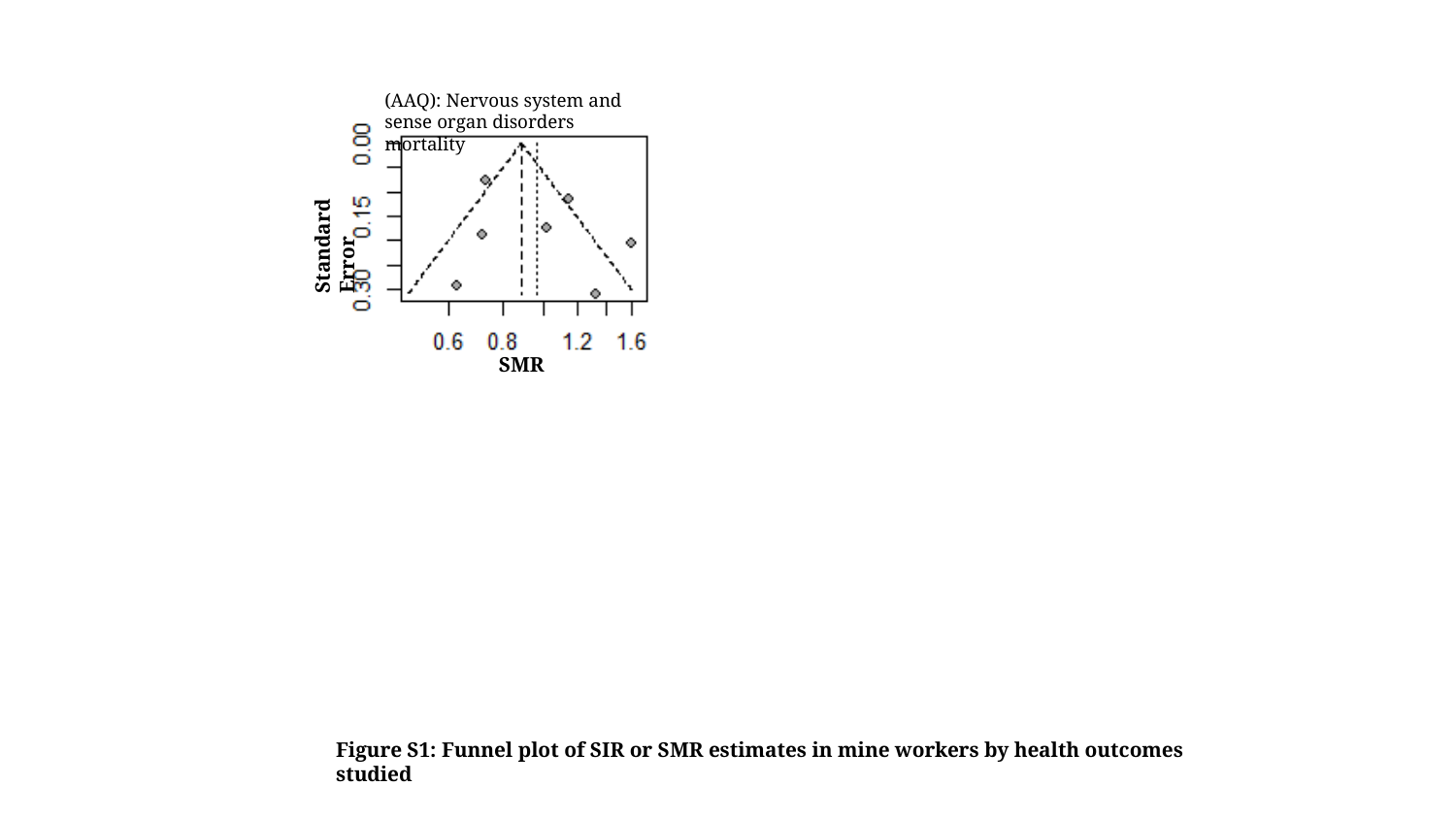

(AAQ): Nervous system and sense organ disorders mortality
Standard Error
SMR
Figure S1: Funnel plot of SIR or SMR estimates in mine workers by health outcomes studied
