# Supplementary material for: Radon exposure and potential health effects other than lung cancer: a systematic review and meta-analysis
Source: Front Public Health. 2024 Sep 25;12:1439355. doi: 10.3389/fpubh.2024.1439355 (PMC11461271; doi:10.3389/fpubh.2024.1439355)
Supplement: Supplementary file 3 [file Presentation_2.pptx]

## Slide 1
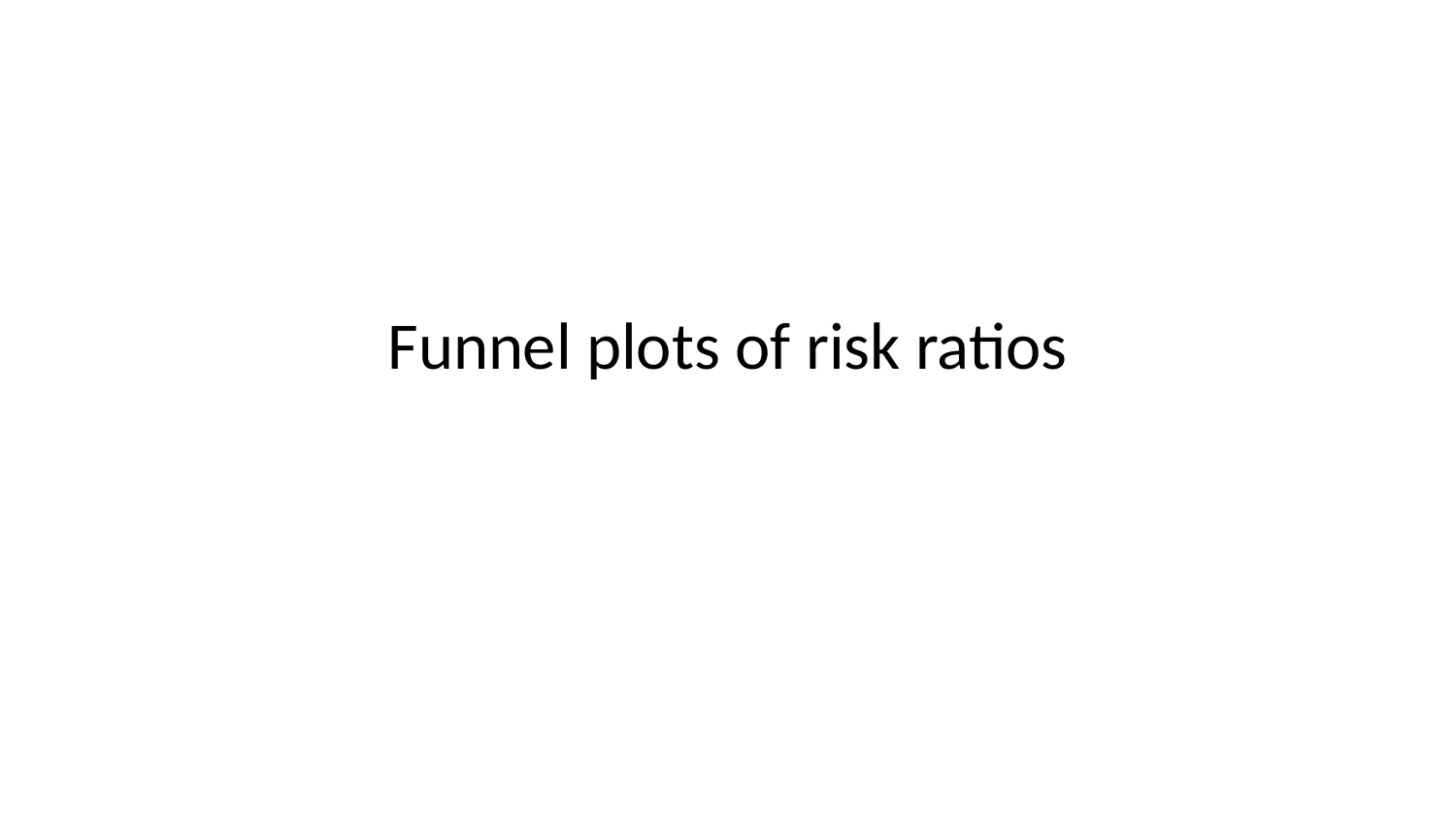

# Funnel plots of risk ratios

## Slide 2
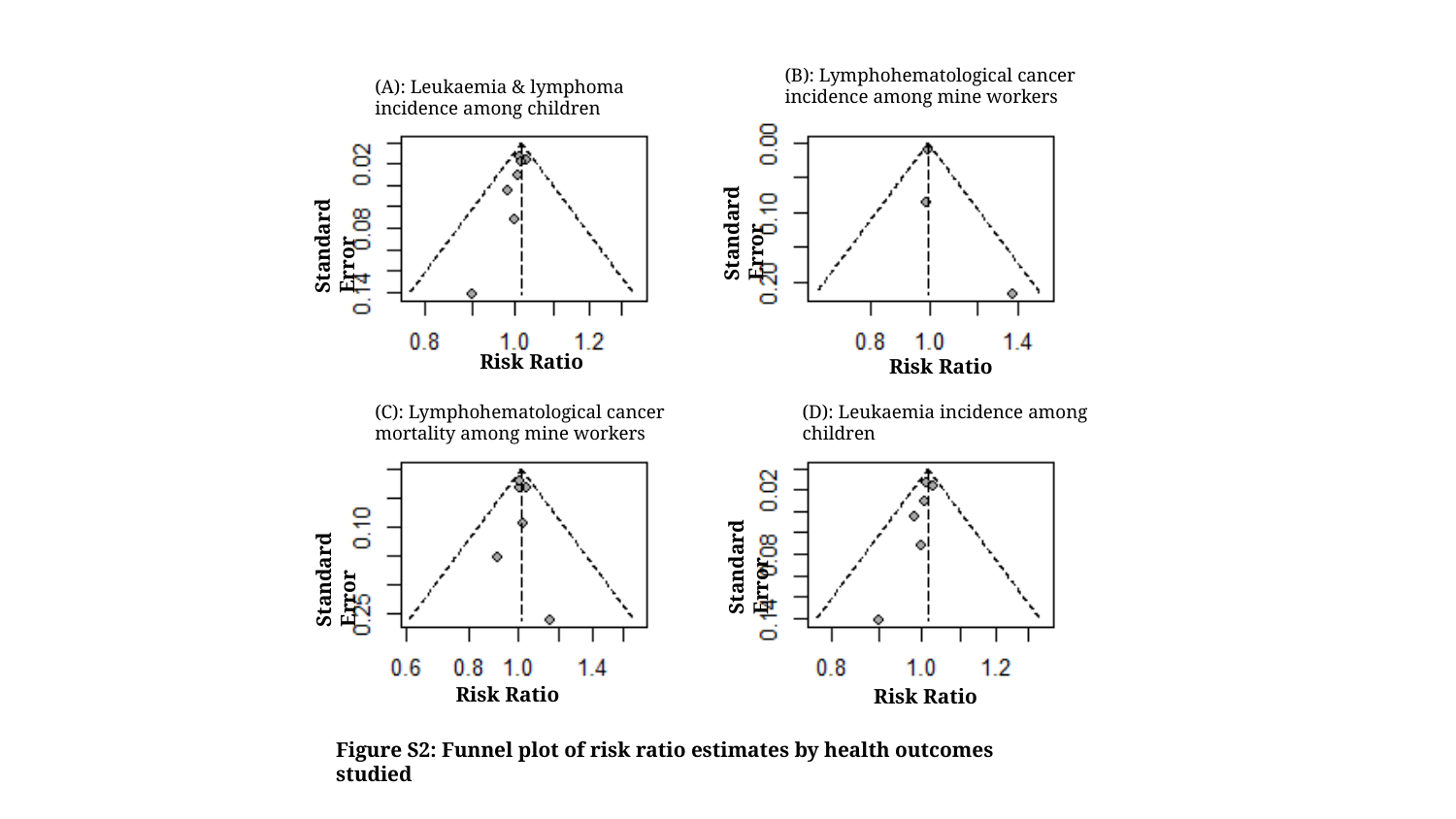

(B): Lymphohematological cancer incidence among mine workers
(A): Leukaemia & lymphoma incidence among children
Standard Error
Standard Error
Risk Ratio
Risk Ratio
(C): Lymphohematological cancer
mortality among mine workers
(D): Leukaemia incidence among children
Standard Error
Standard Error
Risk Ratio
Risk Ratio
Figure S2: Funnel plot of risk ratio estimates by health outcomes studied

## Slide 3
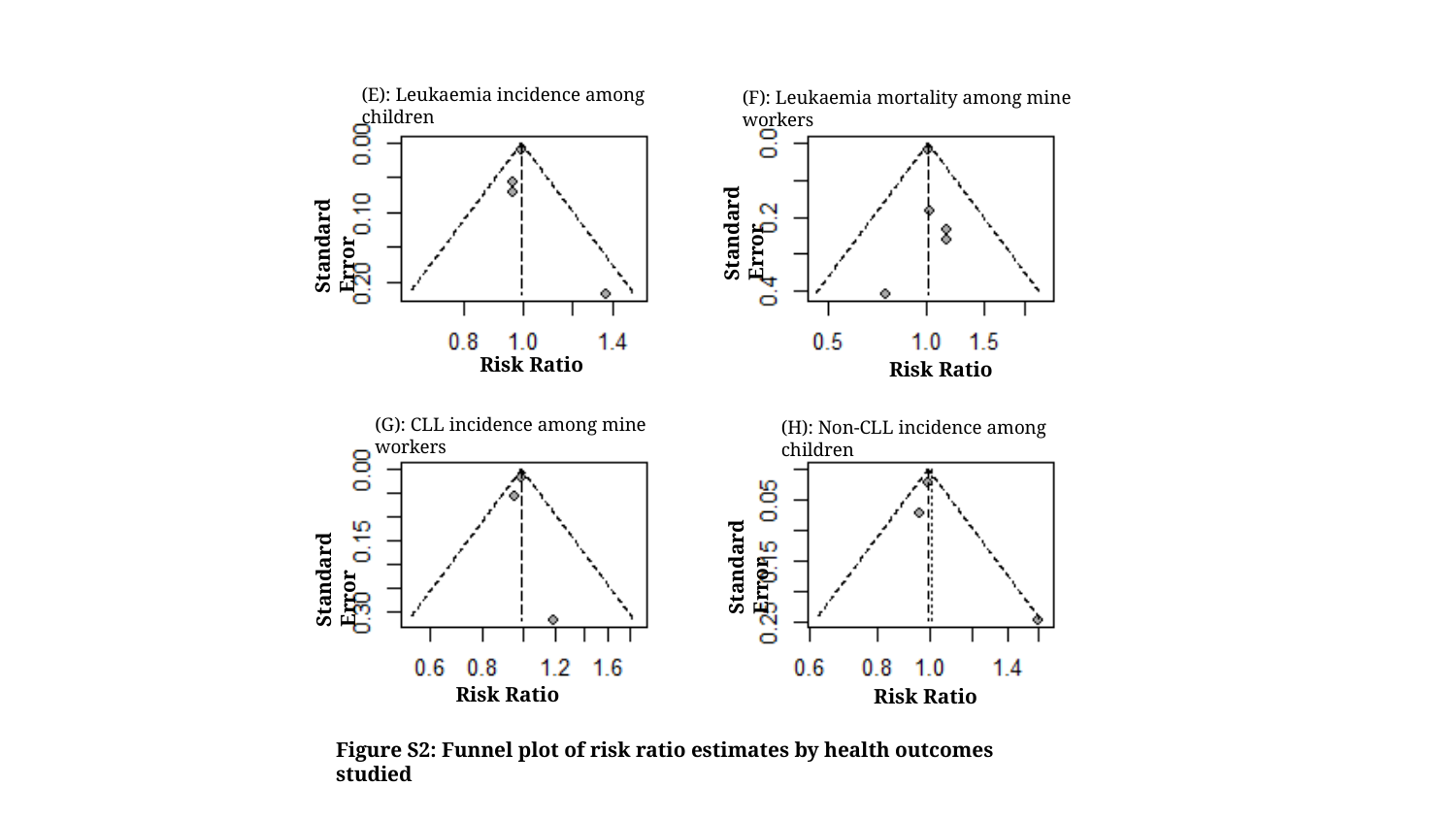

(E): Leukaemia incidence among children
(F): Leukaemia mortality among mine workers
Standard Error
Standard Error
Risk Ratio
Risk Ratio
(G): CLL incidence among mine workers
(H): Non-CLL incidence among children
Standard Error
Standard Error
Risk Ratio
Risk Ratio
Figure S2: Funnel plot of risk ratio estimates by health outcomes studied

## Slide 4
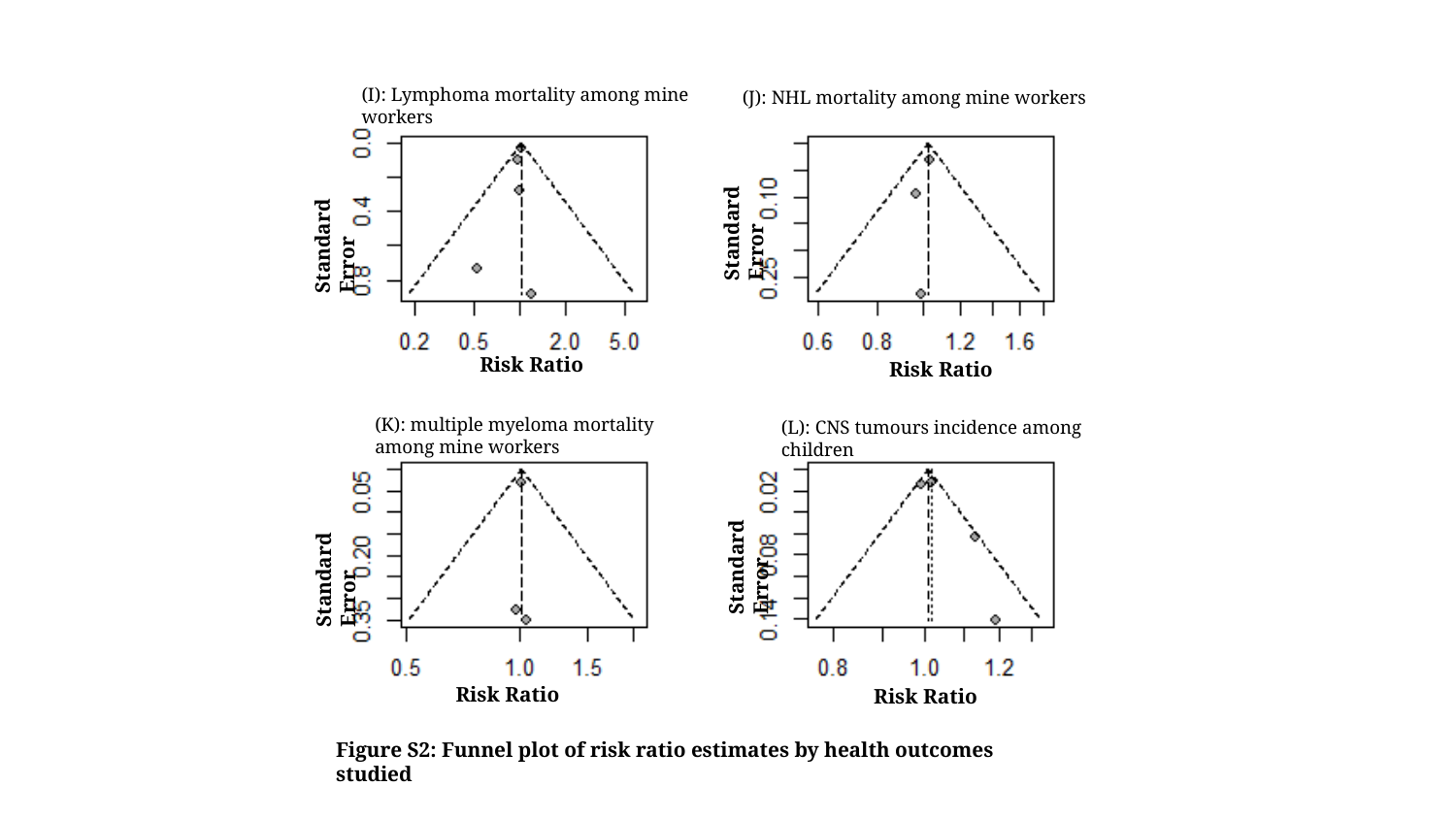

(I): Lymphoma mortality among mine workers
(J): NHL mortality among mine workers
Standard Error
Standard Error
Risk Ratio
Risk Ratio
(K): multiple myeloma mortality among mine workers
(L): CNS tumours incidence among children
Standard Error
Standard Error
Risk Ratio
Risk Ratio
Figure S2: Funnel plot of risk ratio estimates by health outcomes studied

## Slide 5
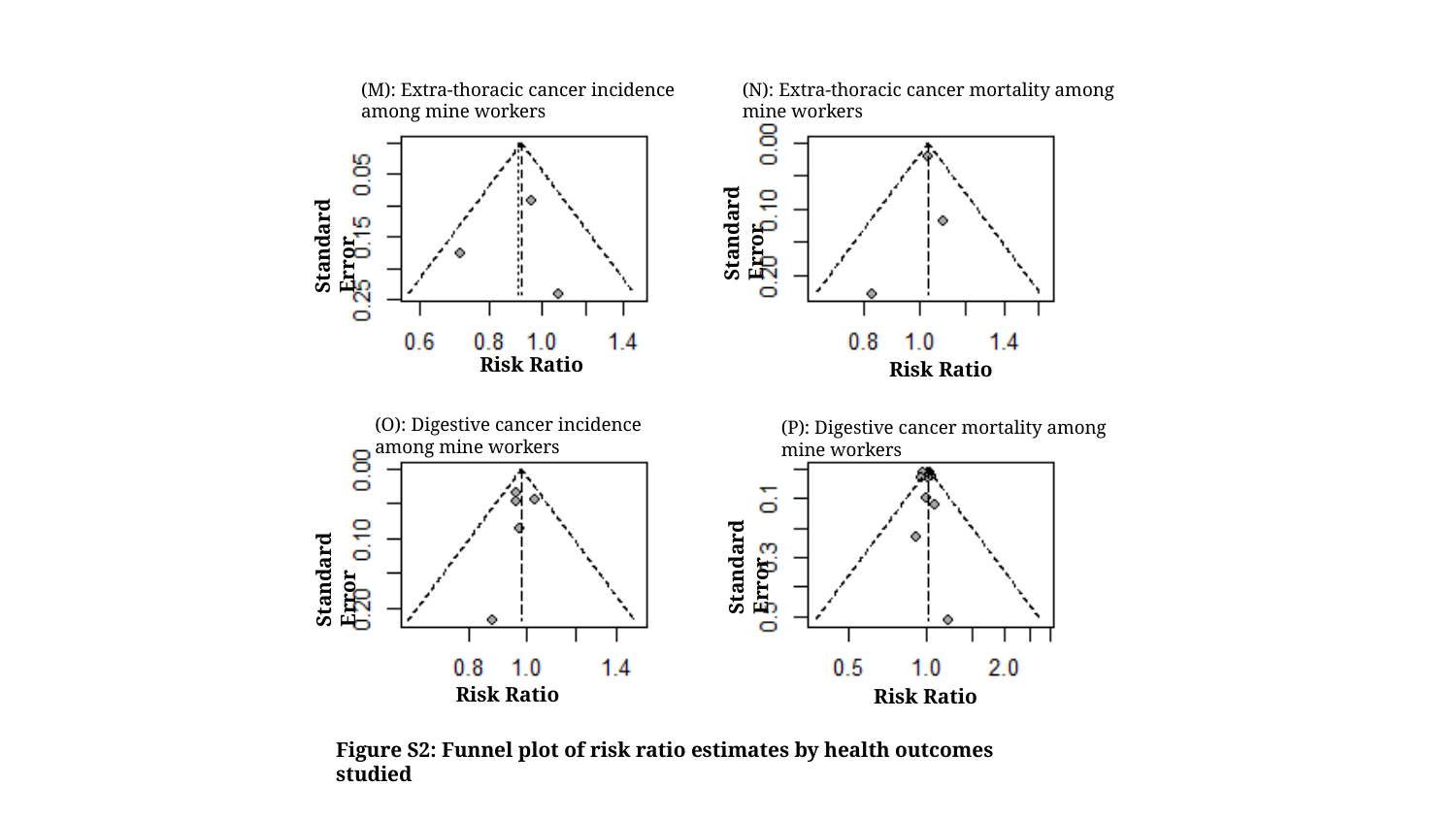

(M): Extra-thoracic cancer incidence among mine workers
(N): Extra-thoracic cancer mortality among mine workers
Standard Error
Standard Error
Risk Ratio
Risk Ratio
(O): Digestive cancer incidence among mine workers
(P): Digestive cancer mortality among mine workers
Standard Error
Standard Error
Risk Ratio
Risk Ratio
Figure S2: Funnel plot of risk ratio estimates by health outcomes studied

## Slide 6
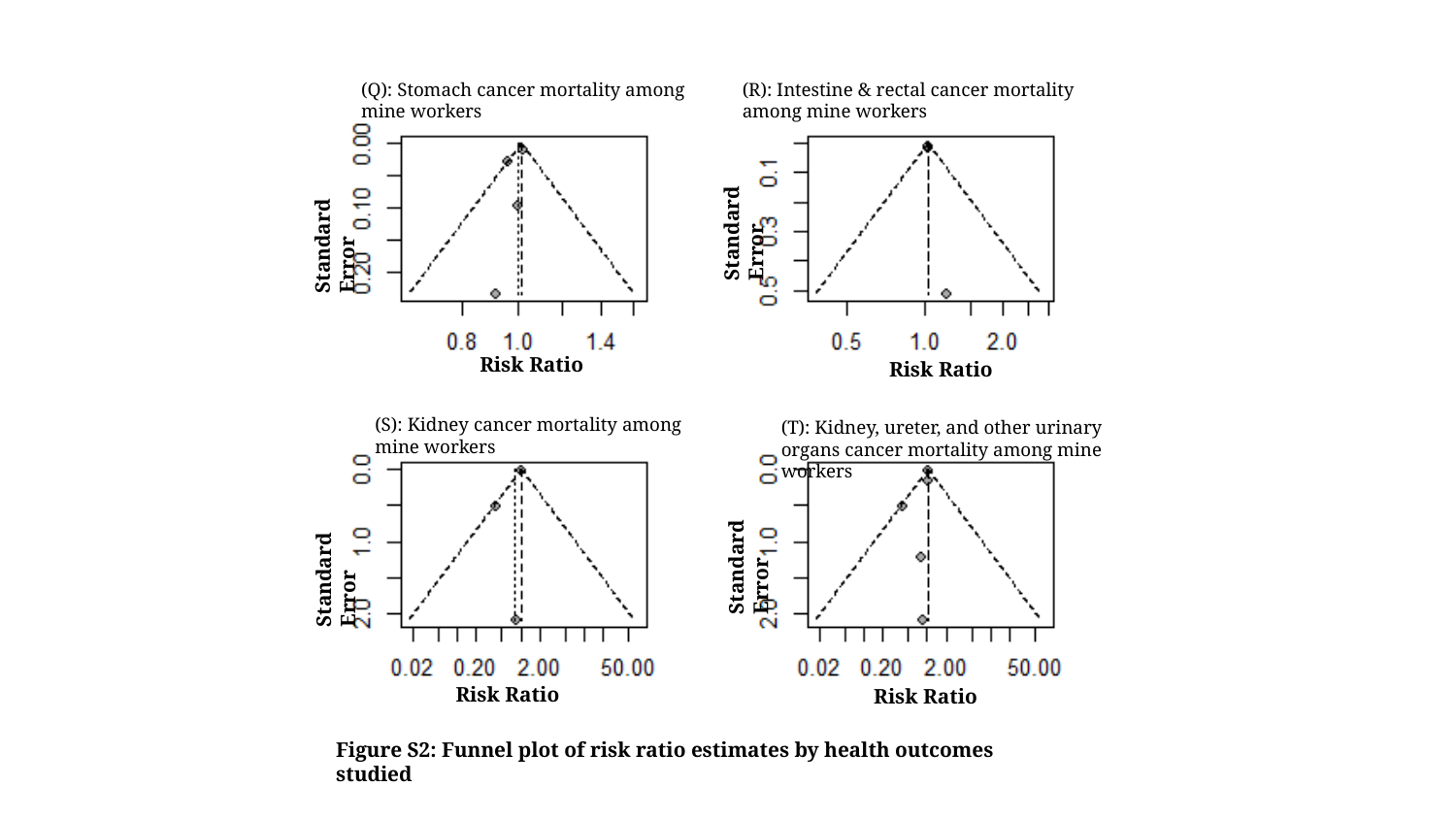

(Q): Stomach cancer mortality among mine workers
(R): Intestine & rectal cancer mortality among mine workers
Standard Error
Standard Error
Risk Ratio
Risk Ratio
(S): Kidney cancer mortality among mine workers
(T): Kidney, ureter, and other urinary organs cancer mortality among mine workers
Standard Error
Standard Error
Risk Ratio
Risk Ratio
Figure S2: Funnel plot of risk ratio estimates by health outcomes studied

## Slide 7
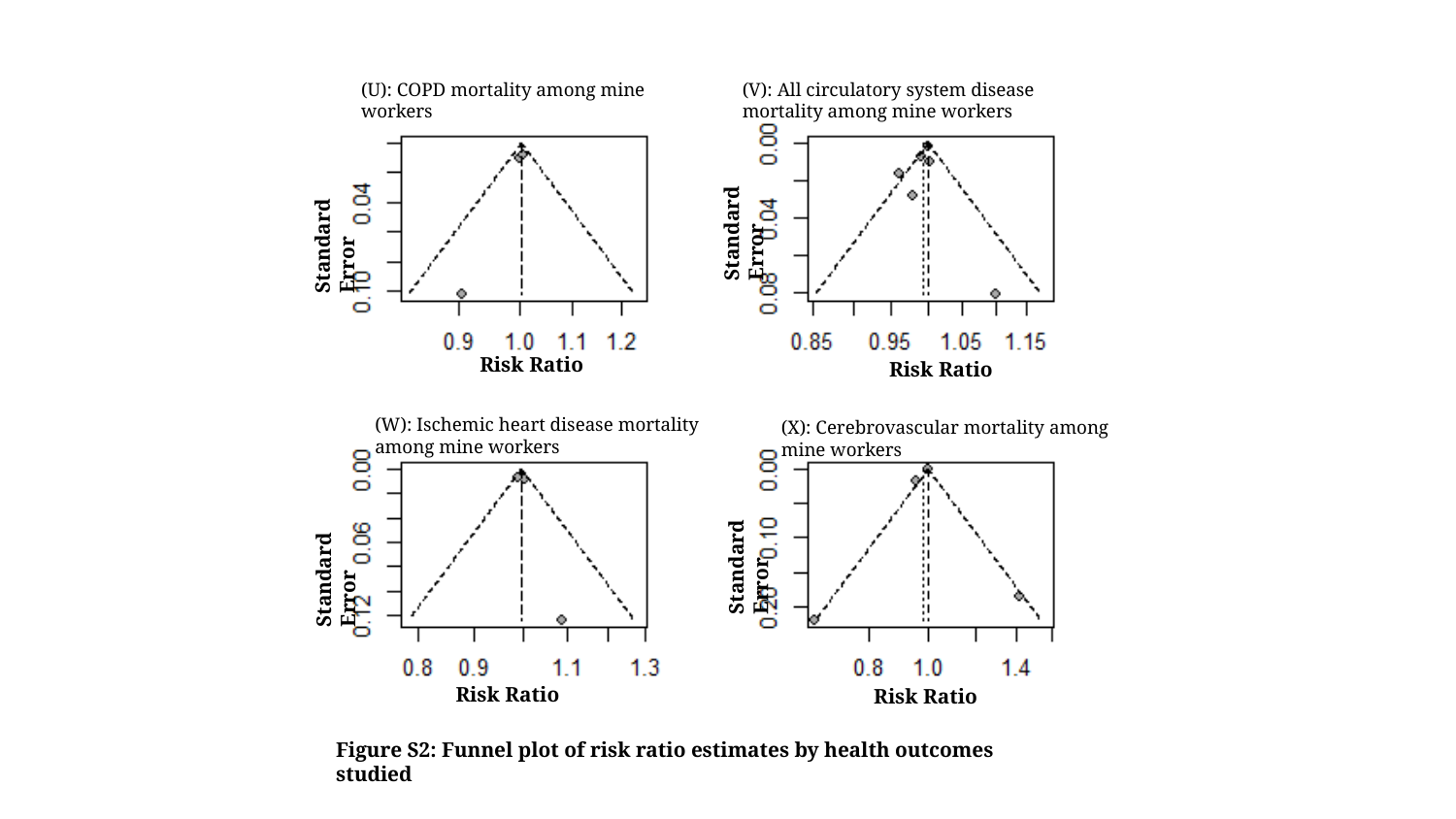

(U): COPD mortality among mine workers
(V): All circulatory system disease mortality among mine workers
Standard Error
Standard Error
Risk Ratio
Risk Ratio
(W): Ischemic heart disease mortality among mine workers
(X): Cerebrovascular mortality among mine workers
Standard Error
Standard Error
Risk Ratio
Risk Ratio
Figure S2: Funnel plot of risk ratio estimates by health outcomes studied
