# Supplementary material for: Radon exposure and potential health effects other than lung cancer: a systematic review and meta-analysis
Source: Front Public Health. 2024 Sep 25;12:1439355. doi: 10.3389/fpubh.2024.1439355 (PMC11461271; doi:10.3389/fpubh.2024.1439355)
Supplement: Supplementary file 4 [file Presentation_3.pptx]

## Slide 1
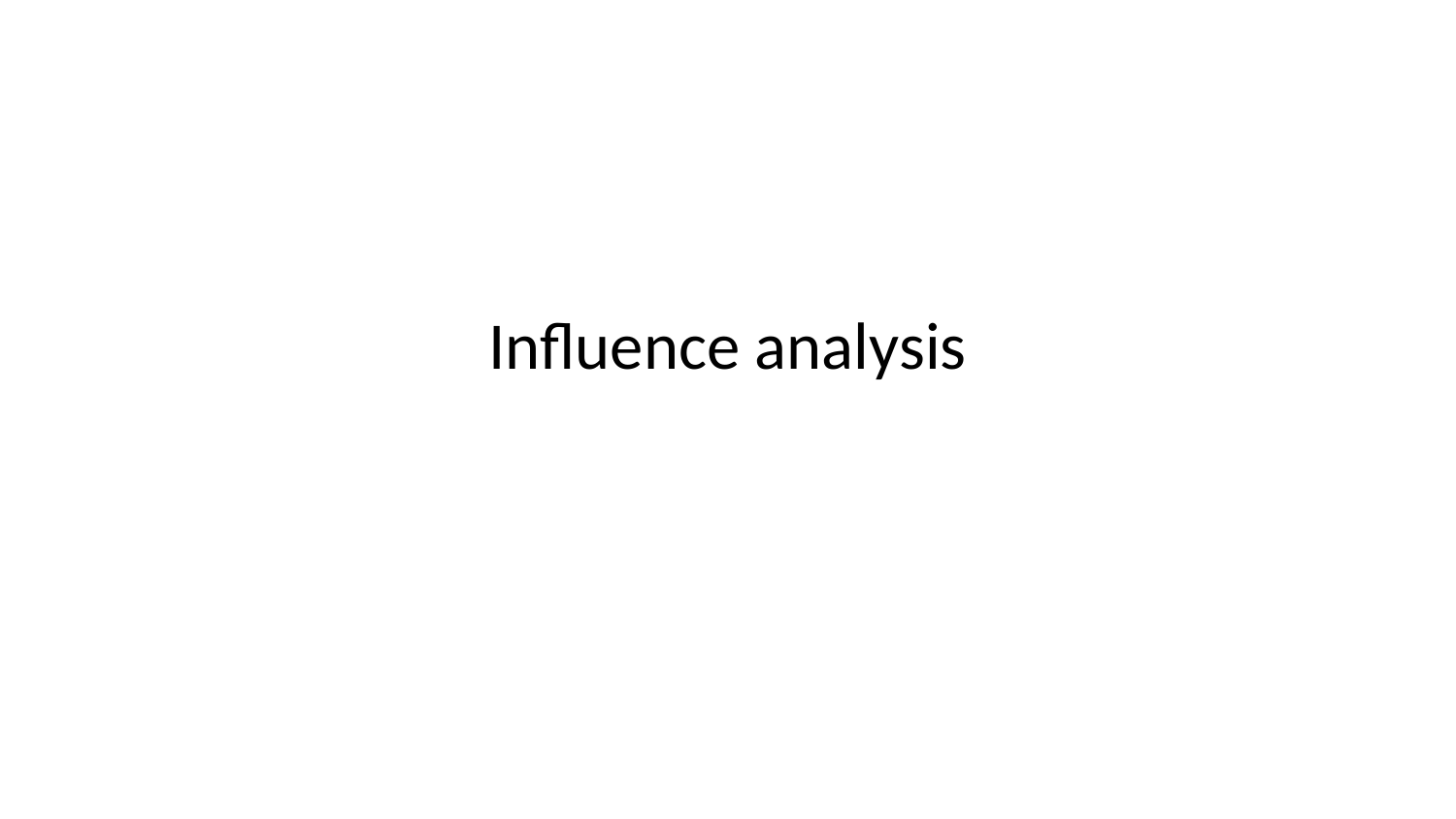

# Influence analysis

## Slide 2
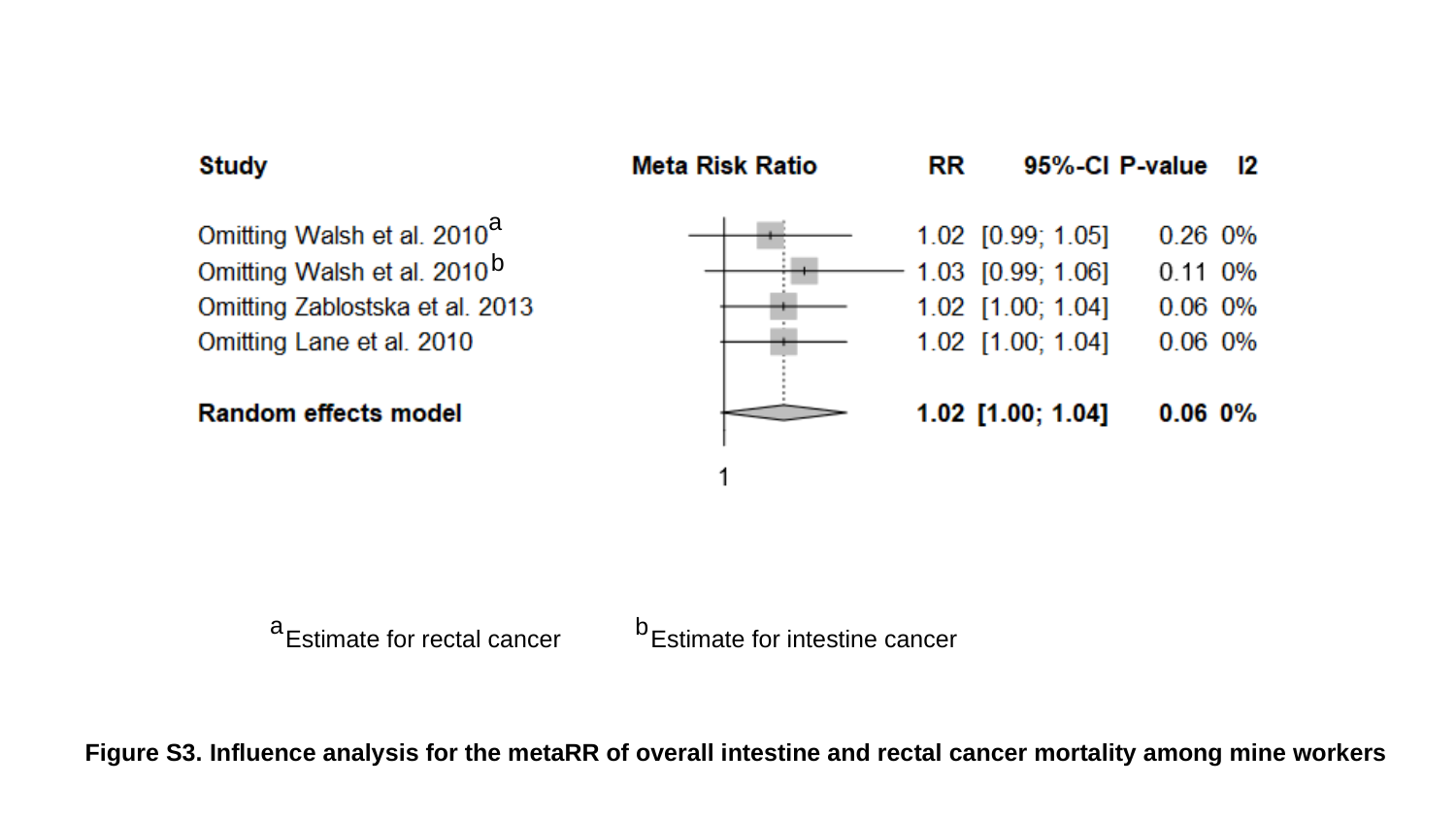

a
b
a
Estimate for rectal cancer
b
Estimate for intestine cancer
Figure S3. Influence analysis for the metaRR of overall intestine and rectal cancer mortality among mine workers

## Slide 3
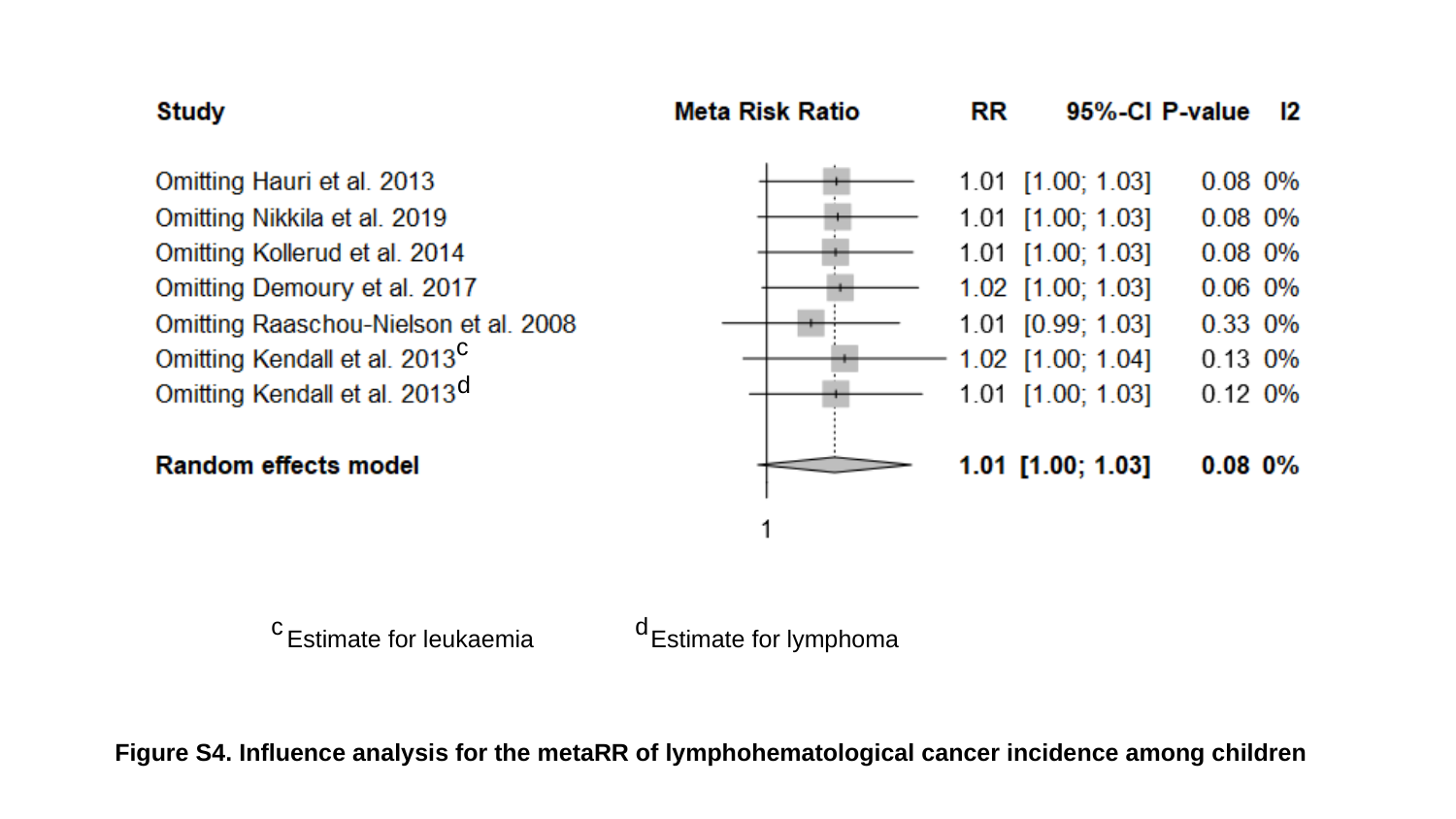

c
d
d
Estimate for lymphoma
c
Estimate for leukaemia
a
b
Figure S4. Influence analysis for the metaRR of lymphohematological cancer incidence among children
