# Supplementary material for: Radon exposure and potential health effects other than lung cancer: a systematic review and meta-analysis
Source: Front Public Health. 2024 Sep 25;12:1439355. doi: 10.3389/fpubh.2024.1439355 (PMC11461271; doi:10.3389/fpubh.2024.1439355)
Supplement: Supplementary file 5 [file Table_2.DOCX]

Supplementary Material

Table S1. List of studies completely excluded from the systematic review and meta-analysis

| **Author, year [reference]** | **Location** | **Reason of exclusion** |
| --- | --- | --- |
| Palmer et al., 2023 (1) | USA | Only effects of joint exposures to radon and other indicators of air quality were studied, therefore no result for the proper effect of radon was available |
| Rage et al. 2014 (2) | France | Overlap study population: the present study included people hired from 1956 who are already included in another one (Rage et al. 2018), with same diseases studied, same estimates. |
| Vacquier et al. 2007 (3) | France | A more recent study (Rage et al. 2018) of an extension of the same cohort exists |
| Laurier et al. 2004 (4) | France | A more recent study (Rage et al. 2018) of an extension of the same cohort exists |
| Vacquier et al. 2011 (5) | France | A more recent study (Rage et al. 2018) of the extended of the same cohort exists |
| Tirmarchel et al. 1993 (6) | France | A more recent study (Rage et al. 2018) of an extension of the same cohort exists |
| Baccini et al. 2008 (7) | | Wrong subject (heat-related mortality) |
| Alarcón-Capel et al. 2021 (8) | Spain | Wrong study type (a systematic review) |
| Sarin et al. 2021 (9) | Hungary | Dosimetry, estimation of dose to specific organ coefficients |
| Elzain et al. 2021 (10) | Sudan | Quantitative risk assessment (risk predictions) |
| Hae-Seong et al. 2018 (11) |  | Wrong outcome (lung cancer) |
| Keil et al. 2015 (12) | USA | Wrong outcome (lung cancer and total death) |
| Bryan et al. 1999 (13) | | Wrong topic (Statistical methods) |
| Carta et al. 1994 (14) | Italie | Another study (Cocco et al. 1994) covering the same cohort and outcomes with a longer follow-up period exists |
| Schuttmann et al. 1993 (15) | Germany | Wrong outcome (lung cancer) and wrong publication type (review) |
| Vienneau et al. 2017 (16) | Switzerland | An updated study exists (Boz et al. 2022) |
| Sade et al. 2019 (17) | USA | The outcome of interest was all-cause mortality. But in our study, we are looking for risk other than lung cancer |
| Axelson et al. 2002 (18) | Sweden | Exposure: gamma-radiation |
| Evrard et al. 2006 (19) | France | A study exists (Berlivet et al. 2021) with extended follow-up, same estimates), but different categorisations for radon. Department vs municipal - level radon exposure. The conclusions of both studies are the same |
| Evrard et al. 2005 (20) | France | A study exists (Berlivet et al. 2021) with extended follow-up, same estimates), but different categorisations for radon. The conclusions of both studies are the same |
| Charles. 2007 (21) |  | Review and dosimetry |
| Lazar et al. 2005 (22) | Hungary | The outcome of interest was all-cancer mortality. But in our study, we are looking for risk other than lung cancer |
| Lazar et al. 2003 (23) | Hungary | The outcome of interest was all-cancer mortality. But in our study, we are looking for risk other than lung cancer |
| Gilmore et al. 2003 (24) | Irland | Not specific to radon |
| Bittany et al. 2022 (25) | USA | Wrong outcome |
| Kreuzer et al. 2021 (26) | Germany | Duplicate with the study Kreuzer et al. 2021 |

Table S1. List of studies completely excluded from the systematic review and meta-analysis (end)

| **Author, year [reference]** | **Location** | **Reason of exclusion** |
| --- | --- | --- |
| Peng et al. 2020 (27) | USA | Wrong outcome (gene expression) |
| Shakoor et al. 2021 (28) | Pakistan | Dosimetry and radon measurement in drinking water |
| Entezari et al. 2020 (29) | Iran | The same study does exist, same study population, same year and journal of publication with two different first author (Duplicate with the study Fathabadi et al. 2020) |
| Brauner et al. 2010 (30) | Denmark | Results are included in another study (Raaschou-Nielsen et al. 2008) |
| Henshaw et al. 2002 (31) | UK | Letter to the editor concerning the study Cartwrigh et al. 2002 |
| Cartwright et al. 2002 (32) | UK | Gamma radiation |
| Jagger et al. 1998 (33) | USA | Wrong outcome (all cancer and lung & bronchus cancer) and wrong exposure (all natural background radiation) |
| Thorne et al. 1996 (34) |  | Letter to the editor concerning the original study Thorne et al. 1996 already included |
| Parker et al. 1996 (35) |  | Editorial including the study Thorne et al. 1996 |
| Tirmarchel et al. 1993 (36) | France | wrong outcome (all cancer, lung cancer) |
| Jacomino et al. 1996 (37) | Brazil | Dosimetry and organ-dose risk estimation |
| Kaatsch et al. 1996 (38) | Germany | Exploratory and descriptive study on risk factor of childhood leukemia. No result available regarding radon exposure effects. See study Kaletsch et al. 1999 |
| Jean-François Lacronique |  | Wrong publication type (meta-analysis |
| Shimek et al. 1991 | USA | Report (review) |
| Akan et al. 2014 (39) | Turkey | Not specific to radon |
| Toth et al. 1998 (40) | Hungry | Wrong outcome (all cancer together) |
| Amandus et al. 1991 (41) | USA | Wrong outcome (Silicosis and lung cancer) |
| Kreuzer et al. 2016 (42) | Germany | Not specific to radon exposure. The Low and high LET included radon gas, radon progeny, LLR |
| Mohner et al. 2010 (43) | Germany | Exposure not specific to radon exposure. The occupational ionizing radiation is exposed based on radon gas, radon progeny, LLR, and the radiographic chest x ray |
| Morfeld et al. 1997 (44) | Germany | Not a reliable study population for radon related health risk but dust (coal miners) |
| Kreuzer et al. 2008 (45) | Germany | Results are included in another studies (Kreuzer et al. ; Walsh et al. 2010) |
| Toti et al. 2005 (46) | Italy | Same as the study Forestière et al. 1998 with frequentist method for OR estimation |
| Roscoe et al. 1995 (47) | USA | Outcomes are already studied in the study Schubauer- Berigan et al. 2009; and Richardson et al. 2021, which got an extended follow-up (1960-2005) and 1958–2012 respectively |
| Villeneuve et al. 1997 (48) | Canada | Already included and studied in the study Villeneuve et al. 2023, which is the updated cohort |
| Villeneuve et al. 2007 (49) | Canada | Already included and studied in the study Villeneuve et al. 2023, which is the updated cohort |

**References**

1. Palmer JD, Prasad RN, Cioffi G, Kruchtko C, Zaorsky NG, Trifiletti DM, et al. Exposure to radon and heavy particulate pollution and incidence of brain tumors. Neuro Oncol. 14 févr 2023;25(2):407‑17.

2. Rage E, Caër-Lorho S, Drubay D, Ancelet S, Laroche P, Laurier D. Mortality analyses in the updated French cohort of uranium miners (1946–2007). International Archives of Occupational and Environmental Health. août 2015;88(6):717‑30.

3. Vacquier B, Caer S, Rogel A, Feurprier M, Tirmarche M, Luccioni C, et al. Mortality risk in the French cohort of uranium miners: Extended follow-up 1946-1999. Occupational and Environmental Medicine. sept 2008;65(9):597‑604.

4. Laurier D, Tirmarche M, Mitton N, Valenty M, Richard P, Poveda S, et al. An update of cancer mortality among the French cohort of uranium miners: Extended follow-up and new source of data for causes of death. European Journal of Epidemiology. févr 2004;19(2):139‑46.

5. Vacquier B, Rage E, Leuraud K, Caër-Lorho S, Houot J, Acker A, et al. The influence of multiple types of occupational exposure to Radon, Gamma Rays and Long-Lived Radionuclides on Mortality Risk in the French \textquotedblpost-55\textquotedbl Sub-cohort of Uranium Miners: 1956-1999. Radiation Research. déc 2011;176:796‑806.

6. Tirmarche M, Raphalen A, Allin F, Chameaud J, Bredon P. Mortality of a cohort of French uranium miners exposed to relatively low radon concentrations. Br J Cancer. mai 1993;67(5):1090‑7.

7. Baccini M, Biggeri A, Accetta G, Kosatsky T, Katsouyanni K, Analitis A, et al. Heat Effects on Mortality in 15 European Cities. Epidemiology. sept 2008;19(5):711.

8. Alarcón-Capel E, Ruano-Ravina A, Barros-Dios JM. Exposición al radón y cáncer genitourinario en mineros. Gaceta Sanitaria. 1 janv 2021;35(1):72‑80.

9. Sarin A, Kaushal A, Bajwa BS, Sharma N. Quantification of doses and health risks to organs and tissues corresponding to different age groups due to radon in water. J Radioanal Nucl Chem. 1 déc 2021;330(3):643‑55.

10. Elzain AEA. Assessment of environmental health risks due to indoor radon levels inside workplaces in Sudan. International Journal of Environmental Analytical Chemistry. 3 mai 2023;103(6):1394‑410.

11. Nam HS, Ryu JS. Indoor Radon and Lung Cancer: National Radon Action Plans Are Urgently Required. Yonsei Med J. 1 nov 2018;59(9):1013‑4.

12. Keil AP, Richardson DB, Troester MA. Healthy Worker Survivor Bias in the Colorado Plateau Uranium Miners Cohort. Am J Epidemiol. 15 mai 2015;181(10):762‑70.

13. Langholz B, Thomas D, Xiang A, Stram D. Latency analysis in epidemiologic studies of occupational exposures: Application to the Colorado plateau uranium miners cohort. American Journal of Industrial Medicine. 1999;35(3):246‑56.

14. Carta P, Cocco P, Picchiri G. Lung cancer mortality and airways obstruction among metal miners exposed to silica and low levels of radon daughters. Am J Ind Med. avr 1994;25(4):489‑506.

15. Schüttmann W. Schneeberg lung disease and uranium mining in the saxon ore mountains (Erzgebirge). American Journal of Industrial Medicine. 1993;23(2):355‑68.

16. Vienneau D, de HK, Hauri D, Vicedo -Cabrera Ana M., Schindler C, Huss A, et al. Effects of Radon and UV Exposure on Skin Cancer Mortality in Switzerland. Environmental Health Perspectives. 125(6):067009.

17. Sade MY, Blomberg AJ, Zanobetti A, Schwartz JD, Coull BA, Kloog I, et al. County-level radon exposure and all-cause mortality risk among Medicare beneficiaries. Environ Int. 11 juin 2019;130:104865.

18. Axelson O, Fredrikson M, Åkerblom G, Hardell L. Leukemia in Childhood and Adolescence and Exposure to Ionizing Radiation in Homes Built from Uranium-Containing Alum Shale Concrete. Epidemiology. mars 2002;13(2):146.

19. Evrard AS, Hémon D, Billon S, Laurier D, Jougla E, Tirmarche M, et al. CHILDHOOD LEUKEMIA INCIDENCE AND EXPOSURE TO INDOOR RADON, TERRESTRIAL AND COSMIC GAMMA RADIATION. Health Physics. juin 2006;90(6):569.

20. Evrard AS, Hémon D, Billon S, Laurier D, Jougla E, Tirmarche M, et al. Ecological association between indoor radon concentration and childhood leukaemia incidence in France, 1990-1998. Eur J Cancer Prev. avr 2005;14(2):147‑57.

21. Charles MW. Radon exposure of the skin: II. Estimation of the attributable risk for skin cancer incidence. J Radiol Prot. août 2007;27(3):253.

22. Lázár I, Tóth E, Köteles GJ, Puhó E, Czeizel AE. An inverse association between cancer mortality rate of women and residential radon in 34 Hungarian villages. J Radioanal Nucl Chem. 1 oct 2005;266(1):43‑8.

23. Lázár I, Tóth E, Marx G, Cziegler I, Köteles G. Effects of residential radon on cancer incidence. Journal of Radioanalytical and Nuclear Chemistry. 1 nov 2004;258(3):519‑24.

24. Gilmore M, Grennan E. A Pilot Study of the Relationship Between Multiple Sclerosis and the Physical Environment in Northwest Ireland. Environmental Geochemistry and Health. 1 mars 2003;25(1):157‑63.

25. Taylor BK, Smith OV, Miller GE. Chronic Home Radon Exposure Is Associated with Higher Inflammatory Biomarker Concentrations in Children and Adolescents. International Journal of Environmental Research and Public Health. janv 2023;20(1):246.

26. Kreuzer M, Deffner V, Schnelzer M, Fenske N. Mortality in Underground Miners in a Former Uranium Ore Mine–Results of a Cohort Study Among Former Employees of Wismut AG in Saxony and Thuringia. Dtsch Arztebl Int. 29 janv 2021;118(4):41‑8.

27. Peng C, DuPre N, VoPham T, Heng YJ, Baker GM, Rubadue CA, et al. Low dose environmental radon exposure and breast tumor gene expression. BMC Cancer. 28 juill 2020;20(1):695.

28. Shakoor H, Jehan N, Khan S, Khattak NU. Investigation of Radon Sources, Health Hazard and Risks assessment for children using analytical and geospatial techniques in District Bannu (Pakistan). International Journal of Radiation Biology. 3 juin 2022;98(6):1176‑84.

29. Entezari M, Ehrampoush MH, Rahimdel A, Shahi MA, Keyghobady N, Jalili M, et al. Is there a relationship between homes’ radon gas of MS and non-MS individuals, and the patients’ paraclinical magnetic resonance imaging and visually evoked potentials in Yazd-Iran? Environ Sci Pollut Res. 1 févr 2021;28(7):8907‑14.

30. Bräuner EV, Andersen CE, Andersen HP, Gravesen P, Lind M, Ulbak K, et al. Is there any interaction between domestic radon exposure and air pollution from traffic in relation to childhood leukemia risk? Cancer Causes Control. nov 2010;21(11):1961‑4.

31. Henshaw DL. Radon and childhood cancer. Br J Cancer. nov 2002;87(11):1336‑7.

32. UK Childhood Cancer Study Investigators. The United Kingdom Childhood Cancer Study of exposure to domestic sources of ionising radiation: 2: gamma radiation. Br J Cancer. 5 juin 2002;86(11):1727‑31.

33. Jagger J. Natural Background Radiation and Cancer Death in Rocky Mountain States and Gulf Coast States. Health Physics. oct 1998;75(4):428.

34. Thorne R, Foreman NK, Mott MG. Radon exposure and incidence of paediatric malignancies. European Journal of Cancer. 1 déc 1996;32(13):2371‑2.

35. Parker L, Craft AW. Radon and childhood cancers. European Journal of Cancer. 1 févr 1996;32(2):201‑4.

36. Kreuzer M, Sogl M, Brüske I, Möhner M, Nowak D, Schnelzer M, et al. Silica dust, radon and death from non-malignant respiratory diseases in German uranium miners. Occup Environ Med. 1 déc 2013;70(12):869‑75.

37. Jacomino VF, Bellintani SA, Oliveira J, Mazzilli BP, Fields DE, Sampa MH, et al. Estimates of cancer mortality due to the ingestion of mineral spring waters from a highly natural radioactive region of Brazil. Journal of Environmental Radioactivity. 1 janv 1996;33(3):319‑29.

38. Kaatsch P, Kaletsch U, Krummenauer F, Meinert R, Miesner A, Haaf G, et al. Case control study on childhood leukemia in Lower Saxony, Germany. Basic considerations, methodology, and summary of results. Klin Padiatr. 1996;208(4):179‑85.

39. Akan Z, Baskurt B, Asliyuksek H, Kam E, Yilmaz A, Yuksel MB, et al. Environmental radioactivity and high incidence rates of stomach and esophagus cancer in the Van Lake region: a causal relationship? Asian Pac J Cancer Prev. 2014;15(1):375‑80.

40. Tóth E, Lázár I, Selmeczi D, Marx G. Lower cancer risk in medium high radon. Pathol Oncol Res. 1998;4(2):125‑9.

41. Amandus H, Costello J. Silicosis and lung cancer in U.S. metal miners. Arch Environ Health. 1991;46(2):82‑9.

42. Kreuzer M, Sobotzki C, Fenske N, Marsh JW, Schnelzer M. Leukaemia mortality and low-dose ionising radiation in the WISMUT uranium miner cohort (1946–2013). Occup Environ Med. 1 avr 2017;74(4):252‑8.

43. Möhner M, Gellissen J, Marsh JW, Gregoratto D. OCCUPATIONAL AND DIAGNOSTIC EXPOSURE TO IONIZING RADIATION AND LEUKEMIA RISK AMONG GERMAN URANIUM MINERS. Health Physics. sept 2010;99(3):314.

44. Morfeld P, Lampert K, Ziegler H, Stegmaier C, Dhom G, Piekarski C. Overall mortality and cancer mortality of coal miners: Attempts to adjust for healthy worker selection effects. The Annals of Occupational Hygiene. 1 janv 1997;41:346‑51.

45. Kreuzer M, Walsh L, Schnelzer M, Tschense A, Grosche B. Radon and risk of extrapulmonary cancers: results of the German uranium miners’ cohort study, 1960-2003. Br J Cancer. 2 déc 2008;99(11):1946‑53.

46. Toti S, Biggeri A, Forastiere F. Adult myeloid leukaemia and radon exposure: a Bayesian model for a case-control study with error in covariates. Statistics in Medicine. 2005;24(12):1849‑64.

47. Roscoe RJ, Deddens JA, Salvan A, Schnorr TM. Mortality among Navajo uranium miners. Am J Public Health. avr 1995;85(4):535‑40.

48. Villeneuve PJ, Morrison HI. Coronary heart disease mortality among Newfoundland fluorspar miners. Scand J Work Environ Health. juin 1997;23(3):221‑6.

49. Villeneuve PJ, Lane RSD, Morrison HI. Coronary heart disease mortality and radon exposure in the Newfoundland fluorspar miners’ cohort, 1950–2001. Radiat Environ Biophys. 1 août 2007;46(3):291‑6.
