# Supplementary material for: Radon exposure and potential health effects other than lung cancer: a systematic review and meta-analysis
Source: Front Public Health. 2024 Sep 25;12:1439355. doi: 10.3389/fpubh.2024.1439355 (PMC11461271; doi:10.3389/fpubh.2024.1439355)
Supplement: Supplementary file 6 [file Table_3.DOCX]

Supplementary Material

Table S2. Summary of studies in children, adults in the general population and mine workers on radon exposure and malignant health outcomes’ (excluding lung cancer) rates and risks included in the systematic revue and meta-analysis

| **Reference Region Study period (mean duration in year)** | **Study design** | **Study population Sample size** | **Radon Exposure assessment** | **Adjustment** | **Health outcome** | **Cases /Controls*** | **Main results** | **NOS** | **UNSCEAR** |
| --- | --- | --- | --- | --- | --- | --- | --- | --- | --- |
| Peckham et al. 2015 (1) USA 1995 - 2011 | Ecological study | Children and pregnancies Age < 20 years at diagnosis 13 geologic regions | Arithmetic mean geological region-level : 45.97 Bq/m^3^ | sex, race, age at diagnostic, county-level SES, urbanization | All lymphoma incidence | 2147 | IRR, incidence rate ratio (95%CI) for 100.000 person-year* across radon quartile in Bq/m^3^ ≤25.90 : 1.00 (reference), >25.90 to ≤40.70 : 0.89 (0.79–1.00) >40.70 to ≤48.10 : 0.90 (0.77–1.04) >48.10 : 1.05 (0.83–1.32) ;  p-for -trend = 0.35 | _ | Moderate |
|  |  |  |  |  | Hodgkin lymphoma | 1248 | IRR (95%CI) across radon quartile in Bq/m^3^  ≤25.90 : 1.00 (reference), >25.90 to ≤40.70 : 0.83 (0.71–0.98) >40.70 to ≤48.10 : 0.94 (0.77–1.14) >48.10 : 0.87 (0.63–1.20) ;  p-for -trend = 0.20 |  |  |
|  |  |  |  |  | Non-Hodgkin lymphoma, excluding Burkitt lymphoma | 658 | IRR (95%CI) across radon quartile in Bq/m^3^ ≤25.90 : 1.00 (reference), >25.90 to ≤40.70 : 1.00 (0.82–1.21) >40.70 to ≤48.10 : 0.82 (0.64–1.05) >48.10 : 1.37 (0.95–1.97) ;  p-for -trend = 0.96 |  |  |
|  |  |  |  |  | Burkitt lymphoma | 241 | IRR (95%CI) across radon quartile in Bq/m^3^ ≤25.90 : 1.00 (reference), >25.90 to ≤40.70 : 1.01 (0.73–1.38) >40.70 to ≤48.10 : 1.04 (0.71–1.52) >48.10 : 1.33 (0.70–2.53) ;  p-for -trend = 0.54 |  |  |
|  |  |  |  |  | Diffuse Large Basal-Cell lymphoma | 315 | IRR (95%CI) across radon quartile in Bq/m^3^  ≤25.90 : 1.00 (reference), >25.90 to ≤40.70 : 1.11 (0.85–1.44) >40.70 to ≤48.10 : 1.01 (0.73–1.39) >48.10 : 1.73 (1.03–2.91) ;  p-for -trend = 0.22 |  |  |
| Steinbuch et al. 1999 (2) USA 1989 - 1993 | Case-control study | Children Age < 18 years at diagnosis n = 427 | Six-month direct radon measurement at participants home. Mean TWA in Bq/m3 : In cases : 49.8 Controls : 56.0 | Maternal education, family income, maternal race, and age | Acute myeloid leukaemia incidence | 173/254 | ORs (95%CI) across indoor residential TWA radon concentration categories in Bq/m3 <37 : 1.0 (Reference) 37–100 : 1.16 (0.7–1.8) >100 : 1.12 (0.6–2.0) ;  p-trend = 0.58 | 7 | Moderate |
| Lubin et al. 1998 (3) USA 1989 - 1993 | Case-control study | Children Age < 15 years at diagnosis n = 948 | At least six-month direct radon measurement at cases and controls current and previous home. Mean TWA in Bq/m3 for cases : 65.4 for control : 79.1 | Age, sex | Acute lymphoblastic leukaemia incidence | 505/443 | RR, Relative Risk (95%CI) across time weighted average radon concentration categories in Bq/m3 <37 : 1.00 (reference) 37–73 : 1.22 (0.8–1.9) 74–147 : 0.82 (0.5–1.4) ≥148 : 1.02 (0.5–2.0) ;  p-trend = 0.18 | 7 | Moderate |
| Collman et al. 1991 (4) USA 1950 - 1979 | Ecological study/cancer mortality | Children Age < 15 years at diagnosis n = 100 counties in North Carolina | County-level geometric mean radon concentration through direct measurement of radon in water supplies | Age, sex | All leukaemia mortality | 1194 | RR (95%CI) across radon categories in pCi/l 0-228 : 1.00 (reference) 229-1375 : 1.26 (1.08 - 1.47) 1376-10,692 : 1.33 (1.13 - 1.57) | _ | Moderate |
|  |  |  |  |  | Lymphoma mortality | 213 | RR (95%CI) across radon categories in pCi/l 0-228 : 1.00 (reference) 229-1375 : 1.13 (0.79 - 1.62) 1376-10,692 : 1.38 (0.95 - 2.02) |  |  |
|  |  |  |  |  | Brain and central nervous system cancer mortality | 454 | RR (95%CI) across radon categories in pCi/l 0-228 : 1.00 (reference) 229-1375 : 1.28 (1.00 - 1.62) 1376-10,692 : 1.18 (0.90- 1.54) |  |  |
|  |  |  |  |  | Kidney cancer mortality | 165 | RR (95%CI) across radon categories in pCi/l 0-228 : 1.00 (reference) 229-1375 : 0.96 (0.65 - 1.41) 1376-10,692 : 1.13 (0.74 - 1.70) |  |  |
|  |  |  |  |  | Bone cancer mortality | 108 | RR (95%CI) across radon categories in pCi/l 0-228 : 1.00 (reference) 229-1375 : 1.22 (0.75 - 1.98) 1376-10,692 : 1.06 (0.62 - 1.83) |  |  |
|  |  |  |  |  | Connective & other soft tissue cancer mortality | 176 | RR (95%CI) across radon categories in pCi/l 0-228 : 1.00 (reference) 229-1375 : 1.00 (0.69 - 1.46) 1376-10,692 : 1.11 (0.74 - 1.66) |  |  |
| Hauri et al. 2013 (5) Switzerland 2000 - 2008 | Cohort study | Children Age < 16 years at diagnosis n = 1,287,354 | Model-based residential radon exposure assessment Arithmetic mean radon concentration in Bq/m3 (range) : 85.7 ( 6.9–337.2) | Age, sex, birth order, socioeconomic status of the parents, background gamma radiation exposure, and period effects | All leukaemia incidence | 283 | HRs (95%CI) per 100 Bq/m3 increase in radon exposure : 0.90 (0.68 - 1.19) HRs across radon exposure categories indicate no statistically significant association | 8 | Moderate |
|  |  |  |  |  | Acute lymphoblastic leukaemia incidence | 225 | HRs (95%CI) per 100 Bq/m3 increase in radon exposure : 0.86 (0.63 - 1.19) HRs across radon exposure categories indicate no statistically significant association |  |  |
|  |  |  |  |  | Central nervous system tumors incidence | 258 | HRs (95%CI) per 100 Bq/m3 increase in radon exposure : 1.19 (0.91 - 1.57) HRs across radon exposure categories indicate no statistically significant association |  |  |
| Kohli et al. 2000 (6) Sweden 1979 - 1995 | Ecological study | Children Age ≤16 years n = 13 communes (53,146 children) | Radon map | Age, sex | Acute lymphoblastic leukaemia incidence | 20 | RR (95%CI) across radon risk area category at birth in Bq/m3 Low risk : 1.00 (reference) High risk : 5.67 (1.06 - 42.27) Normal risk : 4.64 (1.29 - 28.26) Normal & High risk : 4.81 (1.37 - 28.88)  Similar results were found when radon category risk since birth was considered. | _ | Moderate |
| Kaletsch et al. 1999 (7) Germany 1988 - 1993 | Case-control study | Children Age < 15 years at diagnosis n = 291 | One-year direct radon measurement at cases and controls current and previous home. Mean radon in Bq/m3 For acute leukaemia cases : 26.4 For controls : 28.5 | Degree of urbanisation, SES, date of birth and sex matched | Acute leukaemia incidence | 82/209 | OR (95%CI) associated with radon exposure > 70 Bq/m3 :  1.30 (0.32 - 5.33) | 7 | Low |
|  |  |  |  | Age group, gender, degree of urbanisation, SES | Central nervous system tumors incidence | 41/209 | OR (95%CI) associated with radon exposure > 70 Bq/m3 :  3.85 (1.26 - 11.81) |  |  |
| Kendall et al. 2013 (8) UK 1980 - 2006 | Case-control study | Children Age < 15 years at diagnosis (for cases) or enrolment (for controls) n = 64,240 | County Districts-level average radon exposure and a predictive map based on domestic measurements grouped by geological boundaries for radon of the place of residence at birth. Mean radon in Bq/m3 for controls: 16,4 | Age ans sex matched, cumulative gamma-ray exposure, quitiles SES | Lymphoblastic leukaemia incidence | 7267/9571 | RR, relative risk (95%CI) per 1000 Bq/m3 - years increase in cumulative radon exposure : 1.24 (0.94 - 1.64) RR (95%CI) per each mSv in cumulative RBM equivalent dose : 1.07 (0.98 - 1.16) | 7 | High |
|  |  |  |  |  | Acute myeloid leukaemia incidence | 1316/1737 | RR (95%CI) per 1000 Bq/m3 - years increase in cumulative radon exposure : 0.72 (0.37 - 1.40) RR (95%CI) per each mSv in cumulative RBM equivalent dose : 0.91 (0.75 - 1.10) |  |  |
|  |  |  |  |  | Other leukaemia incidence | 475/604 | RR (95%CI) per 1000 Bq/m3 - years increase in cumulative radon exposure : 1.04 (0.41 - 2.61) RR (95%CI) per each mSv in cumulative RBM equivalent dose : 1.01 (0.77 - 1.33) |  |  |
|  |  |  |  |  | All leukaemia incidence | 9058/11,912 | RR (95%CI) per 1000 Bq/m3 - years increase in cumulative radon exposure : 1.12 (0.88 - 1.43) RR (95%CI) per each mSv in cumulative RBM equivalent dose : 1.03 (0.96 - 1.11) |  |  |
|  |  |  |  |  | Hodgkin lymphoma incidence | 939/1,388 | RR (95%CI) per 1000 Bq/m3 - years increase in cumulative radon exposure : 1.07 (0.67 - 1.70) |  |  |
|  |  |  |  |  | Non-Hodgkin lymphoma incidence | 983/1302 | RR (95%CI) per 1000 Bq/m3 - years increase in cumulative radon exposure : 1.29 (0.69 - 2.39) |  |  |
|  |  |  |  |  | Lymphoma incidence | 2319/3274 | RR (95%CI) per 1000 Bq/m3 - years increase in cumulative radon exposure : 1.29 (0.69 - 2.39) |  |  |
|  |  |  |  |  | Lymphoid leukaemia + Non-Hodgkin lymphoma incidence | 8250/10,873 | RR (95%CI) per 1000 Bq/m3 - years increase in cumulative radon exposure : 1.24 (0.96 - 1.60) |  |  |
|  |  |  |  |  | All leukaemia + Non-Hodgkin lymphoma incidence | 10,041/13,214 | RR (95%CI) per 1000 Bq/m3 - years increase in cumulative radon exposure : 1.14 (0.91 - 1.43) |  |  |
|  |  |  |  |  | Brain and central nervous system tumors incidence | 6585/8997 | RR (95%CI) per 1000 Bq/m3 - years increase in cumulative radon exposure : 1.15 (0.88 - 1.50) |  |  |
| Thorne et al. 1996 (9) | Ecological study | Children Age < 15 years at diagnostic n = 2 counties (Devon and Cornwall) including 283 postcode sectors | Postcode sector-level average radon exposure of the residence at time of diagnostic through direct radon measurement in homes. Mean radon in Bq/m3 For postcode sectors with average radon ≥ 100 Bq/m3 : 183 For postcode sectors with average radon <100 Bq/m3 : 57 | _ | All leukaemia incidence | 108 | Incidence rate in postcode sectors with average radon ≥ 100 Bq/m3 compare with those with average radon <100 Bq/m3 : p = 0.60 | _ | Moderate |
|  |  |  |  |  | Acute lymphoblastic leukaemia incidence | _ | p = 0.28 |  |  |
|  |  |  |  |  | Acute myeloid leukaemia incidence | _ | p = 0.11 |  |  |
|  |  |  |  |  | Lymphoma incidence | 30 | p = 0.34 |  |  |
|  |  |  |  |  | Brain and spinal cancer incidence | 68 | p = 0.09 |  |  |
|  |  |  |  |  | Neuroblastoma incidence | 17 | p = 0.02 |  |  |
|  |  |  |  |  | Retinoblastoma incidence | 3 | p = 0.55 |  |  |
|  |  |  |  |  | Kidney cancer incidence | 24 | p = 0.99 |  |  |
|  |  |  |  |  | Liver cancer incidence | 5 | p = 0.66 |  |  |
|  |  |  |  |  | Bone cancer incidence | 16 | p = 0.08 |  |  |
|  |  |  |  |  | Soft tissue sarcomas incidence | 17 | p = 0.79 |  |  |
|  |  |  |  |  | Gonadal and germ cell cancer incidence | 6 | p = 0.67 |  |  |
|  |  |  |  |  | Epithelial cancer incidence | 7 | p = 0.70 |  |  |
| Foreman et al. 1994 (10) UK 1976 - 1985 | Ecological study | Children Age < 15 years at diagnosis n = 4 counties (including Devon and Cornwall counties) | County-level radon exposure | Age | All leukaemia incidence | 245 | Incidence rate in counties with high radon exposure (Devon, Cornwall) compare with counties with low radon exposure (Avon, Gloucestershire) : p = 0.30 | _ | Low |
| Nikkila et al. 2019 (11) Finland 1990 - 2011 | Case-control study | Children Age < 15 years at diagnostic (for cases) or enrolment (for controls) n = 6443 | Model-based residential radon exposure in Bq/m3. Median cumulative radon exposure for cases (IQR) : 301 (121 - 625) Median time-weighted average indoor radon exposure for cases (IQR) : 92 (68 - 123) | Sex and year of birth matched, Down syndrome,  large birth weight, gestational duration, terrestrial  cumulative red bone marrow equivalent dose from gamma radiation and Chernobyl fallout, cumulative red bone marrow dose from CT exposure, maternal  smoking during pregnancy, parental SES and education. | All leukaemia inicdence | 922/ 5350 | OR (95%CI) per 1000 Bq/m3 - years increase in cumulative radon exposure : 1.06 (0.59–1.92) ORs per 10 Bq/m3 increase in and across quartile of TWA radon exposure indicate no statistically significant association. | 9 | High |
|  |  |  |  |  | Acute lymphoblastic leukaemia incidence | 806/NA | OR (95%CI) per 1000 Bq/m3 - years increase in cumulative radon: 1.32 (0.67 - 2.60) ORs (95%CI) per 10 Bq/m3 increase in and across quartile of TWA radon exposure indicate no statistically significant association. |  |  |
|  |  |  |  |  | Others leukaemia incidence | 183/NA | OR (95%CI) per 1000 Bq/m3 - years increase in cumulative radon: 0.42 (0.09 - 1.89) ORs (95%CI) per 10 Bq/m3 increase in and across quartile of TWA radon exposure indicate no statistically significant association. |  |  |
|  |  |  |  |  | Pre-Basal- Acute lymphoblastic leukaemia incidence | 735/NA | OR (95%CI) per 1000 Bq/m3 - years increase in cumulative radon: 1.59 (0.74 - 3.38) ORs (95%CI) per 10 Bq/m3 increase in and across quartile of TWA radon exposure indicate no statistically significant association. |  |  |
| Chen et al. 2019 (12) Canada 2006 - 2015 | Ecological study | Children Age < 15 years at diagnosis n = 33 census metropolitan areas | Census metropolitan area-level average radon exposure based on direct radon measurement in home | Age, sex, census metropolitan area, 24 sociodemographic peer groups | Leukaemia and lymphoma incidence | NA | The linear correlation coefficient r, and p-value between incidence rates and radon exposure suggest no association between radon exposure and leukaemia and lymphoma AML in males : r = 0.05 ; p-value = 0.213 | _ | Low |
| Kollerud et al. 2014 (13) Norway 1967 - 2009 | Cohort study | Children Age < 15 years at diagnostic n = 712,674 | Small area (radium of 300 m to 2000 m from the residence addresses) or the nearest neighbor average radon exposure Mean radon exposure : 91 Bq/m3 | Parity, birth weight, sex, congenital malformations, family income, mother and father’s level of education | All leukaemia incidence | 431 | HR (95%CI) per 100 Bq/m3 increase in radon exposure : 1.00 (0.87 - 1.15) HRs across radon exposure categories indicate no statistically significant association | 8 | High |
|  |  |  |  |  | Acute lymphoblastic leukaemia incidence | 324 | HR (95%CI) per 100 Bq/m3 increase in radon exposure : 0.97 (0.83 - 1.15) HRs across radon exposure categories indicate no statistically significant association |  |  |
|  |  |  |  |  | Central nervous system cancer incidence | 427 | HR (95%CI) per 100 Bq/m3 increase in radon exposure : 1.13 (0.99 - 1.28) HRs across radon exposure categories indicate no statistically significant association |  |  |
|  |  |  |  |  | Leukaemia + Central nervous system cancer incidence | 848 | HR (95%CI) per 100 Bq/m3 increase in radon exposure : 1.06 (0.97 - 1.17) HRs across radon exposure categories indicate no statistically significant association |  |  |
| Yoshinaga et al. 2005 (14) Japan 1999 - 2002 | Case-control study | Children Age < 15 years at diagnostic (for cases) or enrollment (for controls) n = 458 | Direct long term (6 months) radon measurement in individuals home in Bq/m3 Mean radon for cases : 17 for controls : 18 | Age, sex, magnetic field level in subject’s bedroom, maternal education background, and maternal medical X-ray exposure during pregnancy | Acute leukaemia incidence | 227/231 | RR, relative risk (95%CI) across radon concentration categories < 20 : 1.00 (reference)  20–49 : 1.00 (0.62–1.62) 50–99 : 1.57 (0.47–5.22) 100+ : 2.05 (0.18–23.4) ;  p for trend=0.52 | 6 | Moderate |
| Berlivet et al. 2021 (15) France 1990 - 2009 | Ecological study | Children Age < 15 years at diagnosis n = 35,800 municipalities | Model-based municipality-level average radon exposure of the municipality of residence at birth. Mean average radon in Bq/m3 (IQR) : 65 (39–79) | Size of urban unit, deprivation index (FDep), UV-radiation, gamma radiation | Acute leukaemia | 6059 | IRR, incidence rate ratio (95%CI) per 100 Bq/m3 increase in radon exposure : 0.96 (0.89 - 1.04) IRR (95%CI) per 1000 Bq/m3 - years increase in cumulative radon exposure : 0.89 (0.80 - 0.99) SIRs across cumulative radon concentration categories indicate no association, except a marginally significant low incidence rates than expected for exposure at 12.5 to 35.1 | _ | Moderate |
|  |  |  |  |  | Acute lymphoblastic leukaemia incidence | 4982 | IRR (95%CI) per 100 Bq/m3 increase in radon exposure : 0.95 (0.87 - 1.04) IRR (95%CI) per 1000 Bq/m3 - years increase in cumulative radon exposure : 0.91 (0.81 - 1.02) SIRs across cumulative radon concentration categories indicate no statistically significant association |  |  |
|  |  |  |  |  | BCP-ALL | 4156 | IRR (95%CI) per 100 Bq/m3 increase in radon exposure : 0.97 (0.88 - 1.06) IRR (95%CI) per 1000 Bq/m3 - years increase in cumulative radon exposure : 0.93 (0.81 - 1.06) SIRs across cumulative radon concentration categories indicate no statistically significant association |  |  |
|  |  |  |  |  | AML | 957 | IRR (95%CI) per 100 Bq/m3 increase in radon exposure : 0.98 (0.80 - 1.18) IRR (95%CI) per 1000 Bq/m3 - years increase in cumulative radon exposure : 0.78 (0.59 - 1.04). SIRs across cumulative radon concentration categories no association, except a low incidence rate for exposure at 27.0 - 35.1 Bq/m3 |  |  |
| Demoury et al. 2017 (16) France 1990 - 2009 | Ecological study | Children Age < 15 years at diagnostic n = 36,326 municipalities | Model-based municipality-level average radon exposure of the municipality of residence at birth. | Age | All leukaemia incidence | 9056 | SIR per 100 Bq/m3 increase in radon exposure : 1.01 (0.91 - 1.12) SIR (95%CI) per mSv increase AL : 1.00 (0.97 - 1.02) SIRs across red bone marrow dose categories from radon exposure incidacte no association. SIRs across radon concentration categories indicate no association, except a marginally low incidence rate at 12.5–37.1 Bq/m3 | _ | Moderate |
|  |  |  |  |  | Acute lymphoblastic leukaemia incidence | 7434 | SIR per 100 Bq/m3 increase in radon exposure : 1.01 (0.86 - 1.19) SIRs across radon concentration categories indicate no association, except a marginally low/high incidence rate at some exposure level |  |  |
|  |  |  |  |  | Acute myeloid leukaemia incidence | 1465 | SIR per 100 Bq/m3 increase in radon exposure : 0.99 (0.81 - 1.19) SIRs across radon concentration categories indicate no statistically significant association |  |  |
| Demoury et al. 2017 (16) France 2002 - 2007 | Case-control | Children Age < 15 years at diagnostic n = 32,763 | Model-based municipality-level average radon exposure of the municipality of residence at diagnosis. Mean radon at the residence for controls in Bq/m3 : 67.8 | Age | Acute leukaemia incidence | 2763/30,000 | OR per 100 Bq/m3 increase in radon concentration : 0.98 (0.90 -1.07) ORs across radon concentration categories indicate no statistically significant association | _ | Moderate |
|  |  |  |  |  | Acute lymphoblastic leukaemia incidence | 2283/NA | OR per 100 Bq/m3 increase in radon concentration : 0.99 (0.90 - 1.09) ORs across radon concentration categories indicate a positive association for exposure level above 37.7 to 62.8 Bq/m3 |  |  |
|  |  |  |  |  | Acute myeloid leukaemia incidence | 418/NA | OR per 100 Bq/m3 increase in radon concentration : 0.90 (0.71 - 1.13) ORs across radon concentration categories indicate no statistically significant association |  |  |
| Berlivet et al. 2020 (17) France 2000 - 2012 | Ecological study | Children Age < 15 years at diagnostic n = 36,261 municipalities | Model-based municipality-level average radon exposure of the municipality of residence at diagnostic. Mean average radon in Bq/m3 (IQR) : 67.8 (41.0 -82.0) | Age (for SIR); gamma radiation (for IRR) | Central nervous system tumors incidence | 1079 | IRR (95%CI) per 100 Bq/m3 increase in radon exposure : 0.99 (0.91-1.07) IRR (95%CI) per 1000 Bq/m3-years : 1.00 (0.91 - 1.09) SIRs across radon concentration categories indicate no statistically significant association, except a low incidence rate for exposure level > 42.9 to 49.1 | _ | Moderate |
|  |  |  |  |  | Ependymonas incidence | 532 | IRR (95%CI) per 100 Bq/m3 increase in radon exposure : 1.15 (0.90 - 1.46) IRR (95%CI) per 1000 Bq/m3-years : 1.26 (0.92 -1.74) SIRs across radon concentration categories indicate no statistically significant association |  |  |
|  |  |  |  |  | Embryonal central nervous system tumors incidencce | 1079 | IRR (95%CI) per 100 Bq/m3 increase in radon exposure : 0.96 (0.80 - 1.15) IRR (95%CI) per 1000 Bq/m3-years : 0.95 (0.74 - 1.22) SIRs across radon concentration categories indicate no statistically significant association, except a low/high incidence rate at some exposure level |  |  |
|  |  |  |  |  | Gliomas incidence | 3340 | IRR (95%CI) per 100 Bq/m3 increase in radon exposure : 0.96 (0.80 - 1.15) IRR (95%CI) per 1000 Bq/m3-years : 0.97 (0.86 - 1.09) SIRs across radon concentration categories indicate no statistically significant association, except a low/high incidence rate at some exposure level |  |  |
|  |  |  |  |  | Pilocytic astrocytomas incidence | 1215 | IRR (95%CI) per 100 Bq/m3 increase in radon exposure : 0.97 (0.83 - 1.14) IRR (95%CI) per 1000 Bq/m3-years : 0.96 (0.79 - 1.17) SIRs across radon concentration categories indicate no statistically significant association |  |  |
|  |  |  |  |  | Other gliomas | 21 | IRR (95%CI) per 100 Bq/m3 increase in radon exposure : 0.95 (0.84 - 1.08) IRR (95%CI) per 1000 Bq/m3-years : 0.97 (0.84 - 1.12) SIRs across radon concentration categorie suggest no statistically significant, except a low incidence rate at exposure level >49.1 to 55.5 Bq/m3 |  |  |
| Zlobina et al. 2022 (18) Russia, China, France 2007 - 2018 ; 2008 - 2018 ; 2011 - 2016 ; 2014 - 2017 | Pooled ecological study | Children Age < 15 years n = 4 cities | City-level outdoor radon measurement in soil samples (in Bq/m3) | _ | Leukaemia incidence | NA | Correlation between city-level incidence rates and city-level outdoor radon R-square = 0.48 ; p = 0.259 | _ | Moderate |
|  |  |  |  |  |  |  |  |  |  |
| Raaschou-Nielsen et al. 2008 (19) Denmark 1968 - 1994 | Case-control study | Children Age < 15 years n = 9097 | Model-based radon exposure estimation for residences occupied during childhood Mean in Bq/m3 (10th - 90 th) : 48 (10 - 102) | Age and sex matched | All leukaemia incidence | 1153/2306 | Rate Ratio, RR (95%CI) per 10³ Bq/m3 - years increased in cumulative radon exposure : 1.34 (0.97–1.85) RRs across cumulative residential radon exposure categories indicate no statistically significant association | 8 | High |
|  |  |  |  |  | Acute non-lymphoblastic leukaemia incidence | 150/300 | Rate Ratio, RR (95%CI) per 10³ Bq/m3 - years increased in cumulative radon exposure :  0.75 (0.34–1.62)  RRs across cumulative residential radon exposure categories indicate no statistically significant association |  |  |
|  |  |  |  |  | Acute lymphoblastic leukaemia incidence | 860/1720 | Rate Ratio, RR (95%CI) per 10³ Bq/m3 - years increased in cumulative radon exposure :  1.56 (1.05–2.30)  RRs across cumulative radon exposure indicate a statistically significant increased risk at and above 0.89 Bq/m3. |  |  |
|  |  |  |  |  | Other leukaemia incidence | 143/286 | Rate Ratio, RR (95%CI) per 10³ Bq/m3 - years increased in cumulative radon exposure : 1.34 (0.53–3.40) RRs across cumulative residential radon exposure categories indicate no statistically significant association |  |  |
|  |  |  |  |  | Central nervous tumor incidence | 922/2766 | Rate Ratio, RR (95%CI) per 10³ Bq/m3 - years increased in cumulative radon exposure : 0.92 (0.69–1.22) RRs across cumulative residential radon exposure categories indicate no statistically significant association |  |  |
|  |  |  |  |  | Lymphoma incidence | 325/1625 | Rate Ratio, RR (95%CI) per 10³ Bq/m3 - years increased in cumulative radon exposure : 0.94 (0.64–1.38) RRs across cumulative residential radon exposure categories indicate no statistically significant association |  |  |
|  |  |  |  |  | Leukaemia & Lymphoma & central nervous system tumor incidence | 2400/6697 | Rate Ratio, RR (95%CI) per 10³ Bq/m3 - years increased in cumulative radon exposure : 1.04 (0.87–1.26) RRs across cumulative residential radon exposure categories indicate no statistically significant association |  |  |
| Cartwright et al. 2002a (20) UK 1992 - 1996 | Case-control study | Children Age < 15 years n = 5999 | Household average radon concentration through direct long term (6 to 7 months) radon measurement in the residence occupied at the time of diagnosis (for cases) or at the time of the study for controls. Mean radon concentration : 24.0 Bq/m3 | Age, sex, study region, and deprivation | Acute lymphoblastic leukaemia incidence | 805/NA | OR (95%CI) across household average radon concentration categories 0 - 24 : 1.00 (reference) 25 - 49 : 0.80 (0.64 – 0.99) 50 - 99 : 1.06 (0.79 – 1.44) 100 - 199 : 0.57 (0.29 – 1.12) 200+ : 0.81 (0.28 – 2.36) | 7 | Moderate |
|  |  |  |  |  | Non-Hodgkin Lymphoma incidence | 166/NA | OR (95%CI) across household average radon concentration categories 0 - 24 : 1.00 (reference) 25 - 49 : 0.68 (0.43 – 1.10) 50 - 99 : 0.92 (0.48 – 1.73) 100 - 199 : 0.74 (0.23 – 2.39) 200+ : 1.57 (0.36 – 6.92) |  |  |
|  |  |  |  |  | Hodgkin lymphoma incidence | 72/NA | OR (95%CI) across household average radon concentration categories 0 - 24 : 1.00 (reference) 25 - 49 : 0.89 (0.46 – 1.73) 50 - 99 : 1.00 (0.39 – 2.57) |  |  |
|  |  |  |  |  | Central nervous system tumors incidence | 404/NA | OR (95%CI) across household average radon concentration categories 0 - 24 : 1.00 (reference) 25 - 49 : 1.06 (0.77 – 1.32) 50 - 99 : 0.82 (0.52 – 1.29) 100 - 199 : 0.59 (0.24 – 1.46) 200+ : 1.13 (0.34 – 3.78) |  |  |
| McLaughlin et al. 1993 (21) Canada 1950 - 1988 | Case-control study | Children Age < 15 years n = 1002 | Paternal occupational radon exposure before conception | Age, period of time of diagnosis, region of the province matched, Adjusted for maternal age, birth weight, sex, and distance to the nearest nuclear facility | Leukaemia incidence | 112/890 | OR (95%CI) for paternal exposure to radon (internal dose to lung) ≥ 0.1 compared to 0.0 WLM : 2.54 (0.33 - 19.4) OR (95%CI) across paternal internal radon dose to lung categories (WLM)  0.0 : 1.0 (reference) 0.1 - 49 : 1.89 (0.21 - 17.3) ≥ 50 : 5.14 (0.48 - 55.2) p_trend : 0.18 | 8 | High |
| Lucie et al. 1990 (22) UK _ | Ecological study | Children Age < 15 years n = 22 counties | County-level indoor radon concentration | Age and sex matched | Acute Lymphoblastic Leukaemia incidence | NA | Correlation between county-level radon exposure and acute lymphoblastic leukaemia : r = 0.46 ; p < 0.01 | _ | Insufficient information to assess the quality since we had access only to the abstract |
| Henshaw et al. 1990 (23) Canada & Danemark & Finland & France & Germany & Ireland & Italy & Japan & Norway & Netherlands & Poland & Switzerland & Sweden & UK & USA _ | Pooled ecological study | Children n = 13 countries | Country/region-level radon exposure | _ | Leukaemia incidence | NA | Correlation with country/region-level average radon exposure : r = 0.61(0.09 - 0.87) ; p < 0.02 | _ | Moderate |
|  |  |  |  |  | Brain and spinal cancer incidence |  | Correlation with country/region-level average radon exposure : r = 0.62 ; p < 0.02 |  |  |
|  |  |  |  |  | Osteosarcoma incidence |  | Correlation with country/region-level average radon exposure : r = 0.56 ; p < 0.05 |  |  |
|  |  |  |  |  | Melanoma incidence |  | Correlation with country/region-level average radon exposure : r = 0.56 ; p < 0.05 |  |  |
|  |  |  |  |  | Wilm's tumour incidence |  | Correlation with country/region-level average radon exposure : r = 0.47 ; p = 0.1 |  |  |
|  |  |  |  |  | Soft tissue sarcomas incidence |  | Correlation with country/region-level average radon exposure : r = 0.46 ; p = 0.1 |  |  |
|  |  |  |  |  | Neuroblastoma incidence |  | Correlation with country/region-level average radon exposure : 0.43 ; p >0.05 |  |  |
| Muirhead et al. 1991 (24) UK 1969 - 1983 | Ecological study | Children Age < 15 years at diagnosis n = 459 county districs | District-level average radon exposure | _ | Leukaemia + Non-Hodgkin lymphoma incidence | NA | Positive, non-significant regression coef ; p > 0.05 | _ | Moderate |
| Boice et al. 2007 (25) USA 1950 - 2000 | Ecological study | Children living near the uranium and Vanadium mining and milling operations in Montrose county, Colorado Age <20 years at diagnosis | _ | Race, sex, calendar year | All leukaemia mortality | 5 | RR, relative risk (95%CI) which represente SMR in study population (Montrose county) and SMR of the 5 comparison counties. The reference population is the colorado general population : 0.50 (0.20–1.24) | _ | Moderate |
| Boice et al. 2010 (26) USA 1982 - 2004 | Ecological study | Children living near the uranium milling and mining operations in Grants, New Mexico Age ≤ 19 years at diagnosis n = 1 county | _ | Age, sex, race | Leukaemia incidence | 15 | SIR : 1.63 (0.91 - 2.69) | _ | Moderate |
| Boice et al. 2010 (26) USA 1950 - 2004 | Ecological study | Children living near the uranium milling and mining operations in Grants, New Mexico Age ≤ 19 years at diagnosis n = 2 counties | _ | Age, sex, race | Leukaemia mortality | 26 | SMR : 1.06 (0.69 - 1.55) | _ | Moderate |
| Ha et al. 2017 (27) Korea 1999 - 2008 | Ecological study | Children Age < 20 years at diagnosis n = 234 regions | Model-based county-level average radon exposure | Smoking rate, regional deprivation index; | Non-Hodgkin lymphoma incidence | In males : 2843 In females : 2051 | RR, relative risk (95%CrI) per 10 Bq/m3 increase in radon exposure In males : 0.97 (0.93 - 1.02) In females : 1.07 (1.01 - 1.13) | _ | Moderate |
|  |  |  |  |  | Leukaemia incidence | Males : 1044 Females : 523 | RR, relative risk (95%CrI) per 10 Bq/m3 increase in radon exposure In males : 1.00 (0.92 - 1.08) In females : 0.98 (0.88 - 1.08) |  |  |
| Boz et al. 2022 (28) Switzerland 2001 - 2015 (13.6) | Cohort study | Adults in the GP Mean age : 49.1 years n = 4,904,443 | Model-based individual residential geolocation radon concentration Mean (SD): 75.9 (32.0) Range: 25.6–1154.1 | Age, UV, calendar time, sex, marital status, mother tongue, education level, socio-economic index, occupational environment, interaction term between age and radon exposure (centred age * exposure) | Malignant melanoma | 3979 | HRage* (95%IC) per 100 Bq/m3 age 30: 1.24 (0.95 - 1.60)  age 45: 1.17 (0.98 - 1.39)  age 60: 1.10 (0.99 - 1.23)  age 75: 1.04 (0.94 - 1.16) | 7 | Moderate |
|  |  |  |  |  | Non-melanoma skin cancer | 1118 | HR at age 30 (95%IC) per 100 Bq/m3 : 1.06 (0.75 - 1.49) |  |  |
|  |  |  |  |  | Skin cancer | 5097 | HR at age 60 (95%IC) per 100 Bq/m3 : 1.09 (0.99 - 1.21) |  |  |
| Teras et al. 2016 (29) USA 1992 - 2011 | Cohort study | Adults in the GP Mean age in 1992 : 63.0 n = 140,652 | Model-based county-level radon exposure of the corresponding primary residential zip code in 1982 (baseline of the source cohort).  Median : 45.9 Bq/m3 range : 6.3 - 265.7 Bq/m3 | Age, state of residence, race, education, family history of hematologic cancer, smoking status (never, former, current, other/ missing), alcohol use, BMI, pesticide exposure, industrial exposures, water supply type, Additional covariates in female models: postmenopausal estrogen use, postmenopausal estrogen and progesterone use. | Lymphohematological cancer incidence | 3019 | HR (95%IC) per 100 Bq/m3 for all lymphohematological cancer For the whole study population : 1.14 (1.00–1.29) For females : 1.38 (1.15–1.65) For males 0.96 (0.80–1.16) HRs across radon exposure categories indicate no statistically significant association For males, but a significant increased risk was found In females at radon exposure over 148 Bq/m3 | 8 | Moderate |
|  |  |  |  |  | Hodgkin lymphoma incidence | 59 | HR (95%IC) per 100 Bq/m3 increase in mean county-level residential radon exposure For females : 0.82 (0.27-2.49) For males : 0.40 (0.06-2.94) HRs across radon exposure categories indicate no statistically significant association for both male and female |  |  |
|  |  |  |  |  | Follicular lymphoma incidence | 287 | HR (95%IC) per 100 Bq/m3 increase in mean county-level residential radon exposure For females : 1.70 (0.98-2.94) For males : 1.15 (0.64-2.06) HRs across radon exposure categories indicate no statistically significant association For males, but a significant increased risk was found In females at radon exposure over 148 Bq/m3 |  |  |
|  |  |  |  |  | Diffuse large basal-cell lymphoma incidence | 454 | HR (95%IC) per 100 Bq/m3 increase in mean county-level residential radon exposure For females : 1.54 (0.98-2.43) For males : 1.09 (0.66-1.81) HRs across radon exposure categories indicate no statistically significant association for both male and female |  |  |
|  |  |  |  |  | T-cell incidence | 119 | HR (95%IC) per 100 Bq/m3 increase in mean county-level residential radon exposure For females : 2.10 (0.89-5.00) For males : 1.79 (0.72-4.46) HRs across radon exposure categories indicate no statistically significant association for both male and female |  |  |
|  |  |  |  |  | Marginal zone incidence | 95 | HR (95%IC) per 100 Bq/m3 increase in mean county-level residential radon exposure For females : 1.56 (0.67-3.65) For males : 0.34 (0.08-1.44) HRs across radon exposure categories indicate no statistically significant association for both male and female |  |  |
|  |  |  |  |  | Chronic lymphoblastic leukaemia/Small lymphocytic lymphoma incidence | 525 | HR (95%IC) per 100 Bq/m3 increase in mean county-level residential radon exposure For females : 1.22 (0.76-1.96) For males : 0.91 (0.59-1.39) HRs across radon exposure categories indicate no statistically significant association for both male and female |  |  |
|  |  |  |  |  | Myeloid leukaemia incidence | 332 | HR (95%IC) per 100 Bq/m3 increase in mean county-level residential radon exposure For females : 0.86 (0.45-1.63) For males : 0.99 (0.57-1.71) HRs across radon exposure categories indicate no statistically significant association for both male and female |  |  |
|  |  |  |  |  | Multiple myeloma incidence | 435 | HR (95%IC) per 100 Bq/m3 increase in mean county-level residential radon exposure For females : 1.56 (1.00-2.44) For males : 1.04 (0.64-1.69) HRs across radon exposure categories indicate no statistically significant association for both males and females |  |  |
| Ruano-Ravina et al. 2017 (30) Spain 1999 - 2008 | Ecological study | n = 251 municipalities | Average municipality-level radon concentration was based on radon measurements obtained from the Galician Radon Map and from controls belonging to two previous case-controls studies on residential radon and lung cancer | Age group, sex, five-years period durin the estimations of relative risks | Brain cancer mortality | In males : 949 In females : 758 | Spearman’s Rho (p-value) between radon and brain cancer relative risk For males : 0.164 (p-value <0.009) For females : 0.433 (p-value <0.001) | _ | Moderate |
| López-Abente et al. 2018 (31) Spain 1999 - 2008 | Ecological study | Adults in the GP n = 313 municipalities | Average municipality-level radon concentration was based on radon measurements obtained from the Galician Radon Map and from controls belonging to two previous case-controls studies on residential radon and lung cancer Median radon (min - max) in Bq/m3 : 153.9 (6.0 - 2756.0) | Arsenic, altitude (the centroid altitude of each town), sociodemographic and environmental indicators (population size,  percentages of illiteracy, farmers, and unemployment, average number of persons per household, and mean income) | Stomach cancer mortality | Stomach cancer : In male = 3286 In female = 5637 | Relative risks (95%CrI) for a two twofold increase in radon exposure suggest no significant association found In males. Significant associations were found for only stomach and brain cancers In females. Stomach cancer : 1.17 (1.02 - 1.32)  Brain cancer : 1.28 (1.13 - 1.50) No association was found for Buccal cavity and pharynx, Oesophagus, Pancreas, Colorectal, Prostate (In males), Breast (In females), Bladder, Kidney, Thyroid, Non-Hodgkin lymphoma, Leukaemia in both males and females | _ | Moderate |
|  |  |  |  |  | Buccal and pharyngeal cancer mortality | 1800 | Relative risks (95%CrI) for a two twofold increase in radon exposure In males : 1.039 (0.914 - 1.181) In females : 1.068 (0.857- 1.314) |  |  |
|  |  |  |  |  | Oesophagus cancer mortality | 1701 | Relative risks (95%CrI) for a two twofold increase in radon exposure In males : 0.984 (0.890 - 1.091) In females : 1.059 (0.828 - 1.353) |  |  |
|  |  |  |  |  | Stomach cancer mortality | 5637 | Relative risks (95%CrI) for a two twofold increase in radon exposure In males : 1.084 (0.977 - 1.206) In females : 1.174 (1.022 - 1.325) |  |  |
|  |  |  |  |  | Pancreas cancer mortality | 3831 | Relative risks (95%CrI) for a two twofold increase in radon exposure In males : 1.009 (0.919 - 1.095) In females : 0.999 (0.889 - 1.119) |  |  |
|  |  |  |  |  | Colorectal cancer mortality | 10,129 | Relative risks (95%CrI) for a two twofold increase in radon exposure In males : 0.993 (0.935 - 1.060) In females : 0.991 (0.932 - 1.056) |  |  |
|  |  |  |  |  | Prostate cancer mortality | 5176 | Relative risks (95%CrI) for a two twofold increase in radon exposure : 0.940 (0.864 - 1.009) |  |  |
|  |  |  |  |  | Breast cancer mortality | 4001 | Relative risks (95%CrI) for a two twofold increase in radon exposure : 0.963 (0.871 - 1.034) |  |  |
|  |  |  |  |  | Bladder cancer mortality | 3041 | Relative risks (95%CrI) for a two twofold increase in radon exposure In males : 0.946 (0.875 - 1.053) In females : 1.130 (0.969 - 1.323) |  |  |
|  |  |  |  |  | Kidney cancer mortality | 1272 | Relative risks (95%CrI) for a two twofold increase in radon exposure In males : 1.122 (0.980 - 1.279) In females : 1.113 (0.916 - 1.375) |  |  |
|  |  |  |  |  | Brain cancer mortality | 1817 | Relative risks (95%CrI) for a two twofold increase in radon exposure In males : 1.045 (0.930 - 1.175) In females : 1.283 (1.128 - 1.498) |  |  |
|  |  |  |  |  | Thyroid cancer mortality | 295 | Relative risks (95%CrI) for a two twofold increase in radon exposure In males : 1.046 (0.721 - 1.529) In females : 0.95 (0.733 - 1.240) |  |  |
|  |  |  |  |  | Non-Hodgkin lymphoma mortality | 2245 | Relative risks (95%CrI) for a two twofold increase in radon exposure In males : 1.036 (0.927 - 1.160) In females : 1.066 (0.868 - 1.211) |  |  |
|  |  |  |  |  | Leukeamia mortality | 2105 | Relative risks (95%CrI) for a two twofold increase in radon exposure In males : 0.951 (0.856 - 1.069) In females : 1.054 (0.933 - 1.191) |  |  |
| Ruano-Ravina et al. 2014 (32) Spain 1989 - 1998 | Ecological study | Adults in the GP n = 129 municipalities | Average municipal-level radon concentration was based on radon measurements obtained from the Galician Radon Map Median (IQR) in Bq/m3 : 83 (52–124) | Age, sex, calendar period, municipalities | Esophageal cancer mortality | In males : 1169 In females : 217 | Correlation and p-value between average radon and municipality relative risk in males : r = 0.298; p < 0.001  in females : r = 0.045; p = 0.615 | _ | Moderate |
| Nilles et al. 2022 (33) USA 1975 - 2016 | Ecological study | Adults in the GP n = All the 99 counties in the IOWA state (3,016,267 inhabitants) | County-level average radon concentration in pCi/L (based on radon measurment data from 2010 to 2016). Median (IQR) in Bq/m3 : 83 (52–124) | Age, sex | Bone cancer incidence | _ | Correlation coefficient between the average county-level radon exposure and bone cancer age-adjusted incidence rates : r =-0.038 ; P<0.7) | _ | Moderate |
| Messier et al. 2017 (34) USA 1999 - 2009 | Ecological study | Adults in the GP n = 1554 census tracts Mean age (SD) In cases : 34.51 (8.53) In controls : 40.14 (12.45) | Census tract-level average (geometric mean) groundwater radon exposure derived from model-based address-level groundwated radon estimates | Gender, age, indoor air radon, smoking prevalence of the census tract, residential tenure, percent public water, percent white race, and percent black race | Stomach cancer incidence | 5,218 | IRR (95%CI) per 100 Bq/L increase in census tract-level groundwater radon exposure : 1.05 (0.99 - 1.11) | _ | High |
| Messier et al. 2017 (34) USA 1999 - 2009 | Case-only study | Adults in the GP n = 5,218 Mean age (SD) In cases : 34.51 (8.53) In controls : 40.14 (12.45) | Model-based home addrress-level groundwater radon exposure | Age, sex, indoor air radon, smoking, residential tenure, public water use, race | Cluster membership of stomach cancer | 667 cases within 113 clusters (census tracts with higher than the expected stomach cancer) | OR (95%CI) per 100 Bq/L increase in home address-level groundwater radon exposure : 1.24 (1.03 - 1.49) | 7 | Moderate |
| Goyal et al. 2015 (35) USA 1991 - 2009 | Ecological study | Adults in the GP n = counties in Pennsylvania | County-level radon exposure (in pCi/L) | _ | Thyroid cancer incidence | 1340 | Poisson regression beta :  -.00526 ; p = .1013 Negative binomial regression beta : -0.00416 ; p = 0.2336 | _ | Moderate |
| Wheeler et al. 2013 (36) UK 2006 - 2008 | Ecological study | Adults in the GP n = 311 local authorities | Local authority-level radon exposure | Bright sunshine hours, arsenic, income depravation score, employment depravation score, education depravation score | Non- melanoma skin cancer incidence | 216,497 | Linear regression coefficiant β (95%CI) across Local authority-level average radon exposure categories (Bq/m3) 0–39 : 1.00 (reference) 40–44 : 2.08 ( -8.56 - 12.71)  45–49 : -1.94 ( -14.02 - 10.13) 50–59 : 3.08 ( -8.23 - 14.39)  60–74 : 1.37 (-11.50 - 14.23) 75–99 : 9.54 (-4.08 - 23.16) ≥ 100 : 12.47 (-3.90 - 28.84) | _ | Moderate |
| Auvinen et al. 2002 (37) Finland 1981 - 1995 | Case-cohort study | Adults in the GP n = 309 | Radon concentration in the well water samples at 1996 Median (IQR) in Bq/l for cases : 80 (30–320) for the subcohort : 130 (30 - 240) | Age, sex | Leukaemia incidence | 35 | HR (95%CI) across radon concentration categories in Bq/l <300 : 1.00 (reference) 300–999 : 0.91 (0.36–2.31) 1000–15,000 : 0.73 (0.16–3.28) | 7 | Moderate |
| Auvinen et al. 2005 (38) Finland 1981 - 1995 | Case-cohort study | Adults in the GP n = 362 | Radon concentration in the well water samples at 1996 Median (IQR) in Bq/l : for cases : 130 (30 - 240) for the subcohort : 130 (40 - 340) | Age, sex | Stomach cancer incidence | 88 | HR (95%CI) across radon concentration categories in Bq/l <130 : 1.00 (reference) 130–299 : 0.54 (0.25–1.18) 300–15,000 : 0.48 (0.25–0.94) | 7 | Moderate |
| Kurttio et al. 2006 (39) Finland 1981 - 1995 | Case-cohort study | Adults in the GP n = 386 | Radon concentration in the well water samples at 1996 Median (IQR) in Bq/l : for bladder cancer cases : 170 (34 - 550) for kidney cancer cases : 140 (49 - 350) for the subcohort : 130 (39 - 340) | Age, gender, smoking (never, ex-smoker (smoker prior to 1980 and quit), current smoking (smoker after 1979), or missing), BMI (BMI is for kidney cancer only) in sensitivity analysis owner missing data | Bladder cancer incidence | 61 | HR (95%CI) across radon concentration categories in Bq/l <130 : 1.00 (reference) 130–399 : 0.67 (0.31–1.44) 400–19,000 : 1.34 (0.66–2.72) | 7 | Moderate |
|  |  |  |  |  | Kidney cancer incidence | 51 | HR (95%CI) across radon concentration categories in Bq/l <130 : 1.00 (reference) 130–399 : 0.64 (0.30–1.38) 300–19,000 : 0.70 (0.29–1.67) |  |  |
| Forestiere et al. 1992 (40) Italy 1980 - 1986 | Case-control study | Adults in the GP n = 2041 (only males) Age at inclusion : 30 - 80 years | Radon map exposure (geological features) categories define as low, intermediate, high | Age, farming, urbanisation | Myeloid mortality | 20/462 | OR (90% CI) across radon exposure categories) Low : 1.0 (reference) Intermediate : 2.5 (0.80 - 7.9) High : 2.3 (0.9 - 6.1) p-trend : 0.16 | 6 | Moderate |
|  |  |  |  |  | Melanoma mortality | 14/462 | OR (90% CI) across radon exposure categories) Low : 1.0 (reference) Intermediate : 2.0 (0.46 - 8.4) High : 2.8 (0.92 - 8.2) p-trend : 0.13 |  |  |
|  |  |  |  |  | Kidney mortality | 41/462 | OR (90% CI) across radon exposure categories) Low : 1.0 (reference) Intermediate : 0.8 (0.33 - 1.9) High : 2.2 (1.17 - 4.3) p-trend : 0.04 |  |  |
|  |  |  |  |  | Autres cancer mortality | Esophagus : 44 Stomach : 307 Colon-rectum : 171 Pancreas : 66 Larynx : 71 Melanoma : 14 Non-melanoma skin cancer : 7 Prostate : 118 Bladder : 79 Kidney : 41 Brain : 43 Lymphohematological: 110 | OR (90% CI) across radon exposure categories indicate no statistically significant trend for mortality from oesophagus, stomach, colon-rectum, pancreas, larynx, non-melanoma skin cancer, prostate, bladder, brain, and lymphohematological cancers |  |  |
| Forestiere et al. 1998 (41) Italy 1980 - 1989 | Case-control study | Adults in the GP n = 255 (only males) Age at inclusion : 30 - 80 years | Direct radon measurement in individual home. | Age matched | Myeloid leukaemia mortality | 44 | OR (95%CI) across radon exposure quartiles in Bq/m3 ≤ 99 : 1.00 (reference) 100 - 145 : 0.51 (0.2 - 1.3) 146 - 230 : 0.66 (0.3 - 1.6) > 231 : 0.56 (0.2 - 1.4) | 7 | Moderate |
| Puskin et al. 2003 (42) USA 1970 - 1994 | Ecological study | Adults in the GP n = 1585 counties | Cohen's average county-level radon exposure | coounty-specific smoking prevelence ; sex | Several cancers' mortality | _ | Significant negative correlation with oral and pharynx , larynx, esophagus, nasopharynx, bladder, pancreas cancers. Significant positive correlation with prostate cancer. No correlation with breast cancer. No correlation with colon cancer in males, but positive correlation in females. | _ | Moderate |
| Boice et al. 2010 (26) USA 1950 - 2004 | Ecological study | Adults in the GP n = 1 county (county of Cibola in New Mexico state, 23,794 inhabitants) | _ | Age, sex, race | Esophagus cancer mortality | 75 | SMR (95%CI) : 1.23 (0.97 - 1.54) | _ | Moderate |
|  |  |  |  |  | Stomach cancer mortality | 208 | SMR (95%CI) : 1.21 (1.05 - 1.39) ; p < 0.05 |  |  |
|  |  |  |  |  | Colorectal cancer mortality | 359 | SMR (95%CI) : 1.00 (0.90 - 1.11) |  |  |
|  |  |  |  |  | Pancreas cancer mortality | 221 | SMR (95%CI) : 1.10 (0.96 - 1.25) |  |  |
|  |  |  |  |  | Malignant melanoma mortality | 38 | SMR (95%CI) : 0.78 (0.55 - 1.06) |  |  |
|  |  |  |  |  | Breast cancer mortality | 249 | SMR (95%CI) :0.88 (0.78 - 1.00) ; p > 0.05 |  |  |
|  |  |  |  |  | Cervix Uteri cancer mortality | 47 | SMR (95%CI) : 0.85 (0.63 - 1.13) |  |  |
|  |  |  |  |  | Uterus - corpus, other and NOS cancer mortality | 41 | SMR (95%CI) : 0.90 (0.65 - 1.22) |  |  |
|  |  |  |  |  | Ovary cancer mortality | 81 | SMR (95%CI) : 0.89 (0.79 - 1.10) |  |  |
|  |  |  |  |  | Prostate cancer mortality | 211 | SMR (95%CI) : 0.92 (0.80 - 1.05) |  |  |
|  |  |  |  |  | Bladder cancer mortality | 67 | SMR (95%CI) : 0.97 (0.75 - 1.23) |  |  |
|  |  |  |  |  | Kidney cancer mortality | 93 | SMR (95%CI) : 1.08 (0.87 - 1.32) |  |  |
|  |  |  |  |  | Primary liver cancer mortaity | 95 | SMR (95%CI) : 1.15 (0.93 - 1.40) |  |  |
|  |  |  |  |  | Bone cancer mortality | 18 | SMR (95%CI) : 1.17 (0.69 - 1.85) |  |  |
|  |  |  |  |  | Connective tissue cancer mortality | 28 | SMR (95%CI) : 1.18 (0.72 - 1.57) |  |  |
|  |  |  |  |  | Brain and central nervous system cancer mortality | 100 | SMR (95%CI) : 1.16 (0.95 - 1.41) |  |  |
|  |  |  |  |  | Thyroid cancer mortality | 14 | SMR (95%CI) : 1.24 (0.68 - 2.09) |  |  |
|  |  |  |  |  | Non-Hodgkin lymphoma mortality | 106 | SMR (95%CI) : 0.88 (0.72 - 1.07) |  |  |
|  |  |  |  |  | Hodgkin lymphoma mortality | 15 | SMR (95%CI) : 0.72 (0.40 - 1.19) |  |  |
|  |  |  |  |  | Multiple Myeloma mortality | 67 | SMR (95%CI) : 1.05 (0.81 - 1.34) |  |  |
|  |  |  |  |  | All leukaemia mortality | 176 | SMR (95%CI) : 1.12 (0.96 - 1.30) |  |  |
|  |  |  |  |  | Chronic lymphoblastic leukaemia mortality | 23 | SMR (95%CI) : 1.08 (0.68 - 1.62) |  |  |
|  |  |  |  |  | Non-CLL mortality | 153 | SMR (95%CI) : 1.13 (0.95 - 1.32) |  |  |
| Boice et al. 2010 (26) USA 1950 - 2004 | Ecological study | Adults in the GP n = 1 county (county of Cibola in New Mexico state, 23,794 inhabitants) | _ | Age, sex, race | Esophagus cancer incidence | 15 | SIR (95%CI) : 1.14 (0.64 - 1.88) | _ | Moderate |
|  |  |  |  |  | Stomach cancer incidence | 47 | SIR (95%CI) : 1.12 (0.82 - 1.49) |  |  |
|  |  |  |  |  | Colorectal cancer incidence | 152 | SIR (95%CI) : 0.95 (0.80 - 1.11) |  |  |
|  |  |  |  |  | Pancreas cancer incidence | 41 | SIR (95%CI) : 0.99 (0.71 - 1.34) |  |  |
|  |  |  |  |  | Malignant melanoma | 35 | SIR (95%CI) : 0.66 (0.46 - 0.92) ;  p < 0.05 |  |  |
|  |  |  |  |  | Breast cancer incidence | 190 | SIR (95%CI) : 0.85 (0.73 - 0.98) ;  p < 0.05 |  |  |
|  |  |  |  |  | Cervix Uteri cancer incidence | 19 | SIR (95%CI) : 0.67 (0.41 - 1.05) |  |  |
|  |  |  |  |  | Uterus - corpus, other and NOS cancer incidence | 39 | SIR (95%CI) : 0.98 (0.70 - 1.34) |  |  |
|  |  |  |  |  | Ovary cancer incidence | 29 | SIR (95%CI) : 0.90 (0.61 - 1.30) |  |  |
|  |  |  |  |  | Prostate cancer incidence | 252 | SIR (95%CI) : 1.00 (0.88 - 1.13) |  |  |
|  |  |  |  |  | Bladder cancer incidence | 52 | SIR (95%CI) : 0.95 (0.71 - 1.24) |  |  |
|  |  |  |  |  | Kidney cancer incidence | 57 | SIR (95%CI) : 1.11 (0.84 - 1.44) |  |  |
|  |  |  |  |  | Liver cancer mortaity | 21 | SIR (95%CI) : 0.73 (0.45 - 1.12) |  |  |
|  |  |  |  |  | Bone cancer incidence | 3 | SIR (95%CI) : 0.65 (0.13 - 1.89) |  |  |
|  |  |  |  |  | Soft tissue including heart cancer incidence | 9 | SIR (95%CI) : 0.73 (0.33 - 1.38) |  |  |
|  |  |  |  |  | Brain and central nervous system cancer incidence | 24 | SIR (95%CI) : 1.07 (0.69 - 1.60) |  |  |
|  |  |  |  |  | Thyroid cancer incidence | 31 | SIR (95%CI) : 0.88 (0.60 - 1.25) |  |  |
|  |  |  |  |  | Non-Hodgkin lymphoma incidence | 56 | SIR (95%CI) : 1.01 (0.76 - 1.31) |  |  |
|  |  |  |  |  | Hodgkin lymphoma incidence | 7 | SIR (95%CI) : 0.73 (0.29 - 1.50) |  |  |
|  |  |  |  |  | Multiple Myeloma incidence | 13 | SIR (95%CI) : 0.61 (0.33 - 1.05) |  |  |
|  |  |  |  |  | All leukaemia incidence | 62 | SIR (95%CI) : 1.26 (0.96 - 1.61) |  |  |
|  |  |  |  |  | Chronic lymphoblastic leukaemia incidence | 16 | SIR (95%CI) : 1.17 (0.67 - 1.90) |  |  |
|  |  |  |  |  | Non-CLL incidence | 46 | SIR (95%CI) : 1.29 (0.95 - 1.72) |  |  |
| Boice et al. 2007 (43) USA 1979 - 2004 | Cohort study | Adults in the GP n = 1905 | Exposure period : 1936 - 2004 | Age, sex, race, calendar-year | Buccal cavity and pharynx cancer mortality | 1 | SMR (95%CI) : 0.92 (0.19 - 2.68) | 8 | Moderate |
|  |  |  |  |  | Esophagus cancer mortality | 1 | SMR (95%CI) : 0.23 (0.01 - 1.30) |  |  |
|  |  |  |  |  | Stomach cancer mortality | 6 | SMR (95%CI) : 1.21 (0.44 - 2.63) |  |  |
|  |  |  |  |  | Colon cancer mortality | 6 | SMR (95%CI) : 0.33 (0.12 - 0.73) |  |  |
|  |  |  |  |  | Rectal cancer mortality | 6 | SMR (95%CI) : 0.96 (0.20 - 2.81) |  |  |
|  |  |  |  |  | Pancreas cancer mortality | 10 | SMR (95%CI) : 1.02 (0.49 - 1.87) |  |  |
|  |  |  |  |  | Malignant melanoma | 2 | SMR (95%CI) : 0.74 (0.09 - 2.66) |  |  |
|  |  |  |  |  | Non-melanoma skin cancer | 1 | SMR (95%CI) : 1.10 (0.03 - 6.11) |  |  |
|  |  |  |  |  | Breast cancer mortality | 17 | SMR (95%CI) : 1.57 (0.92 - 2.52) |  |  |
|  |  |  |  |  | All uterine cancer mortality | 2 | SMR (95%CI) : 0.82 (0.10 - 2.98) |  |  |
|  |  |  |  |  | Other female genitalorgans cancer mortality | 4 | SMR (95%CI) : 1.05 (0.29 - 2.68) |  |  |
|  |  |  |  |  | Prostate cancer mortality | 14 | SMR (95%CI) : 0.91 (0.50 - 1.53) |  |  |
|  |  |  |  |  | Bladder and other urinary organs cancer mortality | 3 | SMR (95%CI) : 0.59 (0.12 - 1.73) |  |  |
|  |  |  |  |  | Kidney cancer mortality | 4 | SMR (95%CI) : 0.91 (0.25 - 2.33) |  |  |
|  |  |  |  |  | Liver and gallbladder cancer mortaity | 2 | SMR (95%CI) : 0.42 (0.05 - 1.51) |  |  |
|  |  |  |  |  | Bone cancer mortality | 0 | SMR (95%CI) : 0.00 (0.00 - 11.5) |  |  |
|  |  |  |  |  | Brain and central nervous system cancer mortality | 0 | SMR (95%CI) : 0.00 (0.00 - 0.85) |  |  |
|  |  |  |  |  | Thyroid and other endocrine gland cancer mortality | 1 | SMR (95%CI) : 1.79 (0.05 - 9.95) |  |  |
|  |  |  |  |  | All lymphohematological cancer mortality | 15 | SMR (95%CI) : 0.80 (0.45 - 1.33) |  |  |
|  |  |  |  |  | Non-Hodgkin lymphoma mortality | 6 | SMR (95%CI) : 0.80 (0.30 - 1.75) |  |  |
|  |  |  |  |  | Hodgkin lymphoma mortality | 1 | SMR (95%CI) : 1.98 (0.05 - 11.0) |  |  |
|  |  |  |  |  | Multiple Myeloma mortality | 4 | SMR (95%CI) : 1.18 (0.32 - 3.01) |  |  |
|  |  |  |  |  | All leukaemia mortality | 4 | SMR (95%CI) : 0.56 (0.15 - 1.43) |  |  |
|  |  |  |  |  | Chronic lymphoblastic leukaemia mortality | 2 | SMR (95%CI) : 1.26 (0.15 - 4.56) |  |  |
|  |  |  |  |  | Non-CLL leukaemia mortality | 2 | SMR (95%CI) : 0.36 (0.04 - 1.30) |  |  |
| Boice et al. 2007 (25) USA 1950 - 2000 | Ecological study | Adults in the GP n = 1 study county and 5 control counties in Colorado | _ | Age, sex, race, calendar-year | Esophagus cancer mortality | 22 | RR, relative risk (95%CI) which represente the ratio of SMR for the study population (Montrose county) and that for the 5 comparison counties. The reference population was the colorado general population. RR (95%CI) : 0.70 (0.45–1.09) | _ | Moderate |
|  |  |  |  |  | Stomach cancer mortality | 87 | RR (95%CI) : 1.15 (0.92–1.45) |  |  |
|  |  |  |  |  | Colorectal cancer mortality | 207 | RR (95%CI) : 0.95 (0.82–1.10) |  |  |
|  |  |  |  |  | Pancreas cancer mortality | 121 | RR (95%CI) : 1.08 (0.98–1.19) |  |  |
|  |  |  |  |  | Malignant melanoma mortality | 25 | RR (95%CI) : 0.90 (0.59–1.38) ) |  |  |
|  |  |  |  |  | Skin cancer mortality | 37 | RR (95%CI) : 1.06 (0.75–1.50) |  |  |
|  |  |  |  |  | Breast cancer mortality | 126 | RR (95%CI) : 0.86 (0.71–1.03) |  |  |
|  |  |  |  |  | Cervix uteri cancer mortality | 15 | RR (95%CI) : 0.73 (0.43–1.24) |  |  |
|  |  |  |  |  | Corpus uteri cancer mortality | 34 | RR (95%CI) : 1.35 (0.94–1.96) |  |  |
|  |  |  |  |  | Ovary cancer mortality | 49 | RR (95%CI) : 1.35 (0.94–1.96) |  |  |
|  |  |  |  |  | Prostate cancer mortality | 148 | RR (95%CI) : 1.07 (0.90–1.28) |  |  |
|  |  |  |  |  | Bladder cancer mortality | 44 | RR (95%CI) : 1.01 (0.74–1.39) |  |  |
|  |  |  |  |  | Kidney, ureter, and other urinary organs cancer mortality | 34 | RR (95%CI) : 0.80 (0.56–1.14) |  |  |
|  |  |  |  |  | Liver and kidney cancer mortaity | 88 | RR (95%CI) : 0.92 (0.74–1.15) |  |  |
|  |  |  |  |  | Bone cancer mortality | 8 | RR (95%CI) : 1.36 (0.63–2.91) |  |  |
|  |  |  |  |  | Connective and other soft tissue | 12 | RR (95%CI) : 1.30 (0.70–2.42) |  |  |
|  |  |  |  |  | Brain and central nervous system cancer mortality | 44 | RR (95%CI) : 0.93 (0.68–1.28) |  |  |
|  |  |  |  |  | Thyroid gland cancer mortality | 5 | RR (95%CI) : 0.82 (0.32–2.07) |  |  |
|  |  |  |  |  | All lymphohematological cancer mortality | 15 | RR (95%CI) : 0.80 (0.45 - 1.33) |  |  |
|  |  |  |  |  | Non-Hodgkin lymphoma mortality | 75 | RR (95%CI) : 1.05 (0.82–1.34) |  |  |
|  |  |  |  |  | Hodgkin lymphoma mortality | 15 | RR (95%CI) : 1.72 (0.97–3.04) |  |  |
|  |  |  |  |  | Multiple Myeloma mortality | 33 | RR (95%CI) : 0.97 (0.67–1.39) |  |  |
|  |  |  |  |  | All leukaemia mortality | 65 | RR (95%CI) : 0.78 (0.60–1.01) |  |  |
|  |  |  |  |  | Chronic lymphoblastic leukaemia mortality | 10 | RR (95%CI) : 0.71 (0.37–1.36) |  |  |
|  |  |  |  |  | Non-CLL leukaemia mortality | 55 | RR (95%CI) : 0.80 (0.61–1.06) |  |  |
| Boice et al. 2003 (44) USA 1950 - 2001 | Ecological study | Adults in the GP n = 1 study county with prior uranium mining and milling activities, and 4 control counties in Texas | _ | Age, sex, race | Esophagus cancer mortality | 20 | RR, relative risk (95%CI) which represente the ratio of SMR in the study population (Karnes county) with that in the 4 comparison counties. The reference population is the US general population. RR : 1.06 (0.6–1.8) | _ | Moderate |
|  |  |  |  |  | Stomach cancer mortality | 72 | RR (95%CI) : 1.08 (0.8–1.4) |  |  |
|  |  |  |  |  | Colorectal cancer mortality | 168 | RR (95%CI) : 1.17 (1.0–1.4) ; p < 0.05 |  |  |
|  |  |  |  |  | Pancreas cancer mortality | 69 | RR (95%CI) : 1.01 (0.8–1.3) |  |  |
|  |  |  |  |  | Malignant melanoma mortality | 21 | RR (95%CI) : 1.23 (0.7–2.0) |  |  |
|  |  |  |  |  | Female breast cancer mortality | 79 | RR (95%CI) : 1.01 (0.8–1.3) |  |  |
|  |  |  |  |  | Cervix uteri cancer mortality | 18 | RR (95%CI) : 0.76 (0.5–1.3) |  |  |
|  |  |  |  |  | Corpus uteri cancer mortality | 5 | RR (95%CI) : 0.72 (0.3-1.9) |  |  |
|  |  |  |  |  | Ovary cancer mortality | 28 | RR (95%CI) : 0.90 (0.6–1.4) |  |  |
|  |  |  |  |  | Prostate cancer mortality | 76 | RR (95%CI) : 0.95 (0.7–1.2) |  |  |
|  |  |  |  |  | Bladder cancer mortality | 17 | RR (95%CI) : 0.64 (0.4–1.1) |  |  |
|  |  |  |  |  | Kidney, ureter, and other urinary organs cancer mortality | 19 | RR (95%CI) : 0.58 (0.4–1.0) ; p < 0.05 |  |  |
|  |  |  |  |  | Liver cancer mortaity | 27 | RR (95%CI) : 0.81 (0.5–1.2) |  |  |
|  |  |  |  |  | Bone cancer mortality | 11 | RR (95%CI) : 1.35 (0.7–2.8) |  |  |
|  |  |  |  |  | Connective and other soft tissue | <4 | RR (95%CI) : 0.44 (0.1–1.5) |  |  |
|  |  |  |  |  | Brain and central nervous system cancer mortality | 24 | RR (95%CI) : 0.92 (0.6–1.4) |  |  |
|  |  |  |  |  | Thyroid gland cancer mortality | <4 | RR (95%CI) : 0.31 (0.1–1.3) |  |  |
|  |  |  |  |  | All lymphohematological cancer mortality | 15 | RR (95%CI) : 0.80 (0.45 - 1.33) |  |  |
|  |  |  |  |  | Non-Hodgkin lymphoma mortality | 38 | RR (95%CI) : 1.00 (0.7–1.4) |  |  |
|  |  |  |  |  | Hodgkin lymphoma mortality | 12 | RR (95%CI) : 1.79 (0.9–3.6) |  |  |
|  |  |  |  |  | Multiple Myeloma mortality | 22 | RR (95%CI) : 1.37 (0.8–2.3) |  |  |
|  |  |  |  |  | All leukaemia mortality | 59 | RR (95%CI) : 1.15 (0.9–1.6) |  |  |
|  |  |  |  |  |  |  |  |  |  |
| Smith et al. 2007 USA 1973 - 2002 | Ecological study | Adults in the GP n = counties in Iowa state | Model-based county-level average radon exposure | Race, calendar year periods, urban influence code | All leukaemia incidene | _ | RR, relative risk (95% ICr) per 1 pCi/L increase in county-level average radon : 1.04 (0.98 –1.10) | _ | High |
|  |  |  |  |  | Acute lymphoblastic leukaemia incidence | _ | RR (95%CIr): 0.91 (0.78 - 1.03) |  |  |
|  |  |  |  |  | Acute myeloid leukaemia incidence | _ | RR (95%CIr): 1.01 (0.92 - 1.12) |  |  |
|  |  |  |  |  | Chronic lymphoblastic leukaemia incidence | _ | RR (95%CIr) : 1.06 (0.96 - 1.16) |  |  |
|  |  |  |  |  | Chronic myeloid leukaemia incidence | _ | RR (95%CIr): 1.12 (0.98 - 1.27) |  |  |
| Ye et al. 1998 Japan 1976 - 1993 | Ecological study | Adults in the GP n = 1 study county and 3 control counties (4,331 inhabitants in the study county) Age ≥ 40 | Town-level average radon exposure based on 24-hour radon measurement in sampled homes | Age, sex, period | Stomach cancer mortality | 55 | SMR (95%CI) in males: 0.77 (0.52–1.10) in females : 1.05 (0.68–1.55) | _ | Moderate |
|  |  |  |  |  | Liver cancer mortality | 27 | SMR (95%CI) in males : 1.35 (0.62–2.56) in females : 0.93 (0.37–1.91) |  |  |
|  |  |  |  |  | Colorectum cancer mortality | 20 | SMR (95%CI) in males : -0.80 (0.38–1.47) in females : 0.86 (0.41–1.58) |  |  |
|  |  |  |  |  | Leukaemia mortality | 9 | SMR (95%CI) in males : 2.20 (0.71–5.13) in females : 2.26 (0.61–5.78) |  |  |
| Ye et al. 1998 Japan 1976 - 1993 | Cohort study (regarding RR estimation) and ecological study (regarding SIR estimation) | Adults in the GP n = 4 districts in elevated radon level area and 3 districts in control area in Misasa Town (4,331 inhabitants) Age ≥ 40 | _ | Age, sex, period, radon area (for RR analysis) | Oral cavity cancer incidence | 2 | SIR (95%CI) in males : 0.64 (0.07–2.30) | 6 | Moderate |
|  |  |  |  |  | Esophagus cancer incidence | 6 | SIR (95%CI) in males : 0.64 (0.07–2.30) in females : 0.95 (0.01–5.29) |  |  |
|  |  |  |  |  | Stomach cancer incidence | 79 | RR, relative risk (95%CI) of stomach cancer incidence in elevated radon level area compared to control area in male : 0.70 (0.44–1.11) in female : 0.58 (0.34–1.00) SIR (95%CI) in males : 0.72 (0.53–0.96) in females : 0.73 (0.49–1.04) |  |  |
|  |  |  |  |  | Colon cancer incidence | 15 | SIR (95%CI) in males : 0.58 (0.23–1.20) in females : 0.73 (0.32–1.45) |  |  |
|  |  |  |  |  | Rectum cancer incidence | 12 | SIR (95%CI) in males : 0.45 (0.15–1.06) in females : 0.96 (0.38–1.97) |  |  |
|  |  |  |  |  | Liver cancer incidence | 25 | SIR (95%CI) in males : 1.10 (0.69–1.66) in females : 0.36 (0.07–1.05) |  |  |
|  |  |  |  |  | Gallbladder cancer incidence | 5 | SIR (95%CI) in males : 0.22 (0.00–1.22) in females : 0.68 (0.18–1.74) |  |  |
|  |  |  |  |  | Pancreas cancer incidence | 15 | SIR (95%CI) in males : 0.57 (0.19–1.34) in females : 1.62 (0.77–2.97) |  |  |
|  |  |  |  |  | Nose cancer incidence | 5 | SIR (95%CI) in males : 1.44 (0.16–5.19) in females : 3.38 (0.68–9.87) |  |  |
|  |  |  |  |  | Breast cancer incidence | 6 | SIR (95%CI) : 0.55 (0.20–1.20) |  |  |
|  |  |  |  |  | Uterus cancer incidence | 8 | SIR (95%CI) : 0.64 (0.27–1.25) |  |  |
|  |  |  |  |  | Ovary cancer incidence | 1 | SIR (95%CI) : 0.29 (0.00–1.63) |  |  |
|  |  |  |  |  | Prostate cancer incidence | 6 | SIR (95%CI) : 1.43 (0.38–3.66) |  |  |
|  |  |  |  |  | Bladder cancer incidence | 7 | SIR (95%CI) in males : 1.08 (0.39–2.35) in females : 0.94 (0.11–3.39) |  |  |
|  |  |  |  |  | Lymphoma incidence | 14 | SIR (95%CI) in males : 1.97 (0.94–3.63) in females : 1.10 (0.30–2.82) |  |  |
|  |  |  |  |  | All leukaemia incidence | 9 | SIR (95%CI) in males : 2.38 (0.77–5.54) in females : phoma :  Leukaemia : 2.24 (0.60–5.73) |  |  |
|  |  |  |  |  |  |  |  |  |  |
| Mifune et al. 1992 Japan 1952 - 1988 | Ecological study | Adults in the GP n = 1 urban area with high radon level (study area) and 4 farming villages control area in Misasa Age ≥ 40 | _ | Age, sex, period, area | Extrathoracic cancer mortality | 0 | SMR ; p-value in males : 0.000 ; p >0.05 in females : 0.000 ; p >0.05 | _ | Moderate |
|  |  |  |  |  | Stomach cancer mortality | 28 | RR, relative risk (95%CI) for the study area when mortality in the control area was taken as the reference RR (95%CI): 0.59 (0.39–0.88) |  |  |
|  |  |  |  |  | Pancreas cancer mortality | 4 | SMR ; p-value in males : 0.539 ; p >0.05 in females : 0.649 ; p >0.05 |  |  |
|  |  |  |  |  | Peritoneum cancer mortality | 3 | SMR ; p-value in males : 0.950 ; p >0.05 in females : 1.721 ; p >0.05 |  |  |
|  |  |  |  |  | Colorectum cancer mortality | 3 | RR (95%CI) : 0.32 (0.10 - 1.06) SMR ; p-value in males : 0.000 ; p >0.05 in females : 0.000 ; p >0.05 |  |  |
|  |  |  |  |  | Breast cancer mortality | 1 | SMR : 0.257; p >0.05 |  |  |
|  |  |  |  |  | Uterus cancer mortality | 4 | SMR : 0.446 ; p >0.05 |  |  |
| Suzuki et al. 1994 Japan 1989- 1991 | Ecological study | Adults in the GP n = 1 studied city in low radon spa area and 5 control cities Age ≥ 40 | Radon measurement in Spa area | Age, sex | Stomach cancer mortality | 71 | RR, relative risk (95%CI) which represente the ratio of the SMR in the study area (Beppu town) compare to that in the control town (Kitsuki). The reference population for SMR is the Japan population RR (95%CI) : 0.50 (0.30 - 0.83) SMR ; p-value in males : 0.778 ; p <0.05 in females : 0.872 ; p >0.05 | _ | Moderate |
|  |  |  |  |  | Liver cancer mortality | 43 | RR (95%CI): 1.54 (0.86 - 2.76) SMR ; p-value In male : 1.551 ; p <0.05 In female : 1.473 ; p <0.05 |  |  |
|  |  |  |  |  | Rectum cancer mortality | 137 | SMR ; p-value In male : 1.364 ; p >0.05 In female : 1.046 ; p >0.05 |  |  |
|  |  |  |  |  | Colorectum cancer mortality | _ | RR (95%CI): 1.09 (0.61 - 1.96) |  |  |
| Ha et al. 2017 Korea 1999 - 2008 | Ecological study | Adults in the GP n = 234 community regions | Region-level average radon exposure Median : 57 Bq/m3 | Smoking rate, regional deprivation index | Leukaemia and Non-Hodgkin lymphoma incidence |  | RR, relative risk (95%CrI) per 10 Bq/m3 increase in radon For male : 0.98 (0.96 - 1.00) For female : 0.98 (0.95 - 1.00) | _ | Moderate |
|  |  |  |  |  | Non-Hodgkin lymphoma incidence |  | RR, relative risk (95%CrI) per 10 Bq/m3 increase in radon For male : 1.00 (0.98 - 1.02) For female : 1.04 (1.02, 1.07) |  |  |
| Reddy et al. 2009 USA 1992 - 2002 | Ecological study | Adults in the GP n = 210 counties | County-level average radon exposure in pCi/L | _ | Pancreatic cancer incidence |  | Spearman correlation between county-level radon and county-level age-standardize incidence rates of pancreatic cancer : r = 0.042 ; p = 0.582 | _ | Moderate |
| Etherington et al. 1996 UK 1989 - 1992 | Ecological study | Adults in the GP n = 4 postcode sectors in Cornwall and Devon counties | Postcode sectors-level average radon exposure (subdivided in 10 categories) | Sex; age | Esophagus cancer incidence | 696 | Spearman correlation coeficients r for age-standardised incidence rates across the ten domestic radon classes ; and p-value In male : r = -0.0545 ; p = 0.881 In female : r = 0.0303 ; p = 0.9338 | _ | Moderate |
|  |  |  |  |  | Stomach cancer incidence | 1009 | In male : r = -0.3818 ; p = 0.2763 In female : r = 0.2848 ; p = 0.425 |  |  |
|  |  |  |  |  | Colon cancer incidence | 2607 | In male : r = -0.0545 ; p = 0.881 In female : r = 0.7576 ; p = 0.0111 |  |  |
|  |  |  |  |  | Rectum cancer incidence | 1518 | In male : r = -0.6970 ; p = 0.0251 In female : r = 0.0424 ; p = 0.9074 |  |  |
|  |  |  |  |  | Pancreas cancer incidence | 699 | In male : r = -0.7091 ; p = 0.0217 In female : r = 0.0303 ; p = 0.9338 |  |  |
|  |  |  |  |  | Malignant melanoma incidence | 826 | In male : r = 0.3697 ; p = 0.2931 In female : r = 0.7818 ; p = 0.0075 |  |  |
|  |  |  |  |  | Non-melanoma skin cancer incidence | 6531 | In male : r = 0.7333 ; p = 0.0158 In female : r = 0.7454 ; p = 0.0133 |  |  |
|  |  |  |  |  | Prostate cancer incidence | 1947 | r = 0.7091 ; p = 0.0217 |  |  |
|  |  |  |  |  | Female breast cancer incidence | 4198 | r = 0.1634 ; p = 0.6515 |  |  |
|  |  |  |  |  | Cervix uteri cancer incidence | 2600 | r = -0.9000 ; p = 0.0374 |  |  |
|  |  |  |  |  | Ovary cancer incidence | 610 | r = 0.3212 ; p = 0.3655 |  |  |
|  |  |  |  |  | Bladder cancer incidence | 1301 | In male : r = -0.1879 ; p = 0.6032 In female : r = 0.4182 ; p = 0.2291 |  |  |
| Tomasek et al. 2002 Czech republic 1961 - 1999 | Cohort study | Adults in the GP n = 11,803 | Direct radon measurement in individuals' home. | _ | Cancer other than lung cancer mortality | 564 | SMR Cancer other than lung : 0.81 | 6 | Moderate |
| Viel 1993 France 1984 - 1986 | Ecological study | Adults in the GP n = 41 departments Age : 35 - 84 years | Department-level average radon exposure mean : 49 Bq/m3 | Indoor gamma ray dose, socioeconomic status (percentage of workmen in the employed population), and linear geographical gradient (tatitude, longitude) | Acute leukaemia mortality | 219 | OR (95%CI) associated to the median exposure in the highest tertile compared with the median exposure in the lowest tertile : 0.81 (0.59-1.12) | _ | Moderate |
|  |  |  |  |  | Acute myeloid leukaemia mortality | 1060 | OR (95%CI) associated to the median exposure in the highest tertile compared with the median exposure in the lowest tertile : 1.41 (1.23-1.62) |  |  |
|  |  |  |  |  | Acute myeloid leukaemia mortality | 219 | SMR (95%CI) : 0.964 (0.841-1.101) SMRs across radon exposure tertiles indicate no association |  |  |
|  |  |  |  |  | Acute myeloid leukaemia mortality | 1060 | SMR (95%CI) : 1.083 (1.019-1.150) SMRs across radon exposure tertiles indicate no association |  |  |
| Eatough et al. 1993 UK 1975 - 1986 | Ecological study | Children and Adults in the GP Age : 0-74 years | County-level radon exposure | _ | Monocytic leukaemia incidence | _ | Spearman correlation coef r : r=0 75 ; p<10^-6 | _ | Moderate |
|  |  |  |  |  | Myeloid leukeamia incidence |  | Spearman correlation coef : r=0 43 ; p<0.005 |  |  |
|  |  |  |  |  | Lymphoblastic leukaemia incidence |  | Spearman correlation coef : r =0.40 ; p<0.005 |  |  |
|  |  |  |  |  | Other specified leukaemia |  | Spearman correlation coef : r =0.13 ; p> 0.05 |  |  |
| Cohen et al. 1993 (45) USA | Ecological study | Adults in the general population n = 1600 counties | County-level radon expsoure | County-level prevalence of smoking | Lip cancer mortality | 667 | Square of the correlation coeficient (R-square), its standard deviation (t) between mortality and county-level average radon exposure In males : R-square : 1.8 ; t = 3.1 In female : R-square : 0.2 ; t = 0.4 | _ | Moderate |
|  |  |  |  |  | Oral cavity cancer mortality | 2775 | In males : R-square : 3.3 ; t = -7.0 In female : R-square : 1.2 ; t = -3.8 |  |  |
|  |  |  |  |  | Salivary gland cancer mortality | 1487 | In males : R-square : 1.8 ; t = 3.8 In female : R-square : 1.3 ; t = 2.9 |  |  |
|  |  |  |  |  | Nasopharynx cancer mortality | 1224 | In males : R-square : 0.9 ; t = 2.5 In female : R-square : 2.4 ; t = 3.4 |  |  |
|  |  |  |  |  | Esophagus cancer mortality | 2696 | In males : R-square : 0.7 ; t = -3.1 In female : R-square : 0.1 ; t = -1.1 |  |  |
|  |  |  |  |  | Stomach cancer mortality | 3097 | In males : R-square : 0.1 ; t = 1.0 In female : R-square : 0.1 ; t = 1.5 |  |  |
|  |  |  |  |  | Large intestine cancer mortality | 3167 | In males : R-square : 0.0 ; t = 0.8 In female : R-square : 0.5 ; t = 2.7 |  |  |
|  |  |  |  |  | Rectum cancer mortality | 2985 | In males : R-square : 0.1 ; t = 1.1 In female : R-square : 0.2 ; t = 1.6 |  |  |
|  |  |  |  |  | Liver and gallbladder cancer mortality | 3034 | In males : R-square : 0.4 ; t = -2.3 In female : R-square : 0.6 ; t = 3.0 |  |  |
|  |  |  |  |  | Pancreatic cancer mortality | 3143 | In males : R-square : 1.3 ; t = -4.4 In female : R-square : 0.0 ; t = -0.6 |  |  |
|  |  |  |  |  | Nose cancer mortality | 1385 | In males : R-square : 1.3 ; t = 3.1 In female : R-square : 0.5 ; t = 1.6 |  |  |
|  |  |  |  |  | Larynx cancer mortality | 2132 | In males : R-square : 1.2 ; t = -4.0 In female : R-square : 0.8 ; t = 2.3 |  |  |
|  |  |  |  |  | Bone cancer mortality | 2226 | In males : R-square : 0.6 ; t = -2.7 In female : R-square : 1.1 ; t = 3.3 |  |  |
|  |  |  |  |  | Connective tissue cancer mortality | 2328 | In males : R-square : 0.3 ; t = 2.0 In female : R-square : 1.1 ; t = 3.4 |  |  |
|  |  |  |  |  | Malignant melanoma mortality | 2732 | In males : R-square : 1.2 ; t = -4.0 In female : R-square : 0.0 ; t = -0.2 |  |  |
|  |  |  |  |  | Non-melanoma skin cancer mortality | 2180 | In males : R-square : 0.0 ; t = -0.2 In female : R-square : 0.4 ; t = 1.8 |  |  |
|  |  |  |  |  | Breast cancer mortality | 2226 | In males : R-square : 1.4 ; t = 3.0 In female : R-square : 0.0 ; t = 0.9 |  |  |
|  |  |  |  |  | Cervix cancer mortality | 1523 | R-square : 0.0 ; t = 0.0 |  |  |
|  |  |  |  |  | Corpus uterus cancer mortality | 1531 | R-square : 0.8 ; t = 3.5 |  |  |
|  |  |  |  |  | Ovary cancer mortality | 1577 | R-square : 0.1 ; t = 1.5 |  |  |
|  |  |  |  |  | Prostate cancer mortality | 1572 | R-square : 0.4 ; t = 2.5 |  |  |
|  |  |  |  |  | Testis cancer mortality | 505 | R-square : 3.9 ; t = 4.4 |  |  |
|  |  |  |  |  | Bladder and other urinary organs cancer mortality | 2983 | In males : R-square : 0.2 ; t = -1.5 In female : R-square : 0.3 ; t = -2.0 |  |  |
|  |  |  |  |  | Kidney, and ureter cancer mortality | 2991 | In males : R-square : 0.1 ; t = -1.3 In female : R-square : 1.1 ; t = 3.9 |  |  |
|  |  |  |  |  | Eye cancer mortality | 1038 | In males : R-square : 2.6 ; t = 3.7 In female : R-square : 1.9 ; t = 3.0 |  |  |
|  |  |  |  |  | Brain and central nervous system cancer mortality | 3028 | In males : R-square : 0.0 ; t = 0.5 In female : R-square : 0.0 ; t = -0.3 |  |  |
|  |  |  |  |  | Thyroid gland cancer mortality | 1746 | In males : R-square : 3.6 ; t = 5.2 In female : R-square : 0.0 ; t = -0.4 |  |  |
|  |  |  |  |  | Thymus, endocrine glands cancer mortality | 1217 | In males : R-square : 3.9 ; t = 5.0 In female : R-square : 4.6 ; t = 5.2 |  |  |
|  |  |  |  |  | Lymphosarcoma, reticulo Sarcoma cancer mortality | 3077 | In males : R-square : 0.0 ; t = 0.8 In female : R-square : 0.7 ; t = 3.3 |  |  |
|  |  |  |  |  | Hodgkin lymphoma mortality | 2551 | In males : R-square : 0.3 ; t = 1.9 In female : R-square : 0.9 ; t = 3.3 |  |  |
|  |  |  |  |  | Multiple myeloma mortality | 2892 | In males : R-square : 0.7 ; t = 3.1 In female : R-square : 0.6 ; t = 3.0 |  |  |
|  |  |  |  |  | Leukaemia mortality | 3127 | In males : R-square : 0.0 ; t = 0.8 In female : R-square : 0.4 ; t = 2.4 |  |  |
| Miller et al. 1993 (46) USA 1986 - 1988 | Ecological study | Adults in the GP n = 67 counties in Pennsylvania | County-level average radon exposure (pCi/L) | _ | Malignant salivary gland tumors | 345 | Correlation with county-level radon and malignant salivary gland tumors (r^2^) In 1986 : 0.162 In 1987 : 0.251 In 1988 : 0.705 | _ | Moderate |
|  |  |  |  |  | Minor salivary gland tumors incidence | 88 | Correlation with county-level radon r^2^ : 0.716 ; p>0.05 |  |  |
| Miller et al. 1993 (47) Canada 1982 - 1989 | Ecological study | Adults in the GP n = 18 cities | City-level radon concentration | Age, sex, proportion of apartment dwellers | Acute myeloid leukaemia incidence | _ | Correlation between city-level radon and acute myeloid leukaemia r = 0.25 ; p = 0.32 | _ | Moderate |
| Neuberger et al. 1996 (48) USA 1973 - 1993 | Ecological study | Adults in the GP n = 77 counties | County-level average radon exposure (in pCi/L) derived from short-term direct radon measurements in sampled home | Age | In situ and invasive breast cancer incidence | 32,171 | No increase in invasive breast cancer risk in the highest radon counties  of Iowa. No dose response trend | _ | Insufficient information to assess the quality since it is a letter to the editor |
| Lucie et al. 1990 (22) UK | Ecological study | Adults in the GP n = 22 counties | County-level indoor radon concentration | _ | Acute lymphoblastic leukaemia incidence | _ | Weak correlation between county-level radon exposure and Acute lymphoblastic leukaemia | _ | Insufficient information to assess the quality since wit is a letter to the editor |
| Henshaw et al. 1990 (23) UK Canada & Danemark & Finland & France & Germany & Ireland & Italy & Japan & Norway & Netherla,ds & Poland & Switzerland & Sweden & UK & USA | Pooled ecological study | Adults in the GP n = up to 14 countries | Country/region-level radon exposure (Bq/m3) | _ | Myeloid leukaemia incidence | _ | Correlation with country/region-level radon exposure r = 0.65; p < 0.02 | _ | Moderate |
|  |  |  |  |  | Melanoma incidence |  | Correlation with country/region-level radon exposure r = 0.81; p < 0.001 |  |  |
| Eatough et al. 1990 (49) Five continents | Pooled ecological study | Adults in the GP n = 14 countries from the five continents | Population-level average radon exposure | _ | Prostate incidence | _ | Correlation with population-level average radon exposure : r = 0.72 ; p < 0.01 | _ | Moderate |
|  |  |  |  |  | Testis cancer incidence |  | Correlation with population-level average radon exposure : r = 0.45 ; p >0.05 |  |  |
|  |  |  |  |  | Ovary cancer incidence |  | Correlation with population-level average radon exposure : r = 0.51 ; p >0.05 |  |  |
|  |  |  |  |  | Corpus uteri cancer incidence |  | Correlation with population-level average radon exposure : r = 0.49 ; p > 0.05 |  |  |
|  |  |  |  |  | Cervix uteri cancer incidence |  | Correlation with population-level average radon exposure : r = -0.29 ; p > 0.05 |  |  |
|  |  |  |  |  | Unspecified cancer incidence |  | Correlation with population-level average radon exposure : r = -0.41 ; p > 0.05 |  |  |
|  |  |  |  |  | Other female genital |  | Correlation with population-level average radon exposure : r = -0.25 ; p > 0.05 |  |  |
|  |  |  |  |  | Lymphosarcoma incidence |  | Correlation with population-level average radon exposure : In male : r = 0.75 ; p <0.01 In female : r = 0.71 ; p <0.01 |  |  |
| Schwartz et al. 2019 (50) USA 1997 - 2014 | Pooled ecological study | Adults in the GP n = 26 counties | County-level radon exposure | Age, race | Thyroid cancer incidence | _ | No association with radon | _ | Moderate |
| Law et al. 2000 (51) UK 1991 - 1996 | Case-control study | Adults in the GP n = 1561 Age : 16 - 69 years | Direct radon measurement in individual home at diagnosis | Age, sex, deprivation | Acute leukaemia incidence | 578/983 | OR (95%CI) across radon concentration categories in Bq/m3 0 - 24 : 1.00 (reference) 25 - 49 : 1·20 (0·90–1·60) 50 - 99 : 0·82 (0·53–1·25) 100 - 199 : 0·55 (0·28–1·11) 200+ : 0·90 (0·31–2·62) Analysis according to quintiles of radon concentration yield similar results | 7 | Moderate |
| Kjellberg et al. 1995 (52) USA 1985 - 1989 | Ecological study | Adults in the GP Counties in Pennsylvania | Town-level outdoor radon measurement in soil samples (in Bq/m3) | Sex | Stomach cancer incidence | _ | Positive correlation between radon and incidence of stomach cancer in females. Positive correlation with stomach cancer mortality in males, females, and the whole study population. | _ | Insufficient information to assess the quality since we had access only to the abstract |
| Zlobina et al. 2022 (18) Russia, China, France | Ecological study | Adults in the GP n = 4 towns | Town-level outdoor radon measurement in soil samples (in Bq/m3) | _ | Leukaemia incidence | _ | Correlation betweew city-level incidence rates and city-level outdoor radon expressed as R-square : 0.45 ; p = 0.008 | _ | Moderate |
|  |  |  |  | _ | Nasopharyngal carcinoma incidence |  | Coorrelation betweew city-level incidence rates and city-level outdoor radon expressed as R-square : 0.25 ; p = 0.0079 |  |  |
| Schubauer- Berigan et al. 2009 (53) USA 1960- 2005 | Cohort study | Colorado plateau uranium miners n = 4022 (3255 (White Americans, 767 American Indians) | Radon exposure assessment is based on the mine location and year, combined with available radon progeny measurement data. Exposure period : 1950 - 1960 Mean cumulative in WLM In white american : 806 In american indian : 742 | Age, calendar year, race | All cancers other than lung mortality | 2076 | SMRs (95%CI) in White American : 1.21 (1.08 - 1.36)  in American Indian : 0.85 (0.64 - 1.11) | 7 | Moderate |
|  |  |  |  |  | Buccal cavity and pharyngeal cancer mortality | 6 | SMRs (95%CI) in White American : 0.86 (0.32 - 1.88) in American Indian : 0 (0 - 2.24) |  |  |
|  |  |  |  |  | Intestine and rectal cancer mortality | 39 | SMRs (95%CI) in White American : 0.93 (0.64 -1.30)  in American Indian : 0.73 (0.24 - 1.82) |  |  |
|  |  |  |  |  | Peritoneum and other and unspecified digestive organs cancer mortality | 4 | SMRs (95%CI) in White American : 2.12 (0.44 - 6.19)  in American Indian : 2.18 (0.06 - 12.2) |  |  |
|  |  |  |  |  | Breast cancer mortality | 1 | SMRs (95%CI) in White American : 2.60 (0.07 - 14.5)  in American Indian : 0 (0 - 46.3) |  |  |
|  |  |  |  |  | Bone cancer mortality | 3 | SMRs (95%CI) in White American : 3.91 (0.81 - 11.4)  in American Indian : 0 (0 - 32.4) |  |  |
|  |  |  |  |  | Malignant melanoma mortality | 8 | SMRs (95%CI) in White American : 1.28 (0.55 - 2.52)  in American Indian : 0 (0 - 9.85) |  |  |
|  |  |  |  |  | Non-melanoma skin cancer mortality | 6 | SMRs (95%CI) in White American : 2.52 (0.82 - 5.89)  in American Indian : 4.56 (0.12 - 25.4) |  |  |
|  |  |  |  |  | Other and unspecific site cancer mortality | 56 | SMRs (95%CI) in White American : 1.77 (1.30 - 2.35)  in American Indian : 1.14 (0.52- 2.17) |  |  |
|  |  |  |  |  | Lymphohematological cancer mortality | 54 | SMRs (95%CI) in White American : 1.38 (1.03 - 1.82)  in American Indian : 0.41 (0.08 - 1.20) |  |  |
|  |  |  |  |  | Non-Hodgkin lymphoma mortality | 22 | The internally SRRs (95%CI) across radon exposure categories in WLM for the whole study population <120 : 1.00 (reference) 120–<400 : 0.68 (0.21 - 2.2) 400–<1000 : 0.39 (0.10 - 1.6) ≥ 1000 : 1.1 (0.40 - 3.2) SMRs (95%CI) across radon exposure categories indicate no statistically significant association |  |  |
|  |  | Colorado plateau White American uranium miners n = 3255 |  |  | Multiple myeloma mortality | 13 | The internally SRRs (95%CI) across radon categories in WLM among the White Americans <120 : 1.00 (reference) 120–<400 : 0.65 (0.14 - 3.0) 400–<1000 : 1.1 (0.29 - 4.1) ≥ 1000 : 0.23 (0.03 - 2.0) The SMRs across radon exposure categories indicate no statistically significant association |  |  |
| Roscoe et al. 1997 (54) USA 1960 - 1990 | Cohort study | Colorado plateau White American uranium miners n = 3238 Mean age at death : 61 | Radon exposure assessment is based on the mine location and year, combined with available radon progeny measurement data. Exposure period : 1950 - 1964 Mean cumulative radon exposure in WLM (SD) : 811 (1107.1) | Age, sex, calendar year, race | Digestive cancer mortality | 63 | SMR (95%CI) : 1.2 (0.9 - 1.5) | 8 | Moderate |
|  |  |  |  |  | Male genital cancer mortality | 23 | SMR (95%CI) : 1.3 (0.8–1.9) |  |  |
|  |  |  |  |  | All cancer except lung mortality | 170 | SMRs (95%CI) across radon categories  <120 : 1.2 ( 0.8–1.6) 120–<400 : 1.1 (0.8–1.6) 400–≤1000 : 1.4 (1.0–1.8) > 1000 : 1.4 (1.0–1.8) SRRs across radon categories indicate no statistically significant association |  |  |
|  |  |  |  |  | All leukaemia mortality | 13 | The internally SRRs (95%CI) across radon categories  <120 : 1.00 (reference) 120–<400 : 1.5 (0.4–6.1) 400–≤1000 : 0.7 ( 0.1–4.6) >1000 : 1.0 ( 0.1–3.2) SMRs across radon categories indicate no statistically significant association |  |  |
| Rericha et al. 2006 (55) Czech Republic 1977 - 2001 (for leukaemia incidence), 1977 - 1996 (for lymphoma and multiple myeloma incidences) | Case-cohort study | Příbram region uranium miners n = 2558 including subcohort (n = 2393) mean age at start of follow-up : 50  years. | Mean cumulative radon exposure in WLM : 64.1 | Smoking (1) non-smokers and light : less than 10 cigarettes a day for a period not exceeding 5 years; 2) moderate and heavy smokers : over the limit for light smokers; 3) unknown smoking status) | Lymphohematological cancer (leukaemia lymphoma, multiple myeloma) incidence | 170 | RR, relative risk (95%CI) compares 110 WLM (80th percentile of the cumulative lifetime dose) to 3 WLM (20th percentile) : 1.22 (0.87–1.72) ; p = 0.24 | 6 | Moderate |
| Rericha et al. 2006 (55) Czech Republic 1977 - 1996 |  |  |  |  | Hodgkin lymphoma incidence | 23 | RR (95%CI) compare 110 WLM to 3 WLM : 2.12 (0.81–5.52) ; p = 0.12 |  |  |
|  |  |  |  |  | Non-Hodgkin lymphoma incidence | 44 | RR (95%CI) compare 110 WLM to 3 WLM : 0.80 (0.46–1.37) ; p = 0.4 |  |  |
|  |  |  |  |  | Multiple myeloma incidence | 26 | RR (95%CI) compare 110 WLM to 3 WLM : 1.03 (0.47–2.27) ; p : 0.94 |  |  |
| Rericha et al. 2006 (55) Czech Republic 1977 - 2001 |  |  |  |  | Leukaemia incidence | 84 | RR (95%CI) compare 110 WLM to 3 WLM : 1.75 (1.10–2.78) ; p = 0.014 |  |  |
|  |  |  |  |  | Chronic lymphoblastic leukaemia incidence | 53 | RR (95%CI) compare 110 WLM to 3 WLM : 1.98 (1.10–3.59) ; p = 0.016 |  |  |
|  |  |  |  |  | Myeloid leukaemia | 25 | RR (95%CI) compare 110 WLM to 3 WLM : 1.86 (0.79–4.36) ; p = 0.14 |  |  |
| Kelly-Reif et al. 2022 (56) Czech Republic 1977 - 1996 (13.6) | Case-cohort study | Příbram region underground uranium miners (males) n = 1,826 males Mean age at the start of follow-up : 50.7 | Individual annual radon exposure concentration estimates were based on measurements from area monitors. Exposure period : 1946 - 1991 Mean cumulative radon exposure in WLM : 69 | for the continuous model : age, birth cohort, and smoking (ever  smoker; never smoker) for the categorical model : age and birth cohort | Extra-thoracic cancer incidence | 127 | ERR (95%CI) per 100 WLM increase in cumulative radon exposure : 0.07 (-0.17 - 0.72) Relative Rate (95%CI) across categories of cumulative radon exposure 0 - <3 : 1 (ref)  3 - <5 : 3.85 (1.58 - 9.39)  5 - <10 : 2.42 (1.19 - 4.93) 10 - <50 : 1.26 (0.66 - 2.41)  50 - <100 : 1.05 (0.5 - 2.24)  100+ : 1.3 (0.64 - 2.65) | 7 | Moderate |
| Kulich et al. 2011 (57) Czech Republic 1977 - 1996 | Case-cohort study | Příbram region underground uranium miners n = 1707 including subcohort controls (n = 1486) mean age at the start of follow-up : 50.4 years. | Exposure period : 1949 - 1975 Mean cumulative radon in WLM : 84.0 | Age, calendar time, smoking ((1) non-smokers and light : less than 10 cigarettes a day for a period not exceeding 5 years; 2) moderate and heavy smokers : over the limit for light smokers; 3) unknown smoking status) | Non-lung solid cancer incidence | 1020 | RR, Relative Risk (95%CI) compare 180 WLM (90th percentile of the cumulative lifetime dose) to 3 WLM (10th percentile) : 0.88 (0.73–1.04) RRs across radon exposure categories indicate no statistically significant association | 7 | Moderate |
|  |  |  |  |  | Buccal and pharyngeal cancer incidence | 69 | RR (95%CI) compare 180 WLM to 3 WLM : 0.48 (0.21–1.12) RRs (95%CI) across radon exposure categories indicate a statistically significant lower risk and negative linear trend |  |  |
|  |  |  |  |  | All digestive cancer incidence | 561 | RR (95%CI) compare 180 WLM to 3 WLM : 0.87 (0.69–1.09) RRs across radon exposure categories indicate no statistically significant association |  |  |
|  |  |  |  |  | Stomach cancer incidence | 138 | RR (95%CI) compare 180 WLM to 3 WLM : 0.66 (0.43–1.03) RRs across radon exposure categories indicate no statistically significant association |  |  |
|  |  |  |  |  | Colon cancer incidence | 109 | RR (95%CI) compare 180 WLM to 3 WLM :1.23 (0.74–2.07) RRs across radon exposure categories indicate no statistically significant association |  |  |
|  |  |  |  |  | Rectum cancer incidence | 145 | RR (95%CI) compare 180 WLM to 3 WLM : 0.78 (0.50–1.20) RRs across radon exposure categories indicate no statistically significant association |  |  |
|  |  |  |  |  | Liver cancer incidence | 50 | RR (95%CI) compare 180 WLM to 3 WLM : 1.17 (0.55–2.50) RRs across radon exposure categories indicate no statistically significant association |  |  |
|  |  |  |  |  | Gallbladder cancer incidence | 13 | RR (95%CI) compare 180 WLM to 3 WLM : 2.39 (0.52–10.98) RRs across radon exposure categories indicate no statistically significant association |  |  |
|  |  |  |  |  | Pancreas cancer incidence | 61 | RR (95%CI) compare 180 WLM to 3 WLM : 0.89 (0.41–1.92) RRs across radon exposure categories indicate no statistically significant association |  |  |
|  |  |  |  |  | Larynx cancer incidence | 62 | RR (95%CI) compare 180 WLM to 3 WLM : 0.79 (0.38–1.64) RRs across radon exposure categories indicate no statistically significant association |  |  |
|  |  |  |  |  | Melanoma incidence | 23 | RR (95%CI) compare 180 WLM to 3 WLM : 2.92 (0.91–9.42) RRs across radon exposure categories indicate no statistically significant association, except an increase risk for exposure between 50 and 100 WLM |  |  |
|  |  |  |  |  | Genitourinary cancer incidence | 231 | RR (95%CI) compare 180 WLM to 3 WLM : 0.82 (0.57–1.18) RRs across radon exposure categories indicate no statistically significant association, except a decrease risk for exposure between 50 and 100 WLM |  |  |
|  |  |  |  |  | Prostate cancer incidence | 73 | RR (95%CI) compare 180 WLM to 3 WLM : 0.68 (0.36–1.28) RRs across radon exposure categories indicate no statistically significant association |  |  |
|  |  |  |  |  | Bladder cancer incidence | 73 | RR (95%CI) compare 180 WLM to 3 WLM : 0.84 (0.43–1.65) RRs across radon exposure categories indicate no statistically significant association |  |  |
|  |  |  |  |  | Kidney and other and unspecific urinary organs cancer incidence | 66 | RR (95%CI) compare 180 WLM to 3 WLM : 1.13 (0.62–2.04) RRs across radon exposure categories indicate no statistically significant association |  |  |
| Kelly-Reif et al. 2019 (58) Czech Republic 1977 - 1992 | Cohort study | Příbram region underground uranium miners n = 16,434 Mean age at hire : 28 | Exposure period : 1946 - 1976 Mean cumulative radon in WLM (range) 53.2 (1.2–1121.9) | Age, sex | Lip cancer incidence | 6 | SIR (95%CI) : 0.70 (0.14 -1.26) | 7 | Moderate |
|  |  |  |  |  | Tongue cancer incidence | 12 | SIR (95%CI) : 1.58 (0.68 - 2.48) |  |  |
|  |  |  |  |  | Oropharynx cancer incidence | 6 | SIR (95%CI) : 0.78 (0.15 - 1.40) |  |  |
|  |  |  |  |  | Buccal and Pharynx cancer incidence | 41 | SIR (95%CI) : 0.98 (0.71 - 1.31) |  |  |
|  |  |  |  |  | Esophagus cancer incidence | 19 | SIR (95%CI) : 1.36 (0.74 - 1.97) |  |  |
|  |  |  |  |  | Stomach cancer incidence | 108 | SIR (95%CI) : 1.37 (1.11 - 1.63) |  |  |
|  |  |  |  |  | Small intestine, including duodenum cancer incidence | 7 | SIR (95%CI) : 3.26 (0.83 - 5.69) |  |  |
|  |  |  |  |  | Colon cancer incidence | 80 | SIR (95%CI) : 1.06 (0.82 - 1.29) |  |  |
|  |  |  |  |  | Rectum cancer incidence | 119 | SIR (95%CI) : 1.41 (1.16 - 1.66) |  |  |
|  |  |  |  |  | Liver cancer incidence | 38 | SIR (95%CI) : 1.70 (1.16 - 2.25) |  |  |
|  |  |  |  |  | Gallbladder cancer incidence | 9 | SIR (95%CI) : 0.61 (0.21 - 1.00) |  |  |
|  |  |  |  |  | Pancreas cancer incidence | 54 | SIR (95%CI) : 1.31 (0.96 - 1.66) |  |  |
|  |  |  |  |  | Other and ill-defined cancer sites incidence | 16 | SIR (95%CI) : 3.29 (1.67 - 4.91) |  |  |
|  |  |  |  |  | Extrathoracic airway cancer incidence | 80 | SIR (95%CI) : 1.16 (0.95 - 1.39) |  |  |
|  |  |  |  |  | Digestive organs and peritoneum cancer incidence | 453 | SIR (95%CI) : 1.33 (1.21 - 1.46) |  |  |
|  |  |  |  |  | Larynx cancer incience | 45 | SIR (95%CI) : 1.34 (0.95 - 1.73) |  |  |
|  |  |  |  |  | Connective and other soft tissue cancer incidence | 5 | SIR (95%CI) : 0.72 (0.09 - 1.36) |  |  |
|  |  |  |  |  | Malignant melanoma incidence | 18 | SIR (95%CI) : 0.77 (0.41 - 1.13) |  |  |
|  |  |  |  |  | Non-melanoma skin cancer incidence | 129 | SIR (95%CI) : 0.68 (0.56 - 0.80) |  |  |
|  |  |  |  |  | Bone, connective tissue, skin, and breast cancers incidence | 157 | SIR (95%CI) : 0.70 (0.59 - 0.81) |  |  |
|  |  |  |  |  | Prostate cancer incidence | 57 | SIR (95%CI) : 0.86 (0.64 - 1.09) |  |  |
|  |  |  |  |  | Testis cancer incidence | 10 | SIR (95%CI) : 0.85 (0.32 - 1.38) |  |  |
|  |  |  |  |  | Penis cancer incidence | 6 | SIR (95%CI) : 1.76 (0.35 - 3.18) |  |  |
|  |  |  |  |  | Bladder cancer incidence | 54 | SIR (95%CI) : 1.06 (0.78 - 1.35) |  |  |
|  |  |  |  |  | Kidney and other and unspecific urinary organs cancer incidence | 49 | SIR (95%CI) : 0.88 (0.63 - 1.12) |  |  |
|  |  |  |  |  | Genitourinary Organs cancer incidence | 176 | SIR (95%CI) : 0.94 (0.81 - 1.08) |  |  |
|  |  |  |  |  | Brain cancer incidence | 13 | SIR (95%CI) : 0.83 (0.38 - 1.29) |  |  |
|  |  |  |  |  | Thyroid gland cancer incidence | 5 | SIR (95%CI) : 1.17 (0.14 - 2.20) |  |  |
|  |  |  |  |  | Lymphosarcoma and reticulosarcoma incidence | 7 | SIR (95%CI) : 0.94 (0.24 - 1.64) |  |  |
|  |  |  |  |  | Hodgkin-lymphoma incidence | 15 | SIR (95%CI) : 1.57 (0.77 - 2.37) |  |  |
|  |  |  |  |  | Other lymphoid and histiocytic tissue cancer incidence | 10 | SIR (95%CI) : 0.71 (0.27 - 1.15) |  |  |
|  |  |  |  |  | Multiple myeloma incidence | 16 | SIR (95%CI) : 1.75 (0.89 - 2.61) |  |  |
|  |  |  |  |  | Lymphoblastic leukaemia incidence | 21 | SIR (95%CI) : 1.57 (0.90 - 2.25) |  |  |
|  |  |  |  |  | Myeloid leukaemia incidence | 14 | SIR (95%CI) : 1.58 (0.75 - 2.42) |  |  |
|  |  |  |  |  | All Leukaemia incidence | 37 | SIR (95%CI) : 1.51 (1.08 - 2.07) |  |  |
|  |  |  |  |  | Lymphohematological cancer incidence | 85 | SIR (95%CI) : 1.31 (1.05 - 1.61) |  |  |
| Kelly-Reif et al. 2019 (58) Czech Republic 1977 - 1992 | Cohort study | Příbram region underground uranium miners n = 16,434 Mean age at hire : 28 | Exposure period : 1946 - 1976 Mean cumulative radon in WLM (range) 53.2 (1.2–1121.9) | Age, sex | Tongue cancer mortality | 9 | SMR (95%CI) : 1.41 (0.48 - 2.33) | 7 | Moderate |
|  |  |  |  |  | Oropharynx cancer mortality | 3 | SMR (95%CI) : 0.66 (0.00 -1.41) |  |  |
|  |  |  |  |  | Buccal and Pharynx cancer mortality | 25 | SMR (95%CI) : 1.14 (0.75 - 1.65) |  |  |
|  |  |  |  |  | Small intestine, including duodenum cancer mortality | 6 | SMR (95%CI) : 2.91 (0.57 - 5.25) |  |  |
|  |  |  |  |  | Colon cancer mortality | 54 | SMR (95%CI) : 0.90 (0.66 - 1.15) |  |  |
|  |  |  |  |  | Liver cancer mortality | 48 | SMR (95%CI) : 1.63 (1.17 - 2.10) |  |  |
|  |  |  |  |  | Gallbladder cancer mortality | 13 | SMR (95%CI) : 0.88 (0.40 - 1.37) |  |  |
|  |  |  |  |  | Other and ill-defined cancer sites mortality | 14 | SMR (95%CI) : 1.82 (0.86 - 2.77) |  |  |
|  |  |  |  |  | Extra-thoracic airway cancer mortality | 59 | SMR (95%CI) : 1.41 (1.15 - 1.77) |  |  |
|  |  |  |  |  | Digestive organs and peritoneum cancer mortality | 396 | SMR (95%CI) : 1.25 (1.13 - 1.38) |  |  |
|  |  |  |  |  | Connective and other soft tissue cancer mortality | 3 | SMR (95%CI) : 1.07 (0.00 - 2.28) |  |  |
|  |  |  |  |  | Malignant melanoma mortality | 14 | SMR (95%CI) : 1.18 (0.56 - 1.80) |  |  |
|  |  |  |  |  | Non-melanoma skin cancer mortality | 1 | SMR (95%CI) : 0.39 (0.00 - 1.16) |  |  |
|  |  |  |  |  | Bone, connective tissue, skin, and breast cancers mortality | 22 | SMR (95%CI) : 1.00 (0.64 - 1.49) |  |  |
|  |  |  |  |  | Testis cancer mortality | 4 | SMR (95%CI) : 1.05 (0.02 - 2.09) |  |  |
|  |  |  |  |  | Penis cancer mortality | 4 | SMR (95%CI) : 2.81 (0.04 - 5.57) |  |  |
|  |  |  |  |  | Bladder cancer mortality | 29 | SMR (95%CI) : 1.05 (0.67 - 1.43) |  |  |
|  |  |  |  |  | Kidney, ureter, other urinary organs cancer mortality | 41 | SMR (95%CI) : 1.00 (0.69 - 1.31) |  |  |
|  |  |  |  |  | Genitourinary Organs cancer mortality | 108 | SMR (95%CI) : 0.91 (0.75 - 1.09) |  |  |
|  |  |  |  |  | Brain cancer mortality | 13 | SMR (95%CI) : 0.76 (0.35 - 1.18) |  |  |
|  |  |  |  |  | Thyroid gland cancer mortality | 2 | SMR (95%CI) : 0.91 (0.00 - 2.19) |  |  |
|  |  |  |  |  | Lymphosarcoma and reticulosarcoma mortality | 7 | SMR (95%CI) : 1.57 (0.40 - 2.73) |  |  |
|  |  |  |  |  | Other lymphoid and histiocytic tissue cancer mortality | 10 | SMR (95%CI) : 0.98 (0.37 - 1.58) |  |  |
|  |  |  |  |  | Lymphoblastic leukaemia mortality | 11 | SMR (95%CI) : 1.03 (0.42 -1.65) |  |  |
|  |  |  |  |  | Myeloid leukaemia mortality | 12 | SMR (95%CI) : 1.36 (0.59 - 2.14) |  |  |
|  |  |  |  |  | Lymphohematological cancer mortality | 58 | SMR (95%CI) : 1.09 (0.84 - 1.41) |  |  |
| Kelly-Reif et al. 2020 (59) Czech Republic 1977 - 1992 | Cohort study | Příbram region underground uranium miners (males) n = 16,434 Mean age at hire : 28 | Exposure period : 1949 - 1991 Mean cumulative radon in WLM (range) 53.2 (1.2–1121.9) | Age, birth cohort | Extra-thoracic airway cancer mortality | 59 | RR, Rate Ratio (95%CI) : 1.10 (0.87- 1.39) RRs across radon exposure categories and the ERR indicate no statistically significant association | 6 | Moderate |
|  |  |  |  |  | Stomach cancer mortality | 102 | RR (95%CI) : 1.00 (0.82 - 1.21) RRs across radon exposure categories and the ERR indicate no statistically significant association |  |  |
|  |  |  |  |  | Liver cancer mortality | 48 | RR (95%CI) : 1.08 (0.85 - 1.37) RRs across radon exposure categories and the ERR indicate no statistically significant association |  |  |
|  |  |  |  |  | Kidney and other and unspecific urinary organs cancer mortality | 41 | RR (95%CI) : 1.01 (0.74 - 1.37) The ERR per 100 WLM and RRs across radon exposure categories indicate no statistically significant association, except a significant incease in RR for exposure between 25 and 50 WLM |  |  |
|  |  |  |  |  | Non-Hodgkin lymphoma mortality | 17 | RR (95%CI) : 0.99 (0.57- 1.72) RRs across radon exposure categories and the ERR indicate no statistically significant association |  |  |
|  |  |  |  |  | Hodgkin lymphoma | 8 | RR (95%CI) : 0.52 (0.12 - 2.21) RRs across radon exposure categories and the ERR indicate no statistically significant association |  |  |
|  |  |  |  |  | Multiple myeloma mortality | 8 | RR (95%CI) : 0.98 (0.52 - 1.86) RRs across radon exposure categories and the ERR indicate no statistically significant association |  |  |
|  |  |  |  |  | Chronic lymphoblastic leukaemia mortality | 11 | RR (95%CI) : 1.15 (0.72 - 1.82) RRs across radon exposure categories and the ERR indicate no statistically significant association |  |  |
|  |  |  |  |  | Myeloid leukaemia mortality | 12 | RR (95%CI) : 0.75 (0.33 - 1.67) RRs across radon exposure categories and the ERR indicate no statistically significant association |  |  |
|  |  |  |  |  | All lymphohematological cancer mortality | 58 | RR (95%CI) : 0.91 (0.67 - 1.23) RRs across radon exposure categories and the ERR indicate no statistically significant association |  |  |
| Tomasek et al. 1993 (60) Czech Republic 1948 - 1991 | Cohort study | West Bohemia underground uranium miners n = 4320 | Exposure period : 1948 - 1959 Mean cumulative radon exposure : 219 WLM | Age | All cancer other than lung mortality | 292 | SMR (95%CI) : 1.11 (0.98 - 1.24) | 7 | Moderate |
|  |  |  |  |  | Tongue and mouth cancer mortality | 3 | SMR (95%CI) : 0.70 (0.14 - 2.03) |  |  |
|  |  |  |  |  | Salivary gland cancer mortality | 0 | SMR (95%CI) : 0.00 (0.00 - 5.43) |  |  |
|  |  |  |  |  | Liver cancer mortality | 22 | SMR (95%CI) : 1.67 (1.04 - 2.52) |  |  |
|  |  |  |  |  | Gallbladder cancer mortality | 12 | SMR (95%CI) : 2.26 (1.16 - 3.94) |  |  |
|  |  |  |  |  | Nose cancer mortality | 1 | SMR (95%CI) : 1.70 (0.02 - 9.34) |  |  |
|  |  |  |  |  | Bone cancer mortality | 2 | SMR (95%CI) : 0.69 (0.08 - 2.48) |  |  |
|  |  |  |  |  | Connective and other soft tissue mortality | 2 | SMR (95%CI) : 2.11 (0.23 - 7.56) |  |  |
|  |  |  |  |  | Malignant melanoma | 6 | SMR (95%CI) : 1.75 (0.64 - 3.79) |  |  |
|  |  |  |  |  | Non-melanoma skin cancer mortality | 2 | SMR (95%CI) : 1.36 (0.15 - 4.88) |  |  |
|  |  |  |  |  | Testis cancer mortality | 1 | SMR (95%CI) : 1.10 (0.01 - 6.05) |  |  |
|  |  |  |  |  | Bladder cancer mortality | 13 | SMR (95%CI) : 1.15 (0.61 - 1.97) |  |  |
|  |  |  |  |  | Thyroid cancer mortality | 0 | SMR (95%CI) : 0.00 (0.00 - 3.42) |  |  |
|  |  |  |  |  | Other and unspecified cancer mortality | 21 | SMR (95%CI) : 1.02 (0.63 - 1.56) |  |  |
| Navaranjan et al. 2016 (61) Canada 1969 - 2005 | Cohort study | Ontario uranium miners n = 28,546 males and 413 females (were not included in the dose-response analysis given the small number) Mean age at entry in males : 28.8 in females : 27.9 | Mine-specific extrapolations and areas sampling of radon decay products were used to estimate individual exposure to radon. Mean radon in WLM (range) in males : 21.0 (0.0–875.1) in female : 0.2 (0.0–16.3) | Calendar period, attained age | Extra-thoracic airways cancer incidence | _ | ERR (95%CI) : -0.29 (-0.57 - 0.0034) Relative Risks (RRs) across radon exposure categories indicate no statistically significant association, except a significant decrease in RR for exposure between 20 and 50 WLM | 6 | Moderate |
|  |  |  |  |  | Buccal cavity and pharyngeal cancer incidence | 141 | SIR (95%CI) : 0.70 (0.59 - 0.82) |  |  |
|  |  |  |  |  | Oesophageal cancer incidence | 53 | SIR (95%CI) : 0.77 (0.58 - 1.01) |  |  |
|  |  |  |  |  | Stomach cancer incidence | 127 | SIR (95%CI) : 0.81 (0.67 - 0.96) RRs across radon exposure categories and the ERR indicate no statistically significant association |  |  |
|  |  |  |  |  | Colorectal cancer incidence | 485 | SIR (95%CI) : 0.70 (0.64 - 0.77) |  |  |
|  |  |  |  |  | Liver cancer incidence | 37 | SIR (95%CI) : 0.70 (0.50 - 0.97) |  |  |
|  |  |  |  |  | Pancreatic cancer incidence | 105 | SIR (95%CI) : 0.85 (0.70 - 1.03) |  |  |
|  |  |  |  |  | Laryngeal cancer incidence | 83 | SIR (95%CI) : 0.84 (0.67 - 1.05) |  |  |
|  |  |  |  |  | Bone cancer incidence | 7 | SIR (95%CI) : 0.71 (0.28 - 1.46) |  |  |
|  |  |  |  |  | Connective and other soft tissue cancer incidence | 17 | SIR (95%CI) : 0.58 (0.34 - 0.93) |  |  |
|  |  |  |  |  | Malignant melanoma incidence | 41 | SIR (95%CI) : 0.34 (0.24 - 0.45) |  |  |
|  |  |  |  |  | Breast cancer incidence | 12 | SIR (95%CI) : 1.21 (0.63 - 2.12) |  |  |
|  |  |  |  |  | Prostate cancer incidence | 615 | SIR (95%CI) : 0.56 (0.51 - 0.60) |  |  |
|  |  |  |  |  | Testis cancer incidence | 18 | SIR (95%CI) : 0.52 (0.31 - 0.82) |  |  |
|  |  |  |  |  | Kidney cancer incidence | 100 | SIR (95%CI) : 0.63 (0.51 - 0.76) RRs across radon exposure categories and the ERR indicate no statistically significant association |  |  |
|  |  |  |  |  | Bladder and other urinary cancer incidence | 196 | SIR (95%CI) : 0.68 (0.58 - 0.78) |  |  |
|  |  |  |  |  | Brain and central nervous system cancer incidence | 70 | SIR (95%CI) : 0.78 (0.60 - 0.98) |  |  |
|  |  |  |  |  | Thyroid cancer incidence | 17 | SIR (95%CI) : 0.59 (0.34 - 0.94) |  |  |
|  |  |  |  |  | Hodgkin lymphoma incidence | 19 | SIR (95%CI) : 0.63 (0.38 - 0.99) |  |  |
|  |  |  |  |  | Non- Hodgkin lymphoma incidence | 163 | SIR (95%CI) : 0.84 (0.72 - 0.98) |  |  |
|  |  |  |  |  | Multiple myeloma incidence | 49 | SIR (95%CI) : 0.77 (0.57 - 1.02) |  |  |
|  |  |  |  |  | Leukaemia incidence | 116 | SIR (95%CI) : 0.84 (0.69 - 1.01) RRs across radon exposure categories and the ERR indicate no statistically significant association |  |  |
|  |  |  |  |  | Non-CLL incidence | 64 | SIR (95%CI) : 0.90 (0.69 - 1.15) The ERR indicate no statistically significant association |  |  |
|  |  |  |  |  | Chronic lymphoblastic leukaemia incidence | 38 | SIR (95%CI) : 0.67 (0.48 - 0.92) The ERR indicate no statistically significant association |  |  |
| Navaranjan et al. 2016 (61) Canada 1954 - 2007 | Cohort study | Ontario uranium miners n = 28,546 males and 413 females (were not included in the dose-response analysis given the small number) Mean age at entry in males : 28.8 in females : 27.9 | Mine-specific extrapolations and areas sampling of radon decay products were used to estimate individual exposure to radon. Mean radon in WLM (range) in males : 21.0 (0.0–875.1) in females : 0.2 (0.0–16.3) | Calendar period, attained age | Extra-thoracic airways cancer mortality | _ | ERR (95%CI) : -0.17 (-0.64 - 0.30) Relative Risks (RRs) across radon exposure categories indicate no statistically significant association | 6 | Moderate |
|  |  |  |  |  | Buccal cavity and pharyngeal cancer mortality | 53 | SMR (95%CI) : 0.75 (0.56 - 0.98) |  |  |
|  |  |  |  |  | Stomach cancer mortality | 108 | ERR (95%CI) : -0.082 (-0.61 - 0.45) Relative Risks (RRs) across radon exposure categories indicate no statistically significant association |  |  |
|  |  |  |  |  | Colorectal cancer mortality | 276 | SMR (95%CI) : 0.82 (0.72 - 0.92) |  |  |
|  |  |  |  |  | Liver cancer mortality | 38 | SMR (95%CI) : 0.87 (0.62 - 1.20) |  |  |
|  |  |  |  |  | Bone cancer mortality | <6 | SMR (95%CI) : 1.11 (0.80 - 1.49) |  |  |
|  |  |  |  |  | Connective and other soft tissue cancer mortality | 8 | SMR (95%CI) : 0.62 (0.27 - 1.22) |  |  |
|  |  |  |  |  | Malignant melanoma mortality | 21 | SMR (95%CI) : 0.34 (0.24 - 0.45) |  |  |
|  |  |  |  |  | Breast cancer mortality | <6 | SMR (95%CI) : 0.60 (0.07 - 2.16) |  |  |
|  |  |  |  |  | Testis cancer mortality | <6 | SMR (95%CI) : 0.51 (0.10 - 1.48) |  |  |
|  |  |  |  |  | Kidney cancer mortality | 53 | ERR (95%CI) : -0.60 (-1.30 - 0.11) RRs across radon exposure categories indicate no statistically significant association, except a significant decrease ≥15 WLM |  |  |
|  |  |  |  |  | Thyroid cancer mortality | 6 | SMR (95%CI) : 1.26 (0.46 - 2.74) |  |  |
|  |  |  |  |  | Leukaemia mortality | 77 | ERR (95%CI) : 0.15 (-0.62 - 0.92) RRs across radon exposure categories indicate no statistically significant association |  |  |
|  |  |  |  |  | Non-CLL mortality | 47 | SMR (95%CI) : 0.77 (0.56 -1.02) ERR suggest (95%CI): -0.80 (-0.49-2.10) |  |  |
|  |  |  |  |  | Chronic lymphoblastic leukaemia mortality | 18 | SMR (95%CI) : 0.94 (0.56 -1.48) ERR (95%CI) = -0.91 (-1.40 - -0.44) |  |  |
| Zablostska et al. 2014 (62) Canada 1969 - 1999 | Cohort study | Eldorado uramium miners (Underground, surface) n = 16,770 | Exposure period : 1932 - 1980 Mean radon exposure weithed by person-year in WLM (SD, range) : In males : 100.2 (254.4, 0–2,569.0) in females : 3.5 (9.0, 0 - 67.6) | for SIRs : Age and calendar year at risk for ERRs : Employment site, age at risk, calendar year at risk and duration of employment | All leukaemia incidence | 53 | SIR (95%CI) : 0.79 (0.59 - 1.03) | 7 | Moderate |
|  |  |  |  |  | Non-CLL incidence | 30 | ERR (95%CI) per 100 WLM : -0.04 (NE - NE) ; p = 0.489 |  |  |
|  |  |  |  |  | Chronic lymphoblastic leukaemia incidence | 22 | ERR per 100 WLM : -0.04 (NE - NE) ; p = 0.59 |  |  |
|  |  |  |  |  | Hodgkin lymphoma incidence | 14 | SIR (95%CI) : 0.93 (0.51 - 1.57) ERR (95%CI) per 100 WLM : 20.7 (<0 - 324) ; p = 0.568 |  |  |
|  |  |  |  |  | Non-Hodgkin lymphoma incidence | 80 | SIR (95%CI) : 0.89 (0.70 - 1.11) ERR (95%CI) per 100 WLM : 0.04 (<0 - 0.46) ; p = 0.676 |  |  |
|  |  |  |  |  | Lymphoma | 88 | ERR (95%CI) per 100 WLM : 0.06 (<0 - 0.52) ; p = 0.568 |  |  |
|  |  |  |  |  | Myeloma incidence | 20 | SIR (95%CI) : 0.65 (0.40 - 1.01) ERR (95%CI) per 100 WLM : 0.01 (<0 - 0.66) ; p = 0.935 |  |  |
|  |  |  |  |  | Lymphohematological cancer incidence | 160 | ERR (95%CI) per 100 WLM : -0.01 (<0 - 0.17) ; p = 0.823 |  |  |
| Zablostska et al. 2014 (62) Canada 1950 - 1999 | Cohort study | Eldorado uramium miners (Underground, surface) n = 17,660 | Exposure period : 1932 - 1980 Mean radon exposure weithed by person-year in WLM (SD, range) : In males : 100.2 (254.4, 0–2,569.0) in females : 3.5 (9.0, 0 - 67.6) | for SMRs : Age and calendar year at risk for ERRs : Employment site, age at risk, calendar year at risk and duration of employment | All leukaemia mortality | 34 | ERR (95%CI) per 100 WLM : 0.02 (<0 - 0.46) ; p = 0.808 | 7 | Moderate |
|  |  |  |  |  | Hodgkin lymphoma mortality | 7 | ERR (95%CI) per 100 WLM : 0.2 (<0 - 5.79) ; p = 0.576 |  |  |
|  |  |  |  |  | Non-Hodgkin lymphoma mortality | 42 | ERR (95%CI) per 100 WLM : -0.03 (NE) ; p = 0.768 |  |  |
|  |  |  |  |  | Lymphoma mortality | 49 | ERR (95%CI) per 100 WLM : 0 (NE) ; p = 0.9 |  |  |
|  |  |  |  |  | Multiple myeloma mortality | 18 | ERR (95%CI) per 100 WLM : 0.04 (<0 - 1.07) ; p = 0.845 |  |  |
|  |  |  |  |  | Lymphohematological cancer mortality | 101 | ERR (95%CI) per 100 WLM : 0.02 (<0 - 0.23) ; p = 0.799 |  |  |
| Lane et al. 2010 (63) Canada 1969 - 1999 | Cohort study | Eldorado uramium miners (Underground, surface) n = 16,770 | Exposure period : 1932 - 1980 Mean radon exposure weithed by person-year in WLM (SD) : In males : 100.2 (254.4) in female : 4.6 (10.1) | Age at risk, calendar year at risk and duration of employment | Buccal cavity cancer incidence | 50 | ERR per 100 WLM : -0.04 ; p = 0.68 | 7 | Moderate |
|  |  |  |  |  | Stomach cancer incidence | 69 | ERR per 100 WLM : -0.04 ; p = 0.25 |  |  |
|  |  |  |  |  | Colon cancer incidence | 118 | ERR per 100 WLM : -0.04 ; p = 0.4 |  |  |
|  |  |  |  |  | Rectum cancer incidence | 95 | ERR per 100 WLM : 0.03 ; p = 0.54 |  |  |
|  |  |  |  |  | Pancreatic cancer incidence | 59 | ERR per 100 WLM : -0.03 ; p = 0.74 |  |  |
|  |  |  |  |  | Prostate cancer incidence | 350 | ERR (95%CI) per 100 WLM : -0.01 ; p = 0.77 |  |  |
|  |  |  |  |  | Bladder cancer incidence | 89 | ERR (95%CI) per 100 WLM : -0.04 ; p = 0.55 |  |  |
| Lane et al. 2010 (63) Canada 1950 - 1999 | Cohort study | Eldorado uramium miners (Underground, surface) n = 17,660 | Exposure period : 1932 - 1980 Mean radon exposure weithed by person-year in WLM (SD) : In males : 100.2 (254.4) in female : 4.6 (10.1) | Age at risk, calendar year at risk and duration of employment | Stomach cancer mortality | 75 | ERR per 100 WLM : -0.04 ; p= 0.16 | 7 | Moderate |
|  |  |  |  |  | Colon cancer mortality | 82 | ERR per 100 WLM : 0 ; p = 0.99 |  |  |
|  |  |  |  |  | Pancreatic cancer mortality | 67 | ERR per 100 WLM : -0.01 ; p = 0.84 |  |  |
|  |  |  |  |  | Prostate cancer mortality | 98 | ERR (95%CI) per 100 WLM : -0.03 ; p = 0.52 |  |  |
|  |  |  |  |  | Other cancers | 113 | ERR (95%CI) per 100 WLM : 0.06 ; p = 0.51 |  |  |
| Zablostska et al. 2013 (64) Canada 1969 - 1999 | Cohort study | Port Hope uranium miners (Surface and underground uranium miners, millers) n = 2645 males | Exposure period : 1932 - 1980 Mean cumulative radon exposure in WLM (SD, range) In males : 13.3 (45.9, 0–627.6) | For SIRs : age at risk, calendar year at risk For ERRs : age at risk, calendar year at risk and duration of employment | Lip cancer incidence | 5 | SIR (95%CI) : 0.82 (0.27 - 1.91) | 7 | Moderate |
|  |  |  |  |  | Oesophageal cancer incidence | 5 | SIR (95%CI) : 0.80 (0.26 - 1.87) |  |  |
|  |  |  |  |  | Stomach cancer incidence | 13 | SIR (95%CI) : 0.75 (0.40 - 1.28) |  |  |
|  |  |  |  |  | Colon cancer incidence | 33 | SIR (95%CI) : 0.83 (0.57 - 1.16) |  |  |
|  |  |  |  |  | Rectal cancer incidence | 22 | SIR (95%CI) : 0.92 (0.58 - 1.39) |  |  |
|  |  |  |  |  | Pancreatic cancer incidence | 10 | SIR (95%CI) : 0.81 (0.39 - 1.49) |  |  |
|  |  |  |  |  | Laryngeal cancer incidence | 11 | SIR (95%CI) : 1.15 (0.58 - 2.06) |  |  |
|  |  |  |  |  | Malignant melanoma incidence | 11 | SIR (95%CI) : 1.33 (0.67 - 2.39) |  |  |
|  |  |  |  |  | Prostate cancer incidence | 89 | SIR (95%CI) : 0.94 (0.76 - 1.16) |  |  |
|  |  |  |  |  | Kidney cancer incidence | 5 | SIR (95%CI) : 0.38 (0.12 - 0.89) |  |  |
|  |  |  |  |  | Bladder and other urinary cancer incidence | 30 | SIR (95%CI) : 1.01 (0.68 - 1.45) |  |  |
|  |  |  |  |  | Bladder cancer incidence | 26 | SIR (95%CI) : 0.91 (0.60 - 1.33) |  |  |
|  |  |  |  |  | Brain and other central nervous system cancer incidence | 9 | SIR (95%CI) : 1.21 (0.55 - 2.30) |  |  |
|  |  |  |  |  | Brain cancer incidence | 9 | SIR (95%CI) : 1.30 (0.60 - 2.47) |  |  |
| Zablostska et al. 2013 (64) Canada 1950 - 2001 | Cohort study | Port Hope uranium miners (Surface and underground uranium miners, millers) n = 2645 males | Exposure period : 1932 - 1980 Mean cumulative radon exposure in WLM (SD, range) In males : 13.3 (45.9, 0–627.6) | For SMRs : age at risk, calendar year at risk For ERRs : age at risk, calendar year at risk and duration of employment | Colon cancer mortality | 22 | SMR (95%CI) : 0.82 (0.52 - 1.25) | 7 | Moderate |
|  |  |  |  |  | Rectal cancer mortality | 15 | ERR (95%CI) per 100 WLM : 0.21 (<-0.34 - 2.31) ; p = 0.74 |  |  |
|  |  |  |  |  | Kidney cancer mortality | 6 | ERR (95%CI) per 100 WLM : -0.16 (<-0.39 - 49.51) ; p = 0.92 |  |  |
|  |  |  |  |  | Bladder cancer mortality | 10 | SMR (95%CI) : 1.28 (0.61 - 2.35) ERR (95%CI) per 100 WLM : -0.16 (<-0.39 - 49.51) ; p = 0.90 |  |  |
|  |  |  |  |  | Brain cancer mortality | 5 | SMR (95%CI) : 0.74 (0.24 - 1.73) ERR (95%CI) per 100 WLM : -0.15 (< -0.34 - 1.74) ; p = 0.90 |  |  |
|  |  |  |  |  | Lymphohematological cancer mortality | 17 | ERR (95%CI) per 100 WLM : -0.16 (< -0.34 - 14.27) ; p = 0.79 |  |  |
| Kreuzer et al. 2014 (65) Germany 1946 - 2008 (37) | Cohort study | Wismut male uranium miners (underground, open pit, surface, milling) n = 58,690 | Cumulative radon exposure in Working Level Months (WLM) was determined from a comprehensive job-exposure matrix, based on ambient measurements and detailed expert rating Exposure period : 1946 - 1989) Mean cumulative radon in the exposed group : 280 | Age, calendar year | Extra-thoracic airways cancer mortality | 234 | ERR (95%CI) per 100 WLM increase in cumulative radon exposure : 0.035 (-0.009 - 0.080) Relative Risks, RRs across cumulative radon exposure categories indicate no statistically significant association | 6 | Moderate |
|  |  |  |  |  | Tongue and mouth cancer mortality | 55 | ERR (95%CI) : 0.030 (-0.073 - 0.132) Relative Risks, RRs across cumulative radon exposure categories indicate no statistically significant association |  |  |
|  |  |  |  |  | Pharynx cancer mortality | 74 | ERR (95%CI) : 0.077 (-0.040 - 0.193) Relative Risks, RRs across cumulative radon exposure categories indicate no statistically significant association |  |  |
|  |  |  |  |  | Nose cancer mortality | 9 | ERR (95%CI) : 0.142 (-0.260 - 0.545) |  |  |
|  |  |  |  |  | Larynx cancer mortality | 94 | ERR (95%CI) : 0.017 (-0.035 - 0.070) Relative Risks, RRs across cumulative radon exposure categories indicate no statistically significant association |  |  |
| Walsh et al. 2010 (66) Germany 1946 - 2003 (34) | Cohort study | Wismut male uranium miners (underground, open pit, surface, milling) n = 58,987 Mean age at first exposure : 25 | Cumulative radon exposure in Working Level Months (WLM) was determined from a comprehensive job-exposure matrix, based on ambient measurements and detailed expert rating Exposure period : 1946 - 1990 Mean person-years weighted cumulative exposure to radon : 218 WLM | Age, calendar year | Liver cancer mortality | 159 | ERR (95%CI) per 100 WLM increase in cumulative radon exposure : 0.044 (-0.008 - 0.095) | 6 | Moderate |
|  |  |  |  |  | Non-Hodgkin lymphoma mortality | 87 | ERR : 0.032 (-0.036 - 0.101) |  |  |
|  |  |  |  |  | Rectum cancer mortality | 2074 | ERR : 0.028 (-0.008 - 0.064) |  |  |
|  |  |  |  |  | Stomach cancer mortality | 595 | ERR : 0.022 ( 0.001 - 0.042) |  |  |
|  |  |  |  |  | Gallbladder cancer mortality | 81 | ERR : 0.021 (-0.035 - 0.077) |  |  |
|  |  |  |  |  | Bladder and other urinary organs cancer mortality | 177 | ERR : 0.020 (-0.016 - 0.056) |  |  |
|  |  |  |  |  | Kidney cancer mortality | 171 | ERR : 0.017 (-0.023 - 0.058) |  |  |
|  |  |  |  |  | Colon cancer mortality | 301 | ERR : 0.016 (-0.012 - 0.045) |  |  |
|  |  |  |  |  | Extrapulmonary cancer mortality | 3355 | ERR : 0.014 ( 0.006 - 0.023) |  |  |
|  |  |  |  |  | Prostate cancer mortality | 264 | ERR : 0.000 (-0.024 - 0.024) |  |  |
|  |  |  |  |  | Pancreas cancer mortality | 229 | ERR : 0.001 (-0 029 - 0.026) |  |  |
|  |  |  |  |  | Brain and central nervous system cancer mortality | 115 | ERR : -0.018 (-0.051 - 0.018) |  |  |
|  |  |  |  |  | Esophagus cancer mortality | 126 | ERR : -0.025 (-0.053 - 0.002) |  |  |
|  |  |  |  |  | Multiple Myeloma mortality | 55 | ERR : 0.007 (-0.056 - 0.071) |  |  |
|  |  |  |  |  | Leukaemia mortality | 128 | ERR : 0.005 (-0.034 - 0.045) |  |  |
| Kreuzer et al. 2015 (67) Germany 1970 - 2008 | Cohort study | Wismut male uranium miners milling n = 4054 Mean age at first employment (range) : 25 (14–61) | Cumulative radon exposure in Working Level Months (WLM) was determined from a comprehensive job-exposure matrix, based on ambient measurements and detailed expert rating Exposure period : 1946 - 1990 Mean cumulative exposure to radon among exposed miners in WLM (range) : 280 (> 0 , 3224) | Age, sex, calendar year | Buccal & pharynx cancer mortality | 10 | SMR (95%CI) : 0.82 (0.31 - 1.33) | 6 | Moderate |
|  |  |  |  |  | Colon cancer mortality | 21 | SMR (95%CI) : 0.66 (0.38 - 0.94) |  |  |
|  |  |  |  |  | Gallbladder cancer mortality | 6 | SMR (95%CI) : 0.84 (0.17 - 1.51) |  |  |
|  |  |  |  |  | Kidney, ureter, other urinary organs cancer mortality | 11 | SMR (95%CI) : 0.59 (0.24 - 0.94) |  |  |
|  |  |  |  |  | Bladder cancer mortality | 20 | SMR (95%CI) : 1.03 (0.58 - 1.48) |  |  |
| Kreuzer et al. 2015 (67) Germany 1946 - 2008 |  |  |  |  | Stomach cancer mortality |  | RR (95%CI) across cumulative radon exposure categories in WLM 0 - 5 : 1.00 (reference) 5 - 20 : 0.94 (0.42 - 2.10) 10 - 25 : 0.94 (0.45 - 1.90) 25 - 127 : 2.20 (0.95 - 5.22) |  |  |
|  |  |  |  |  | Lymphohematological cancer mortality | 23 | ERR : 0.84 (-4.80 - 6.49) |  |  |
| Kreuzer et al. 2021 (68) Germany 1960 - 2013 | Cohort study | Wismut male underground uranium miners n = 35,204 | Cumulative radon exposure in Working Level Months (WLM) was determined from a comprehensive job-exposure matrix, based on ambient measurements and detailed expert rating Exposure period : 1946 - 1989 Mean cumulative radon exposure : 364 WLM | Age, sex, calendar year | Buccal & pharynx cancer mortality | 117 | SMR (95%CI) : 0.97 (0.80 - 1.16) | 6 | Moderate |
|  |  |  |  |  | Stomach cancer mortality | 504 | SMRs (95%CI) across cumulative radon exposure categories < 10 : 1.24 (0.72 - 1.98) 10 - 99 : 1.48 (1.20 - 1.80) 100 - 499 : 1.18 (1.00 - 1.38) 500 – 999 : 1.18 (0.98 - 1.42) ≥ 1000 : 1.43 (1.18 - 1.73) p for linear trend = 0.579 |  |  |
|  |  |  |  |  | Liver cancer mortality | 175 | SMR (95%CI) : 1.34 (1.15 - 1.55) SMRs across cumulative radon exposure categories shown a significant excess mortality from ≥ 500 WLM ; p-trend = 0.020 |  |  |
|  |  |  |  |  | Gallbladder cancer mortality | 62 | SMR (95%CI) : 0.97 (0.75 - 1.25) |  |  |
|  |  |  |  |  | Nose cancer mortality | 9 | SMR (95%CI) : 1.35 (0.62 - 2.56) |  |  |
|  |  |  |  |  | Bone cancer mortality | 9 | SMR (95%CI) : 0.61 (0.28 - 1.16) |  |  |
|  |  |  |  |  | Malignant melanoma mortality | 43 | SMR (95%CI) : 0.97 (0.71 - 1.31) |  |  |
|  |  |  |  |  | Non-malanoma skin cancer | 8 | SMR (95%CI) : 0.69 (0.30 - 1.37) |  |  |
|  |  |  |  |  | Connective tissue cancer mortality | 14 | SMR (95%CI) : 0.74 (0.40 - 1.23) |  |  |
|  |  |  |  |  | Testis cancer mortality | 19 | SMR (95%CI) : 0.81 (0.49 - 1.26) |  |  |
|  |  |  |  |  | Kidney, ureter, other urinary organs cancer mortality | 174 | SMR (95%CI) : 1.01 (0.87 - 1.18) |  |  |
|  |  |  |  |  | Bladder cancer mortality | 172 | SMR (95%CI) : 1.03 (0.88 - 1.19) |  |  |
|  |  |  |  |  | Thyroid gland cancer mortality | 11 | SMR (95%CI) : 0.93 (0.47 - 1.67) |  |  |
|  |  |  |  |  | Non-CLL mortality | 84 | SMR (95%CI) : 0.88 (0.70 - 1.09) |  |  |
|  |  |  |  |  | Myeloid leukaemia mortality | 66 | SMR (95%CI) : 0.96 (0.74 - 1.22) |  |  |
|  |  |  |  |  | Cancer excluding lung mortality | 3153 | SMR (95%CI) : 1.06 (1.02 - 1.09) SMRs across cumulative radon exposure categories shown a significant excess mortality only for ≥ 1000 WLM ; p-trend = 0.002 |  |  |
| Mohner et al. 2006 (69) Germany 1950 - 1989 | Nested case-control study | Wismut male uranium miners (underground, open pit, surface, milling) n = 1357 Mean age at hire (range) : in cases : 30.8 (16 -67) in controls : 30.7 (14 - 63) | Cumulative radon exposure in Working Level Months (WLM) was determined from a comprehensive job-exposure matrix, based on ambient measurements and detailed expert rating. Mean cumulative radon : 275.7 WLM. | Year of birth, attained age | All leukaemia incidence | 377/980 | ERR (90%CI) : -0.007 (-0.039 - 0.026) OR (90%CI) across cumulative radon exposure categories indicate no statistically significant association | 6 | Moderate |
|  |  |  |  |  | Chronic lymphocytic leukaemia incidence | 159/406 | ERR (90%CI) : -0.007 (-0.039 - 0.026) ORs across cumulative radon exposure categories indicate no statistically significant association |  |  |
|  |  |  |  |  | Acute myeloid leukaemia incidence | 73/189 | ERR (90%CI) : -0.006 (-0.052 - 0.040) ORs across cumulative radon exposure categories indicate no statistically significant association, except a dicrease risk when exposure between ≥100 - 499 |  |  |
|  |  |  |  |  | Non-CLL incidence | 218/574 | ERR (90%CI) : -0.006 (-0.035 - 0.023) ORs across cumulative radon exposure categories indicate no statistically significant association |  |  |
| Mohner et al. 2008 (70) Germany 1950 - 1989 | Nested case-control study | Wismut male uranium miners (underground, open pit, surface, milling) n = 1483 Mean age at hire (range) : in cases : 30.8 (16 -67) in controls : 30.7 (14 - 63) | Cumulative radon exposure in Working Level Months (WLM) was determined from a comprehensive job-exposure matrix, based on ambient measurements and detailed expert rating. Mean cumulative radon : 275.7 WLM. | Year of birth, attained age, smoking (Never smoked, Smoked at some time, Not stated), alcohol consumption | Laryngeal cancver incidence | 554/929 | ORs (95%CI) across cumulative radon exposure categories in WLM < 50 : 1.00 (reference) 50− <100 : 1.21 (0.82−1.78) 100−<500 : 1.11 (0.84−1.45) 500−<1000 : 0.97 (0.67−1.40) ≥1000 : 1.13 (0.74−1.72) | 5 | Moderate |
|  |  |  |  |  |  |  |  |  |  |
|  |  |  |  |  |  |  |  |  |  |
|  |  |  |  |  |  |  |  |  |  |
| Zablotska et al. 2018 (71) Canada & Germany 1946 - 2008 in Wismut cohort 1950 - 1999 in Port Hope cohort | Pooled cohort study | Uranium processing workers with no mining experience (Port hope and Wismut millers) n = 7431 (625 females, and 6806 male). Average age at start of employement (SD) : 29 (10) to 30 (11) | Exposure period : 1942 - 1996 across studies Mean cumulative RDP exposure among males : 16.6 WLM (SD = 49.8) Exposure period : 1932 - 1990 across cohorts | Age at risk, cohort, duration of employment | Solid cancer excluding lung cancer mortality | 407 | ERR (95%CI) per 100 WLM ; p_value in males : 0.09 (< -0.19 - 0.76) ; p = 0.63 in females : 2.60 (< -2.08 - 13.9) ; p = 0.29 | 8 | Moderate |
|  |  |  |  |  | Breast cancer mortality | 7 | ERR (95%CI) per 100 WLM ; p_value in females : 5.37 (<−10.3 - 281) ; p = 0.53 |  |  |
| Rage et al. 2018 (72) France 1946 - 2007 (34.7) | Cohort study | French male uranium miners n = 5400 Mean age at enrollment (min - max) into the cohort : 29.0 (16.0–68.5) Mean age at exit (min - max) : 63.6 (18.0–85.1) | Retrospective expert radon exposure reconstruction, worksite ambiante radon gas concentration measurement, measurement through individual dosimeters. Exposure period : in the main cohort : 1946 - 1990 in the 1977 post-JOUAC cohort : 1957 - 2001 Mean radon (se) in WLM : 35.1 (69.9) ; max = 960.1 WLM | For SMRs : Calendar period, attained age, sex, duration of employment, cumulative radon exposur For ERR : calendar year and attained age | All cancers excluding lung cancer mortality | 513 | SMR (95%CI) : 1.04 (0.96 -1.14) ERR per 100 WLM increase in cumulation radon exposure : -0.006 ; p >0.05 | 6 | Moderate |
|  |  |  |  |  | All cancers excluding lung and kidney cancer mortality | 489 | SMR (95%CI) : 1.03 (0.94 - 1.12) |  |  |
|  |  |  |  |  | Buccal cavity and pharynx cancer mortality | 42 | SMR (95%CI) : 0.94 (0.68 - 1.27) |  |  |
|  |  |  |  |  | Intestine, colon, and rectum cancer mortality | 63 | SMR (95%CI) : 1.05 (0.80 - 1.34) |  |  |
|  |  |  |  |  | Liver cancer mortality | 32 | SMR (95%CI) : 1.15 (0.78 - 1.62) |  |  |
|  |  |  |  |  | Nose cancer mortality | 13 | SMR (95%CI) : 1.19 (0.63 - 2.03) |  |  |
|  |  |  |  |  | Brain and central nervous system cancer mortality | 28 | ERR per 100 WLM increase : -0.12 ; p=0.35 |  |  |
|  |  |  |  |  | Bladder cancer mortality | 25 | SMR (95%CI) : 1.19 (0.77 - 1.75) |  |  |
|  |  |  |  |  | Kidney, ureter and other organs cancer mortality | 24 | SMR (95%CI) : 1.58 (1.01 - 2.35) ERR per 100 WLM increase : 0.27 ; p>0.05 |  |  |
|  |  |  |  |  | Lymphohaematological cancer mortality excluding leukaemia | 23 | SMR (95%CI) : 1.02 (0.64 - 1.53) |  |  |
|  |  |  |  |  | Non-CLL mortality | 21 | SMR (95%CI) : 1.51 (0.94 - 2.31) |  |  |
| Drubay et al. 2014 (73) France 1956 - 2007 | Cohort study | French post-1955 subcohort of male uranium miners n = 3377 Median age at end of follow-up (range) : 58.6 (19.5 - 85.0) | Worksite ambiante radon gas concentration measurement, and measurement through individual dosimeters. Exposure period : 1956 - 1990 Median (range) cumulative radon exposure in WLM : 4.7 (0 - 128.4) | Age (for HR); age and sex (for SMR) | Kidney, ureter, and other urinary organs cancer mortality | 11 | HR (95%CI) per 100 WLM increase : 0.805 (0.074 - 8.746) | 8 | High |
|  |  |  |  |  |  |  |  |  |  |
|  |  |  |  |  |  |  |  |  |  |
| Drubay et al. 2014 (73) Germany 1946 - 2003 | Cohort study | Wismut male uranium miners (underground, open pit, surface, milling) n = 58,986 Median age at end of follow-up (range) : 60.4 (15.3 - 103.2) | Cumulative radon exposure in Working Level Months (WLM) was determined from a comprehensive job-exposure matrix, based on ambient measurements and detailed expert rating. Exposure period : 1946 - 1989 Median (range) cumulative radon exposure in WLM : 18.4 (0 - 3,224.5) | Age (for HR); age and sex (for SMR) | Kidney, ureter, and other urinary organs cancer mortality | 174 | HR (95%CI) per 100 WLM increase : 1.023 (0.993 -1.053) | 8 | High |
|  |  |  |  |  |  |  |  |  |  |
|  |  |  |  |  |  |  |  |  |  |
|  |  |  |  |  |  |  |  |  |  |
| Richardson et al. 2021 (74) USA & Canada & France & Germany & Czech Republic 1946 - 2013 across studies | Pooled cohort study | Uranium miners (open-pit miners, underground miners and surface workers) n = 118,329 males | Exposure period : 1942 - 1996 across studies | Age, calendar-year, (and race for USA cohorts only) | Buccal cancer mortality | 161 | SMR (95%CI) : 0.77 (0.66 - 0.90) | 9 | High |
|  |  |  |  |  | Pharynx cancer mortality | 175 | SMR (95%CI) : 0.83 (0.71 - 0.96) |  |  |
|  |  |  |  |  | Oesophagus cancer mortality | 351 | SMR (95%CI) : 0.92 (0.83 - 1.03) |  |  |
|  |  |  |  |  | Stomach cancer mortality | 1058 | SMR (95%CI) : 1.08 (1.02 - 1.15) |  |  |
|  |  |  |  |  | Intestine (small intestine + colon) cancer mortality | 919 | SMR (95%CI) : 0.89 (0.83 - 0.95) |  |  |
|  |  |  |  |  | Rectum cancer mortality | 554 | SMR (95%CI) : 0.96 (0.89 - 1.05) |  |  |
|  |  |  |  |  | Liver and gallbladder cancer mortality | 549 | SMR (95%CI) : 1.15 (1.06 - 1.25) |  |  |
|  |  |  |  |  | Pancreas cancer mortality | 641 | SMR (95%CI) : 0.96 (0.89 - 1.04) |  |  |
|  |  |  |  |  | Larynx cancer mortality | 229 | SMR (95%CI) : 1.10 (0.97 - 1.26) |  |  |
|  |  |  |  |  | Prostate cancer mortality | 857 | SMR (95%CI) : 0.84 (0.79 - 0.90) |  |  |
|  |  |  |  |  | Kidney cancer mortality | 392 | SMR (95%CI) : 0.96 (0.87 - 1.06) |  |  |
|  |  |  |  |  | Bladder and other urinary cancer mortality | 421 | SMR (95%CI) : 0.85 (0.77 - 0.94) |  |  |
|  |  |  |  |  | Skin cancer mortality | 133 | SMR (95%CI) : 0.86 (0.72 - 1.02) |  |  |
|  |  |  |  |  | Brain and central nervous system cancer mortality | 298 | SMR (95%CI) : 0.87 (0.77 - 0.97) |  |  |
|  |  |  |  |  | Hodgkin lymphoma mortality | 65 | SMR (95%CI) : 0.90 (0.70 - 1.15) |  |  |
|  |  |  |  |  | Non-Hodgkin lymphoma mortality | 321 | SMR (95%CI) : 0.92 (0.83 - 1.03) |  |  |
|  |  |  |  |  | Multiple myeloma mortality | 161 | SMR (95%CI) : 0.88 (0.75 - 1.03) |  |  |
|  |  |  |  |  | Leukaemia mortality | 396 | SMR (95%CI) : 0.93 (0.84 - 1.03) |  |  |
| Silver et al. 2013 (75) USA 1951 -2004 (37) | Cohort study | Fernald Feed Materials Production Center workers n = 6409 Mean age at first hire : 30.1 | Exposure period : 1951 - 1985 | Gender, race, age, calendar period, pay code (hourly/salaried) and birth year (spline terms) and consider all radiologic exposures simultaneously, non-radiological exposure (occupational exposure to acids) | Stomach cancer mortality | 32 | SMR (95%CI) : in hourly males : 1.15 (0.68 - 1.82) in salaried males : 1.77 (0.91 - 3.09) in hourly females : 0.00 (0.00 - 13.2) in salaried females : 1.93 0.23 - 6.96) | 8 | High |
|  |  |  |  |  | Intestine cancer mortality | 70 | SMR (95%CI) : in hourly males : 1.16 (0.86 - 1.53) in salaried males : 0.71 (0.39 - 1.18) in hourly females : 1.66 (0.20 - 6.00) in salaried females : 0.82 (0.22 - 2.09) |  |  |
|  |  |  |  |  | Kidney cancer mortality | 18 | SMR (95%CI) in hourly males : 0.95 (0.49 - 1.66) in salaried males : 0.49 (0.10 - 1.44) in hourly females : 0.00 (0.00 - 18.2) in salaried males : 2.99 (0.62 - 8.75) |  |  |
|  |  |  |  |  | Bladder and other urinary organs cancer mortality | 24 | SMR (95%CI) in hourly males : 1.15 (0.64 - 1.89) in salaried males : 1.00 (0.37 - 2.17) in hourly females : 0.00 0.00 - 23.7) in salaried females : 5.13 (1.06 - 15.0) |  |  |
|  |  |  |  |  | Non-Hodgkin lymphoma mortality | 32 | SMR (95%CI) in hourly males : 0.97 (0.58 - 1.54) in salaried males : 1.33 (0.69 - 2.32) in hourly females : 4.41 (0.53 - 16.0) in salaried females : 0.00 (0.00 - 1.71) |  |  |
|  |  |  |  |  | Hodgkin lymphoma mortality | 6 | SMR (95%CI) in hourly males: 1.81 (0.59 - 4.22) in salaried males : 0.76 (0.02 - 4.23) in hourly females : 0.00 (0.00 - 70.1) in salaried females : 0.00 (0.00 - 13.0) |  |  |
|  |  |  |  |  | Leukaemia cancer mortality | 35 | SMR (95%CI) in hourly males : 0.92 (0.54 - 1.48) in salaried males : 1.71 (0.95 - 2.81) in hourly females : 0.00 (0.00 - 9.23) in salaried females : 1.61 (0.33 - 4.70) |  |  |
|  |  |  |  |  | Multiple myeloma mortality | 19 | SMR (95%CI) in hourly males : 1.44 (0.75 - 2.52) in salaried males : 1.82 (0.73 - 3.75) in hourly females : 0.00 (0.00 - 17.7) in salaried females : 0 0.00 (0.00 - 3.96) |  |  |
| Boice et al. 2008 (76) USA 1979 - 2005 | Cohort study | Uranium miners or millers with mining experience n = 2745 (2500 males, 245 females) | Exposure period : 1942 - 1996 across studies | Sex, age, and calendar year | Buccal and pharyngal cancer mortality | 2 | SMR (95%CI) : 0.48 (0.06 -1.73) | 8 | Moderate |
|  |  |  |  |  | Colon cancer mortality | 11 | SMR (95%CI) : 0.67 (0.33 - 1.19) |  |  |
|  |  |  |  |  | Breast cancer mortality | 2 | SMR (95%CI) : 0.90 (0.11 - 3.25) |  |  |
|  |  |  |  |  | All uterine cancer mortality | 0 | SMR (95%CI) : 0.00 (0.00 - 8.35) |  |  |
|  |  |  |  |  | Other female genital organs mortality | 2 | SMR (95%CI) : 3.17 (0.38 - 11.5) |  |  |
|  |  |  |  |  | Malignant melanoma mortality | 6 | SMR (95%CI) : 1.57 (0.57 - 3.41) |  |  |
|  |  |  |  |  | Thyroid and other endocrine glands cancer mortality | 1 | SMR (95%CI) : 1.71 (0.04 - 9.52) |  |  |
|  |  |  |  |  | Bone cancer mortality | 0 | SMR (95%CI) : 0.00 (0.00 - 9.87) |  |  |
|  |  |  |  |  | All lymphohematological cancer mortality | 23 | SMR (95%CI) : 1.18 (0.75 - 1.77) |  |  |
|  |  |  |  |  | Chronic lymphoblastic leukaemia mortality | 4 | SMR (95%CI) : 2.65 (0.72 - 6.79) |  |  |
|  |  |  |  |  | Non-CLL mortality | 8 | SMR (95%CI) : 1.36 (0.59 - 2.68) |  |  |
| Veiga et al. 2006 (77) Brazil 1942 - 1997 | Cohort study | Coal miners (underground, surface workers) n = 2856 | Exposure period : 1942 -1997 | Age, calendar period, sex | Oesophagus cancer mortality | 4 | SMR (95%CI) : 0.55 (0.21 - 1.48) | 7 | Moderate |
|  |  |  |  |  | Stomach cancer mortality | 11 | SMR (95%CI) : 0.92 (0.51 - 1.66) |  |  |
|  |  |  |  |  | Colorectal cancer mortality | 1 | SMR (95%CI) : 0.23 (0.03 - 1.65) |  |  |
|  |  |  |  |  | Larynx cancer mortality | 1 | SMR (95%CI) : 0.34 (0.48 - 2.45) |  |  |
|  |  |  |  |  | Prostate cancer mortality | 2 | SMR (95%CI) : 0.39 (0.09 - 1.55) |  |  |
|  |  |  |  |  | Leukaemia cancer mortality | 1 | SMR (95%CI) : 0.39 (0.05 - 2.78) |  |  |
| Darby et al. 1995 (78) Sweden 1951 - 1990 | Cohort study | Underground iron miners n = 1294 | Exposure period : 1897 - 1976 | calendar year | Tongue and month cancer mortality | 0 | SMR (95%CI) : 0.00 (0.00 - 6.36) | 5 | Moderate |
|  |  |  |  |  | Salivary gland cancer mortality | 0 | SMR (95%CI) : 0.00 (0.00 - 23.06) |  |  |
|  |  |  |  |  | Pharynx cancer mortality | 0 | SMR (95%CI) : 0.00 (0.00 - 3.02) |  |  |
|  |  |  |  |  | Esophagus cancer mortality | 4 | SMR (95%CI) : 1.36 (0.37 - 3.47) |  |  |
|  |  |  |  |  | Stomach cancer mortality | 40 | SMR (95%CI) : 1.45 (1.04 - 1.98) SMRs across cumulative radon categories indicate a non-significant (+) linear trend |  |  |
|  |  |  |  |  | Intestine cancer mortality | 9 | SMR (95%CI) : 0.88 (0.40 - 1.67) |  |  |
|  |  |  |  |  | Rectum cancer mortality | 13 | SMR (95%CI) : 1.94 (1.03 - 3.31) SMRs across cumulative radon categories indicate a non-significant (-) linear trend |  |  |
|  |  |  |  |  | Liver cancer mortality | 8 | SMR (95%CI) : 1.93 (0.83 - 3.81) |  |  |
|  |  |  |  |  | Gallbladder cancer mortality | 3 | SMR (95%CI) : 1.41 (0.29 - 4.12) SMRs across cumulative radon categories indicate a non-significant (+) linear trend |  |  |
|  |  |  |  |  | Pancreas cancer mortality | 7 | SMR (95%CI) : 0.59 (0.24 - 1.21) |  |  |
|  |  |  |  |  | Nose cancer mortality | 0 | SMR (95%CI) : 0.00 (0.00 - 9.46) |  |  |
|  |  |  |  |  | Larynx cancer mortality | 0 | SMR (95%CI) : 0.00 (0.00 - 6.47) |  |  |
|  |  |  |  |  | Bone cancer mortality | 0 | SMR (95%CI) : 0.00 (0.00 - 4.19) |  |  |
|  |  |  |  |  | Connective and other soft tissue cancer mortality | 0 | SMR (95%CI) : 0.00 (0.00 - 7.53) |  |  |
|  |  |  |  |  | Malignant melanoma mortality | 0 | SMR (95%CI) : 0.00 (0.00 - 2.82) SMRs across cumulative radon categories indicate a non-significant (+) linear trend |  |  |
|  |  |  |  |  | Non-melanoma skin cancer mortality | 2 | SMR (95%CI) : 7.14 (0.87 - 25.80) |  |  |
|  |  |  |  |  | Prostate cancer mortality | 29 | SMR (95%CI) : 1.20 (0.81 - 1.73) |  |  |
|  |  |  |  |  | Testis cancer mortality | 0 | SMR (95%CI) : 0.00 (0.00 - 19.42) |  |  |
|  |  |  |  |  | Bladder cancer mortality | 5 | SMR (95%CI) : 0.98 (0.32 - 2.29) |  |  |
|  |  |  |  |  | Kidney cancer mortality | 6 | SMR (95%CI) : 0.85 (0.31 - 1.85) |  |  |
|  |  |  |  |  | Brain and central nervous system cancer mortality | 7 | SMR (95%CI) : 2.22 (0.89 - 4.58) |  |  |
|  |  |  |  |  | Thyroid cancer mortality | 0 | SMR (95%CI) : 0.00 (0.00 - 4.50) |  |  |
|  |  |  |  |  | Non-Hodgkin lymphoma | 3 | SMR (95%CI) : 0.76 (0.16 - 2.22) |  |  |
|  |  |  |  |  | Hodgkin lymphoma mortality | 4 | SMR (95%CI) : 2.70 (0.74 - 6.92) |  |  |
|  |  |  |  |  | Multiple myeloma mortality | 7 | SMR (95%CI) : 1.92 (0.77 - 3.96) |  |  |
|  |  |  |  |  | Leukaemia mortality | 6 | SMR (95%CI) : 1.05 (0.39 - 2.30) SMRs across cumulative radon categories indicate a non-significant (-) linear trend |  |  |
|  |  |  |  |  | Other and unspecified sites cancer mortality | 9 | SMR (95%CI) : 1.22 (0.56 - 2.32) |  |  |
|  |  |  |  |  | All cancers other than lung mortality | 162 | SMR (95%CI) : 1.21 (1.03 - 1.41) SMRs across cumulative radon categories indicate a non-significant (+) linear trend |  |  |
| Darby et al. 1995 (79) USA & Canada & France & Germany & England & Czech Republic & China | Pooled cohort study | Underground iron miners n = 64,209 | Average cumulative radon = 155 WLM | Age, calendar year | Primary liver cancer mortality | 50 | SMR (95%CI) : 1.73 (1.29 - 2.28) | 7 | Moderate |
|  |  |  |  |  | Unspecified liver cancer mortality | 3 | SMR (95%CI) : 0.43 (0.09 - 1.26) |  |  |
|  |  |  |  |  | Acute myeloid leukaemia | 12 | SMR (95%CI) : 1.16 (0.60 - 2.02) |  |  |
|  |  |  |  |  | Other and unspecified leukaemia mortality | 118 | SMR (95%CI) : 1.12 (0.93 - 1.35) |  |  |
|  |  |  |  |  | All cancers other than lung mortality | 1179 | SMR (95%CI) : 1.01 (0.95 - 1.07) |  |  |
| Cocco et al. 1994 (80) Italy 1960 - 1988 | Cohort study | Sardinian and zinc miners n = 4740 | Exposure period : 1932 - 1971 | Age, calendar year | Buccal cavity and pharynx cancer mortality | 8 | SMR (95%CI) : 0.61 (0.26 - 1.21) | 7 | Moderate |
|  |  |  |  |  | Digestive system cancer mortality | 86 | SMR (95%CI) : 0.83 (0.66 - 1.03) |  |  |
|  |  |  |  |  | Oesophagus cancer mortality | 6 | SMR (95%CI) : 0.89 (0.32 - 1.93) |  |  |
|  |  |  |  |  | Stomach cancer mortality | 27 | SMR (95%CI) : 0.94 (0.62 - 1.37) |  |  |
|  |  |  |  |  | Intestine and rectum cancer mortality | 12 | SMR (95%CI) : 0.64 (0.33 - 1.12) |  |  |
|  |  |  |  |  | Liver cancer mortality | 16 | SMR (95%CI) : 0.62 (0.35 - 1.26) |  |  |
|  |  |  |  |  | Pancreas cancer mortality | 10 | SMR (95%CI) : 0.68 (0.33 - 1.25) |  |  |
|  |  |  |  |  | Peritoneum and retroperitoneum cancer mortality | 6 | SMR (95%CI) : 3.67 (1.35 - 7.98) |  |  |
|  |  |  |  |  | Larynx cancer mortality | 11 | SMR (95%CI) : 0.80 (0.40 - 1.44) |  |  |
|  |  |  |  |  | Prostate cancer mortality | 16 | SMR (95%CI) : 1.21 (0.69 - 1.97) |  |  |
|  |  |  |  |  | Bladder cancer mortality | 17 | SMR (95%CI) : 1.15 (0.67 - 1.84) |  |  |
|  |  |  |  |  | Kidney, ureter, other urinary organs cancer mortality | 7 | SMR (95%CI) : 1.28 (0.52 - 2.64) |  |  |
|  |  |  |  |  | Brain and central nervous system cancer mortality | 8 | SMR (95%CI) : 1.17 (0.50 - 2.30) |  |  |
|  |  |  |  |  | Lymphohaematological cancer mortality | 21 | SMR (95%CI) : 0.91 (0.56 - 1.39) |  |  |
|  |  |  |  |  | Non-Hodgkin lymphoma mortality | 6 | SMR (95%CI) : 1.16 (0.42 - 2.52) |  |  |
| Chen et al. 1990 (81) China 1970 - 1982 | Cohort study | Haematite mine workers n = 6444 males | _ | Age | Stomach cancer mortality | 18 | SMR (95%CI) : 0.8 (0.5 - 1.3) | 7 | Moderate |
|  |  |  |  |  | Liver cancer mortality | 17 | SMR (95%CI) : 0.8 (0.4 - 1.2) |  |  |
|  |  |  |  |  | Oesphagus cancer mortality | 11 | SMR (95%CI) : 0.6 (0.3 - 1.1) |  |  |
| Golden et al. 2019 (82) USA 1942 - 2012 (43.3) | Cohort study | Uranium processing workers n = 2514 white males Mean age at start of follow-up = 30.3 (range 16.1–65.3). | Exposure period : 1942 - 1966 | Age, sex, calendar year, race | Buccal and pharyngal cancer mortality | 8 | SMR (95%CI) : 0.76 (0.33 - 1.49) | 8 | High |
|  |  |  |  |  | Esophagus cancer mortality | 15 | SMR (95%CI) : 1.09 (0.61 - 1.80) |  |  |
|  |  |  |  |  | Stomach cancer mortality | 7 | SMR (95%CI) : 0.47 (0.19 - 0.97) |  |  |
|  |  |  |  |  | Colon cancer mortality | 46 | SMR (95%CI) : 1.09 (0.80 - 1.45) |  |  |
|  |  |  |  |  | Rectum cancer mortality | 8 | SMR (95%CI) : 0.89 (0.38 - 1.76) |  |  |
|  |  |  |  |  | Liver and gallbladder cancer mortality | 6 | SMR (95%CI) : 0.89 (0.38 - 1.76) |  |  |
|  |  |  |  |  | Pancreatic cancer mortality | 29 | SMR (95%CI) : 1.13 (0.76 - 1.62) |  |  |
|  |  |  |  |  | Larynx cancer mortality | 5 | SMR (95%CI) : 0.87 (0.28 - 2.04) |  |  |
|  |  |  |  |  | Breast cancer mortality | _ | SMR (95%CI) : 0.00 (0.00 - 5.90) |  |  |
|  |  |  |  |  | Prostate cancer mortality | 48 | SMR (95%CI) : 0.99 (0.73 - 1.32) |  |  |
|  |  |  |  |  | Testis & other male genital organs cancer mortality | 1 | SMR (95%CI) : 0.76 (0.01 - 4.23) |  |  |
|  |  |  |  |  | Kidney cancer mortality | 13 | SMR (95%CI) : 1.04 (0.55 - 1.77) |  |  |
|  |  |  |  |  | Bladder & Other Urinary organs cancer mortality | 12 | SMR (95%CI) : 0.75 (0.39 - 1.31) |  |  |
|  |  |  |  |  | Malignant melanoma mortality | 10 | SMR (95%CI) : 1.28 (0.61 - 2.36) |  |  |
|  |  |  |  |  | Brain and central nervous system cancer mortality | 22 | SMR (95%CI) : 1.85 (1.16 - 2.80) |  |  |
|  |  |  |  |  | Thyroid & Other Endocrine Glands cancer mortality | 1 | SMR (95%CI) : 0.70 (0.01 - 3.89) |  |  |
|  |  |  |  |  | Bone cancer mortality | 2 | SMR (95%CI) : 1.75 (0.20 - 6.32) |  |  |
|  |  |  |  |  | Connective & Other Soft Tissue cancer mortality | 3 | SMR (95%CI) : 1.23 (0.25 - 3.59) |  |  |
|  |  |  |  |  | Lymphohematological tissue cancer mortality | 57 | SMR (95%CI) : 1.14 (0.86 - 1.47) |  |  |
|  |  |  |  |  | Non-Hodgkin lymphoma mortality | 25 | SMR (95%CI) : 1.34 (0.87 - 1.98) |  |  |
|  |  |  |  |  | Hodgkin lymphoma mortality | 2 | SMR (95%CI) : 0.78 (0.09 - 2.81) |  |  |
|  |  |  |  |  | All leukaemia mortality | 23 | SMR (95%CI) : 1.15 (0.73 - 1.73) |  |  |
|  |  |  |  |  | Chronic lymphoblastic leukaemia mortality | 5 | SMR (95%CI) : 1.07 (0.35 - 2.50) |  |  |
|  |  |  |  |  | Non-CLL mortality | 18 | SMR (95%CI) : 1.17 (0.69 - 1.85) |  |  |
|  |  |  |  |  | Multiple myeloma mortality | 6 | SMR (95%CI) : 0.71 (0.26 - 1.54) |  |  |
| Tomasek et al. 1994 (83) Czech Republic 1948 - 1991 | Cohort study | Underground uranium miners in West Bohemia n = 4320 Mean age at start of follow-up = 30.3 (range 16.1–65.3). | Exposure period : 1948 - 1959 Mean cumulative radon exposure : 219 WLM | Age | Cancers other than lung cancer mortality | 292 | SMR : 1.11 ; p > 0.05 | 7 | Moderate |
| Hodgson et al. 1990 (84) UK 1941 - 1986 | Cohort study | Tin miners (underground, surface, intermediate), mortality n = 3010 | Exposure period : 1941 - 1984 | Age, calendar period | Stomach cancer mortality | 27 | SMR : 1.41 ; p > 0.05 | 6 | Moderate |
|  |  |  |  |  | Leukaemia mortality | 7 | SMR : 1.73 ; p > 0.05 |  |  |
| Xiang-Zhen et al. 1993 (85) China 1976 - 1987 | Cohort study | Tin miners n = 17,143 males and 2,795 females | Mean radon exposure among exposed miners In males : 275.4 WLM In females : 66.4 | Age, sex | Naso-pharyngeal cancer mortality | 17 | Relative risks across radon exposure tertiles indicate apparently increasing linear trend | 6 | Moderate |
|  |  |  |  |  | Esophagus cancer mortality | 8 | Relative risks across radon exposure tertiles indicate apparently increasing linear trend |  |  |
|  |  |  |  |  | Stomach cancer mortality | 32 | No apparent linear trend observed |  |  |
|  |  |  |  |  | Liver cancer mortality | 45 | No apparent linear trend observed |  |  |
|  |  |  |  |  | Rectum cancer mortality | 13 | No apparent linear trend observed |  |  |
|  |  |  |  |  | Leukaemia mortality | 12 | No apparent linear trend observed |  |  |
|  |  |  |  |  | Colon cancer mortality | 15 | Relative risks across radon exposure tertiles indicate apparently decreasing linear trend |  |  |
|  |  |  |  |  | Bladder cancer mortality | 11 | Relative risks across radon exposure tertiles indicate apparently increasing linear trend |  |  |
|  |  |  |  |  | Lymphoma mortality | 5 | No apparent linear trend observed |  |  |
|  |  |  |  |  | Brain cancer mortality | 6 | No apparent linear trend observed |  |  |

CI: Confidence interval; TWA: Time-weighted average; OR: Odds ratio; Bq/m^3^ : Becquerel per cubic meter; pCi/l: picoCurie/liter; HR: Hazard ratio; SES: Socio-economic status; RBM: Red bone marrow; IQR: Interquartile range; AML: Acute myeloid leukaemia; SIR: Standardize incidence ratio; IRR: Incidence rate ratio; SMR: Standardize mortality ratio; SD: Standard deviation; GP: general population; CrI: Credibility interval; BMI: Body mass index; Non-CLL: Leukaemia, excluding chronic lymphoblastic leukaemia; SRR: Standardized rate ratio; WLM: Working level month; ERR: excess relative risk

References

1. Peckham EC, Scheurer ME, Danysh HE, Lubega J, Langlois PH, Lupo PJ. Residential Radon Exposure and Incidence of Childhood Lymphoma in Texas, 1995-2011. Int J Environ Res Public Health. 25 sept 2015;12(10):12110‑26.

2. Steinbuch M, Weinberg CR, Buckley JD, Robison LL, Sandler DP. Indoor residential radon exposure and risk of childhood acute myeloid leukaemia. Br J Cancer. nov 1999;81(5):900‑6.

3. Lubin JH, Linet MS, Boice JD, Buckley J, Conrath SM, Hatch EE, et al. Case-control study of childhood acute lymphoblastic leukemia and residential radon exposure. J Natl Cancer Inst. 18 févr 1998;90(4):294‑300.

4. Collman GW, Loomis DP, Sandler DP. Childhood cancer mortality and radon concentration in drinking water in North Carolina. Br J Cancer. avr 1991;63(4):626‑9.

5. Hauri D, Spycher B, Huss A, Zimmermann F, Grotzer M, von der Weid N, et al. Domestic Radon Exposure and Risk of Childhood Cancer: A Prospective Census-Based Cohort Study. Environ Health Perspect. 1 oct 2013;121(10):1239‑44.

6. Kohli S, Noorlind B, Lofman O. Childhood leukaemia in areas with different radon levels: a spatial and temporal analysis using GIS. J Epidemiol Community Health. nov 2000;54(11):822‑6.

7. Kaletsch U, Kaatsch P, Meinert R, Schüz J, Czarwinski R, Michaelis J. Childhood cancer and residential radon exposure – results of a population-based case-control study in Lower Saxony (Germany). Radiat Environ Biophys. 1 sept 1999;38(3):211‑5.

8. Kendall GM, Little MP, Wakeford R, Bunch KJ, Miles JCH, Vincent TJ, et al. A record-based case-control study of natural background radiation and the incidence of childhood leukaemia and other cancers in Great Britain during 1980–2006. Leukemia. janv 2013;27(1):3‑9.

9. Thorne R, Foreman NK, Mott MG. Radon in Devon and Cornwall and paediatric malignancies. European Journal of Cancer. 1 févr 1996;32(2):282‑5.

10. Foreman NK, Thorne R, Berry PJ, Oakhill A, Mott MG. Childhood malignancies in the south-west region of England, 1976-1985. Med Pediatr Oncol. 1994;23(1):14‑9.

11. Nikkilä A, Arvela H, Mehtonen J, Raitanen J, Heinäniemi M, Lohi O, et al. Predicting residential radon concentrations in Finland: Model development, validation, and application to childhood leukemia. Scandinavian Journal of Work, Environment & Health. 2020;46(3):278‑92.

12. Chen J, Xie L. DOMESTIC RADON EXPOSURE AND CHILDHOOD LEUKAEMIA AND LYMPHOMA: A POPULATION-BASED STUDY IN CANADA. Radiation Protection Dosimetry. 1 oct 2019;184(3‑4):486‑92.

13. Del Risco Kollerud R, Blaasaas KG, Claussen B. Risk of leukaemia or cancer in the central nervous system among children living in an area with high indoor radon concentrations: results from a cohort study in Norway. Br J Cancer. 23 sept 2014;111(7):1413‑20.

14. Yoshinaga S, Tokonami S, Akiba S, Nitta H, Kabuto M. Case-control study of residential radon and childhood leukemia in Japan: results from preliminary analyses. International Congress Series. 1 févr 2005;1276:233‑5.

15. Berlivet J, Hémon D, Cléro É, Ielsch G, Laurier D, Faure L, et al. Residential exposure to natural background radiation at birth and risk of childhood acute leukemia in France, 1990–2009. Journal of Environmental Radioactivity. 1 juill 2021;233:106613.

16. Demoury C, Marquant F, Ielsch G, Goujon S, Debayle C, Faure L, et al. Residential Exposure to Natural Background Radiation and Risk of Childhood Acute Leukemia in France, 1990–2009. Environmental Health Perspectives. avr 2017;125(4):714‑20.

17. Berlivet J, Hémon D, Cléro É, Ielsch G, Laurier D, Guissou S, et al. Ecological association between residential natural background radiation exposure and the incidence rate of childhood central nervous system tumors in France, 2000-2012. J Environ Radioact. janv 2020;211:106071.

18. Zlobina A, Farkhutdinov I, Carvalho FP, Wang N, Korotchenko T, Baranovskaya N, et al. Impact of Environmental Radiation on the Incidence of Cancer and Birth Defects in Regions with High Natural Radioactivity. International Journal of Environmental Research and Public Health. janv 2022;19(14):8643.

19. Raaschou-Nielsen O, Andersen CE, Andersen HP, Gravesen P, Lind M, Schüz J, et al. Domestic radon and childhood cancer in Denmark. Epidemiology. juill 2008;19(4):536‑43.

20. The United Kingdom Childhood Cancer Study of exposure to domestic sources of ionising radiation: 1: radon gas. Br J Cancer. 5 juin 2002;86(11):1721‑6.

21. McLaughlin JR, King WD, Anderson TW, Clarke EA, Ashmore JP. Paternal radiation exposure and leukaemia in offspring: the Ontario case-control study. BMJ. 16 oct 1993;307(6910):959‑66.

22. Lucie NP. Radon and Acute Lymphoblastic Leukaemia. Leukemia & Lymphoma. 1 janv 1990;3(3):213‑6.

23. Henshaw DL, Eatough JP, Richardson RB. Radon as a causative factor in induction of myeloid leukaemia and other cancers. The Lancet. 28 avr 1990;335(8696):1008‑12.

24. Muirhead CR, Butland BK, Green BMR, Draper GJ. Childhood leukaemia and natural radiation. The Lancet. 23 févr 1991;337(8739):503‑4.

25. Boice JD, Mumma MT, Blot WJ. Cancer and Noncancer Mortality in Populations Living Near Uranium and Vanadium Mining and Milling Operations in Montrose County, Colorado, 1950–2000. rare. juin 2007;167(6):711‑26.

26. Boice JD, Mumma MT, Blot WJ. Cancer Incidence and Mortality in Populations Living Near Uranium Milling and Mining Operations in Grants, New Mexico, 1950–2004. rare. sept 2010;174(5):624‑36.

27. Ha M, Hwang S sik, Kang S, Park NW, Chang BU, Kim Y. Geographical Correlations between Indoor Radon Concentration and Risks of Lung Cancer, Non-Hodgkin’s Lymphoma, and Leukemia during 1999–2008 in Korea. Int J Environ Res Public Health. avr 2017;14(4):344.

28. Boz S, Berlin C, Kwiatkowski M, Bochud M, Bulliard JL, Zwahlen M, et al. A prospective cohort analysis of residential radon and UV exposures and malignant melanoma mortality in the Swiss population. Environ Int. nov 2022;169:107437.

29. Teras LR, Diver WR, Turner MC, Krewski D, Sahar L, Ward E, et al. Residential radon exposure and risk of incident hematologic malignancies in the Cancer Prevention Study-II Nutrition Cohort. Environ Res. juill 2016;148:46‑54.

30. Ruano-Ravina A, Aragonés N, Kelsey KT, Pérez-Ríos M, Piñeiro-Lamas M, López-Abente G, et al. Residential radon exposure and brain cancer: an ecological study in a radon prone area (Galicia, Spain). Sci Rep. 15 juin 2017;7(1):3595.

31. López-Abente G, Núñez O, Fernández-Navarro P, Barros-Dios JM, Martín-Méndez I, Bel-Lan A, et al. Residential radon and cancer mortality in Galicia, Spain. Science of The Total Environment. 1 janv 2018;610‑611:1125‑32.

32. Ruano-Ravina A, Aragonés N, Pérez-Ríos M, López-Abente G, Barros-Dios JM. Residential radon exposure and esophageal cancer. An ecological study from an area with high indoor radon concentration (Galicia, Spain). Int J Radiat Biol. avr 2014;90(4):299‑305.

33. Nilles JD, Lim D, Boyer MP, Wilson BD, Betar RA, Showalter HA, et al. The occurrence of bone and joint cancers and their association with rural living and radon exposure in Iowa. Environ Geochem Health. 5 avr 2022;

34. Messier KP, Serre ML. Lung and stomach cancer associations with groundwater radon in North Carolina, USA. Int J Epidemiol. 1 avr 2017;46(2):676‑85.

35. Groves-Kirkby CJ, Denman AR, Campbell J, Crockett RGM, Phillips PS, Rogers S. Is environmental radon gas associated with the incidence of neurodegenerative conditions? A retrospective study of multiple sclerosis in radon affected areas in England and Wales. J Environ Radioact. avr 2016;154:1‑14.

36. Wheeler BW, Kothencz G, Pollard AS. Geography of non-melanoma skin cancer and ecological associations with environmental risk factors in England. Br J Cancer. juill 2013;109(1):235‑41.

37. Auvinen A, Kurttio P, Pekkanen J, Pukkala E, Ilus T, Salonen L. Uranium and other natural radionuclides in drinking water and risk of leukemia: a case–cohort study in Finland. Cancer Causes Control. 1 nov 2002;13(9):825‑9.

38. Auvinen A, Salonen L, Pekkanen J, Pukkala E, Ilus T, Kurttio P. Radon and other natural radionuclides in drinking water and risk of stomach cancer: A case-cohort study in Finland. International Journal of Cancer. 2005;114(1):109‑13.

39. Kurttio P, Salonen L, Ilus T, Pekkanen J, Pukkala E, Auvinen A. Well water radioactivity and risk of cancers of the urinary organs. Environmental Research. 1 nov 2006;102(3):333‑8.

40. Forastiere F, Quiercia A, Cavariani F, Miceli M, Perucci CA, Axelson O. Cancer risk and radon exposure. Lancet. 2 mai 1992;339(8801):1115.

41. Forastiere F, Sperati A, Cherubini G, Miceli M, Biggeri A, Axelson O. Adult myeloid leukaemia, geology, and domestic exposure to radon and gamma radiation: a case control study in central Italy. Occup Environ Med. févr 1998;55(2):106‑10.

42. Puskin JS. SMOKING AS A CONFOUNDER IN ECOLOGIC CORRELATIONS OF CANCER MORTALITY RATES WITH AVERAGE COUNTY RADON LEVELS. Health Physics. avr 2003;84(4):526.

43. Boice JD, Cohen SS, Mumma MT, Chadda B, Blot WJ. Mortality among residents of Uravan, Colorado who lived near a uranium mill, 1936-84. J Radiol Prot. sept 2007;27(3):299‑319.

44. Jr JDB, Mumma M, Schweitzer S, Blot WJ. Cancer mortality in a Texas county with prior uranium mining and milling activities, 1950–2001. J Radiol Prot. sept 2003;23(3):247.

45. Cohen BL. Relationship Between Exposure to Radon and Various Types of Cancer. Health Physics. nov 1993;65(5):529.

46. Miller AS, Harwick RD, Alfaro-Miranda M, Sundararajan M. Search for correlation of radon levels and incidence of salivary gland tumors. Oral Surgery, Oral Medicine, Oral Pathology. 1 janv 1993;75(1):58‑63.

47. Miller D, Morrison H, Semenciw R, Mao Y. Leukemia and residential exposure to radon. Can J Public Health. 1993;84(3):205‑6.

48. Neuberger JS, Field RW. Radon and Breast Cancer. Risk Analysis. 1996;16(6):729‑30.

49. Eatough JP, Henshaw DL. Radon and prostate cancer. Lancet. 26 mai 1990;335(8700):1292.

50. Schwartz GG, Klug MG. Thyroid Cancer Incidence Rates in North Dakota are Associated with Land and Water Use. International Journal of Environmental Research and Public Health. janv 2019;16(20):3805.

51. Law GR, Kane EV, Roman E, Smith A, Cartwright R. Residential radon exposure and adult acute leukaemia. Lancet. 27 mai 2000;355(9218):1888.

52. Kjellberg S, Wiseman JS. The relationship of radon to gastrointestinal malignancies. Am Surg. sept 1995;61(9):822‑5.

53. Schubauer-Berigan MK, Daniels RD, Pinkerton LE. Radon exposure and mortality among white and American Indian uranium miners: an update of the Colorado Plateau cohort. Am J Epidemiol. 15 mars 2009;169(6):718‑30.

54. Roscoe RJ. An update of mortality from all causes among white uranium miners from the Colorado plateau study group. American Journal of Industrial Medicine. 1997;31(2):211‑22.

55. Ře řicha V, Kulich M, Ře řicha R, Shore DL, Sandler DP. Incidence of Leukemia, Lymphoma, and Multiple Myeloma in Czech Uranium Miners: A Case–Cohort Study. Environmental Health Perspectives. juin 2006;114(6):818‑22.

56. Kelly-Reif K, Sandler DP, Shore D, Schubauer-Berigan M, Troester M, Nylander-French L, et al. Lung and extrathoracic cancer incidence among underground uranium miners exposed to radon progeny in the Příbram region of the Czech Republic: a case-cohort study. Occup Environ Med. févr 2022;79(2):102‑8.

57. Kulich M, Reřicha V, Reřicha R, Shore DL, Sandler DP. Incidence of non-lung solid cancers in Czech uranium miners: a case-cohort study. Environ Res. avr 2011;111(3):400‑5.

58. Kelly-Reif K, Sandler DP, Shore D, Schubauer-Berigan M, Troester MA, Nylander-French L, et al. Mortality and cancer incidence among underground uranium miners in the Czech Republic 1977-1992. Occup Environ Med. août 2019;76(8):511‑8.

59. Kelly-Reif K, Sandler DP, Shore D, Schubauer-Berigan MK, Troester MA, Nylander-French L, et al. Radon and cancer mortality among underground uranium miners in the Příbram region of the Czech Republic. Am J Ind Med. oct 2020;63(10):859‑67.

60. Tomásek L, Darby SC, Swerdlow AJ, Placek V, Kunz E. Radon exposure and cancers other than lung cancer among uranium miners in West Bohemia. Lancet. 10 avr 1993;341(8850):919‑23.

61. Navaranjan G, Berriault C, Do M, Villeneuve PJ, Demers PA. Cancer incidence and mortality from exposure to radon progeny among Ontario uranium miners. Occup Environ Med. déc 2016;73(12):838‑45.

62. Zablotska LB, Lane RSD, Frost SE, Thompson PA. Leukemia, lymphoma and multiple myeloma mortality (1950-1999) and incidence (1969-1999) in the Eldorado uranium workers cohort. Environ Res. avr 2014;130:43‑50.

63. Lane RSD, Frost SE, Howe GR, Zablotska LB. Mortality (1950–1999) and Cancer Incidence (1969–1999) in the Cohort of Eldorado Uranium Workers. rare. oct 2010;174(6a):773‑85.

64. Zablotska LB, Lane RSD, Frost SE. Mortality (1950–1999) and cancer incidence (1969–1999) of workers in the Port Hope cohort study exposed to a unique combination of radium, uranium and γ-ray doses. BMJ Open. 1 janv 2013;3(2):e002159.

65. Kreuzer M, Dufey F, Marsh JW, Nowak D, Schnelzer M, Walsh L. Mortality from cancers of the extra-thoracic airways in relation to radon progeny in the Wismut cohort, 1946-2008. Int J Radiat Biol. nov 2014;90(11):1030‑5.

66. Walsh L, Dufey F, Tschense A, Schnelzer M, Grosche B, Kreuzer M. RADON AND THE RISK OF CANCER MORTALITY—INTERNAL POISSON MODELS FOR THE GERMAN URANIUM MINERS COHORT. Health Physics. sept 2010;99(3):292.

67. Kreuzer M, Dufey F, Laurier D, Nowak D, Marsh JW, Schnelzer M, et al. Mortality from internal and external radiation exposure in a cohort of male German uranium millers, 1946–2008. International Archives of Occupational and Environmental Health. mai 2015;88(4):431‑41.

68. Kreuzer M, Deffner V, Schnelzer M, Fenske N. Mortality in Underground Miners in a Former Uranium Ore Mine–Results of a Cohort Study Among Former Employees of Wismut AG in Saxony and Thuringia. Dtsch Arztebl Int. 29 janv 2021;118(4):41‑8.

69. Möhner M, Lindtner M, Otten H, Gille HG. Leukemia and exposure to ionizing radiation among German uranium miners. American Journal of Industrial Medicine. 2006;49(4):238‑48.

70. Möhner M, Lindtner M, Otten H. IONIZING RADIATION AND RISK OF LARYNGEAL CANCER AMONG GERMAN URANIUM MINERS. Health Physics. déc 2008;95(6):725.

71. Zablotska LB, Fenske N, Schnelzer M, Zhivin S, Laurier D, Kreuzer M. Analysis of mortality in a pooled cohort of Canadian and German uranium processing workers with no mining experience. Int Arch Occup Environ Health. 1 janv 2018;91(1):91‑103.

72. Rage E, Caër-Lorho S, Laurier D. Low radon exposure and mortality among Jouac uranium miners An update of the French cohort (1946-2007). Journal of Radiological Protection. mars 2018;38(1):92‑108.

73. Drubay D, Caër-Lorho S, Laroche P, Laurier D, Rage E. Mortality from Circulatory System Diseases among French Uranium Miners A Nested Case-Control Study. Radiation Research. mai 2015;183(5):550‑62.

74. Richardson DB, Rage E, Demers PA, Do MT, DeBono N, Fenske N, et al. Mortality among uranium miners in North America and Europe: the Pooled Uranium Miners Analysis (PUMA). International Journal of Epidemiology. 1 avr 2021;50(2):633‑43.

75. Silver SR, Bertke SJ, Hein MJ, Daniels RD, Fleming DA, Anderson JL, et al. Mortality and ionising radiation exposures among workers employed at the Fernald Feed Materials Production Center (1951–1985). Occup Environ Med. 1 juill 2013;70(7):453‑63.

76. Jr JDB, Cohen SS, Mumma MT, Chadda B, Blot WJ. A cohort study of uranium millers and miners of Grants, New Mexico, 1979–2005. J Radiol Prot. août 2008;28(3):303.

77. Veiga LHS, Amaral ECS, Colin D, Koifman S. A retrospective mortality study of workers exposed to radon in a Brazilian underground coal mine. Radiat Environ Biophys. 1 juill 2006;45(2):125‑34.

78. Darby SC, Radford EP, Whitley E. Radon exposure and cancers other than lung cancer in Swedish iron miners. Environ Health Perspect. mars 1995;103 Suppl 2:45‑7.

79. Darby SC, Whitley E, Howe GR, Hutchings SJ, Kusiak RA, Lubin JH, et al. Radon and cancers other than lung cancer in underground miners: a collaborative analysis of 11 studies. J Natl Cancer Inst. 1 mars 1995;87(5):378‑84.

80. Cocco PL, Carta P, Belli S, Picchiri GF, Flore MV. Mortality of Sardinian lead and zinc miners: 1960-88. Occupational and Environmental Medicine. 1 oct 1994;51(10):674‑82.

81. Chen SY, Hayes RB, Liang SR, Li QG, Stewart PA, Blair A. Mortality experience of haematite mine workers in China. Br J Ind Med. mars 1990;47(3):175‑81.

82. Golden AP, Ellis ED, Cohen SS, Mumma MT, Leggett RW, Wallace PW, et al. Updated mortality analysis of the Mallinckrodt uranium processing workers, 1942–2012. International Journal of Radiation Biology. 3 avr 2022;98(4):701‑21.

83. Tomásek L, Swerdlow AJ, Darby SC, Placek V, Kunz E. Mortality in uranium miners in west Bohemia: a long-term cohort study. Occup Environ Med. mai 1994;51(5):308‑15.

84. Hodgson JT, Jones RD. Mortality of a cohort of tin miners 1941-86. Occupational and Environmental Medicine. 1 oct 1990;47(10):665‑76.

85. Xiang-Zhen X, Lubin JH, Jun-Yao L, Li-Fen Y, Sheng LQ, Lan Y, et al. A Cohort Study in Southern China of Tin Miners Exposed to Radon and Radon Decay Products. Health Physics. févr 1993;64(2):120.
