# Supplementary material for: Radon exposure and potential health effects other than lung cancer: a systematic review and meta-analysis
Source: Front Public Health. 2024 Sep 25;12:1439355. doi: 10.3389/fpubh.2024.1439355 (PMC11461271; doi:10.3389/fpubh.2024.1439355)
Supplement: Supplementary file 7 [file Table_4.DOCX]

Supplementary Material

Table 3. Summary of studies in children, adults in the general population and mine workers on radon exposure and non-malignant health outcomes’ rates and risks included in the systematic revue and meta-analysis

| **Reference Region Study period (mean duration in year)** | **Study design** | **Study population Sample size** | **Radon Exposure assessment** | **Adjustment** | **Health outcome** | **Cases /Control*** | **Main results** | **NOS** | **UNSCEAR** |
| --- | --- | --- | --- | --- | --- | --- | --- | --- | --- |
| Langlois et al. 2016 (1) USA 1999 - 2009 | Cross-sectional study | Children and pregnancies n = 4,380,695 | Arithmetic mean geological region-level average ranged from 0.25 to 3.30 pCi/l | Race, age, education | Prenatal and within 1 year after delivery birth defects | 172,797 | Prevalence ratio of overall birth defects per 1 pCi/l increase in region-level average radon exposure (95%CI): 1.01(0.90 - 1.14). Results indicate increased risk for cystic hygroma, lymphangioma any site, cleft lip with or without cleft palate, and a decreased risk for reduction defects of the lower limb, Associations were suggested but not quite as consistent for Down syndrome. | 6 | Moderate |
| Zlobina et al. 2022 (2) Russia, China, France 2007 - 2018 ; 2008 - 2018 ; 2011 - 2016 ; 2014 - 2017 | Pooled ecological study | Children Age < 15 years n = 4 cities | City-level outdoor radon measurement in soil samples (in Bq/m3) | _ | Congenital malformations of the fetus | NA | Correlation between city-level incidence rates and city-level outdoor radon R-square = 0.35; p = 0.049 | _ | Moderate |
| Mukharesh et al. 2022 (3) USA 2008 - 2013 ; 2013 - 2014 ; 2015 - 2020 | Cross-sectional study | Children n = 3242 Age ≤ 15 years | Model-based ZIP code-level average radon exposure of the schools. Range of radon 5-month moving average in pCi/l : 1.02–1.77 | Age, race, cohort, and year | Asthma history | 810 | OR (95%CI) associated with 5-month moving average of radon exposure : 1.97 (1.25 - 3.11) Lower magnitude was found for other time windows of exposure (1, 7 , 12 and 24 months moving averages). | 6 | Moderate |
| Sheehan et al. 2020 (4) UK 2000 - 2011 | Ecological study | Children Age ≤ 9 years n = 354 local authority districts | Local authority district-level average radon potential class based on the UK radon atlas | Age, Sex, Nitrogen dioxide, lead in soil, race, overcrowding, Index of multiple deprivation living environment domain | Diabetes mellitus incidence | 13,948 | Relative risk, RR (95%CrI) between the diabetes mellitus and radon potential class : 1.044 (1.015 - 1.074) | _ | Moderate |
| Kim et al. 2020 (5) South Koree 2007 - 2015 | Cross-sectional study | Adults in the GP Mean age : 58.7 years n = 28, 557 | City/province-level average value of measured radon from 2012 to 2016. Mean radon (SD) : 103.1 (22.0) | Age, sex, hypertension, diabetes, dyslipidaemia, ischemic heart, BMI, household income, education status, occupation, smoking, alcohol consumption,  exercise, dietary intake | Stoke (prevalent cases) | 926 | OR (95%IC) per Bq/m3 increase : 1.004 (1.001–1.007) ; P=.01 High radon level (>100 Bq/m3) vs low level of radon (≤ 100 Bq/m3) : 1.242 (1.069–1.444) ; p-value = .005 | 6 | Moderate |
| Ruano-Ravina et al. 2020 (6) Spain | Hospital-based case-control study | Adults in the GP Median age (IQR) : in cases : 64 (60–68) in controls : 60 (51–67) n = 936 | At least 3-month direct radon measurements in participants home at the time of enrolment Median (IQR) in Bq/m3 : in cases : 157 (93–309) control : 165 (106–303) | Age, sex, education, tobacco consumption (never; light smokers: <33 pack-year; moderate smokers: 34–66 pack-years ; heavy smokers: >66 pack-years) | COPD (prevalent cases) | 189/747 | OR (95%IC) across radon exposure categories (in Bq/m3) ≤50 : 1.0 (reference) 51–147 : 1.26 (0.40–3.98) 148–199 : 2.06 (0.60–7.13) >199 : 1.58 (0.50–4.98) | 5 | Moderate |
| Pando-Sandoval et al. 2022 (7) Spain | Hospital-based case-control study | Adults in the GP Median age (IQR) : in cases : 64 (60–68) n = 936 | Direct individual radon measurements in home of cases involved in a previous case-controls studies on residential radon and lung cancer Median radon concentration : 68.0 Bq/m3 median residential radon concentration in Bq/m3 (25th–75th percentile) : 157 (93–309) | _ | COPD primary diagnosis, and hospital admission | 189/747 | Association between FEV1% categories and radon exposure categories : p-value of chi2 test = 0.460 Association between number of hospital admission categories due to COPD exacerbation in preceding 2 years and radon exposure categories : p-value of chi2 test = 0.465 | 5 | Moderate |
| Barbosa-Lorenzo et al. 2017 (8) Spain 2008 - 2013 | Ecological study | Adults in the GP Age ≥45 years n = 237 municipalities (1,292,628 persons) | Average municipality-level radon concentration was based on radon measurements obtained from the Galician Radon Map and from controls belonging to two previous case-controls studies on residential radon and lung cancer. Median radon (min - max) in Bq/m3 : 107.0 (13.0 - 569.5) | Percentage of illiterate, percentage of social security affiliations by gender, median income for retired persons, municipality gross product, average of primary care physicians by inhabitant, percentage of workers and rurality index, bladder cancer standardized mortality rate and minimum distance to hospital | COPD (prevalent) | 49,393 | Relative risk, RR (95%CI) per 100 Bq/m3 increased in radon associated to: - COPD diagnosed in primary care : 0.95 (0.93–0.97) - COPD hospital admission : 1.04 (1.00–1.08) RRs (95%CI) for radon exposure ≥ 50 compare to < 50 Bq/m3 indicate no statistically significant association for COPD hospital admission, but a marginally increased risk of COPD diagnosed in primary care | _ | Moderate |
| Mauriz-Barreiro et al. 2022 (9) Spain January - December 2017 | Ecological study | Adults in the GP n = 46 municipalities (442,950 inhabitants) Median age (IQR) : 41 (33 - 56 ) | Municipality-level average geometric mean radon concentration in Bq/m3 based on radon measurements obtained from the Galician Radon Map. Range : 65.0 - 257.6 | Age, sex | Inflammatory Bowel Disease incidence | 96 | Correlation between cumulative incidence and average radon concentration at municipality level r = 0.13; p-value = 0.5 In females : r = -0.13 ; p = 0.5 In males : r = 0.06 ; p = 0.7 | _ | Moderate |
| Fathabadi et al. 2020 (10) Iran 2017 - 2018 | Case-control study | Adults in the GP n = 145 Mean age (SD) In cases : 34.51 (8.53) In controls : 40.14 (12.45) | Direct long-term radon measurement in participants' home | _ | Multiple sclerosis (prevalent cases) | 45/100 | OR (95%CI) : 0.167 (0.01 - 4.86) ; p = 0.983 | 5 | Moderate |
| Schwartz et al. 2016 (11) USA 2001 - 2010 | Ecological study | Adults in the GP n = 47 states | State-level radon score proportional to the weighted average of radon measured at home in all the counties of that state | Race (percentage of white) | Motor neuron disease mortality | _ | No statistically significant relationship between radon and motor neuron disease (p for covariance = 0.972) | _ | Moderate |
| Groves-Kirkby et al. 2016 (12) UK 2005 - 2012 | Cohort study | Adults in the GP | Radon bands defined based on the percentage of homes within a postcode sector where radon levels above 200 Bq/m3 | Race (percentage of white) | Multiple sclerosis incidence | 115 | ERR per 100 Bq/m3 increase in mean radon : 0.22 ; p = 0.0961 | 5 | Moderate |
| Boice et al. 2007 (13) USA 1979 - 2004 | Cohort study | Adults in the GP n = 1905 | Exposure period : 1936 - 2004 | Age, sex, race, calendar-year | Diabetes mellitus mortality | 17 | SMR : 0.98 (0.57 - 1.57) | 8 | Moderate |
|  |  |  |  |  | Mental and behavioural disorders mortality | 5 | SMR (95%CI) : 0.50 (0.16 - 1.18) |  |  |
|  |  |  |  |  | Nervous system disease mortality | 20 | SMR (95%CI) : 1.10 (0.67 - 1.69) |  |  |
|  |  |  |  |  | Cerebrovescular disease mortality | 32 | SMR (95%CI) : 0.65 (0.44 - 0.91) |  |  |
|  |  |  |  |  | All heart disease mortality | 204 | SMR (95%CI) : 0.76 (0.66 - 0.87) |  |  |
|  |  |  |  |  | Bronchitis, emphysema, asthma mortality | 21 | SMR (95%CI) : 1.03 (0.64 - 1.57) |  |  |
|  |  |  |  |  | Cirrhosis of liver mortality | 2 | SMR (95%CI) : 0.20 (0.02 - 0.72) |  |  |
|  |  |  |  |  | Nephritis and nephrosis mortality | 6 | SMR (95%CI) : 0.69 (0.25 - 1.50) |  |  |
| Boice et al. 2007 (14) USA 1960 - 1999 | Ecological study | Adults in the GP n = 1 study county and 5 control counties in Colorado | _ | Age, sex, race, calendar-year | Diabetes mellitus mortality | 152 | RR, relative risk (95%CI) which represents the ratio of SMR for the study population (Montrose county) and that for the 5 comparison counties. The reference population was the Colorado general population. RR (95%CI) : 1.02 (0.86–1.21) | _ | Moderate |
|  |  |  |  |  | Mental and behavioural disorders mortality | 5 | RR (95%CI) : 0.50 (0.16 - 1.18) |  |  |
|  |  |  |  |  | Nervous system disease mortality | 20 | RR (95%CI) : 1.10 (0.67 - 1.69) |  |  |
|  |  |  |  |  | Cerebrovascular mortality | 720 | RR (95%CI) : 1.07 (0.99–1.16) |  |  |
|  |  |  |  |  | All heart disease mortality | 2638 | RR (95%CI) : 0.98 (0.94–1.02) |  |  |
|  |  |  |  |  | Hypertension with heart disease mortality | 58 | RR (95%CI) : 0.72 (0.55–0.94) |  |  |
|  |  |  |  |  | Hypertension without heart disease mortality | 23 | RR (95%CI) : 0.65 (0.42–0.99) |  |  |
|  |  |  |  |  | Bronchitis, emphysema, asthma mortality | 188 | RR (95%CI) : 1.07 (0.92–1.25) |  |  |
|  |  |  |  |  | Bronchitis mortality | 37 | RR (95%CI) : 0.89 (0.63–1.26) |  |  |
|  |  |  |  |  | Emphysema mortality | 126 | RR (95%CI) : 1.10 (0.91–1.33) |  |  |
|  |  |  |  |  | Asthma mortality | 25 | RR (95%CI) : 1.30 (0.84–1.99) |  |  |
|  |  |  |  |  | Ulcer of stomach and duodenum mortality | 44 | RR (95%CI) : : 1.22 (0.88–1.68) |  |  |
|  |  |  |  |  | Cirrhosis of liver mortality | 97 | RR (95%CI) : 1.11 (0.90–1.38) |  |  |
|  |  |  |  |  | Nephritis and nephrosis mortality | 68 | RR (95%CI) : 1.09 (0.84–1.41) |  |  |
| Suzuki et al. 1994 (15) Japan 1989- 1991 | Ecological study | Adults in the GP n = 1 studied city in low radon spa area and 5 control cities Age ≥ 40 | Radon measurement in Spa area | Age, sex | All circulatory system disease mortality | _ | RR (95%CI): 1.27 (1.01 - 1.61) | _ | Moderate |
|  |  |  |  |  | Cerebrovascular mortality | _ | RR (95%CI) : 1.00 (0.75 - 1.32) |  |  |
| Lehrer et al. 2017 (16) USA | Ecological study | Adults in the GP n = 50 states and district in columbia | State-level average radon exposure | Age, hypertension death rate, diabetes deaths rate | Alzheimer disease mortality | 84,767 | Correlation between radon and alzheimer disease deaths rates : 0.169 ; p <0.001 | _ | Moderate |
| Neilson et al. 1996 (17) UK 1981 - 1989 | Ecological study | Adults in the GP n = 55 counties | County-level average radon exposure in Bq/m3 | Age, sex, life expectancy at age 15 years | Motor neuron disease mortality | 9220 | Partial linear model coef in the whole study population : 0.3675 ; p = 0.0018) in men : 0.4285 ; p = 0.0002 In Women : 0.1279 ; p > 0.05 | _ | Moderate |
| Bolviken et al. 2003 (18) Norway 1951 - 1978 | Pooled ecological study | Adults in the GP n = 73 rural municipalities | Rural/municipality-level average radon exposure | Age, sex | Multiple sclerosis incidence | _ | Correlation coefficients are significant at p <0.01 for about 70% of the radon exposure area windows positions | _ | Moderate |
| Kelly-Reif et al. 2022 (19) USA 1960 - 2016 | Cohort study | Colorado plateau uranium miners n=4021 miners (male) Median age at hire : 29 years | Radon exposure assessment is based the mine location and year, combined with available radon progeny measurement data. Exposure period : 1950 - 1960 Median cumulative radon exposure : 431 WLM | For SMRs calculation : age, calendar period, racialization. For ERR : attained age (time scale), birth cohort, duration of employment (in decades). | Asthma mortality | 11 | SMR (95%CI) : 1.97 (0.98 - 3.53) | 8 | Moderate |
|  |  |  |  |  | COPD mortality | 254 | ERR (95%CI) per 100 WLM increase in cumulative radon exposure : 0.00 (0.00 - 0.02) |  |  |
| Schubauer- Berigan et al. 2009 (20) USA 1977- 2004 | Cohort study | Colorado plateau uranium miners n = 4022 (3255 (White Americanss, 767 American Indians) | Radon exposure assessment is based on the mine location and year, combined with available radon progeny measurement data. Exposure period : 1950 - 1960 Mean cumulative in WLM inn White Americanss : 806 in american indians : 742 | Age, calendar year, race | Systemic end-stage renal disease incidence | 25 | SIRs (95%CI) in White Americans : 0.54 (0.25 - 1.0) in American Indians: 1.1 (0.64 - 1.8) | 7 | Moderate |
|  |  |  |  |  | Diabetes mellitus incidence | 20 | SIRs (95%CI) in White Americans : 0.65 (0.21 - 1.5)  in American Indians: 2.2 (1.3 - 3.7) |  |  |
|  |  |  |  |  | Hypertensive disease incidence | 4 | SIRs (95%CI) in White Americans : 0.60 (0.16 - 1.5)  in American Indians: 0.00 (NE - NE) |  |  |
|  |  |  |  |  | Non-systemic end-stage renal disease incidence | 6 | SIRs (95%CI) in White Americans : 1.4 (0.37 - 3.5)  in American Indians: 1.4 (0.17 - 5.2) |  |  |
| Schubauer- Berigan et al. 2009 (20) USA 1960- 2005 | Cohort study | Colorado plateau uranium miners n = 4022 (3255 (White Americanss, 767 American Indians) | Radon exposure assessment is based on the mine location and year, combined with available radon progeny measurement data. Exposure period : 1950 - 1960 Mean cumulative in WLM In White Americans : 806 In American Indians: 742 | Age, calendar year, race | All causes except lung cancer, COPD, and pneumoconioses mortality | 2076 | SMRs (95%CI) in White Americans : 1.22 (1.16 - 1.28)  in American Indians: 0.93 (0.85 - 1.02) | 7 | Moderate |
| Schubauer- Berigan et al. 2009 (20) USA 1960- 2005 | Cohort study | Colorado plateau uranium miners White Americanss : 3255 American Indians : 767 | Radon exposure assessment is based on the mine location and year, combined with available radon progeny measurement data. Exposure period : 1950 - 1960 Mean cumulative in WLM In White Americanss : 806 In American indians : 742 | Age, calendar year, race | Blood and blood-forming organs disease mortality | 12 | SMRs (95%CI) in White Americans : 2.07 (1.07 - 3.61)  in American Indians: 0 (0 - 2.19) | 7 | Moderate |
|  |  |  |  |  | Diabetes mellitus mortality | 56 | SMRs (95%CI) in White Americans : 1.10 (0.75 - 1.55)  in American Indians : 1.16 (0.74 - 1.73) |  |  |
|  |  |  |  |  | Mental disorders excluding alcoholism mortality | 17 | SMRs (95%CI) in White Americans : 1.54 (0.90 - 2.47)  in American Indians: 0 (0 - 0.94) |  |  |
|  |  |  |  |  | Nervous system disorders mortality | 42 | SMRs (95%CI) in White Americans : 1.02 (0.70 - 1.44)  in American Indians: 1.32 (0.63 - 2.43) |  |  |
|  |  |  |  |  | Rheumatic heart disease mortality | 15 | SMRs (95%CI) in White Americans : 1.49 (0.83 - 2.46)  in American Indians0 (0 - 1.47) |  |  |
|  |  |  |  |  | Chronic disease of endocardium mortality | 5 | SMRs (95%CI) in White Americans : 0.74 (0.24 - 1.73)  in American Indians: 0 (0 - 2.91) |  |  |
|  |  |  |  |  | Cardiomyopathy mortality | 13 | SMRs (95%CI) in White Americans : 0.93 (0.55 - 1.71)  in American Indians: 0.71 (0.15 - 2.07) |  |  |
|  |  |  |  |  | Conductive disorder mortality | 49 | SMRs (95%CI) in White Americans : 1.78 (1.21 - 2.52)  in American Indians: 1.85 (1.09 - 2.92) |  |  |
|  |  |  |  |  | Hypertension (with and without mention of heart disease) mortality | 10 | SMRs (95%CI) in White Americans : 0.48 (0.19 - 1.02)  in American Indians: 0.37 (0.08 - 1.07) |  |  |
|  |  |  |  |  | Other heart diseases mortality | 58 | SMRs (95%CI) in White Americans : 1.62 (1.17 - 2.19)  in American Indians: 1.62 (0.93 - 2.63) |  |  |
|  |  |  |  |  | Cerebrovascular disease mortality | 92 | SMRs (95%CI) in White Americans : 0.98 (0.78 - 1.23)  in American Indians: 0.53 (0.29 - 0.90) |  |  |
|  |  |  |  |  | Diseases of arteries, veins, lymphatic system mortality | 50 | SMRs (95%CI) in White Americans : 1.03 (0.76, 1.37)  in American Indians: 0.33 (0.07 - 0.98) |  |  |
|  |  |  |  |  | Acute glomerulonephritis and acute renal failure mortality | 6 | SMRs (95%CI) in White Americans : 3.17 (1.16 - 6.89)  in American Indians: 0.00 (NA - NA) |  |  |
|  |  |  |  |  | Chronic and unspecified nephritis and renal failure mortality | 18 | SMRs (95%CI) in White Americans : 1.20 (0.65 - 2.01)  in American Indians: 0.56 (0.15 - 1.44) |  |  |
|  |  |  |  |  | Digestive system disease excluding cirrhosis mortality | 52 | SMRs (95%CI) in White Americans : 1.16 (0.85 - 1.56)  in American Indians:0.43 (0.17 - 0.93) |  |  |
|  |  |  |  |  | Skin and subcutaneous tissues diseases mortality | 3 | SMRs (95%CI) in White Americans : 2.18 (0.45 - 6.38)  in American Indians: 0 (0 - 3.50) |  |  |
|  |  |  |  |  | Musculoskeletal and connective tissue diseases mortality | 10 | SMRs (95%CI) in White Americans : 1.49 (0.60 - 3.06)  in American Indians: 1.98 (0.41 - 5.78) |  |  |
|  |  |  |  |  | Other genitourinary diseases mortality | 15 | SMRs (95%CI) in White Americans : 1.24 (0.62 - 2.26)  in American Indians: 0.74 (0.20 - 1.90) |  |  |
| Roscoe et al. 1997 (21) USA 1960 - 1990 | Cohort study | Colorado plateau White Americans uranium miners n = 3238 Mean age at death : 62 | Radon exposure assessment is based on the mine location and year, combined with available radon progeny measurement data. Exposure period : 1950 - 1964 Mean cumulative radon exposure in WLM (SD) : 811 (1107.1) | Age, sex, calendar year, race | Blood and blood-forming organs disease mortality | 7 | Internally SRRs (95%CI) across radon categories in WLM <120 : 1.00 (reference) 120–<400 : 1.4 (0.1–15.1) 400–≤1000: 1.6 ( 0.1–17.6) >1000 : 1.6 ( 0.1–17.3)  SMRs across radon categories indicate no statistically significant association | 8 | Moderate |
|  |  |  |  |  | Mental disorders mortality | 14 | SMR (95%CI) : 1.4 (0.7–2.2) |  |  |
|  |  |  |  |  | Emphysema mortality | 56 | SMR (95%CI) : 2.5 (1.9–3.2) |  |  |
| Kelly-Reif et al. 2019 (22) Czech Republic 1977 - 1992 | Cohort study | Příbram region underground uranium miners n = 16,434 Mean age at hire : 28 | Exposure period : 1946 - 1976 Mean cumulative radon in WLM (range) 53.2 (1.2–1121.9) | Age, sex | Diabetes mellitus mortality | 34 | SMR (95%CI) : 0.75 (0.50- 1.00) | 7 | Moderate |
|  |  |  |  |  | Blood & Blood Forming disease organs mortality | 5 | SMR (95%CI) : 1.29 (0.15 - 2.42) |  |  |
|  |  |  |  |  | Mental disorders mortality | 20 | SMR (95%CI) : 1.88 (1.05 - 2.71) |  |  |
|  |  |  |  |  | Nervous System Disorders mortality | 19 | SMR (95%CI) : 0.72 (0.39 - 1.04) |  |  |
|  |  |  |  |  | Acute Myocardial Infarction mortality | 230 | SMR (95%CI) : 0.38 (0.33- 0.42) |  |  |
|  |  |  |  |  | Cerebrovascular disease mortality | 148 | SMR (95%CI) : 0.35 (0.29- 0.41) |  |  |
|  |  |  |  |  | Atherosclerosis mortality | 403 | SMR (95%CI) : 3.88 (3.50- 4.26) |  |  |
|  |  |  |  |  | Arteries, arterioles, capillaries, and allied conditions (Including atherosclerosis) mortality | 419 | SMR (95%CI) : 3.46 (3.12- 3.79) |  |  |
|  |  |  |  |  | COPD and Allied Conditions including asthma mortality | 96 | SMR (95%CI) : 0.84 (0.67- 1.01) |  |  |
|  |  |  |  |  | Stomach & Duodenum diseases mortality | 17 | SMR (95%CI) : 0.65 (0.34- 0.97) |  |  |
|  |  |  |  |  | Pancreas disease mortality | 20 | SMR (95%CI) : 1.35 (0.76- 1.94) |  |  |
|  |  |  |  |  | Genitourinary System diseases mortality | 55 | SMR (95%CI) : 0.73 (0.54- 0.92) |  |  |
|  |  |  |  |  | Nephritis, Nephrotic syndrome, and Nephrosis mortality | 26 | SMR (95%CI) : 0.94 (0.58- 1.30) |  |  |
|  |  |  |  |  | Urinary System and Male Genital Organs diseases mortality | 8 | SMR (95%CI) : 0.36 (0.15- 0.7) |  |  |
| Villeneuve et al. 2023 (23) Canada 1950 - 2016 | Cohort study | n = 2050 (1735 underground miners at some point, 315 exclusively surface workers) | Retrospective radon exposure reconstruction based on inspector report, mine samples measurement, and individual dosimeters measures. Exposure period : 1933 - 1978 Mean annual radon exposure among underground miners : 43.6 WLM | Attained age, calendar period, and average number of cigarettes smoked daily | Circulatory system disease mortality | 480 | ERR (95%CI) per 100 WLM increase in cumulative radon expsosure 0.002 (-0.020 - 0.023) SMR (95%CI) = 0.82 (0.74 - 0.91) Relative Risks RRs (95%CI) across cumulative radon exposure quintiles indicate no statistically significant association | 6 | Moderate |
|  |  |  |  |  | Ischemic heart disease mortality | 290 | ERR (95%CI) per 100 WLM increase in cumulative radon expsosure 0.005 (-0.031 - 0.022) RRs (95%CI) across cumulative radon exposure quintiles indicate no statistically significant association |  |  |
|  |  |  |  |  | Acute myocardial infarction mortality | 175 | ERR (95%CI) per 100 WLM increase in cumulative radon expsosure 0.026 (-0.018 - 0.070) RRs (95%CI) across cumulative radon exposure quintiles indicate no statistically significant association |  |  |
|  |  |  |  |  | Cerebrovascular mortality | 60 | ERR (95%CI) per 100 WLM increase in cumulative radon expsosure -0.35 (-0.76 - 0.00) RRs (95%CI) across cumulative radon exposure quintiles indicate no statistically significant association |  |  |
| Navaranjan et al. 2016 (24) Canada 1954 - 2007 | Cohort study | Ontario uranium miners n = 28,546 males and 413 females (were not included in the dose-response analysis given the small number) Mean age at entry in males : 28.8 in females : 27.9 | Mine-specific extrapolations and areas sampling of radon decay products were use to estimate individual exposure to radon. Mean radon in WLM (range) in males : 21.0 (0.0–875.1) in females : 0.2 (0.0–16.3 | Calendar period, attained age | Major cardiovascular disease mortality | 2681 | SMR (95%CI) : 0.85 (0.82 - 0.89) | 6 | Moderate |
|  |  |  |  |  | CeVD mortality | 315 | SMR (95%CI) : 0.76 (0.68 - 0.84) |  |  |
| Zeng et al. 2022 (25) Canada 1992 - 2018 | Cohort study | Ontario male uranium miners n = 34,536 | Radon exposure was estimated from a job-exposure matrice Exposure period : 1915–1988 Mean cumulative radon exposure in WLM (SD) : 7.5 (24.4) | Attained age, attained age square, birth year throughout the follow‐up period, exposure to self‐reported McIntyre Powder (respirable aluminum) | Alzheimer disease incidence | 789 | Incidence Rate Ratios, IRRs (95%CI) across cumulative radon exposure categories >0 - 1 : 1.00 (reference) >1 − 5 : 1.23 (1.05–1.45) >5 − 10 : 1.09 (0.83–1.45) >10 − 20 : 0.93 (0.67–1.28) >20 − 50 : 1.09 (0.79–1.51) >50 : 1.03 (0.73–1.44) | 6 | Moderate |
|  |  |  |  |  | Alzheimer with other dementias disease incicence | 4633 | IRRs (95%CI) >0 - 1 : 1.00 (reference) >1 − 5 : 0.95 (0.88–1.01) >5 − 10 : 1.03 (0.92–1.16) >10 − 20 : 1.01 (0.89–1.15) >20 − 50 : 0.96 (0.84–1.10) >50 : 0.93 (0.8–1.06) |  |  |
|  |  |  |  |  | Parkinson disease incidence | 595 | IRRs (95%CI) >0 - 1 : 1.00 (reference) >1 − 5 : 0.97 (0.83–1.13) >5 − 10 : 1.23 (0.97–1.55) >10 − 20 : 0.83 (0.62–1.11) >20 − 50 : 0.86 (0.63–1.17) >50 : 0.78 (0.56–1.07) |  |  |
|  |  |  |  |  | Parkinsonism incidence | 949 | IRRs (95%CI) >0 - 1 : 1.00 (reference) >1 − 5 : 0.97 (0.83–1.13) >5 − 10 : 1.23 (0.97–1.55) >10 − 20 : 0.83 (0.62–1.11) >20 − 50 : 0.86 (0.63–1.17) >50 : 0.78 (0.56–1.07) |  |  |
|  |  |  |  |  | Motor neuron disease incidence | 64 | IRR (95%CI) >0 - 1 : 1.00 (reference) >1 − 5 : 0.53 (0.31–0.90) |  |  |
| Lane et al. 2010 (26) Canada 1950 - 1999 | Cohort study | Eldorado uramium miners (Underground, surface) n = 17,660 | Exposure period : 1932 - 1980 Mean radon exposure weithed by person-year in WLM (SD) : In men : 100.2 (254.4) in female : 4.6 (10.1) | Age at risk, calendar year at risk and duration of employment | Diabetes mellitus mortality | 64 | ERR per 100 WLM : 0 ; p = 0.98 | 7 | Moderate |
|  |  |  |  |  | Endocrine cancer mortality | 61 | ERR per 100 WLM : -0.04 ; p = 0.29 |  |  |
|  |  |  |  |  | Ischemic heart disease mortality | 1235 | ERR per 100 WLM : -0.01 ; p = 0.18 |  |  |
|  |  |  |  |  | Stroke mortality | 244 | ERR (95%CI) per 100 WLM : -0.04 ; p = 0.012 |  |  |
|  |  |  |  |  | Other cardiovascular diseases mortality | 317 | ERR (95%CI) per 100 WLM : -0.02 ; p = 0.49 |  |  |
|  |  |  |  |  | Cirrhosis of liver mortality | 60 | ERR (95%CI) per 100 WLM : 0.03 ; p = 0.77 |  |  |
|  |  |  |  |  | Digestive diseases mortality | 179 | ERR (95%CI) per 100 WLM : -0.03 ; p = 0.33 |  |  |
| Zablostska et al. 2013 (27) Canada 1950 - 1999 | Cohort study | Port Hope uranium miners (Surface and underground uranium miners, millers) n = 2645 males | Exposure period : 1932 - 1980 Mean cumulative radon exposure in WLM (SD, range) in men : 13.3 (45.9, 0–627.6) | For SMRs : age at risk, calendar year at risk For ERRs : age at risk, calendar year at risk and duration of employment | Diabetes mellitus mortality | 14 | SMR (95%CI) : 0.72 (0.39 - 1.21) | 7 | Moderate |
|  |  |  |  |  | Nervous System Disorders mortality | 11 | SMR (95%CI) : 0.63 (0.31 - 1.12) |  |  |
|  |  |  |  |  | COPD and Allied Conditions including asthma mortality | 25 | SMR (95%CI) : 0.58 (0.37 - 0.85) |  |  |
|  |  |  |  |  | Circulatory system disease mortality | 514 | ERR (95%CI) per 100 WLM : 0.10 (-0.05 - 0.32) ; p = 0.22 |  |  |
|  |  |  |  |  | Hypertensive disease mortality | 13 | SMR (95%CI) : 2.67 (1.42 - 4.57) |  |  |
|  |  |  |  |  | Cerebrovascular mortality | 71 | SMR (95%CI) : 1.03 (0.81 - 1.30) |  |  |
|  |  |  |  |  | Other circulatory system disease mortality | 84 | SMR (95%CI) : 1.06 (0.85 - 1.32) |  |  |
|  |  |  |  |  | Other genitourinary diseases mortality | 11 | SMR (95%CI) : 0.86 (0.43 - 1.54) |  |  |
|  |  |  |  |  | Nephritis and nephrosis mortality | 7 | SMR (95%CI) : 1.10 (0.44 - 2.27) |  |  |
| Kreuzer et al. 2013 (28) Germany 1946 - 2008 | Cohort study | Wismut male uranium miners (underground, open pit, surface, milling) n = 58,690 | Cumulative radon exposure in Working Level Months (WLM) was determined from a comprehensive job-exposure matrix, based on ambient measurements and detailed expert rating Exposure period : 1946 - 1990) Mean cumulative radon in the exposed group : 280 | Age, calendar year, duration of employment | COPD mortality | 715 | ERR per 100 WLM increase in cumulative radon exposure : 0.007 ; p = 0.41 Relative Risks, RRs across cumulative radon exposure categories indicate no association | 6 | Moderate |
|  |  |  |  |  |  |  |  |  |  |
|  |  |  |  |  |  |  |  |  |  |
|  |  |  |  |  |  |  |  |  |  |
|  |  |  |  |  |  |  |  |  |  |
| Kreuzer et al. 2010 (29) Germany 1946 - 2003 (35) | Cohort study | Wismut male uranium miners (underground, open pit, surface, milling) n = 58,987 Mean age at first exposure (range) : 25 (14 - 67) | Cumulative radon exposure in Working Level Months (WLM) was determined from a comprehensive job-exposure matrix, based on ambient measurements and detailed expert rating Exposure period : 1946 - 1990 Mean cumulative exposure to radon among exposed miners in WLM (range) : 280 (> 0 , 3224) | Age, calendar year | All circulatory system disease mortality | 7395 | ERR and p-value per 100 WLM increase in cumulative radon exposure : 0.001; p > 0.5 | 6 | Moderate |
|  |  |  |  |  | Ischemic heart disease mortality | 5141 | ERR : 0.000 ; p > 0.5 |  |  |
|  |  |  |  |  | Acute myocardial infarction mortality | 2074 | ERR : 0.008 ; p = 0.114 |  |  |
|  |  |  |  |  | Cerebrovascular disease mortality | 1742 | ERR : 0.001; p > 0.5 |  |  |
| Kreuzer et al. 2006 (30) Germany 1946 -1998 (30.5) | Cohort study | Wismut male uranium miners (underground, open pit, surface, milling) n=59,001 | Cumulative radon exposure in Working Level Months (WLM) was determined from a comprehensive job-exposure matrix, based on ambient measurements and detailed expert rating Exposure period : 1946 - 1989 Mean cumulative exposure to radon : 241 WLM | Age, calendar year | All circulatory system disease mortality | 5417 | Relative Risk, RR (95%CI) across cumulative radon exposure categories 0 : 1.00 (reference)  >0 - 100 : 0.96 (0.89–1.03)  >100 - 400 : 0.93 (0.85–1.02) >400 - 800 : 0.98 (0.91–1.05) >800 - 1600 : 0.92 (0.85–0.98) >1600 : 1.11 (0.97–1.25) | 6 | Moderate |
|  |  |  |  |  | Ischemic heart disease mortality | 3719 | RRs (95%CI) 0 : 1.00 (reference)  >0 - 100 : 0.93 (0.85–1.00) >100 - 400 : 0.91 (0.80–1.01) >400 - 800 : 0.97 (0.88–1.05) >800 - 1600 : 0.89 (0.81–0.96) >1600 : 1.08 (0.91–1.24) |  |  |
|  |  |  |  |  | Cerebrovascular disease mortality | 1297 | RRs (95%CI) 0 : 1.00 (reference)  >0 - 100 : 1.02 (0.89–1.17) >100 - 400 : 1.01 (0.82–1.20) >400 - 800 : 0.99 (0.85–1.14) >800 - 1600 : 0.97 (0.82–1.11) >1600 : 1.14 (0.84–1.44) |  |  |
|  |  |  |  |  |  |  |  |  |  |
| Kreuzer et al. 2015 (31) Germany 1970 - 2008 | Cohort study | Wismut male uranium miners milling n = 4054 Mean age at first employment (range) : 25 (14–61) | Cumulative radon exposure in Working Level Months (WLM) was determined from a comprehensive job-exposure matrix, based on ambient measurements and detailed expert rating Exposure period : 1946 - 1990 Mean cumulative exposure to radon among exposed miners in WLM (range) : 280 (> 0 , 3224) | Age, sex, calendar year | Cerebrovascular mortality | 159 | SMR (95%CI) : 1.15 (0.97 - 1.32) | 6 | Moderate |
|  |  |  |  |  | Urogenital system diseases mortality | 12 | SMR (95%CI) : 1.65 (0.77 - 3.08) ERR : 0.88 (-6.28 - 8.04) |  |  |
| Kreuzer et al. 2021 (32) Germany 1960 - 2013 | Cohort study | Wismut male underground uranium miners n = 35,204 | Cumulative radon exposure in Working Level Months (WLM) was determined from a comprehensive job-exposure matrix, based on ambient measurements and detailed expert rating Exposure period : 1946 - 1989 Mean cumulative radon exposure : 364 WLM | Age, sex, calendar year | Metabolic diseases mortality | 363 | SMR (95%CI) : 1.00 (0.90 - 1.11) | 6 | Moderate |
|  |  |  |  |  | Mental disorders : 0.81 (0.70 - 0.94) | 191 | SMR (95%CI) : 1.05 (0.87 - 1.27) |  |  |
|  |  |  |  |  | Nervous system disease mortality | 163 | SMR (95%CI) : 0.73 (0.62 - 0.85) |  |  |
|  |  |  |  |  | Amyotrophic lateral sclerosis mortality | 20 | SMR (95%CI) : 1.10 (0.67 - 1.70) |  |  |
|  |  |  |  |  | Cerebrovascular disease mortality | 1335 | SMR (95%CI) : 1.33 (1.26 - 1.41) |  |  |
|  |  |  |  |  | Kidney diseases mortality | 105 | SMR (95%CI) : 0.62 (0.51 - 0.75) |  |  |
| Zablotska et al. 2018 (33) Canada & Germany 1946 - 2008 (in Wismut cohort) 1950 - 1999 (in Port Hope cohort) | Pooled cohort study | Uranium processing workers with no mining experience (Port hope and Wismut millers) n = 7431 (625 females, and 6806 males). Average age at start of employement (SD) : 29 (10) to 30 (11) | Exposure period : 1942 - 1996 across studies Mean cumulative RDP exposure among males : 16.6 WLM (SD = 49.8) Exposure period : 1932 - 1990 accros cohorts | For ERRs : Age at risk, cohort, duration of employment For RRs of CSD : calendar time, age at risk, cohort and duration of employment | All circulatory system disease mortality | 1358 | Relative Risks, RRs (95%CI) across cumulative radon exposure categories 0–0.34 : 1.00 (reference) 0.35–1.09 : 1.45 (1.09 - 1.94) 1.10–2 : 1.17 (0.89 - 1.53) 3–7 : 1.49 (1.15 - 1.92) 8–23 : 1.32 (1.02 - 1.70) 24–49 : 1.44 (1.07 - 1.94) 50–99 : 1.84 (1.28 - 2.64) 100–623 : 1.47 (0.99 - 2.20) | 8 | Moderate |
|  |  |  |  |  | Hypertensive disease mortality | 49 | ERR (95%CI) per 100 WLM ; p_value in males : 0.13 (< -0.81 - 3.38) ; p = 0.82 |  |  |
| Rage et al. 2018 (34) France 1946 - 2007 (34.7) | Cohort study | French male uranium miners n = 5400 Mean age at enrolment (min - max) into the cohort : 29.0 (16.0–68.5) Mean age at exit (min - max) : 63.6 (18.0–85.1) | Retrospective expert radon exposure reconstruction, worksite ambiante radon gas concentration measurement, measurement through individual dosimeters. Exposure period : in the main cohort : 1946 - 1990 in the 1977 post-JOUAC cohort : 1957 - 2001 Mean radon (se) in WLM : 35.1 (69.9) ; max = 960.1 WLM | For SMRs : Calendar period, attained age, sex, duration of employment, cumulative radon exposur For ERR : calendaryear and attained age | All circulatory system disease mortality | 446 | ERR (95%CI) per 100 WLM increase : 0.10 (-0.04 - 0.29) ; p = 0.16 | 6 | Moderate |
|  |  |  |  |  | Ischemic heart disease mortality | 169 | ERR per 100 WLM increase : 0.09 ; p=0.46 |  |  |
|  |  |  |  |  | Cerebroveascular disease mortality | 105 | SMR (95%CI) : 0.70 (0.46 - 1.02) ERR (95%CI) per 100 WLM increase : 0.42 (0.04 - 1.04) ; p = 0.02 |  |  |
| Nusinovici et al. 2010 (35) France 1946 - 1999 (30.1) | Cohort study | French male uranium miners n = 5086 Mean age at hire (range) : 27.8 (15.0–67.4) | Retrospective expert radon exposure reconstruction, worksite ambient radon gas concentration measurement, and measurement through individual dosimeters. Exposure period : 1946 - 1990 mean cumulative radon exposure in WLM : 36.6 | For SMR : Attained age, calendar year, duration of employment, age at first employment For internal ERR : Attained age, calendar year | All circulatory system mortality | 319 | External ERR (95%CI) per 100 WLM increase : 0.10 (-0.04 - 0.29) RR (95%CI) : 0.92 (0.72 - 1.19) | 6 | Moderate |
|  |  |  |  |  | Ischemic heart disease mortality | 125 | External ERR (95%CI) per 100 WLM increase : 0.013 (ND - 0.28) RR (95%CI) : 0.90 (0.60 - 1.35) |  |  |
|  |  |  |  |  | Cerebrovascular mortality | 80 | External ERR (95%CI) per 100 WLM increase : 0.492 (0.07 - 1.23) SMRs and RRs across radon exposure categories indicate no statistically significant association, but a significantly positive trend |  |  |
| Drubay et al. 2015 (36) France 1956 - 2007 (34.7) | Case-cohort study | French male uranium miners n = 313 Age at first employment, mean (SD) : 26.0 (5.6) Mean age at hire (range) : 27.8 (15.0–67.4) | Retrospective expert radon exposure reconstruction, worksite ambient radon gas concentration measurement, and measurement through individual dosimeters. Exposure period : 1956 - 1990 Mean cumulative radon exposure in WLM (SD) : 22.2 (28.0) | For all CSD : High-resting heart rate, smoking status (Never smoker, Former smoker, Current smoker, hypertriglyceridemia For IHD : hypertension, high-resting heart rate and diabetes For CeVD : hypertension and high gamma glutamyl transpeptidase GGT | All Circulatory system disease mortality | 76/313 | Hazard Ratio, HR (95%CI) per 100 WLM increase in cumulative radon exposure : 1.44 (0.66 - 3.14) | 7 | Moderate |
|  |  |  |  |  | Ischemic heart disease mortality | 26 | HR (95%CI) : 0.85 (0.21 - 3.49) |  |  |
|  |  |  |  |  | Cerebrovascular disease mortality | 16 | HR (95%CI) : 2.12 (0.48 - 9.32) |  |  |
| Golden et al. 2021 (37) Canada & USA | Pooled cohort study | Uranium processing facility workers n = 12,403 (11,059 males and 1344 females) | _ | _ | Dementia and Alzheimer’s disease mortality | 101 | SMR (95%CI) in males : 1.29 (1.04 - 1.5) in females : 0.98 (0.20 - 1.76) | 6 | Moderate |
|  |  |  |  |  |  |  |  |  |  |
|  |  |  |  |  | All Circulatory system disease mortality | 16,921 | SMR (95%CI) : 0.88 (0.86 - 0.89) |  |  |
|  |  |  |  |  | Ischemic heart disease mortality | 9457 | SMR (95%CI) : 0.92 (0.91 - 0.94) |  |  |
|  |  |  |  |  | Chronic obstructive pulmonary disease mortality | 1729 | SMR (95%CI) : 0.98 (0.93 - 1.02) |  |  |
|  |  |  |  |  | Digestive System diseases mortality | 2480 | SMR (95%CI) : 0.93 (0.89 - 0.96) |  |  |
|  |  |  |  |  | Cirrhosis mortality | 1415 | SMR (95%CI) : 0.99 (0.94 - 1.05) |  |  |
| Silver et al. 2013 (38) USA 1951 -2004 (37) | Cohort study | Fernald Feed Materials Production Center workers n = 6409 Mean age at first hire : 30.1 | Exposure period : 1951 - 1985 | Gender, race, age, calendar period, pay code (hourly/salaried) and birth year (spline terms) and consider all radiologic exposures simultaneously, non-radiological exposure (occupational exposure to acids) | Chronic obstructive pulmonary disease mortality | 115 | SMR (95%CI) : in hourly males : 1.01 (0.81 - 1.25) in salaried males : 0.43 (0.25 - 0.69) in hourly females : 0.00 (0.00 - 2.15) in salaried females : 1.29 (0.64 - 2.31)  HR at 10 WLM, ERR per 10 WLM, ERRs across radon exposure categories indicate no statistically significant association. | 8 | High |
|  |  |  |  |  | Chronic and unspecified nephritis and renal failure mortality | 21 | SMR (95%CI) : in hourly males : 0.83 (0.44 - 1.41) in salaried males : 0.85 (0.31 - 1.85) in hourly females : 2.29 (0.06 - 12.8) in salaried females : 0.61 (0.02 - 3.41) |  |  |
| Boice et al. 2008 (39) USA 1979 - 2005 | Cohort study | Uranium miners or millers with mining experience n = 2745 (2500 males, 245 females) | Exposure period : 1942 - 1996 across studies | Sex, age, and calendar year | Diabetes mellitus mortality | 20 | SMR (95%CI) : 1.20 (0.74 - 1.86) | 8 | Moderate |
|  |  |  |  |  | Mental and behavioural disorders mortality | 9 | SMR (95%CI) : 1.10 (0.50 - 2.08) |  |  |
|  |  |  |  |  | Cerebrovascular disease mortality | 32 | SMR (95%CI) : 0.98 (0.67 - 1.38) |  |  |
|  |  |  |  |  | All heart disease mortality | 218 | SMR (95%CI) : 0.93 (0.81 - 1.06) |  |  |
|  |  |  |  |  | Bronchitis, emphysema, asthma mortality | 35 | SMR (95%CI) : 1.78 (1.24 - 2.48) |  |  |
|  |  |  |  |  | Nephritis and nephrosis mortality | 6 | SMR (95%CI) : 0.86 (0.32 - 1.87) |  |  |
| Veiga et al. 2006 (40) Brazil 1942 - 1997 | Cohort study | Coal miners (underground, surface workers) n = 2856 | Exposure period : 1942 -1997 | Age, calendar period, sex | Endocrine and blood diseases mortality | 19 | SMR (95%CI) : 1.28 (0.82 - 2.01) | 7 | Moderate |
|  |  |  |  |  | Diabetes mortality | 15 | SMR (95%CI) : 1.14 (0.69 - 1.90) |  |  |
|  |  |  |  |  | All circulatory system disease mortality | 167 | SMR (95%CI) : 1.00 (0.86 - 1.17) |  |  |
|  |  |  |  |  | Hypertensive disease mortality | 12 | SMR (95%CI) : 1.02 (0.56 - 1.76) |  |  |
|  |  |  |  |  | Ischemic heart disease mortality | 59 | SMR (95%CI) : 0.97 (0.76 - 1.26) |  |  |
|  |  |  |  |  | Cerebrovascular disease mortality | 61 | SMR (95%CI) : 0.71 (0.71 - 1.18) |  |  |
|  |  |  |  |  | Chronic respiratory disease mortality | 9 | SMR (95%CI) : 0.49 (0.25 - 0.95) |  |  |
|  |  |  |  |  | Digestive system mortality | 16 | SMR (95%CI) : 0.53 (0.32 - 0.87) |  |  |
|  |  |  |  |  | Cirrhosis mortality | 9 | SMR (95%CI) : 0.55 (0.28 - 1.06) |  |  |
|  |  |  |  |  | Genital urinary diseases mortality | 4 | SMR (95%CI) : 0.72 (0.27 - 1.92) |  |  |
| Cocco et al. 1994 (41) Italy 1960 - 1988 | Cohort study | Sardinian and zinc miners n = 4740 | Exposure period : 1932 - 1971 | Age, calendar year | Diabetes mortality | 9 | SMR (95%CI) : 0.38 (0.17 - 0.72) | 7 | Moderate |
|  |  |  |  |  | All circulatory system disease mortality | 258 | SMR (95%CI) : 0.63 (0.56 - 0.72) |  |  |
|  |  |  |  |  | Digestive diseases mortality | 85 | SMR (95%CI) : 0.72 (0.58 - 0.89) |  |  |
|  |  |  |  |  | Cirrhosis mortality | 46 | SMR (95%CI) : 0.57 (0.41 - 0.76) |  |  |
|  |  |  |  |  | Urinary organs disease mortality | 29 | SMR (95%CI) : 1.60 (1.07 - 2.29) |  |  |
| Golden et al. 2019 (42) USA 1942 - 2012 (43.3) | Cohort study | Uranium processing workers n = 2514 white males Mean age at start of follow-up = 30.3 (range 16.1–65.3). | Exposure period : 1942 - 1966 | Age, sex, calendar year, race | Diabetes mortality | 27 | SMR (95%CI) : 0.65 (0.43 - 0.94) | 8 | High |
|  |  |  |  |  | Mental and Behavioral Disorders mortality | 33 | SMR (95%CI) : 1.04 (0.72 - 1.47) |  |  |
|  |  |  |  |  | Nervous system disease mortality | 72 | SMR (95%CI) : 1.14 (0.89 - 1.43) |  |  |
|  |  |  |  |  | Cerebrovascular disease mortality | 114 | SMR (95%CI) : 1.03 (0.85 - 1.24) |  |  |
|  |  |  |  |  | All Heart Disease mortality | 648 | SMR (95%CI) : 0.89 (0.82 - 0.96) |  |  |
|  |  |  |  |  | Ischemic heart disease mortality | 521 | SMR (95%CI) : 0.92 (0.84 - 1.00) |  |  |
|  |  |  |  |  | Bronchitis, Emphysema, Asthma mortality | 57 | SMR (95%CI) : 0.85 (0.65 - 1.10) |  |  |
|  |  |  |  |  | Cirrhosis of liver mortality | 32 | SMR (95%CI) : 0.91 (0.62 - 1.28) |  |  |
|  |  |  |  |  | Nephritis and Nephrosis mortality | 31 | SMR (95%CI) : 1.27 (0.86 - 1.80) |  |  |
|  |  |  |  |  | Myelodysplastic Syndrome mortality | 2 | SMR (95%CI) : 0.74 (0.08 - 2.69) |  |  |
|  |  |  |  |  | Polycythemia Vera mortality | _ | SMR (95%CI) : 0.74 (0.08 - 2.69) |  |  |
|  |  |  |  |  | Dementia, Alzheimer’s disease mortality | 50 | SMR (95%CI) : 1.18 (0.88 - 1.55) |  |  |
|  |  |  |  |  | Dementia, Alzheimer’s, Parkinson’s & Motor Neuron Diseases mortality | 71 | SMR (95%CI) : 1.17 (0.91 - 1.48) |  |  |
| Tomasek et al. 1994 (43) Czech Republic 1948 - 1991 | Cohort study | Underground uranium miners in West Bohemia n = 4320 Mean age at start of follow-up = 30.3 (range 16.1–65.3). | Exposure period : 1948 - 1959 Mean cumulative radon exposure : 219 WLM | Age | Mental disorders mortality | 14 | SMR : 5.18 ; p < 0.001 | 7 | Moderate |
|  |  |  |  |  | Rheumatic fever and rheumatic heart disease mortality | 13 | SMR : 0.64 ; p > 0.05 |  |  |
|  |  |  |  |  | Other digestive diseases (excluding Cirrhosis of liver) | 37 | SMR : 0.94 ; p > 0.05 |  |  |
|  |  |  |  |  | Urinary diseases mortality | 23 | SMR : 0.77 ; p > 0.05 |  |  |
| Ahlman et al. 1991 (44) Finland 1968 - 1986 | Cohort study | Sulfide ore miners (only men) n = 597 | Exposure period : 1954 - 1973 | Age, sex | All circulatory system disease mortality | 55 | SMR : 1.78 ; p < 0.01 | 6 | Moderate |
|  |  |  |  |  | Ischemic heart disease mortality | 44 | SMR : 1.99 ; p < 0.01 |  |  |
| Hodgson et al. 1990 (45) UK 1941 - 1987 | Cohort study | Tin miners (underground, surface, intermediate), mortality n = 3011 | Exposure period : 1941 - 1985 | Age, calendar period | Ischemic heart disease mortality | 208 | SMR : 1.07 ; p > 0.05 | 6 | Moderate |
|  |  |  |  |  |  |  |  |  |  |
| Xiang-Zhen et al. 1993 (46) China 1976 - 1988 | Cohort study | Tin miners n = 17,143 males and 2,795 females | Mean radon exposure among exposed miners In males : 275.4 WLM In females : 66.5 | Age, sex | Ischemic heart disease mortality | 47 | No apparent linear trend observed | 6 | Moderate |
|  |  |  |  |  | Rheumatic heart disease mortality | 15 | No apparent linear trend observed |  |  |
|  |  |  |  |  | Pulmonary heart disease mortality | 263 | No apparent linear trend observed |  |  |
|  |  |  |  |  | Cerebrovascular disease mortality | 302 | Relative risks across radon exposure tertiles indicate apparently increasing linear trend |  |  |
|  |  |  |  |  | Other circulatory system disease mortality | 108 | No apparent linear trend observed |  |  |
|  |  |  |  |  | Chronic bronchitis mortality | 19 | No apparent linear trend observed |  |  |

pCi/l: picoCurie/liter; CI: Confidence interval; Bq/m3 : Becquerel per cubic meter; NA= Not available; OR: Odds ratio; CrI: Credibility interval; GP: general population;BMI: Body mass index; SD: Standard deviation; IQR: Interquartile range; COPD: Chronic obstructive pulmonary disease; FEV1: Forced expiratory volume in one second; ERR: excess relative risk; SMR: Standardize mortality ratio; WLM: Working level month; SIR: Standardize incidence ratio; CSD: circulatory system disease; ND: Not disponible; CSD: Circulatory system disease; IHD: Ischemic heart disease; CeVD: Cerebrovascular disease; HR: Hazard ratio

**References**

1. Langlois PH, Lee M, Lupo PJ, Rahbar MH, Cortez RK. RESIDENTIAL RADON AND BIRTH DEFECTS: A POPULATION-BASED ASSESSMENT. Birth Defects Res A Clin Mol Teratol. janv 2016;106(1):5‑15.

2. Zlobina A, Farkhutdinov I, Carvalho FP, Wang N, Korotchenko T, Baranovskaya N, et al. Impact of Environmental Radiation on the Incidence of Cancer and Birth Defects in Regions with High Natural Radioactivity. International Journal of Environmental Research and Public Health. janv 2022;19(14):8643.

3. Mukharesh L, Greco KF, Banzon T, Koutrakis P, Li L, Hauptman M, et al. Environmental radon and childhood asthma. Pediatr Pulmonol. déc 2022;57(12):3165‑8.

4. Sheehan A, Freni Sterrantino A, Fecht D, Elliott P, Hodgson S. Childhood type 1 diabetes: an environment-wide association study across England. Diabetologia. 2020;63(5):964‑76.

5. Kim SH, Park JM, Kim H. The prevalence of stroke according to indoor radon concentration in South Koreans: Nationwide cross section study. Medicine (Baltimore). janv 2020;99(4):e18859.

6. Ruano-Ravina A, Cameselle-Lago C, Torres-Durán M, Pando-Sandoval A, Dacal-Quintas R, Valdés-Cuadrado L, et al. Indoor Radon Exposure and COPD, Synergic Association? A Multicentric, Hospital-Based Case-Control Study in a Radon-Prone Area. Arch Bronconeumol. oct 2021;57(10):630‑6.

7. Pando-Sandoval A, Ruano-Ravina A, Torres-Durán M, Dacal-Quintas R, Valdés-Cuadrado L, Hernández-Hernández JR, et al. Residential radon and characteristics of chronic obstructive pulmonary disease. Sci Rep. 26 janv 2022;12:1381.

8. Barbosa-Lorenzo R, Ruano-Ravina A, Ramis R, Aragonés N, Kelsey KT, Carballeira-Roca C, et al. Residential radon and COPD. An ecological study in Galicia, Spain. Int J Radiat Biol. févr 2017;93(2):222‑30.

9. Mauriz-Barreiro V, Barreiro-de Acosta M, Bastón-Rey I, Ferreiro-Iglesias R, Calviño-Suárez C, Barros-Dios JM, et al. Radon exposure and inflammatory bowel disease in a radon prone area. Rev Esp Enferm Dig. juill 2022;114(7):405‑9.

10. Abaszadeh Fathabadi Z, Ehrampoush MH, Mirzaei M, Mokhtari M, Nadi Sakhvidi M, Rahimdel A, et al. The relationship of indoor radon gas concentration with multiple sclerosis: a case-control study. Environ Sci Pollut Res. 1 mai 2020;27(14):16350‑61.

11. Schwartz GG, Klug MG. Motor neuron disease mortality rates in U.S. states are associated with well water use. Amyotrophic Lateral Sclerosis and Frontotemporal Degeneration. 16 nov 2016;17(7‑8):528‑34.

12. Groves-Kirkby CJ, Denman AR, Campbell J, Crockett RGM, Phillips PS, Rogers S. Is environmental radon gas associated with the incidence of neurodegenerative conditions? A retrospective study of multiple sclerosis in radon affected areas in England and Wales. J Environ Radioact. avr 2016;154:1‑14.

13. Boice JD, Cohen SS, Mumma MT, Chadda B, Blot WJ. Mortality among residents of Uravan, Colorado who lived near a uranium mill, 1936-84. J Radiol Prot. sept 2007;27(3):299‑319.

14. Boice JD, Mumma MT, Blot WJ. Cancer and Noncancer Mortality in Populations Living Near Uranium and Vanadium Mining and Milling Operations in Montrose County, Colorado, 1950–2000. rare. juin 2007;167(6):711‑26.

15. Suzuki Y, Honjo S, Kawamura H, Koishi F, Suzuki T, Hirohata T. Cancer Mortality in Low Radon Spa Area. Jpn J Cancer Res. nov 1994;85(11):1063‑6.

16. Lehrer S, Rheinstein PH, Rosenzweig KE. Association of Radon Background and Total Background Ionizing Radiation with Alzheimer’s Disease Deaths in U.S. States. Journal of Alzheimer’s Disease. 1 janv 2017;59(2):737‑41.

17. Neilson S, Robinson I, Rose FC. Ecological correlates of motor neuron disease mortality: a hypothesis concerning an epidemiological association with radon gas and gamma exposure. J Neurol. 1 avr 1996;243(4):329‑36.

18. Bølviken B, Celius EG, Nilsen R, Strand T. Radon: A Possible Risk Factor in Multiple Sclerosis. Neuroepidemiology. 13 janv 2003;22(1):87‑94.

19. Kelly-Reif K, Sandler DP, Shore D, Schubauer-Berigan M, Troester M, Nylander-French L, et al. Lung and extrathoracic cancer incidence among underground uranium miners exposed to radon progeny in the Příbram region of the Czech Republic: a case-cohort study. Occup Environ Med. févr 2022;79(2):102‑8.

20. Schubauer-Berigan MK, Daniels RD, Pinkerton LE. Radon exposure and mortality among white and American Indian uranium miners: an update of the Colorado Plateau cohort. Am J Epidemiol. 15 mars 2009;169(6):718‑30.

21. Roscoe RJ. An update of mortality from all causes among white uranium miners from the Colorado plateau study group. American Journal of Industrial Medicine. 1997;31(2):211‑22.

22. Kelly-Reif K, Sandler DP, Shore D, Schubauer-Berigan M, Troester MA, Nylander-French L, et al. Mortality and cancer incidence among underground uranium miners in the Czech Republic 1977-1992. Occup Environ Med. août 2019;76(8):511‑8.

23. Villeneuve PJ, Morrison HI, Volesky K, Lane RSD. Circulatory system disease mortality and occupational exposure to radon progeny in the cohort of Newfoundland Fluorspar Miners between 1950 and 2016. Int Arch Occup Environ Health. 1 avr 2023;96(3):411‑8.

24. Navaranjan G, Berriault C, Do M, Villeneuve PJ, Demers PA. Cancer incidence and mortality from exposure to radon progeny among Ontario uranium miners. Occup Environ Med. déc 2016;73(12):838‑45.

25. Zeng X, Berriault C, Arrandale VH, DeBono NL, Harris MA, Demers PA. Radon exposure and risk of neurodegenerative diseases among male miners in Ontario, Canada: A cohort study. Am J Ind Med. févr 2023;66(2):132‑41.

26. Lane RSD, Frost SE, Howe GR, Zablotska LB. Mortality (1950–1999) and Cancer Incidence (1969–1999) in the Cohort of Eldorado Uranium Workers. rare. oct 2010;174(6a):773‑85.

27. Zablotska LB, Lane RSD, Frost SE. Mortality (1950–1999) and cancer incidence (1969–1999) of workers in the Port Hope cohort study exposed to a unique combination of radium, uranium and γ-ray doses. BMJ Open. 1 janv 2013;3(2):e002159.

28. Kreuzer M, Sogl M, Brüske I, Möhner M, Nowak D, Schnelzer M, et al. Silica dust, radon and death from non-malignant respiratory diseases in German uranium miners. Occup Environ Med. 1 déc 2013;70(12):869‑75.

29. Kreuzer M, Grosche B, Schnelzer M, Tschense A, Dufey F, Walsh L. Radon and risk of death from cancer and cardiovascular diseases in the German uranium miners cohort study: follow-up 1946–2003. Radiat Environ Biophys. 1 mai 2010;49(2):177‑85.

30. Kreuzer M, Kreisheimer M, Kandel M, Schnelzer M, Tschense A, Grosche B. Mortality from cardiovascular diseases in the German uranium miners cohort study, 1946–1998. Radiat Environ Biophys. 1 sept 2006;45(3):159‑66.

31. Kreuzer M, Dufey F, Laurier D, Nowak D, Marsh JW, Schnelzer M, et al. Mortality from internal and external radiation exposure in a cohort of male German uranium millers, 1946–2008. International Archives of Occupational and Environmental Health. mai 2015;88(4):431‑41.

32. Kreuzer M, Deffner V, Schnelzer M, Fenske N. Mortality in Underground Miners in a Former Uranium Ore Mine–Results of a Cohort Study Among Former Employees of Wismut AG in Saxony and Thuringia. Dtsch Arztebl Int. 29 janv 2021;118(4):41‑8.

33. Zablotska LB, Fenske N, Schnelzer M, Zhivin S, Laurier D, Kreuzer M. Analysis of mortality in a pooled cohort of Canadian and German uranium processing workers with no mining experience. Int Arch Occup Environ Health. 1 janv 2018;91(1):91‑103.

34. Rage E, Caër-Lorho S, Laurier D. Low radon exposure and mortality among Jouac uranium miners An update of the French cohort (1946-2007). Journal of Radiological Protection. mars 2018;38(1):92‑108.

35. Nusinovici S, Vacquier B, Leuraud K, Metz-Flamant C, Caër-Lorho S, Acker A, et al. Mortality from circulatory system diseases and low-level radon exposure in the French cohort study of uranium miners, 1946-1999. Scandinavian Journal of Work, Environment and Health. sept 2010;36(5):373‑83.

36. Drubay D, Caër-Lorho S, Laroche P, Laurier D, Rage E. Mortality from Circulatory System Diseases among French Uranium Miners A Nested Case-Control Study. Radiation Research. mai 2015;183(5):550‑62.

37. Golden AP, Milder CM, Ellis ED, Anderson JL, Boice Jr. JD, Bertke SJ, et al. Cohort profile: four early uranium processing facilities in the US and Canada. International Journal of Radiation Biology. 3 juin 2021;97(6):833‑47.

38. Silver SR, Bertke SJ, Hein MJ, Daniels RD, Fleming DA, Anderson JL, et al. Mortality and ionising radiation exposures among workers employed at the Fernald Feed Materials Production Center (1951–1985). Occup Environ Med. 1 juill 2013;70(7):453‑63.

39. Jr JDB, Cohen SS, Mumma MT, Chadda B, Blot WJ. A cohort study of uranium millers and miners of Grants, New Mexico, 1979–2005. J Radiol Prot. août 2008;28(3):303.

40. Veiga LHS, Amaral ECS, Colin D, Koifman S. A retrospective mortality study of workers exposed to radon in a Brazilian underground coal mine. Radiat Environ Biophys. 1 juill 2006;45(2):125‑34.

41. Cocco PL, Carta P, Belli S, Picchiri GF, Flore MV. Mortality of Sardinian lead and zinc miners: 1960-88. Occupational and Environmental Medicine. 1 oct 1994;51(10):674‑82.

42. Golden AP, Ellis ED, Cohen SS, Mumma MT, Leggett RW, Wallace PW, et al. Updated mortality analysis of the Mallinckrodt uranium processing workers, 1942–2012. International Journal of Radiation Biology. 3 avr 2022;98(4):701‑21.

43. Tomásek L, Swerdlow AJ, Darby SC, Placek V, Kunz E. Mortality in uranium miners in west Bohemia: a long-term cohort study. Occup Environ Med. mai 1994;51(5):308‑15.

44. Ahlman K, Koskela RS, Kuikka P, Koponen M, Annanmäki M. Mortality among sulfide ore miners. Am J Ind Med. 1991;19(5):603‑17.

45. Hodgson JT, Jones RD. Mortality of a cohort of tin miners 1941-86. Occupational and Environmental Medicine. 1 oct 1990;47(10):665‑76.

46. Xiang-Zhen X, Lubin JH, Jun-Yao L, Li-Fen Y, Sheng LQ, Lan Y, et al. A Cohort Study in Southern China of Tin Miners Exposed to Radon and Radon Decay Products. Health Physics. févr 1993;64(2):120.
