# Supplementary material for: Radon exposure and potential health effects other than lung cancer: a systematic review and meta-analysis
Source: Front Public Health. 2024 Sep 25;12:1439355. doi: 10.3389/fpubh.2024.1439355 (PMC11461271; doi:10.3389/fpubh.2024.1439355)
Supplement: Supplementary file 8 [file Table_5.DOCX]

Supplementary Material

The summary tables only include single diseases for simplicity purpose, diseases’ subtypes and large group of diseases were thus excluded. Note that in case the risk measure or the standardize incidence or mortality ratio were estimated across radon exposure categories, or study population subgroups (by gender, race, payroll study etc …), studies were classified in negative (-) or positive (+) association when a significant association was reported at least for one of the categories or subgroups, depending on the direction of the association.

Table S4. Summary of results based on studies that provided standardize incidence and/mortality ratio (SIR/SMR), number of studies (references)

| Cancer | Children in the general population | | | | | | Adults in the general population | | | | | | Mine workers | | | | | | | |
| --- | --- | --- | --- | --- | --- | --- | --- | --- | --- | --- | --- | --- | --- | --- | --- | --- | --- | --- | --- | --- |
|  | Morbidity | | | Mortality | | | Morbidity | | | Mortality | | | Incidence | | | | Mortality | | | |
|  | No | (-) | (+) | No | (-) | (+) | No | (-) | (+) | No | (-) | (+) | No | (-) | (+) | No | | (-) | (+) |  |
| All leukaemia | 1(1) |  |  | 2(1,2) |  |  | 2(1,3) |  |  | 3(1,2,4) | 1(2) |  | 2(5,6) |  | 1(7) | 5(8–12) | |  |  |  |
| CLL |  |  |  |  |  |  | 1(1) |  |  | 3(1,2,4) |  |  |  | 1(5) |  | 3(5,11,13) | |  |  |  |
| Non-CLL |  |  |  |  |  |  | 1(1) |  |  | 2(1,4) | 1(2) |  | 1(5) |  |  | 5(5,11,13–15) | |  |  |  |
| ALL |  | 2(16,17) | 1(16) |  |  |  |  |  |  | 1(18) |  |  |  |  |  |  | |  |  |  |
| Lymphoblastic leukaemia |  |  |  |  |  |  |  |  |  |  |  |  | 1(7) |  |  | 1(7) | |  |  |  |
| Myeloid Leukaemia |  |  |  |  |  |  |  |  |  |  |  |  | 1(7) |  |  | 2(7,14) | |  |  |  |
| AML | 1(16) |  | 1(17) |  |  |  |  |  |  |  |  | 1(18) |  |  |  | 1(10) | |  |  |  |
| CML |  |  |  |  |  |  |  |  |  |  |  |  |  |  |  |  | |  |  |  |
| AL | 1(16) | 1(17) | 1(16) |  |  |  |  |  |  |  |  |  |  |  |  |  | |  |  |  |
| All lymphoma |  |  |  |  |  |  | 1(3) |  |  |  |  |  |  |  |  |  | |  |  |  |
| NHL |  |  |  |  |  |  | 1(1) |  |  | 3(1,2,4) |  |  | 1(6) | 1(5) |  | 5(8,10,11,19,20) | |  |  |  |
| HL |  |  |  |  |  |  | 1(1) |  |  | 3(1,2,4) |  |  | 2(6,7) | 1(5) |  | 4(8,10,11,19) | |  |  |  |
| Multiple myeloma |  |  |  |  |  |  | 1(1) |  |  | 3(1,2,4) |  |  | 3(5–7) |  |  | 4(8,10,11,19) | |  |  |  |
| Brain & CNS | 1(21) |  |  |  |  |  |  |  |  | 2(1,2) | 1(4) |  | 1(22) |  |  | 2(10,20) | | 1(8) | 1(11) |  |
| Brain |  |  |  |  |  |  | 1(1) |  |  |  |  |  | 2(7,22) | 1(5) |  | 2(7,22) | |  |  |  |
| Bone cancer |  |  |  |  |  |  | 1(1) |  |  | 3(1,2,4) |  |  | 1(5) |  |  | 7(5,10,11,13,14,23,24) | |  |  |  |
| Connective & other soft tissue |  |  |  |  |  |  |  |  |  | 2(1,2) |  |  | 1(7) | 1(5) |  | 6(5,7,10,11,14,24) | |  |  |  |
| Malignant melanoma |  |  |  |  |  |  |  | 1(1) |  | 3(1,2,4) |  |  | 2(7,22) | 1(5) |  | 7(7,10,11,13,14,23,24) | | 1(5) |  |  |
| NMSC |  |  |  |  |  |  |  |  |  | 1(4) |  |  |  | 1(7) |  | 5(7,10,14,23,24) | |  |  |  |
| Skin |  |  |  |  |  |  |  |  |  | 1(2) |  |  |  |  |  | 1(8) | |  |  |  |
| Nose |  |  |  |  |  |  | 1(3) |  |  |  |  |  |  |  |  | 4(10,14,15,24) | |  |  |  |
| Lip |  |  |  |  |  |  |  |  |  |  |  |  | 2(7,22) |  |  |  | |  |  |  |
| Tongue |  |  |  |  |  |  |  |  |  |  |  |  | 1(7) |  |  | 1(7) | |  |  |  |
| Larynx |  |  |  |  |  |  | 1(3) |  |  |  |  |  | 3(5,7,22) |  |  | 5(8–11,20) | |  |  |  |
| Oropharynx |  |  |  |  |  |  |  |  |  |  |  |  | 1(7) |  |  | 1(7) | |  |  |  |
| Salivary gland |  |  |  |  |  |  |  |  |  |  |  |  |  |  |  | 2(10,24) | |  |  |  |
| Tongue and month |  |  |  |  |  |  |  |  |  |  |  |  |  |  |  | 2(10,24) | |  |  |  |
| Pharynx |  |  |  |  |  |  |  |  |  |  |  |  |  |  |  | 1(10) | | 1(8) |  |  |
| Buccal cavity |  |  |  |  |  |  |  |  |  |  |  |  |  |  |  |  | | 1(8) |  |  |
| Buccal cavity and pharynx |  |  |  |  |  |  | 1(3) |  |  | 1(4) |  |  | 1(7) | 1(5) |  | 8(7,11,13–15,20,23,25) | | 1(5) |  |  |
| Oesophagus |  |  |  |  |  |  | 2(1,3) |  |  | 3(1,2,4) |  |  | 3(5,7,22) |  |  | 6(8–11,20,26) | |  |  |  |
| Stomach |  |  |  |  |  |  | 1(1) | 1(3) |  | 3(2–4) | 1(27) | 1(1) | 1(22) | 1(5) | 1(7) | 5(9,12,19,20,26) | | 1(11) | 2(8,10) |  |
| Liver |  |  |  |  |  |  | 2(1,3) |  |  | 1(3) | 1(27) |  |  | 1(5) | 1(7) | 5(5,10,15,20,26) | |  | 3(7,14,24) |  |
| Gallbladder |  |  |  |  |  |  | 1(3) |  |  |  |  |  | 1(7) |  |  | 4(7,10,14,25) | |  | 1(24) |  |
| Liver & gallbladder |  |  |  |  |  |  |  |  |  | 2(3,4) |  |  |  |  |  | 1(11) | |  | 1(8) |  |
| Pancreas |  |  |  |  |  |  | 2(1,3) |  |  | 4(1,2,4,28) |  |  | 3(5,7,22) |  |  | 4(8,10,11,20) | |  |  |  |
| Small intestine |  |  |  |  |  |  |  |  |  |  |  |  | 1(7) |  |  | 1(7) | |  |  |  |
| Intestine (small intestine + colon) |  |  |  |  |  |  |  |  |  |  |  |  |  |  |  | 2(10,19) | | 1(8) |  |  |
| Colon |  |  |  |  |  |  | 1(3) |  |  |  | 1(4) |  | 2(7,22) |  |  | 4(7,11,13,22) | | 1(25) |  |  |
| Rectum |  |  |  |  |  |  | 1(3) |  |  | 2(4,27) |  |  | 1(22) |  | 1(7) | 2(8,11) | |  | 1(10) |  |
| Colorectal |  |  |  |  |  |  | 1(1) |  |  | 3(1–3) |  |  |  | 1(5) |  | 1(9) | | 1(5) |  |  |
| Peritoneum and retroperitoneum |  |  |  |  |  |  |  |  |  | 1(28) |  |  |  |  |  |  | |  | 1(20) |  |
| Breast |  |  |  |  |  |  | 1(3) | 1(1) |  | 3(1,4,28) | 1(2) |  | 1(5) |  |  | 4(5,11,13,23) | |  |  |  |
| Cervix uteri |  |  |  |  |  |  | 1(1) |  |  | 2(1,2) |  |  |  |  |  |  | |  |  |  |
| Uterus corpus |  |  |  |  |  |  | 2(1,3) |  |  | 4(1,2,4,28) |  |  |  |  |  |  | |  |  |  |
| Ovary |  |  |  |  |  |  | 2(1,3) |  |  | 2(1,2) |  |  |  |  |  |  | |  |  |  |
| Bladder |  |  |  |  |  |  | 2(1,3) |  |  | 2(1,2) |  |  | 2(7,22) |  |  | 8(7,10,14,15,20,22,24,25) | |  |  |  |
| Bladder and other urinary organs |  |  |  |  |  |  |  |  |  | 1(4) |  |  | 1(22) | 1(5) |  | 1(11) | | 1(8) | 1(19) |  |
| Kidney |  |  |  |  |  |  |  |  |  | 1(4) |  |  |  | 2(5,22) |  | 4(8,10,11,19) | |  |  |  |
| Kidney, ureter, other urinary organs |  |  |  |  |  |  |  |  |  | 1(2) |  |  | 1(7) |  |  | 3(7,14,20) | | 1(25) | 1(15) |  |
| Prostate |  |  |  |  |  |  | 2(1,3) |  |  | 2(1,2) |  |  | 2(7,22) | 1(5) |  | 4(9–11,20) | | 1(8) |  |  |
| Testis |  |  |  |  |  |  |  |  |  |  |  |  | 1(7) | 1(5) |  | 5(5,7,10,14,24) | |  |  |  |
| Penis |  |  |  |  |  |  |  |  |  |  |  |  | 1(7) |  |  | 1(7) | |  |  |  |
| Thyroid gland |  |  |  |  |  |  | 1(1) |  |  | 2(1,2) |  |  | 1(7) | 1(5) |  | 5(5,7,10,14,24) | |  |  |  |

No: results suggesting no statistically significant association; (-): results suggesting a negative statistically significant association; (+) : results suggesting a positive statistically significant association; CLL : Chronic lymphocytic leukaemia; Non-CLL: Leukaemia, excluding CLL; ALL: Acute lymphocytic leukaemia; AML: Acute myeloid leukaemia; CML: Chronic myeloid leukaemia; AL: Acute leukaemia; NHL: Non-Hodgkin lymphoma; HL: Hodgkin lymphoma; CNS: Central nervous system; NMSC: Non-malignant skin cancer.

Table S5. Summary of results based on studies that provided standardize incidence and/mortality ratio (SIR/SMR), number of studies (references)

| Non-malignant diseases | Children in the general population | | | | | | Adults in the general population | | | | | | Mine workers | | | | | |
| --- | --- | --- | --- | --- | --- | --- | --- | --- | --- | --- | --- | --- | --- | --- | --- | --- | --- | --- |
|  | Morbidity | | | Mortality | | | Morbidity | | | Mortality | | | Incidence | | | Mortality | | |
|  | No | (-) | (+) | No | (-) | (+) | No | (-) | (+) | No | (-) | (+) | No | (-) | (+) | No | (-) | (+) |
| Metabolic disease |  |  |  |  |  |  |  |  |  |  |  |  |  |  |  | 1(14) |  |  |
| Diabetes mellitus |  |  |  |  |  |  |  |  |  | 2(2,4) |  |  |  |  | 1(23) | 6(7,9,13,20,22,23) | 1(11) |  |
|  |  |  |  |  |  |  |  |  |  |  |  |  |  |  |  |  |  |  |
| Bronchitis, emphysema, asthma |  |  |  |  |  |  |  |  |  | 2(2,4) |  |  |  |  |  | 1(11) |  | 1(13) |
| Asthma |  |  |  |  |  |  |  |  |  | 1(2) |  |  |  |  |  | (29) |  |  |
| Emphysema |  |  |  |  |  |  |  |  |  | 1(2) |  |  |  |  |  |  |  | 1(30) |
| Bronchitis |  |  |  |  |  |  |  |  |  | 1(2) |  |  |  |  |  |  |  |  |
| COPD |  |  |  |  |  |  |  |  |  |  |  |  |  |  |  | 2(8,19) |  |  |
| COPD and allied conditions including asthma |  |  |  |  |  |  |  |  |  |  |  |  |  |  |  | 1(7) | 1(22) |  |
| All CSD |  |  |  |  |  |  |  |  |  |  |  |  |  |  |  | 1(9) | 2(8,20,31) | 1(32) |
| IHD |  |  |  |  |  |  |  |  |  |  |  |  |  |  |  | 3(9,11,12) | 1(8) | 1(32) |
| AMI |  |  |  |  |  |  |  |  |  |  |  |  |  |  |  |  | 1(7) |  |
| Stroke/CeVD |  |  |  |  |  |  |  |  |  |  | 1(4) | 1(2) |  |  |  | 6(9,11,13,15,22,25) | 3(5,7,23) | 1(14) |
| Major CVD |  |  |  |  |  |  |  |  |  |  |  |  |  |  |  |  | 1(5) |  |
| All heart disease |  |  |  |  |  |  |  |  |  | 1(2) | 1(4) |  |  |  |  | 1(13) | 1(11) |  |
| Hypertension with & without heart disease |  |  |  |  |  |  |  |  |  |  |  |  | 1(23) |  |  | 2(9,23) |  | 1(22) |
| Rheumatic heart disease |  |  |  |  |  |  |  |  |  |  |  |  |  |  |  | 2(23,33) |  |  |
| Chronic disease of endocardium |  |  |  |  |  |  |  |  |  |  |  |  |  |  |  | 1(23) |  |  |
| Cardiomyopathy |  |  |  |  |  |  |  |  |  |  |  |  |  |  |  | 1(23) |  |  |
| Diseases of arteries, veins, lymphatic system |  |  |  |  |  |  |  |  |  |  |  |  |  |  |  |  | 1(23) |  |
| Atherosclerosis |  |  |  |  |  |  |  |  |  |  |  |  |  |  |  |  |  | 1(7) |
| Arteries, arterioles, capillaries, and allied conditions (Including atherosclerosis) |  |  |  |  |  |  |  |  |  |  |  |  |  |  |  |  |  | 1(7) |
| Blood and blood-forming organs disease |  |  |  |  |  |  |  |  |  |  |  |  |  |  |  | 1(7) |  | 1(23) |
| ALS |  |  |  |  |  |  |  |  |  |  |  |  |  |  |  | 1(14) |  |  |
| Dementias & Alzheimer disease |  |  |  |  |  |  |  |  |  |  |  |  |  |  |  | 1(11) |  | 1(34) |
| Mental & behavioural disorders |  |  |  |  |  |  |  |  |  | 1(4) |  |  |  |  |  | 3(11,13,14) | 1(23) | 2(7,33) |
| Nervous system disease |  |  |  |  |  |  |  |  |  | 1(4) |  |  |  |  |  | 4(7,11,22,23) | 1(14) | 1(13) |
| Digestive system mortality |  |  |  |  |  |  |  |  |  |  |  |  |  |  |  |  | 3(8,9,20) |  |
| Cirrhosis of liver |  |  |  |  |  |  |  |  |  | 1(2) | 1(4) |  |  |  |  | 3(8,9,11) | 1(20) |  |
| Stomach and duodenum disease |  |  |  |  |  |  |  |  |  |  |  |  |  |  |  |  | 1(7) |  |
| Pancreas disease |  |  |  |  |  |  |  |  |  |  |  |  |  |  |  | 1(7) |  |  |
| Kidney disease |  |  |  |  |  |  |  |  |  |  |  |  |  |  |  |  | 1(14) |  |
| Nephritis & nephrosis |  |  |  |  |  |  |  |  |  | 2(2,4) |  |  |  |  |  | 4(7,11,13,22) |  |  |
| Acute glomerulonephritis and acute renal failure |  |  |  |  |  |  |  |  |  |  |  |  |  |  |  |  |  | 1(23) |
| Chronic and unspecified nephritis and renal failure |  |  |  |  |  |  |  |  |  |  |  |  |  |  |  | 2(19,23) |  |  |
| Genitourinary organs disease |  |  |  |  |  |  |  |  |  |  |  |  |  |  |  | 3(9,25,33) | 1(7) |  |
| Systemic ESRD |  |  |  |  |  |  |  |  |  |  |  |  | 1(23) |  |  |  |  |  |
| Non-systemic ESRD |  |  |  |  |  |  |  |  |  |  |  |  | 1(23) |  |  |  |  |  |
| Skin and subcutaneous tissues diseases |  |  |  |  |  |  |  |  |  |  |  |  |  |  |  | 1(23) |  |  |
| Musculoskeletal and connective tissue diseases |  |  |  |  |  |  |  |  |  |  |  |  |  |  |  | 1(23) |  |  |

No: results suggesting no statistically significant association; (-): results suggesting a negative statistically significant association; (+) : results suggesting a positive statistically significant association; COPD: Chronic obstructive pulmonary disease; CSD: Circulatory system disease; IHD: Ischaemic heart disease; AMI: Acute myocardial infarction; CeVD: Cerebrovascular disease; CVD: Cardiovascular disease; ALS: Amyotrophic lateral sclerosis; ESRD: End-stage renal disease.

Table S6. Summary of results from studies providing radon related risk estimates, number of studies (references)

| Cancer | Children in the general population | | | | | | Adults in the general population | | | | | | Mine workers | | | | | |
| --- | --- | --- | --- | --- | --- | --- | --- | --- | --- | --- | --- | --- | --- | --- | --- | --- | --- | --- |
|  | Morbidity | | | Mortality | | | Morbidity | | | Mortality | | | Morbidity | | | Mortality | | |
|  | No | (-) | (+) | No | (-) | (+) | No | (-) | (+) | No | (-) | (+) | No | (-) | (+) | No | (-) | (+) |
| All leukaemia | 10 (35–44) |  | 1 (45) | 1 (2) |  | 1 (46) | 3 (44,47,48) |  |  | 3 (2,49,50) |  |  | 2(5,51) |  | 1(52) | 4(5,6,30,53) |  |  |
| CLL |  |  |  |  |  |  | 2 (48,54) |  |  | 1 (2) |  |  | 3(5,6,51) |  | 1(52) | 1(55) | 1(5) |  |
| Non-CLL |  |  |  |  |  |  |  |  |  | 1 (2) |  |  | 3(5,6,51) |  |  | 1(5) |  |  |
| ALL | 6(17,35,37,39,40,56) | 2 (57,58) | 3 (16,42,59) |  |  |  | 1 (48) |  | 1 (57) | 1 (18) |  |  |  |  |  |  |  |  |
| Lymphoblastic leukaemia | 1 (36) |  |  |  |  |  |  |  | 1(60) |  |  |  |  |  |  |  |  |  |
| Myeloid Leukaemia |  |  |  |  |  |  | 1(54) |  | 2(45,60) | 1(61) |  |  | 1(52) |  |  | 1(55) |  |  |
| AML | 7 (16,17,36,37,42,62,63) |  |  |  |  |  | 2(48,64) |  |  |  |  | 1(18) |  | 1(51) |  |  |  |  |
| CML |  |  |  |  |  |  | 1(48) |  |  |  |  |  |  |  |  |  |  |  |
| AL | 4 (16,65,66) | 1(17) |  |  |  |  | 1(67) |  |  |  |  |  |  |  |  |  |  |  |
| All lymphoma | 4 (36,37,42,68) |  |  | 1(46) |  |  |  |  |  |  |  |  | 1(6) |  |  | 1(6) |  |  |
| NHL | 4 (36,58,63,68) |  | 1 (44) |  |  |  |  |  | 1(44) | 3(2,49,50) |  |  | 2(6,52) |  |  | 4(6,23,53,55) |  |  |
| HL | 3 (36,58,68) |  |  |  |  |  | 1(54) |  |  | 2(2,14) |  |  | 2(6,52) |  |  | 2(6,55) |  |  |
| Multiple myeloma |  |  |  |  |  |  | 1(54) |  |  | 2(2,50) |  |  | 2(6,52) |  |  | 4(6,23,53,55) |  |  |
| Brain & CNS | 7 (21,35–37,40,42,58) |  | 2 (45,65) | 1 (46) |  |  |  |  |  | 2(2,50) |  |  |  |  |  | 2(15,53) |  |  |
| Brain |  |  |  |  |  |  |  |  |  | 1(69) |  | 2(49,70) |  |  |  | 1(22) |  |  |
| Bone cancer | 1 (37) |  |  | 1 (46) |  |  | 1(71) |  |  | 2(2,50) |  |  |  |  |  |  |  |  |
| Connective & other soft tissue |  |  |  | 1 (46) |  |  |  |  |  | 2(2,50) |  |  |  |  |  |  |  |  |
| Malignant melanoma |  |  | 1 (45) |  |  |  |  |  | 2(45,72) | 3(2,50,73) |  | 1(69) |  |  | 1(74) |  |  |  |
| NMSC |  |  |  |  |  |  | 1(75) |  | 1(72) | 2(69,73) |  |  |  |  |  |  |  |  |
| Skin |  |  |  |  |  |  |  |  |  | 2(2,73) |  |  |  |  |  |  |  |  |
| Nose |  |  |  |  |  |  |  |  |  |  |  |  |  |  |  | 1(76) |  |  |
| Larynx |  |  |  |  |  |  |  |  |  | 1(69) | 1(77) |  | 2(74,78) |  |  | 1(76) |  |  |
| Nasopharynx |  |  |  |  |  |  |  |  |  |  | 1(77) |  |  |  |  |  |  |  |
| Salivary gland |  |  |  |  |  |  | 1(79) |  |  |  |  |  |  |  |  |  |  |  |
| Tongue and month |  |  |  |  |  |  |  |  |  |  |  |  |  |  |  | 1(76) |  |  |
| Pharynx |  |  |  |  |  |  |  |  |  |  |  |  |  |  |  | 1(76) |  |  |
| Buccal cavity and pharynx |  |  |  |  |  |  |  |  |  | 1(49) | 1(77) |  | 1(80) | 1(74) |  |  |  |  |
| Esophagus |  |  |  |  |  |  | 1(72) |  |  | 4(2,49,50,69) | 1(77) | 1(81) |  |  |  | 1(53) |  |  |
| Stomach |  |  |  |  |  |  | 4(3,72,82,83) |  | 1(84) | 3(2,50,69) | 2(27,28) | 2(49,84) | 3(5,74,80) |  |  | 4(5,25,55,80) |  | 1(53) |
| Liver | 1 (37) |  |  |  |  |  |  |  |  | 2(27,50) |  |  | 1(74) |  |  | 2(53,55) |  |  |
| Gallbladder |  |  |  |  |  |  |  |  |  |  |  |  | 1(74) |  |  | 1(53) |  |  |
| Liver & gallbladder |  |  |  |  |  |  |  |  |  |  |  |  |  |  |  |  |  |  |
| Pancreas |  |  |  |  |  |  | 1(85) | 1(72) |  | 4(2,49,50,69) | 1(77) |  | 2(74,80) |  |  | 2(53,80) |  |  |
| Colon |  |  |  |  |  |  |  |  | 1(72) |  |  | 1(77) | 2(74,80) |  |  | 2(53,80) |  |  |
| Rectum |  |  |  |  |  |  |  | 1(72) |  |  |  |  | 2(74,80) |  |  | 2(22,53) |  |  |
| Colorectal |  |  |  |  |  |  |  |  |  | 5(2,27,28,49,69) |  | 1(50) |  |  |  |  |  |  |
| Breast |  |  |  |  |  |  | 2(72,86) |  |  | 4(2,49,50,77) |  |  |  |  |  | 1(87) |  |  |
| Cervix uteri |  |  |  |  |  |  | 1(88) | 1(72) |  | 3(2,50) |  |  |  |  |  |  |  |  |
| Uterus corpus |  |  |  |  |  |  | 1(88) |  |  | 2(2,50) |  |  |  |  |  |  |  |  |
| Ovary |  |  |  |  |  |  | 1(72) |  |  | 2(2,50) |  |  |  |  |  |  |  |  |
| Bladder |  |  |  |  |  |  | 2(72,89) |  |  | 3(2,49,69) | 1(77) |  | 2(74,80) |  |  | 1(22) |  |  |
| Bladder and other urinary organs |  |  |  |  |  |  |  |  |  |  |  |  |  |  |  | 1(53) |  |  |
| Kidney | 1 (37) |  |  | 1 (46) |  |  | 1(89) |  |  | 1(2) | 1(50) | 1(69) | 1(5) |  |  | 2(22,53) | 1(5) |  |
| Kidney and other urinary organs |  |  |  |  |  |  |  |  |  | 2(49) |  |  | 1(74) |  |  | 1(15) |  | 1(55) |
| Prostate |  |  |  |  |  |  |  |  | 2(72,88) | 2(2,69) |  | 1(77) | 2(74,80) |  |  | 2(53,80) |  |  |
| Testis |  |  |  |  |  |  | 1(88) |  |  |  |  |  |  |  |  |  |  |  |
| Thyroid gland |  |  |  |  |  |  | 2(90,91) |  |  | 3(2,49,50) |  |  |  |  |  |  |  |  |

No: results suggesting no statistically significant association; (-): results suggesting a negative statistically significant association; (+) : results suggesting a positive statistically significant association; CLL : Chronic lymphocytic leukaemia; Non-CLL: Leukaemia, excluding CLL; ALL: Acute lymphocytic leukaemia; AML: Acute myeloid leukaemia; CML: Chronic myeloid leukaemia; AL: Acute leukaemia; NHL: Non-Hodgkin lymphoma; HL: Hodgkin lymphoma; CNS: Central nervous system; NMSC: Non-malignant skin cancer

Table S7. Summary of results from studies providing radon related risk estimates, number of studies (references)

| Non-malignant diseases | Children in the general population | | | | | | Adults in the general population | | | | | | Mine workers | | | | | |
| --- | --- | --- | --- | --- | --- | --- | --- | --- | --- | --- | --- | --- | --- | --- | --- | --- | --- | --- |
|  | Morbidity | | | Mortality | | | Morbidity | | | Mortality | | | Morbidity | | | Mortality | | |
|  | No | (-) | (+) | No | (-) | (+) | No | (-) | (+) | No | (-) | (+) | No | (-) | (+) | No | (-) | (+) |
| Birth defect | 1(92) |  | 1(41) |  |  |  |  |  |  |  |  |  |  |  |  |  |  |  |
| Diabetes mellitus |  |  | 1(93) |  |  |  |  |  |  | 1 (2) |  |  |  |  |  | 1(80) |  |  |
| Bronchitis, emphysema, asthma |  |  |  |  |  |  |  |  |  | 1(2) |  |  |  |  |  |  |  |  |
| Asthma |  |  | 1(94) |  |  |  |  |  |  | 1(2) |  |  |  |  |  |  |  |  |
| COPD |  |  |  |  |  |  | 3(95–97) |  |  |  |  |  |  |  |  | 3(19,29,98) |  |  |
| All CSD |  |  |  |  |  |  |  |  |  |  |  | 1(27) |  |  |  | 4(15,22,31,99) |  |  |
| IHD |  |  |  |  |  |  |  |  |  |  |  |  |  |  |  | 4 (15,31,80,99) |  |  |
| AMI |  |  |  |  |  |  |  |  |  |  |  |  |  |  |  | 2(99,31) |  |  |
| Stroke/CeVD |  |  |  |  |  |  |  |  | 1(100) |  | 2 (2,27) |  |  |  |  | 3(15,31,99) | 1(80) |  |
| All heart disease |  |  |  |  |  |  |  |  |  | 1(2) |  |  |  |  |  |  |  |  |
| Hypertension with & without heart disease |  |  |  |  |  |  |  |  |  |  | 1(2) |  |  |  |  | 1(87) |  |  |
| Blood and blood-forming organs disease mortality |  |  |  |  |  |  |  |  |  |  |  |  |  |  |  | 1(30) |  |  |
| Digestive system mortality |  |  |  |  |  |  |  |  |  |  |  |  |  |  |  | 1(80) |  |  |
| IBD |  |  |  |  |  |  | 1(101) |  |  |  |  |  |  |  |  |  |  |  |
| Cirrhosis of liver |  |  |  |  |  |  |  |  |  | 1(2) |  |  |  |  |  | 1(80) |  |  |
| Nephritis & nephrosis |  |  |  |  |  |  |  |  |  | 1 (2) |  |  |  |  |  |  |  |  |
| Genitourinary organs disease |  |  |  |  |  |  |  |  |  |  |  |  |  |  |  | 1(25) |  |  |
| Motor neuron disease |  |  |  |  |  |  |  |  |  | 1(102) |  | 1(103) |  | 1(104) |  |  |  |  |
| Multiple sclerosis |  |  |  |  |  |  | 2(105,106) |  | 1(107) |  |  |  |  |  |  |  |  |  |
| Alzheimer disease |  |  |  |  |  |  |  |  |  |  | 1(108) |  |  |  | 1(104) |  |  |  |
| Alzheimer with other dementias disease |  |  |  |  |  |  |  |  |  |  |  |  | 1(104) |  |  |  |  |  |
| Parkinson disease |  |  |  |  |  |  |  |  |  |  |  |  | 1(104) |  |  |  |  |  |
| Parkinsonism |  |  |  |  |  |  |  |  |  |  |  |  | 1(104) |  |  |  |  |  |

No: results suggesting no statistically significant association; (-): results suggesting a negative statistically significant association; (+) : results suggesting a positive statistically significant association; COPD: Chronic obstructive pulmonary disease; CSD: Circulatory system disease; CeVD: Cerebrovascular disease; IHD: Ischaemic heart disease; AMI: Acute myocardial infarction; IBD: Inflammatory bowel disease.

References

1. Boice JD, Mumma MT, Blot WJ. Cancer Incidence and Mortality in Populations Living Near Uranium Milling and Mining Operations in Grants, New Mexico, 1950–2004. rare. sept 2010;174(5):624‑36.

2. Boice JD, Mumma MT, Blot WJ. Cancer and Noncancer Mortality in Populations Living Near Uranium and Vanadium Mining and Milling Operations in Montrose County, Colorado, 1950–2000. rare. juin 2007;167(6):711‑26.

3. Ye W, Sobue T, Lee VS, Tanooka H, Mifune M, Suyama A, et al. Mortality and Cancer Incidence in Misasa, Japan, a Spa Area with Elevated Radon Levels. Jpn J Cancer Res. août 1998;89(8):789‑96.

4. Boice JD, Cohen SS, Mumma MT, Chadda B, Blot WJ. Mortality among residents of Uravan, Colorado who lived near a uranium mill, 1936-84. J Radiol Prot. sept 2007;27(3):299‑319.

5. Navaranjan G, Berriault C, Do M, Villeneuve PJ, Demers PA. Cancer incidence and mortality from exposure to radon progeny among Ontario uranium miners. Occup Environ Med. déc 2016;73(12):838‑45.

6. Zablotska LB, Lane RSD, Frost SE, Thompson PA. Leukemia, lymphoma and multiple myeloma mortality (1950-1999) and incidence (1969-1999) in the Eldorado uranium workers cohort. Environ Res. avr 2014;130:43‑50.

7. Kelly-Reif K, Sandler DP, Shore D, Schubauer-Berigan M, Troester MA, Nylander-French L, et al. Mortality and cancer incidence among underground uranium miners in the Czech Republic 1977-1992. Occup Environ Med. août 2019;76(8):511‑8.

8. Richardson DB, Rage E, Demers PA, Do MT, DeBono N, Fenske N, et al. Mortality among uranium miners in North America and Europe: the Pooled Uranium Miners Analysis (PUMA). International Journal of Epidemiology. 1 avr 2021;50(2):633‑43.

9. Veiga LHS, Amaral ECS, Colin D, Koifman S. A retrospective mortality study of workers exposed to radon in a Brazilian underground coal mine. Radiat Environ Biophys. 1 juill 2006;45(2):125‑34.

10. Darby SC, Radford EP, Whitley E. Radon exposure and cancers other than lung cancer in Swedish iron miners. Environ Health Perspect. mars 1995;103 Suppl 2:45‑7.

11. Golden AP, Ellis ED, Cohen SS, Mumma MT, Leggett RW, Wallace PW, et al. Updated mortality analysis of the Mallinckrodt uranium processing workers, 1942–2012. International Journal of Radiation Biology. 3 avr 2022;98(4):701‑21.

12. Hodgson JT, Jones RD. Mortality of a cohort of tin miners 1941-86. Occupational and Environmental Medicine. 1 oct 1990;47(10):665‑76.

13. Jr JDB, Cohen SS, Mumma MT, Chadda B, Blot WJ. A cohort study of uranium millers and miners of Grants, New Mexico, 1979–2005. J Radiol Prot. août 2008;28(3):303.

14. Kreuzer M, Deffner V, Schnelzer M, Fenske N. Mortality in Underground Miners in a Former Uranium Ore Mine–Results of a Cohort Study Among Former Employees of Wismut AG in Saxony and Thuringia. Dtsch Arztebl Int. 29 janv 2021;118(4):41‑8.

15. Rage E, Caër-Lorho S, Laurier D. Low radon exposure and mortality among Jouac uranium miners An update of the French cohort (1946-2007). Journal of Radiological Protection. mars 2018;38(1):92‑108.

16. Demoury C, Marquant F, Ielsch G, Goujon S, Debayle C, Faure L, et al. Residential Exposure to Natural Background Radiation and Risk of Childhood Acute Leukemia in France, 1990–2009. Environmental Health Perspectives. avr 2017;125(4):714‑20.

17. Berlivet J, Hémon D, Cléro É, Ielsch G, Laurier D, Faure L, et al. Residential exposure to natural background radiation at birth and risk of childhood acute leukemia in France, 1990–2009. Journal of Environmental Radioactivity. 1 juill 2021;233:106613.

18. VIEL JF. Radon Exposure and Leukaemia in Adulthood. International Journal of Epidemiology. 1 août 1993;22(4):627‑31.

19. Silver SR, Bertke SJ, Hein MJ, Daniels RD, Fleming DA, Anderson JL, et al. Mortality and ionising radiation exposures among workers employed at the Fernald Feed Materials Production Center (1951–1985). Occup Environ Med. 1 juill 2013;70(7):453‑63.

20. Cocco PL, Carta P, Belli S, Picchiri GF, Flore MV. Mortality of Sardinian lead and zinc miners: 1960-88. Occupational and Environmental Medicine. 1 oct 1994;51(10):674‑82.

21. Berlivet J, Hémon D, Cléro É, Ielsch G, Laurier D, Guissou S, et al. Ecological association between residential natural background radiation exposure and the incidence rate of childhood central nervous system tumors in France, 2000-2012. J Environ Radioact. janv 2020;211:106071.

22. Zablotska LB, Lane RSD, Frost SE. Mortality (1950–1999) and cancer incidence (1969–1999) of workers in the Port Hope cohort study exposed to a unique combination of radium, uranium and γ-ray doses. BMJ Open. 1 janv 2013;3(2):e002159.

23. Schubauer-Berigan MK, Daniels RD, Pinkerton LE. Radon exposure and mortality among white and American Indian uranium miners: an update of the Colorado Plateau cohort. Am J Epidemiol. 15 mars 2009;169(6):718‑30.

24. Tomásek L, Darby SC, Swerdlow AJ, Placek V, Kunz E. Radon exposure and cancers other than lung cancer among uranium miners in West Bohemia. Lancet. 10 avr 1993;341(8850):919‑23.

25. Kreuzer M, Dufey F, Laurier D, Nowak D, Marsh JW, Schnelzer M, et al. Mortality from internal and external radiation exposure in a cohort of male German uranium millers, 1946–2008. International Archives of Occupational and Environmental Health. mai 2015;88(4):431‑41.

26. Chen SY, Hayes RB, Liang SR, Li QG, Stewart PA, Blair A. Mortality experience of haematite mine workers in China. Br J Ind Med. mars 1990;47(3):175‑81.

27. Suzuki Y, Honjo S, Kawamura H, Koishi F, Suzuki T, Hirohata T. Cancer Mortality in Low Radon Spa Area. Jpn J Cancer Res. nov 1994;85(11):1063‑6.

28. Mifune M, Sobue T, Arimoto H, Komoto Y, Kondo S, Tanooka H. Cancer Mortality Survey in a Spa Area (Misasa, Japan) with a High Radon Background. Jpn J Cancer Res. janv 1992;83(1):1‑5.

29. Kelly-Reif K, Bertke S, Daniels RD, Richardson DB, Schubauer-Berigan MK. Nonmalignant respiratory disease mortality in male Colorado Plateau uranium miners, 1960-2016. Am J Ind Med. oct 2022;65(10):773‑82.

30. Roscoe RJ. An update of mortality from all causes among white uranium miners from the Colorado plateau study group. American Journal of Industrial Medicine. 1997;31(2):211‑22.

31. Villeneuve PJ, Morrison HI, Volesky K, Lane RSD. Circulatory system disease mortality and occupational exposure to radon progeny in the cohort of Newfoundland Fluorspar Miners between 1950 and 2016. Int Arch Occup Environ Health. 1 avr 2023;96(3):411‑8.

32. Ahlman K, Koskela RS, Kuikka P, Koponen M, Annanmäki M. Mortality among sulfide ore miners. Am J Ind Med. 1991;19(5):603‑17.

33. Tomásek L, Swerdlow AJ, Darby SC, Placek V, Kunz E. Mortality in uranium miners in west Bohemia: a long-term cohort study. Occup Environ Med. mai 1994;51(5):308‑15.

34. Golden AP, Milder CM, Ellis ED, Anderson JL, Boice Jr. JD, Bertke SJ, et al. Cohort profile: four early uranium processing facilities in the US and Canada. International Journal of Radiation Biology. 3 juin 2021;97(6):833‑47.

35. Hauri D, Spycher B, Huss A, Zimmermann F, Grotzer M, von der Weid N, et al. Domestic Radon Exposure and Risk of Childhood Cancer: A Prospective Census-Based Cohort Study. Environ Health Perspect. 1 oct 2013;121(10):1239‑44.

36. Kendall GM, Little MP, Wakeford R, Bunch KJ, Miles JCH, Vincent TJ, et al. A record-based case-control study of natural background radiation and the incidence of childhood leukaemia and other cancers in Great Britain during 1980–2006. Leukemia. janv 2013;27(1):3‑9.

37. Thorne R, Foreman NK, Mott MG. Radon in Devon and Cornwall and paediatric malignancies. European Journal of Cancer. 1 févr 1996;32(2):282‑5.

38. Foreman NK, Thorne R, Berry PJ, Oakhill A, Mott MG. Childhood malignancies in the south-west region of England, 1976-1985. Med Pediatr Oncol. 1994;23(1):14‑9.

39. Nikkilä A, Arvela H, Mehtonen J, Raitanen J, Heinäniemi M, Lohi O, et al. Predicting residential radon concentrations in Finland: Model development, validation, and application to childhood leukemia. Scandinavian Journal of Work, Environment & Health. 2020;46(3):278‑92.

40. Del Risco Kollerud R, Blaasaas KG, Claussen B. Risk of leukaemia or cancer in the central nervous system among children living in an area with high indoor radon concentrations: results from a cohort study in Norway. Br J Cancer. 23 sept 2014;111(7):1413‑20.

41. Zlobina A, Farkhutdinov I, Carvalho FP, Wang N, Korotchenko T, Baranovskaya N, et al. Impact of Environmental Radiation on the Incidence of Cancer and Birth Defects in Regions with High Natural Radioactivity. International Journal of Environmental Research and Public Health. janv 2022;19(14):8643.

42. Raaschou-Nielsen O, Andersen CE, Andersen HP, Gravesen P, Lind M, Schüz J, et al. Domestic radon and childhood cancer in Denmark. Epidemiology. juill 2008;19(4):536‑43.

43. McLaughlin JR, King WD, Anderson TW, Clarke EA, Ashmore JP. Paternal radiation exposure and leukaemia in offspring: the Ontario case-control study. BMJ. 16 oct 1993;307(6910):959‑66.

44. Ha M, Hwang S sik, Kang S, Park NW, Chang BU, Kim Y. Geographical Correlations between Indoor Radon Concentration and Risks of Lung Cancer, Non-Hodgkin’s Lymphoma, and Leukemia during 1999–2008 in Korea. Int J Environ Res Public Health. avr 2017;14(4):344.

45. Henshaw DL, Eatough JP, Richardson RB. Radon as a causative factor in induction of myeloid leukaemia and other cancers. The Lancet. 28 avr 1990;335(8696):1008‑12.

46. Collman GW, Loomis DP, Sandler DP. Childhood cancer mortality and radon concentration in drinking water in North Carolina. Br J Cancer. avr 1991;63(4):626‑9.

47. Auvinen A, Kurttio P, Pekkanen J, Pukkala E, Ilus T, Salonen L. Uranium and other natural radionuclides in drinking water and risk of leukemia: a case–cohort study in Finland. Cancer Causes Control. 1 nov 2002;13(9):825‑9.

48. Smith BJ, Zhang L, Field RW. Iowa radon leukaemia study: a hierarchical population risk model for spatially correlated exposure measured with error. Statistics in Medicine. 2007;26(25):4619‑42.

49. López-Abente G, Núñez O, Fernández-Navarro P, Barros-Dios JM, Martín-Méndez I, Bel-Lan A, et al. Residential radon and cancer mortality in Galicia, Spain. Science of The Total Environment. 1 janv 2018;610‑611:1125‑32.

50. Jr JDB, Mumma M, Schweitzer S, Blot WJ. Cancer mortality in a Texas county with prior uranium mining and milling activities, 1950–2001. J Radiol Prot. sept 2003;23(3):247.

51. Möhner M, Lindtner M, Otten H, Gille HG. Leukemia and exposure to ionizing radiation among German uranium miners. American Journal of Industrial Medicine. 2006;49(4):238‑48.

52. Ře řicha V, Kulich M, Ře řicha R, Shore DL, Sandler DP. Incidence of Leukemia, Lymphoma, and Multiple Myeloma in Czech Uranium Miners: A Case–Cohort Study. Environmental Health Perspectives. juin 2006;114(6):818‑22.

53. Walsh L, Dufey F, Tschense A, Schnelzer M, Grosche B, Kreuzer M. RADON AND THE RISK OF CANCER MORTALITY—INTERNAL POISSON MODELS FOR THE GERMAN URANIUM MINERS COHORT. Health Physics. sept 2010;99(3):292.

54. Teras LR, Diver WR, Turner MC, Krewski D, Sahar L, Ward E, et al. Residential radon exposure and risk of incident hematologic malignancies in the Cancer Prevention Study-II Nutrition Cohort. Environ Res. juill 2016;148:46‑54.

55. Kelly-Reif K, Sandler DP, Shore D, Schubauer-Berigan MK, Troester MA, Nylander-French L, et al. Radon and cancer mortality among underground uranium miners in the Příbram region of the Czech Republic. Am J Ind Med. oct 2020;63(10):859‑67.

56. Lubin JH, Linet MS, Boice JD, Buckley J, Conrath SM, Hatch EE, et al. Case-control study of childhood acute lymphoblastic leukemia and residential radon exposure. J Natl Cancer Inst. 18 févr 1998;90(4):294‑300.

57. Lucie NP. Radon and Acute Lymphoblastic Leukaemia. Leukemia & Lymphoma. 1 janv 1990;3(3):213‑6.

58. The United Kingdom Childhood Cancer Study of exposure to domestic sources of ionising radiation: 1: radon gas. Br J Cancer. 5 juin 2002;86(11):1721‑6.

59. Kohli S, Noorlind B, Lofman O. Childhood leukaemia in areas with different radon levels: a spatial and temporal analysis using GIS. J Epidemiol Community Health. nov 2000;54(11):822‑6.

60. Eatough JP, Henshaw DL. Radon and monocytic leukaemia in England. J Epidemiol Community Health. déc 1993;47(6):506‑7.

61. Forastiere F, Sperati A, Cherubini G, Miceli M, Biggeri A, Axelson O. Adult myeloid leukaemia, geology, and domestic exposure to radon and gamma radiation: a case control study in central Italy. Occup Environ Med. févr 1998;55(2):106‑10.

62. Steinbuch M, Weinberg CR, Buckley JD, Robison LL, Sandler DP. Indoor residential radon exposure and risk of childhood acute myeloid leukaemia. Br J Cancer. nov 1999;81(5):900‑6.

63. Chen J, Xie L. DOMESTIC RADON EXPOSURE AND CHILDHOOD LEUKAEMIA AND LYMPHOMA: A POPULATION-BASED STUDY IN CANADA. Radiation Protection Dosimetry. 1 oct 2019;184(3‑4):486‑92.

64. Miller D, Morrison H, Semenciw R, Mao Y. Leukemia and residential exposure to radon. Can J Public Health. 1993;84(3):205‑6.

65. Kaletsch U, Kaatsch P, Meinert R, Schüz J, Czarwinski R, Michaelis J. Childhood cancer and residential radon exposure – results of a population-based case-control study in Lower Saxony (Germany). Radiat Environ Biophys. 1 sept 1999;38(3):211‑5.

66. Yoshinaga S, Tokonami S, Akiba S, Nitta H, Kabuto M. Case-control study of residential radon and childhood leukemia in Japan: results from preliminary analyses. International Congress Series. 1 févr 2005;1276:233‑5.

67. Law GR, Kane EV, Roman E, Smith A, Cartwright R. Residential radon exposure and adult acute leukaemia. Lancet. 27 mai 2000;355(9218):1888.

68. Peckham EC, Scheurer ME, Danysh HE, Lubega J, Langlois PH, Lupo PJ. Residential Radon Exposure and Incidence of Childhood Lymphoma in Texas, 1995-2011. Int J Environ Res Public Health. 25 sept 2015;12(10):12110‑26.

69. Forastiere F, Quiercia A, Cavariani F, Miceli M, Perucci CA, Axelson O. Cancer risk and radon exposure. Lancet. 2 mai 1992;339(8801):1115.

70. Ruano-Ravina A, Aragonés N, Kelsey KT, Pérez-Ríos M, Piñeiro-Lamas M, López-Abente G, et al. Residential radon exposure and brain cancer: an ecological study in a radon prone area (Galicia, Spain). Sci Rep. 15 juin 2017;7(1):3595.

71. Nilles JD, Lim D, Boyer MP, Wilson BD, Betar RA, Showalter HA, et al. The occurrence of bone and joint cancers and their association with rural living and radon exposure in Iowa. Environ Geochem Health. 5 avr 2022;

72. Etherington D j., Pheby D f. h., Bray F i. An ecological study of cancer incidence and radon levels in South West England. European Journal of Cancer. 1 juin 1996;32(7):1189‑97.

73. Boz S, Berlin C, Kwiatkowski M, Bochud M, Bulliard JL, Zwahlen M, et al. A prospective cohort analysis of residential radon and UV exposures and malignant melanoma mortality in the Swiss population. Environ Int. nov 2022;169:107437.

74. Kulich M, Reřicha V, Reřicha R, Shore DL, Sandler DP. Incidence of non-lung solid cancers in Czech uranium miners: a case-cohort study. Environ Res. avr 2011;111(3):400‑5.

75. Wheeler BW, Kothencz G, Pollard AS. Geography of non-melanoma skin cancer and ecological associations with environmental risk factors in England. Br J Cancer. juill 2013;109(1):235‑41.

76. Kreuzer M, Dufey F, Marsh JW, Nowak D, Schnelzer M, Walsh L. Mortality from cancers of the extra-thoracic airways in relation to radon progeny in the Wismut cohort, 1946-2008. Int J Radiat Biol. nov 2014;90(11):1030‑5.

77. Puskin JS. SMOKING AS A CONFOUNDER IN ECOLOGIC CORRELATIONS OF CANCER MORTALITY RATES WITH AVERAGE COUNTY RADON LEVELS. Health Physics. avr 2003;84(4):526.

78. Möhner M, Lindtner M, Otten H. IONIZING RADIATION AND RISK OF LARYNGEAL CANCER AMONG GERMAN URANIUM MINERS. Health Physics. déc 2008;95(6):725.

79. Miller AS, Harwick RD, Alfaro-Miranda M, Sundararajan M. Search for correlation of radon levels and incidence of salivary gland tumors. Oral Surgery, Oral Medicine, Oral Pathology. 1 janv 1993;75(1):58‑63.

80. Lane RSD, Frost SE, Howe GR, Zablotska LB. Mortality (1950–1999) and Cancer Incidence (1969–1999) in the Cohort of Eldorado Uranium Workers. rare. oct 2010;174(6a):773‑85.

81. Ruano-Ravina A, Aragonés N, Pérez-Ríos M, López-Abente G, Barros-Dios JM. Residential radon exposure and esophageal cancer. An ecological study from an area with high indoor radon concentration (Galicia, Spain). Int J Radiat Biol. avr 2014;90(4):299‑305.

82. Messier KP, Serre ML. Lung and stomach cancer associations with groundwater radon in North Carolina, USA. Int J Epidemiol. 1 avr 2017;46(2):676‑85.

83. Auvinen A, Salonen L, Pekkanen J, Pukkala E, Ilus T, Kurttio P. Radon and other natural radionuclides in drinking water and risk of stomach cancer: A case-cohort study in Finland. International Journal of Cancer. 2005;114(1):109‑13.

84. Kjellberg S, Wiseman JS. The relationship of radon to gastrointestinal malignancies. Am Surg. sept 1995;61(9):822‑5.

85. Reddy NK, Bhutani MS. Racial Disparities in Pancreatic Cancer and Radon Exposure: A Correlation Study. Pancreas. mai 2009;38(4):391.

86. Neuberger JS, Field RW. Radon and Breast Cancer. Risk Analysis. 1996;16(6):729‑30.

87. Zablotska LB, Fenske N, Schnelzer M, Zhivin S, Laurier D, Kreuzer M. Analysis of mortality in a pooled cohort of Canadian and German uranium processing workers with no mining experience. Int Arch Occup Environ Health. 1 janv 2018;91(1):91‑103.

88. Eatough JP, Henshaw DL. Radon and prostate cancer. Lancet. 26 mai 1990;335(8700):1292.

89. Kurttio P, Salonen L, Ilus T, Pekkanen J, Pukkala E, Auvinen A. Well water radioactivity and risk of cancers of the urinary organs. Environmental Research. 1 nov 2006;102(3):333‑8.

90. Goyal N, Camacho F, Mangano J, Goldenberg D. Evaluating for a geospatial relationship between radon levels and thyroid cancer in Pennsylvania. Laryngoscope. janv 2015;125(1):E45-49.

91. Schwartz GG, Klug MG. Thyroid Cancer Incidence Rates in North Dakota are Associated with Land and Water Use. International Journal of Environmental Research and Public Health. janv 2019;16(20):3805.

92. Langlois PH, Lee M, Lupo PJ, Rahbar MH, Cortez RK. RESIDENTIAL RADON AND BIRTH DEFECTS: A POPULATION-BASED ASSESSMENT. Birth Defects Res A Clin Mol Teratol. janv 2016;106(1):5‑15.

93. Sheehan A, Freni Sterrantino A, Fecht D, Elliott P, Hodgson S. Childhood type 1 diabetes: an environment-wide association study across England. Diabetologia. 2020;63(5):964‑76.

94. Mukharesh L, Greco KF, Banzon T, Koutrakis P, Li L, Hauptman M, et al. Environmental radon and childhood asthma. Pediatr Pulmonol. déc 2022;57(12):3165‑8.

95. Ruano-Ravina A, Cameselle-Lago C, Torres-Durán M, Pando-Sandoval A, Dacal-Quintas R, Valdés-Cuadrado L, et al. Indoor Radon Exposure and COPD, Synergic Association? A Multicentric, Hospital-Based Case-Control Study in a Radon-Prone Area. Arch Bronconeumol. oct 2021;57(10):630‑6.

96. Barbosa-Lorenzo R, Ruano-Ravina A, Ramis R, Aragonés N, Kelsey KT, Carballeira-Roca C, et al. Residential radon and COPD. An ecological study in Galicia, Spain. Int J Radiat Biol. févr 2017;93(2):222‑30.

97. Pando-Sandoval A, Ruano-Ravina A, Torres-Durán M, Dacal-Quintas R, Valdés-Cuadrado L, Hernández-Hernández JR, et al. Residential radon and characteristics of chronic obstructive pulmonary disease. Sci Rep. 26 janv 2022;12:1381.

98. Kreuzer M, Sogl M, Brüske I, Möhner M, Nowak D, Schnelzer M, et al. Silica dust, radon and death from non-malignant respiratory diseases in German uranium miners. Occup Environ Med. 1 déc 2013;70(12):869‑75.

99. Kreuzer M, Grosche B, Schnelzer M, Tschense A, Dufey F, Walsh L. Radon and risk of death from cancer and cardiovascular diseases in the German uranium miners cohort study: follow-up 1946–2003. Radiat Environ Biophys. 1 mai 2010;49(2):177‑85.

100. Kim SH, Park JM, Kim H. The prevalence of stroke according to indoor radon concentration in South Koreans: Nationwide cross section study. Medicine (Baltimore). janv 2020;99(4):e18859.

101. Mauriz-Barreiro V, Barreiro-de Acosta M, Bastón-Rey I, Ferreiro-Iglesias R, Calviño-Suárez C, Barros-Dios JM, et al. Radon exposure and inflammatory bowel disease in a radon prone area. Rev Esp Enferm Dig. juill 2022;114(7):405‑9.

102. Schwartz GG, Klug MG. Motor neuron disease mortality rates in U.S. states are associated with well water use. Amyotrophic Lateral Sclerosis and Frontotemporal Degeneration. 16 nov 2016;17(7‑8):528‑34.

103. Neilson S, Robinson I, Rose FC. Ecological correlates of motor neuron disease mortality: a hypothesis concerning an epidemiological association with radon gas and gamma exposure. J Neurol. 1 avr 1996;243(4):329‑36.

104. Zeng X, Berriault C, Arrandale VH, DeBono NL, Harris MA, Demers PA. Radon exposure and risk of neurodegenerative diseases among male miners in Ontario, Canada: A cohort study. Am J Ind Med. févr 2023;66(2):132‑41.

105. Abaszadeh Fathabadi Z, Ehrampoush MH, Mirzaei M, Mokhtari M, Nadi Sakhvidi M, Rahimdel A, et al. The relationship of indoor radon gas concentration with multiple sclerosis: a case-control study. Environ Sci Pollut Res. 1 mai 2020;27(14):16350‑61.

106. Groves-Kirkby CJ, Denman AR, Campbell J, Crockett RGM, Phillips PS, Rogers S. Is environmental radon gas associated with the incidence of neurodegenerative conditions? A retrospective study of multiple sclerosis in radon affected areas in England and Wales. J Environ Radioact. avr 2016;154:1‑14.

107. Bølviken B, Celius EG, Nilsen R, Strand T. Radon: A Possible Risk Factor in Multiple Sclerosis. Neuroepidemiology. 13 janv 2003;22(1):87‑94.

108. Lehrer S, Rheinstein PH, Rosenzweig KE. Association of Radon Background and Total Background Ionizing Radiation with Alzheimer’s Disease Deaths in U.S. States. Journal of Alzheimer’s Disease. 1 janv 2017;59(2):737‑41.
