# Supplementary material for: Radon exposure and potential health effects other than lung cancer: a systematic review and meta-analysis
Source: Front Public Health. 2024 Sep 25;12:1439355. doi: 10.3389/fpubh.2024.1439355 (PMC11461271; doi:10.3389/fpubh.2024.1439355)
Supplement: Supplementary file 9 [file Table_6.DOCX]

Supplementary Material

Table S8. Results of meta-analyses for Standardized Mortality or Incidence Ratio of malignant and non-malignant health outcomes, except lung cancer, among mine workers, using fixed effect model

| **Health outcome** |  | **Number of estimates included in the meta-analysis(reference)^α^** | **Total cases/Total sample size** | **Country** | **Meta SIR/SMR (95%CI)** | **p value for the meta SIR/SMR** | **Cochran's Q-test p for residual heterogeneity** | **I-square value (%) for residual heterogeneity (95%CI)** |
| --- | --- | --- | --- | --- | --- | --- | --- | --- |
| *Lymphohematological cancer* | | |  |  |  |  |  |  |
|  | Incidence among mine workers | 9 (1–3) | 599/193,734 | Canada, Czech Republic | 0.880 (0.813-0.954) | **0.002** | **0.009** | 60.717 (18.492-81.067) |
|  | Mortality | 22 (4–10) | 1141/514,368 | USA & Canada & France & Germany & Czech Republic, Brazil, USA, Italy, Sweden, UK | 0.970 (0.914-1.029) | 0.314 | **0.019** | 42.552 (4.720-65.362) |
| Leukaemia |  |  |  |  |  |  |  |  |
|  | Incidence | 3 (1–3) | 206/60,759 | Czech Republic, Canada | 0.922 (0.805-1.057) | 0.246 | **0.003** | 82.877 (47.764-94.387) |
|  | Mortality | 8 (4–6,8–10) | 468/134,223 | USA & Canada & France & Germany & Czech Republic, Brazil, USA, Sweden, UK | 0.977 (0.890-1.072) | 0.618 | 0.130 | 37.505 (0.000-72.397) |
| Chronic lymphocytic leukaemia | | |  |  |  |  |  |  |
|  | Mortality | 3 (1,10,11) | 27/33,805 | Canada, USA | 1.129 (0.781-1.632) | 0.518 | 0.150 | 47.37 (0.000-84.58) |
| Leukaemia excluding Chronic lymphocytic leukaemia | | |  |  |  |  |  |  |
|  | Mortality | 5 (1,10–13) | 178/74,409 | Canada, France, USA, Germany | 0.950 (0.821-1.099) | 0.492 | **0.059** | 55.891 (0.000-83.685) |
| Myeloid leukaemia | |  |  |  |  |  |  |  |
|  | Mortality | 2 (13,14) | 69/39,524 | Germany, Czech Republic | 0.963 (0.762-1.217) | 0.754 | 0.900 | 0.000 (NA-NA) |
| Lymphoma | |  |  |  |  |  |  |  |
|  | Incidence | 7 (1–3) | 308/137,952 | Czech Republic, Canada | 0.875 (0.785-0.976) | **0.016** | **0.092** | 44.953 (0.000-76.827) |
|  | Mortality | 12 (4,6,7,9,10) | 464/260,099 | USA & Canada & France & Germany & Czech Republic, USA, Italy, Sweden | 0.975 (0.888-1.069) | 0.587 | **0.092** | 37.453 (0.000-68.382) |
| Hodgkin lymphoma | |  |  |  |  |  |  |  |
|  | Incidence | 3 (1–3) | 48/60,759 | Czech Republic, Canada | 1.009 (0.776-1.313) | 0.945 | **0.013** | 76.991 (25.008-92.94) |
|  | Mortality | 5 (4,6,9,10) | 77/127,588 | USA & Canada & France & Germany & Czech Republic, USA, Sweden | 0.998 (0.798-1.247) | 0.983 | 0.130 | 43.800 (0.000-79.367) |
| Non-Hodgkin lymphoma | |  |  |  |  |  |  |  |
|  | Incidence | 4 (1–3) | 260/77,193 | Czech Republic, Canada | 0.85 (0.754-0.958) | **0.008** | 0.838 | 0.000 (0.000-84.688) |
|  | Mortality | 7 (4,6,7,9,10) | 387/132,511 | USA & Canada & France & Germany & Czech Republic, Italy, Sweden, USA | 0.97 (0.876-1.074) | 0.557 | 0.108 | 42.410 (0.000-75.789) |
| Multiple myeloma | |  |  |  |  |  |  |  |
|  | Incidence | 3 (1–3) | 85/60,759 | Czech Republic, Canada | 0.919 (0.75-1.127) | 0.419 | **0.001** | 85.576 (57.786-95.071) |
|  | Mortality | 5 (4,6,9,10) | 193/127,588 | USA & Canada & France & Germany & Czech Republic, USA, Sweden | 0.957 (0.829-1.103) | 0.542 | **0.036** | 61.168 (0.000-85.416) |
| *Brain and central nervous system cancer* | | |  |  |  |  |  |  |
|  | Incidence | 3 (1,3,15) | 92/48,393 | Czech Republic, Canada | 0.821 (0.677-0.997) | **0.046** | 0.450 | 0.000 (0.000-89.598) |
|  | Mortality | 4 (4,7,9,10) | 335/126,877 | USA & Canada & France & Germany & Czech Republic, Italy, Sweden, USA | 0.935 (0.844-1.037) | 0.202 | **< 0.001** | 83.333 (57.629-93.444) |
| Brain cancer | |  |  |  |  |  |  |  |
|  | Incidence | 2 (3,15) | 22/19,434 | Czech Republic, Canada | 0.958 (0.666-1.379) | 0.819 | 0.259 | 21.587 (NA-NA) |
|  | Mortality | 2 (3,15) | 18/19,079 | Czech Republic, Canada | 0.756 (0.511-1.117) | 0.160 | 0.956 | 0.000 (NA-NA) |
| *Malignant melanoma* | |  |  |  |  |  |  |  |
|  | Incidence | 3 (1,3,15) | 70/48,393 | Czech Republic, Canada | 0.52 (0.421-0.642) | **< 0.001** | **< 0.001** | 91.194 (77.208-96.598) |
|  | Mortality | 7 (1,3,10,11,13,14,16) | 108/93,018 | USA, Czech Republic, Canada, Germany | 1.022 (0.852-1.226) | 0.812 | **0.097** | 44.021 (0.000-76.45) |
| *Non-melanoma skin cancer* | | |  |  |  |  |  |  |
|  | Mortality | 6 (3,9,13,14,16) | 19/61,274 | USA, Czech Republic, Germany, Sweden | 1.31 (0.868-1.976) | 0.199 | **0.002** | 73.830 (40.234-88.541) |
| *Extra-thoracic airways cancer* | | |  |  |  |  |  |  |
|  | Incidence | 5 (1,3,15) | 320/80,352 | Canada, Czech Republic | 0.871 (0.786-0.965) | **0.009** | **0.001** | 77.732 (46.352-90.757) |
|  | Mortality | 11 (4,5,7,10,12–14) | 621/417,275 | Germany, France, USA & Canada & France & Germany & Czech Republic, Brazil, Italy, USA, Czech Republic | 0.906 (0.836-0.981) | **0.015** | **0.039** | 47.638 (0.000-73.899) |
| Nose cancer | |  |  |  |  |  |  |  |
|  | Mortality | 3 (12–14) | 23/44,924 | Germany, France, Czech Republic | 1.274 (0.855-1.899) | 0.233 | 0.903 | 0.000 (0.000-89.598) |
| Laryngeal cancer | |  |  |  |  |  |  |  |
|  | Incidence | 3 (1,3,15) | 139/48,393 | Czech Republic, Canada | 1.037 (0.882-1.219) | 0.659 | **0.025** | 73.022 (9.342-91.972) |
|  | Mortality | 4 (4,5,7,10) | 246/128,439 | USA & Canada & France & Germany & Czech Republic, Brazil, Italy, USA | 1.071 (0.940-1.221) | 0.300 | 0.454 | 0.000 (0.000-84.688) |
| Buccal and pharyngeal cancer | | |  |  |  |  |  |  |
|  | Incidence | 3 (1,3,15) | 187/48,393 | Czech Republic, Canada | 0.758 (0.661-0.869) | **< 0.001** | 0.134 | 50.182 (0.000-85.581) |
|  | Mortality | 4 (4,7,10) | 352/243,912 | USA & Canada & France & Germany & Czech Republic, Italy, USA | 0.795 (0.717-0.883) | **< 0.001** | 0.780 | 0.000 (0.000-84.688) |
| Tongue and mouth cancer | |  |  |  |  |  |  |  |
|  | Mortality | 2 (3,14) | 12/20,754 | Czech Republic | 1.241 (0.788-1.955) | 0.351 | 0.244 | 26.429 (NA-NA) |
| Lip cancer |  |  |  |  |  |  |  |  |
|  | Incidence | 2 (3,15) | 11/19,434 | Czech Republic, Canada | 0.737 (0.455-1.194) | 0.215 | 0.763 | 0.000 (NA-NA) |
| *Thyroid and other endocrine gland cancer* | | |  |  |  |  |  |  |
|  | Mortality | 5 (1,3,10,11,13) | 21/85,443 | Czech Republic, Canada, Germany, USA | 1.017 (0.688-1.503) | 0.933 | 0.913 | 0.000 (0.000-79.204) |
| Thyroid cancer | |  |  |  |  |  |  |  |
|  | Incidence | 2 (1,3) | 22/45,393 | Czech Republic, Canada | 0.751 (0.516-1.092) | 0.134 | 0.087 | 65.808 (0-92.238) |
|  | Mortality | 3 (1,3,13) | 19/80,184 | Czech Republic, Canada, Germany | 1.008 (0.667-1.524) | 0.968 | 0.802 | 0.000 (0.000-89.598) |
| *Digestive cancer* | |  |  |  |  |  |  |  |
|  | Incidence | 9 (1,3,15) | 1228/144,270 | Canada, Czech Republic | 0.923 (0.872-0.978) | **0.007** | **< 0.001** | 91.928 (86.911-95.022) |
|  | Mortality | 34 (4–10,17) | 4546/782,389 | USA & Canada & France & Germany & Czech Republic, Brazil, USA, China, Italy, Sweden, UK | 0.998 (0.969-1.029) | 0.912 | **< 0.001** | 60.212 (42.11-72.653) |
| Esophagus cancer | |  |  |  |  |  |  |  |
|  | Incidence | 3 (1,3,15) | 77/48,393 | Czech Republic, Canada | 0.93 (0.752-1.149) | 0.501 | **0.049** | 66.788 (0.000-90.416) |
|  | Mortality | 6 (4,5,7,9,10,17) | 391/136,177 | USA & Canada & France & Germany & Czech Republic, Brazil, Italy, USA, China, Sweden | 0.914 (0.822-1.016) | 0.095 | 0.543 | 0.000 (0.000-74.625) |
| Stomach cancer | |  |  |  |  |  |  |  |
|  | Incidence | 3 (1,3,15) | 248/48,393 | Czech Republic, Canada | 1.03 (0.915-1.16) | 0.623 | **< 0.001** | 89.684 (72.225-96.169) |
|  | Mortality | 10 (4–10,17) | 1222/145,407 | USA & Canada & France & Germany & Czech Republic, USA, Brazil, Italy, China, Sweden, UK | 1.092 (1.030-1.157) | **0.003** | **0.052** | 46.347 (0.000-74.174) |
| Liver and gallbladder cancer | | |  |  |  |  |  |  |
|  | Mortality | 6 (4,7,9,10,17) | 599/134,615 | Italy, China, USA & Canada & France & Germany & Czech Republic, Sweden, USA | 1.124 (1.038-1.218) | **0.004** | **0.028** | 60.139 (2.207-83.752) |
| Liver cancer | |  |  |  |  |  |  |  |
|  | Incidence | 2 (1,3) | 75/45,393 | Czech Republic, Canada | 1.166 (0.943-1.442) | 0.156 | **< 0.001** | 93.884 (80.475-98.084) |
|  | Mortality | 8 (1,3,7,9,12–14,17) | 356/102,382 | Czech Republic, Canada, France, Italy, China, Germany, Sweden | 1.273 (1.150-1.409) | **< 0.001** | **0.003** | 68.123 (33.081-84.815) |
| Gallbladder cancer | |  |  |  |  |  |  |  |
|  | Mortality | 5 (3,9,13,14,18) | 96/61,306 | Czech Republic, Germany, Sweden | 1.048 (0.867-1.268) | 0.627 | **0.058** | 56.237 (0.000-83.800) |
| Pancreatic cancer | |  |  |  |  |  |  |  |
|  | Incidence | 3 (1,3,15) | 169/48,393 | Canada, Czech Republic | 0.997 (0.862-1.152) | 0.965 | 0.017 | 75.618 (19.601-92.606) |
|  | Mortality | 4 (4,7,9,10) | 687/126,877 | USA & Canada & France & Germany & Czech Republic, Italy, USA, Sweden | 0.956 (0.886-1.033) | 0.258 | 0.287 | 20.539 (0.000-87.833) |
| Intestine and rectal cancer | | |  |  |  |  |  |  |
|  | Mortality | 12 (4–7,9,10) | 1632/258,273 | USA & Canada & France & Germany & Czech Republic, USA, Brazil, Italy, Sweden | 0.926 (0.881-0.974) | **0.003** | 0.066 | 41.297 (0.000-70.209) |
| Intestine cancer | |  |  |  |  |  |  |  |
|  | Mortality | 7 (4,6,9,10) | 1044/128,540 | USA & Canada & France & Germany & Czech Republic, USA, Sweden | 0.908 (0.854-0.965) | **0.002** | 0.345 | 11.047 (0.000-74.033) |
| Colorectal cancer | |  |  |  |  |  |  |  |
|  | Incidence | 5 (1,3,15) | 739/67,827 | Canada, Czech Republic | 0.866 (0.806-0.932) | **< 0.001** | **< 0.001** | 93.059 (86.752-96.363) |
| Colon cancer | |  |  |  |  |  |  |  |
|  | Incidence | 2 (3,15) | 113/19,434 | Canada, Czech Republic | 0.996 (0.841-1.179) | 0.960 | 0.217 | 34.464 (NA-NA) |
|  | Mortality | 5 (3,10,11,15,18) | 154/28,392 | Czech Republic, Canada, USA, Germany | 0.870 (0.750-1.009) | 0.065 | 0.224 | 29.638 (0.000-72.755) |
| Rectal cancer | |  |  |  |  |  |  |  |
|  | Incidence | 2 (15,18) | 141/19,434 | Czech Republic, Canada | 1.331 (1.143-1.549) | **< 0.001** | **0.059** | 71.877 (0.000-93.673) |
|  | Mortality | 3 (4,9,10) | 575/122,137 | USA & Canada & France & Germany & Czech Republic, USA, Sweden | 0.977 (0.895-1.067) | 0.605 | **0.038** | 69.474 (0.000-91.095) |
| *Kidney, ureter, other urinary organs cancer* | |  |  |  |  |  |  |  |
|  | Incidence | 3 (1,3,15) | 154/48,393 | Canada, Czech Republic | 0.701 (0.606-0.812) | **< 0.001** | **0.036** | 69.922 (0.000-91.206) |
|  | Mortality | 7 (4,6,7,9,10) | 436/133,097 | USA & Canada & France & Germany & Czech Republic, Italy, Sweden, USA | 0.968 (0.882-1.064) | 0.503 | 0.364 | 8.404 (0.000-73.262) |
| Kidney cancer | |  |  |  |  |  |  |  |
|  | Incidence | 2 (1,15) | 105/31,959 | Canada | 0.615 (0.512-0.739) | **< 0.001** | 0.256 | 22.644 (NA-NA) |
|  | Mortality | 6 (4,6,9,10) | 429/128,357 | USA & Canada & France & Germany & Czech Republic, Sweden, USA | 0.964 (0.877-1.059) | 0.446 | 0.309 | 16.259 (0.000-78.75) |
| *Bladder and other urinary organ cancer* | | |  |  |  |  |  |  |
|  | Incidence | 3 (1,3,15) | 280/48,393 | Czech Republic, Canada | 0.779 (0.696-0.873) | **< 0.001** | **0.003** | 83.309 (49.393-94.495) |
|  | Mortality | 7 (4,6,7,9,10) | 479/133,097 | Italy, USA, USA & Canada & France & Germany & Czech Republic, Sweden | 0.882 (0.803-0.968) | **0.008** | **0.034** | 56.098 (0.000-81.124) |
| Bladder cancer | |  |  |  |  |  |  |  |
|  | Incidence | 2 (3,15) | 80/19,434 | Czech Republic, Canada | 1.014 (0.827-1.244) | 0.891 | 0.506 | 0.000 (NA-NA) |
|  | Mortality | 7 (3,7,12–15,18) | 288/72,797 | Czech Republic, France, Canada, Italy, Germany | 1.063 (0.953-1.186) | 0.273 | 0.982 | 0.000 (0.000-70.809) |
| *Testis and other male genital organ cancer, excluding prostate cancer* | | | |  |  |  |  |  |
|  | Mortality | 5 (1,3,10,13,14) | NA/87,018 | Czech Republic, Canada, Germany, USA | 0.831 (0.593-1.164) | 0.281 | 0.848 | 0.000 (0.000-79.204) |
| Testis cancer | |  |  |  |  |  |  |  |
|  | Incidence | 2 (1,3) | 28/45,393 | Czech Republic, Canada | 0.655 (0.470-0.913) | **0.012** | 0.148 | 52.320 (0.000-88.065) |
|  | Mortality | 4 (1,3,13,14) | NA/84,504 | Czech Republic, Canada, Germany | 0.834 (0.591-1.176) | 0.300 | 0.713 | 0.000 (0.000-84.688) |
| *Prostate cancer* | |  |  |  |  |  |  |  |
|  | Incidence | 3 (1,3,15) | 761/48,393 | Czech Republic, Canada | 0.605 (0.568-0.644) | **< 0.001** | **< 0.001** | 93.366 (83.984-97.252) |
|  | Mortality | 5 (4,5,7,9,10) | 952/129,733 | USA & Canada & France & Germany & Czech Republic, Brazil, Italy, USA, Sweden | 0.861 (0.807-0.919) | **< 0.001** | 0.103 | 48.042 (0.000-80.962) |
| *Bone cancer* | |  |  |  |  |  |  |  |
|  | Mortality | 5 (1,10,13,14,16) | NA/73,839 | USA, Canada, Germany, Czech Republic | 0.943 (0.625-1.424) | 0.781 | **0.04** | 60.216 (0.000-85.107) |
| *Connective and other soft tissue cancer* | | |  |  |  |  |  |  |
|  | Incidence | 2 (1,3) | 22/45,393 | Czech Republic, Canada | 0.626 (0.429-0.915) | **0.016** | 0.593 | 0.000 (NA-NA) |
|  | Mortality | 5 (1,3,10,13,14) | 30/87,018 | Czech Republic, Canada, USA, Germany | 0.855 (0.616-1.187) | 0.350 | 0.422 | 0.000 (0.000-79.204) |
| *Breast cancer* | |  |  |  |  |  |  |  |
|  | Mortality | 3 (1,11,16) | NA/34,546 | USA, Canada | 0.967 (0.434-2.157) | 0.935 | 0.403 | 0.000 (0.000-89.598) |
| *Chronic obstructive pulmonary disease & asthma* | |  |  |  |  |  |  |  |
|  | Mortality | 6 (4,6,10,19) | 1912/131,084 | USA & Canada & France & Germany & Czech Republic, USA | 0.977 (0.940-1.015) | 0.230 | **0.002** | 74.003 (40.703-88.603) |
| Chronic obstructive pulmonary disease | | |  |  |  |  |  |  |
|  | Mortality | 4 (4,6) | 1844/124,549 | USA & Canada & France & Germany & Czech Republic, USA | 0.977 (0.939-1.016) | 0.237 | **0.006** | 76.113 (34.349-91.309) |
| Bronchitis, emphysema, and asthma | | |  |  |  |  |  |  |
|  | Mortality | 4 (10,11,19,20) | 159/12,518 | USA | 1.578 (1.356-1.836) | **< 0.001** | **< 0.001** | 91.787 (82.151-96.221) |
| *All circulatory system disease* | | |  |  |  |  |  |  |
|  | Mortality | 6 (4,5,7,10,21,22) | 18,643/133,600 | USA & Canada & France & Germany & Czech Republic, Brazil, Italy, Finland, USA, Canada | 0.879 (0.870-0.889) | **< 0.001** | **< 0.001** | 90.225 (82.417-94.565) |
| Ischemic heart disease | | |  |  |  |  |  |  |
|  | Mortality | 5 (4,5,8,10,21) | 10,289/127,306 | USA & Canada & France & Germany & Czech Republic, Brazil, Finland, UK, USA | 0.928 (0.909-0.947) | **< 0.001** | **< 0.001** | 88.711 (76.322-94.617) |
| Cerebrovascular disease/Stroke | | |  |  |  |  |  |  |
|  | Mortality | 11 (1,3,5,10–13,15,16,18) | 2383/104,420 | USA, Czech Republic, Canada, France, Brazil, Germany | 1.03 (0.989-1.073) | 0.155 | **< 0.001** | 96.657 (95.344-97.6) |
| Hypertension | |  |  |  |  |  |  |  |
|  | Mortality | 4 (5,15,16) | 35/9523 | USA, Brazil, Canada | 1.146 (0.828-1.586) | 0.410 | **< 0.001** | 84.346 (60.757-93.755) |
| *Diabetes mellitus* | |  |  |  |  |  |  |  |
|  | Mortality | 8 (3,5,7,10,11,15,16) | 175/35,956 | USA, Czech Republic, Canada, Brazil, Italy | 0.867 (0.752-1.000) | 0.050 | **0.015** | 59.571 (11.908-81.445) |
| *Digestive disorders* | |  |  |  |  |  |  |  |
|  | Mortality | 4 (4,5,7,10) | 2613/128,439 | USA, USA & Canada & France & Germany & Czech Republic, Brazil, Italy | 0.923 (0.894-0.952) | **< 0.001** | **0.016** | 70.904 (16.875-89.816) |
| Cirrhosis and other liver disease | | |  |  |  |  |  |  |
|  | Mortality | 4 (4,5,7,10) | 1502/128,439 | USA & Canada & France & Germany & Czech Republic, Brazil, USA, Italy | 0.963 (0.910-1.019) | 0.187 | **0.001** | 81.839 (52.943-92.991) |
| *Mental and behavioral disorders* | | |  |  |  |  |  |  |
|  | Mortality | 6 (3,10,11,13,16,23) | 284/64,472 | Czech Republic, USA, Germany | 1.075 (0.956-1.209) | 0.225 | **< 0.001** | 92.735 (86.913-95.967) |
| *Nervous system and sense organ disorders* | | |  |  |  |  |  |  |
|  | Mortality | 7 (3,10,11,13,15,16) | 330/63,564 | Czech Republic, Canada, USA, Germany | 0.885 (0.797-0.983) | **0.023** | **< 0.001** | 75.163 (47.359-88.281) |

α One reference can contribute to the meta-analysis with more than one estimate, depending on if estimates were available for different subtypes of the health outcome of interest, or for subgroups of the study population by sex, race, pay-roll status etc …; NA: Not available

References

1. Navaranjan G, Berriault C, Do M, Villeneuve PJ, Demers PA. Cancer incidence and mortality from exposure to radon progeny among Ontario uranium miners. Occup Environ Med. déc 2016;73(12):838‑45.

2. Zablotska LB, Lane RSD, Frost SE, Thompson PA. Leukemia, lymphoma and multiple myeloma mortality (1950-1999) and incidence (1969-1999) in the Eldorado uranium workers cohort. Environ Res. avr 2014;130:43‑50.

3. Kelly-Reif K, Sandler DP, Shore D, Schubauer-Berigan M, Troester MA, Nylander-French L, et al. Mortality and cancer incidence among underground uranium miners in the Czech Republic 1977-1992. Occup Environ Med. août 2019;76(8):511‑8.

4. Richardson DB, Rage E, Demers PA, Do MT, DeBono N, Fenske N, et al. Mortality among uranium miners in North America and Europe: the Pooled Uranium Miners Analysis (PUMA). International Journal of Epidemiology. 1 avr 2021;50(2):633‑43.

5. Veiga LHS, Amaral ECS, Colin D, Koifman S. A retrospective mortality study of workers exposed to radon in a Brazilian underground coal mine. Radiat Environ Biophys. 1 juill 2006;45(2):125‑34.

6. Silver SR, Bertke SJ, Hein MJ, Daniels RD, Fleming DA, Anderson JL, et al. Mortality and ionising radiation exposures among workers employed at the Fernald Feed Materials Production Center (1951–1985). Occup Environ Med. 1 juill 2013;70(7):453‑63.

7. Cocco PL, Carta P, Belli S, Picchiri GF, Flore MV. Mortality of Sardinian lead and zinc miners: 1960-88. Occupational and Environmental Medicine. 1 oct 1994;51(10):674‑82.

8. Hodgson JT, Jones RD. Mortality of a cohort of tin miners 1941-86. Occupational and Environmental Medicine. 1 oct 1990;47(10):665‑76.

9. Darby SC, Radford EP, Whitley E. Radon exposure and cancers other than lung cancer in Swedish iron miners. Environ Health Perspect. mars 1995;103 Suppl 2:45‑7.

10. Golden AP, Ellis ED, Cohen SS, Mumma MT, Leggett RW, Wallace PW, et al. Updated mortality analysis of the Mallinckrodt uranium processing workers, 1942–2012. International Journal of Radiation Biology. 3 avr 2022;98(4):701‑21.

11. Jr JDB, Cohen SS, Mumma MT, Chadda B, Blot WJ. A cohort study of uranium millers and miners of Grants, New Mexico, 1979–2005. J Radiol Prot. août 2008;28(3):303.

12. Rage E, Caër-Lorho S, Laurier D. Low radon exposure and mortality among Jouac uranium miners An update of the French cohort (1946-2007). Journal of Radiological Protection. mars 2018;38(1):92‑108.

13. Kreuzer M, Deffner V, Schnelzer M, Fenske N. Mortality in Underground Miners in a Former Uranium Ore Mine–Results of a Cohort Study Among Former Employees of Wismut AG in Saxony and Thuringia. Dtsch Arztebl Int. 29 janv 2021;118(4):41‑8.

14. Tomásek L, Darby SC, Swerdlow AJ, Placek V, Kunz E. Radon exposure and cancers other than lung cancer among uranium miners in West Bohemia. Lancet. 10 avr 1993;341(8850):919‑23.

15. Zablotska LB, Lane RSD, Frost SE. Mortality (1950–1999) and cancer incidence (1969–1999) of workers in the Port Hope cohort study exposed to a unique combination of radium, uranium and γ-ray doses. BMJ Open. 1 janv 2013;3(2):e002159.

16. Schubauer-Berigan MK, Daniels RD, Pinkerton LE. Radon exposure and mortality among white and American Indian uranium miners: an update of the Colorado Plateau cohort. Am J Epidemiol. 15 mars 2009;169(6):718‑30.

17. Chen SY, Hayes RB, Liang SR, Li QG, Stewart PA, Blair A. Mortality experience of haematite mine workers in China. Br J Ind Med. mars 1990;47(3):175‑81.

18. Kreuzer M, Dufey F, Laurier D, Nowak D, Marsh JW, Schnelzer M, et al. Mortality from internal and external radiation exposure in a cohort of male German uranium millers, 1946–2008. International Archives of Occupational and Environmental Health. mai 2015;88(4):431‑41.

19. Kelly-Reif K, Bertke S, Daniels RD, Richardson DB, Schubauer-Berigan MK. Nonmalignant respiratory disease mortality in male Colorado Plateau uranium miners, 1960-2016. Am J Ind Med. oct 2022;65(10):773‑82.

20. Roscoe RJ. An update of mortality from all causes among white uranium miners from the Colorado plateau study group. American Journal of Industrial Medicine. 1997;31(2):211‑22.

21. Ahlman K, Koskela RS, Kuikka P, Koponen M, Annanmäki M. Mortality among sulfide ore miners. Am J Ind Med. 1991;19(5):603‑17.

22. Villeneuve PJ, Morrison HI, Volesky K, Lane RSD. Circulatory system disease mortality and occupational exposure to radon progeny in the cohort of Newfoundland Fluorspar Miners between 1950 and 2016. Int Arch Occup Environ Health. 1 avr 2023;96(3):411‑8.

23. Tomásek L, Swerdlow AJ, Darby SC, Placek V, Kunz E. Mortality in uranium miners in west Bohemia: a long-term cohort study. Occup Environ Med. mai 1994;51(5):308‑15.
