# Supplementary material for: Radon exposure and potential health effects other than lung cancer: a systematic review and meta-analysis
Source: Front Public Health. 2024 Sep 25;12:1439355. doi: 10.3389/fpubh.2024.1439355 (PMC11461271; doi:10.3389/fpubh.2024.1439355)
Supplement: Supplementary file 10 [file Table_7.DOCX]

Supplementary Material

Table S9. Results of meta-analyses on exposure-risk relationships between radon exposure and malignant and non-malignant health outcomes, except lung cancer, among children, adults in the general population, and mine workers, using fixed effect model

| **Health outcome** |  | **Number of estimates included in the meta-analysis (reference)^α^** | **Total cases/Total sample size** | **Country** | **Meta Risk Ratio per 100 Bq/m^3β^ or 100 WLM^λ^ (95%CI)** | **p value for the metaRR** | **Cochran's Q-test p for residual heterogeneity** | **I-square value (%) for residual heterogeneity (95%CI)** |
| --- | --- | --- | --- | --- | --- | --- | --- | --- |
| *Lymphohematological cancer* | | |  |  |  |  |  |  |
|  | Incidence in children | 7 (1–6) | 17,106/2,069,256 | Switzerland, Finland, Norway, France, Denmark, UK | 1.014 (0.998-1.031) | 0.083 | 0.890 | 0.000 (0.000-70.809) |
|  | Incidence among mine workers | 3 (7–9) | 653/45,469 | Canada, Germany | 0.995 (0.973-1.017) | 0.634 | 0.339 | 7.616 (0.000-90.390) |
|  | Mortality among mine workers | 6 (7,8,10,11) | 545/23,8177 | Czech Republic, Germany, Canada | 1.011 (0.982-1.040) | 0.473 | 0.944 | 0.000 (0.000-74.625) |
| Leukaemia |  |  |  |  |  |  |  |  |
|  | Incidence among children | 6 (1–6) | 14,787/2,063,663 | Switzerland, Finland, Norway, France, Denmark, UK | 1.014 (0.996-1.033) | 0.116 | 0.806 | 0.000 (0.000-74.625) |
|  | Incidence among mine workers | 4 (7–9) | 545/60,835 | Canada, Germany | 0.993 (0.972-1.014) | 0.502 | 0.430 | 0.000 (0.000-84.688) |
|  | Mortality among mine workers | 5 (7,8,10,11) | 301/136,637 | Germany, Canada, Czech Republic | 1.006 (0.968-1.046) | 0.753 | 0.892 | 0.000 (0.000-79.204) |
| Chronic lymphocytic leukaemia | | |  |  |  |  |  |  |
|  | Incidence among mine workers | 3 (7–9) | 227/44,477 | Canada, Germany | 0.991 (0.96-1.022) | 0.563 | 0.731 | 0.000 (0.000-89.598) |
|  | Mortality among mine workers | 2 (8,11) | 29/44,980 | Czech Republic, Canada | 0.989 (0.633-1.543) | 0.960 | 0.008 | 85.751 (42.766-96.453) |
| Leukaemia excluding Chronic lymphocytic leukaemia | | |  |  |  |  |  |  |
|  | Incidence among mine workers | 3 (7–9) | 245/44,477 | Canada, Germany | 0.994 (0.953-1.038) | 0.801 | 0.140 | 49.057 (0.000-85.189) |
|  | Mortality among mine workers | 2 (8,11) | 59/44,980 | Czech Republic, Canada | 1.365 (0.871-2.141) | 0.175 | 0.076 | 68.198 (0.000-92.827) |
| Lymphoma | |  |  |  |  |  |  |  |
|  | Mortality among mine workers | 5 (7,10,11) | 161/124,327 | Czech Republic, Germany, Canada | 1.024 (0.963-1.088) | 0.449 | 0.867 | 0.000 (0.000-79.204) |
| Hodgkin lymphoma | |  |  |  |  |  |  |  |
|  | Mortality among mine workers | 2 (7,11) | 15/32,670 | Canada, Czech Republic | 0.733 (0.241-2.226) | 0.584 | 0.468 | 0.000 (NA-NA) |
| Non-Hodgkin lymphoma | |  |  |  |  |  |  |  |
|  | Mortality among mine workers | 3 (7,10,11) | 146/91,657 | Czech Republic, Germany, Canada | 1.025 (0.964-1.089) | 0.431 | 0.822 | 0.000 (0.000-89.598) |
| Multiple myeloma | |  |  |  |  |  |  |  |
|  | Mortality among mine workers | 3 (7,10,11) | 81/91,657 | Germany, Canada, Czech Republic | 1.007 (0.947-1.070) | 0.823 | 0.992 | 0.000 (0.000-89.598) |
| *Brain and central nervous system tumours* | | |  |  |  |  |  |  |
|  | Incidence among children | 4 (1,3,5,6) | 8262/2,024,707 | Switzerland, Norway, Denmark, UK | 1.007 (0.988-1.026) | 0.467 | 0.108 | 50.626 (0.000-83.674) |
| *Brain and central nervous system cancer* | | |  |  |  |  |  |  |
|  | Mortality among mine workers | 2 (10,12) | 120/61,632 | Germany, Canada | 0.982 (0.947-1.018) | 0.319 | 0.809 | 0.000 (NA-NA) |
| *Malignant melanoma* | |  |  |  |  |  |  |  |
|  | Mortality among adults in the general population | 2 (13,14) | 5226/5,716,404 | Switzerland, USA | 1.095 (0.993-1.209) | 0.069 | 0.879 | 0.000 (NA-NA) |
| *Non-melanoma skin cancer* | | |  |  |  |  |  |  |
|  | Mortality among adults in the general population | 2 (13,14) | 1431/5,716,404 | Switzerland, USA | 0.939 (0.705-1.251) | 0.668 | 0.197 | 39.900 (NA-NA) |
| *Extra-thoracic airways cancer* | | |  |  |  |  |  |  |
|  | Incidence among mine workers | 3 (8,15,16) | 401/45,738 | Czech Republic, Canada | 0.916 (0.787-1.066) | 0.257 | 0.252 | 27.397 (0.000-92.448) |
|  | Mortality among mine workers | 3 (8,11,17) | 1747/103,670 | Canada, Germany, Czech Republic | 1.035 (0.993-1.079) | 0.106 | 0.552 | 0.000 (0.000-89.598) |
| *Digestive cancer* | |  |  |  |  |  |  |  |
|  | Incidence among mine workers | 5 (8,16) | 468/90,010 | Canada | 0.977 (0.934-1.022) | 0.312 | 0.74 | 0.000 (0.000-79.204) |
|  | Mortality among mine workers | 11 (8,10–12,16) | 1933/450,453 | Czech Republic, Germany, Canada | 1.011 (1.000-1.023) | 0.060 | 0.144 | 31.889 (0.000-66.518) |
| Stomach cancer | |  |  |  |  |  |  |  |
|  | Incidence among mine workers | 2 (8,16) | 196/43,912 | Canada | 0.958 (0.895-1.025) | 0.213 | 0.694 | 0.000 (NA-NA) |
|  | Mortality among mine workers | 4 (8,10,11,16) | 880/120,203 | Czech Republic, Germany, Canada | 1.015 (0.997-1.034) | 0.110 | 0.219 | 32.242 (0.000-75.843) |
| Liver cancer | |  |  |  |  |  |  |  |
|  | Mortality among mine workers | 2 (10,11) | 207/75,421 | Germany, Czech Republic | 1.045 (0.998-1.095) | 0.063 | 0.784 | 0.000 (NA-NA) |
| Pancreatic cancer | |  |  |  |  |  |  |  |
|  | Mortality among mine workers | 2 (10,16) | 296/75,223 | Germany, Canada | 1 (0.977-1.024) | 0.982 | 0.823 | 0.000 (NA-NA) |
| Intestine and rectal cancer | | |  |  |  |  |  |  |
|  | Mortality among mine workers | 3 (10,12,16) | 639/136,855 | Germany, Canada | 1.021 (0.999-1.043) | 0.063 | 0.828 | 0.000 (0.000-89.598) |
| Rectal cancer | |  |  |  |  |  |  |  |
|  | Mortality among mine workers | 2 (10,12) | 256/61,632 | Germany, Canada | 1.028 (0.993-1.064) | 0.113 | 0.751 | 0.000 (NA-NA) |
| *Kidney, ureter, other urinary organs cancer* | |  |  |  |  |  |  |  |
|  | Mortality among mine workers | 5 (8,11,12,18) | 285/109,988 | Canada, France, Germany, Czech Republic | 1.022 (0.993-1.052) | 0.137 | 0.509 | 0.000 (0.000-79.204) |
| Kidney cancer | |  |  |  |  |  |  |  |
|  | Mortality among mine workers | 3 (8,10,12) | 230/90,178 | Canada, Germany | 1.016 (0.976-1.056) | 0.443 | 0.200 | 37.781 (0.000-80.446) |
| *Bladder and other urinary organ cancer* | | |  |  |  |  |  |  |
|  | Mortality among mine workers | 2 (10,12) | 187/61,632 | Germany, Canada | 1.020 (0.985-1.056) | 0.264 | 0.923 | 0.000 (NA-NA) |
| *Prostate cancer* | |  |  |  |  |  |  |  |
|  | Mortality among mine workers | 2 (10,16) | 362/75,223 | Germany, Canada | 0.998 (0.975-1.021) | 0.866 | 0.521 | 0.000 (NA-NA) |
| *Chronic obstructive pulmonary disease* | | |  |  |  |  |  |  |
|  | Mortality among mine workers | 3 (19–21) | 1073/69,120 | USA, Germany | 1.004 (0.991-1.016) | 0.563 | 0.514 | 0.000 (0.000-89.598) |
| *All circulatory system disease* | | |  |  |  |  |  |  |
|  | Mortality among mine workers | 6 (16,22–24) | 10,117/115,145 | Germany, France, Canada | 1.000 (0.997-1.003) | 0.850 | 0.065 | 51.861 (0.000-80.813) |
| Ischemic heart disease | |  |  |  |  |  |  |  |
|  | Mortality among mine workers | 3 (16,22–24) | 6830/82,673 | Germany, France, Canada | 0.997 (0.986-1.008) | 0.551 | 0.321 | 11.997 (0.000-90.846) |
| Cerebrovascular disease / Stroke | | |  |  |  |  |  |  |
|  | Mortality among mine workers | 4 (16,22–24) | 2151/82,673 | Germany, France, Canada | 1.001 (0.998-1.004) | 0.653 | 0.003 | 78.495 (42.187-92.000) |

^α^ One reference can contribute to the meta-analysis with more than one estimate depending on if estimates were available for different subtypes of the health outcome of interest, or for subgroups of the study population by sex, race, pay-roll status etc …; ^β^ 100 Bq/m3 is the unite of exposure increment for residential exposure (among children and adults in the general population); ^λ^ 100 WLM is the unite of exposure increment for occupational exposure (among mine workers); NA: Not available.

**References**

1. Hauri D, Spycher B, Huss A, Zimmermann F, Grotzer M, von der Weid N, et al. Domestic Radon Exposure and Risk of Childhood Cancer: A Prospective Census-Based Cohort Study. Environ Health Perspect. 1 oct 2013;121(10):1239‑44.

2. Nikkilä A, Arvela H, Mehtonen J, Raitanen J, Heinäniemi M, Lohi O, et al. Predicting residential radon concentrations in Finland: Model development, validation, and application to childhood leukemia. Scandinavian Journal of Work, Environment & Health. 2020;46(3):278‑92.

3. Del Risco Kollerud R, Blaasaas KG, Claussen B. Risk of leukaemia or cancer in the central nervous system among children living in an area with high indoor radon concentrations: results from a cohort study in Norway. Br J Cancer. 23 sept 2014;111(7):1413‑20.

4. Demoury C, Marquant F, Ielsch G, Goujon S, Debayle C, Faure L, et al. Residential Exposure to Natural Background Radiation and Risk of Childhood Acute Leukemia in France, 1990–2009. Environmental Health Perspectives. avr 2017;125(4):714‑20.

5. Raaschou-Nielsen O, Andersen CE, Andersen HP, Gravesen P, Lind M, Schüz J, et al. Domestic radon and childhood cancer in Denmark. Epidemiology. juill 2008;19(4):536‑43.

6. Kendall GM, Little MP, Wakeford R, Bunch KJ, Miles JCH, Vincent TJ, et al. A record-based case-control study of natural background radiation and the incidence of childhood leukaemia and other cancers in Great Britain during 1980–2006. Leukemia. janv 2013;27(1):3‑9.

7. Zablotska LB, Lane RSD, Frost SE, Thompson PA. Leukemia, lymphoma and multiple myeloma mortality (1950-1999) and incidence (1969-1999) in the Eldorado uranium workers cohort. Environ Res. avr 2014;130:43‑50.

8. Navaranjan G, Berriault C, Do M, Villeneuve PJ, Demers PA. Cancer incidence and mortality from exposure to radon progeny among Ontario uranium miners. Occup Environ Med. déc 2016;73(12):838‑45.

9. Möhner M, Lindtner M, Otten H, Gille HG. Leukemia and exposure to ionizing radiation among German uranium miners. American Journal of Industrial Medicine. 2006;49(4):238‑48.

10. Walsh L, Dufey F, Tschense A, Schnelzer M, Grosche B, Kreuzer M. RADON AND THE RISK OF CANCER MORTALITY—INTERNAL POISSON MODELS FOR THE GERMAN URANIUM MINERS COHORT. Health Physics. sept 2010;99(3):292.

11. Kelly-Reif K, Sandler DP, Shore D, Schubauer-Berigan MK, Troester MA, Nylander-French L, et al. Radon and cancer mortality among underground uranium miners in the Příbram region of the Czech Republic. Am J Ind Med. oct 2020;63(10):859‑67.

12. Zablotska LB, Lane RSD, Frost SE. Mortality (1950–1999) and cancer incidence (1969–1999) of workers in the Port Hope cohort study exposed to a unique combination of radium, uranium and γ-ray doses. BMJ Open. 1 janv 2013;3(2):e002159.

13. Turner MC, Krewski D, Chen Y, Pope CA III, Gapstur SM, Thun MJ. Radon and Nonrespiratory Mortality in the American Cancer Society Cohort. American Journal of Epidemiology. 1 nov 2012;176(9):808‑14.

14. Boz S, Berlin C, Kwiatkowski M, Bochud M, Bulliard JL, Zwahlen M, et al. A prospective cohort analysis of residential radon and UV exposures and malignant melanoma mortality in the Swiss population. Environ Int. nov 2022;169:107437.

15. Kelly-Reif K, Sandler DP, Shore D, Schubauer-Berigan M, Troester M, Nylander-French L, et al. Lung and extrathoracic cancer incidence among underground uranium miners exposed to radon progeny in the Příbram region of the Czech Republic: a case-cohort study. Occup Environ Med. févr 2022;79(2):102‑8.

16. Lane RSD, Frost SE, Howe GR, Zablotska LB. Mortality (1950–1999) and Cancer Incidence (1969–1999) in the Cohort of Eldorado Uranium Workers. rare. oct 2010;174(6a):773‑85.

17. Kreuzer M, Dufey F, Marsh JW, Nowak D, Schnelzer M, Walsh L. Mortality from cancers of the extra-thoracic airways in relation to radon progeny in the Wismut cohort, 1946-2008. Int J Radiat Biol. nov 2014;90(11):1030‑5.

18. Drubay D, Ancelet S, Acker A, Kreuzer M, Laurier D, Rage E. Kidney cancer mortality and ionizing radiation among French and German uranium miners. Radiat Environ Biophys. 1 août 2014;53(3):505‑13.

19. Silver SR, Bertke SJ, Hein MJ, Daniels RD, Fleming DA, Anderson JL, et al. Mortality and ionising radiation exposures among workers employed at the Fernald Feed Materials Production Center (1951–1985). Occup Environ Med. 1 juill 2013;70(7):453‑63.

20. Kreuzer M, Sogl M, Brüske I, Möhner M, Nowak D, Schnelzer M, et al. Silica dust, radon and death from non-malignant respiratory diseases in German uranium miners. Occup Environ Med. 1 déc 2013;70(12):869‑75.

21. Kelly-Reif K, Bertke S, Daniels RD, Richardson DB, Schubauer-Berigan MK. Nonmalignant respiratory disease mortality in male Colorado Plateau uranium miners, 1960-2016. Am J Ind Med. oct 2022;65(10):773‑82.

22. Kreuzer M, Grosche B, Schnelzer M, Tschense A, Dufey F, Walsh L. Radon and risk of death from cancer and cardiovascular diseases in the German uranium miners cohort study: follow-up 1946–2003. Radiat Environ Biophys. 1 mai 2010;49(2):177‑85.

23. Villeneuve PJ, Morrison HI, Volesky K, Lane RSD. Circulatory system disease mortality and occupational exposure to radon progeny in the cohort of Newfoundland Fluorspar Miners between 1950 and 2016. Int Arch Occup Environ Health. 1 avr 2023;96(3):411‑8.

24. Rage E, Caër-Lorho S, Laurier D. Low radon exposure and mortality among Jouac uranium miners An update of the French cohort (1946-2007). Journal of Radiological Protection. mars 2018;38(1):92‑108.
